# Phenotyping cotton leaf chlorophyll via *in situ* hyperspectral reflectance sensing, spectral vegetation indices, and machine learning

**DOI:** 10.3389/fpls.2024.1495593

**Published:** 2024-11-21

**Authors:** Kelly R. Thorp, Alison L. Thompson, Matthew T. Herritt

**Affiliations:** ^1^ United States Department of Agriculture (USDA), Agricultural Research Service (ARS), Grassland Soil and Water Research Laboratory, Temple, TX, United States; ^2^ United States Department of Agriculture (USDA), Agricultural Research Service (ARS), U.S. Arid-Land Agricultural Research Center, Maricopa, AZ, United States; ^3^ United States Department of Agriculture (USDA), Agricultural Research Service (ARS), Wheat Health, Genetics, and Quality Research Unit, Pullman, WA, United States

**Keywords:** chlorophyll, cotton, high-throughput, machine learning, mapping population, phenomics, spectroradiometer, spectral index

## Abstract

Cotton (*Gossypium hirsutum* L.) leaf chlorophyll (Chl) has been targeted as a phenotype for breeding selection to improve cotton tolerance to environmental stress. However, high-throughput phenotyping methods based on hyperspectral reflectance sensing are needed to rapidly screen cultivars for chlorophyll in the field. The objectives of this study were to deploy a cart-based field spectroradiometer to measure cotton leaf reflectance in two field experiments over four growing seasons at Maricopa, Arizona and to evaluate 148 spectral vegetation indices (SVI’s) and 14 machine learning methods (MLM’s) for estimating leaf chlorophyll from spectral information. Leaf tissue was sampled concurrently with reflectance measurements, and laboratory processing provided leaf Chl *a*, Chl *b*, and Chl *a+b* as both areas-basis (µg cm^-2^) and mass-basis (mg g^-1^) measurements. Leaf reflectance along with several data transformations involving spectral derivatives, log-inverse reflectance, and SVI’s were evaluated as MLM input. Models trained with 2019–2020 data performed poorly in tests with 2021–2022 data (e.g., RMSE=23.7% and r^2^ = 0.46 for area-basis Chl *a+b*), indicating difficulty transferring models between experiments. Performance was more satisfactory when training and testing data were based on a random split of all data from both experiments (e.g., RMSE=10.5% and r^2^ = 0.88 for area basis Chl *a+b*), but performance beyond the conditions of the present study cannot be guaranteed. Performance of SVI’s was in the middle (e.g., RMSE=16.2% and r^2^ = 0.69 for area-basis Chl *a+b*), and SVI’s provided more consistent error metrics compared to MLM’s. Ensemble MLM’s which combined estimates from several base estimators (e.g., random forest, gradient booting, and AdaBoost regressors) and a multi-layer perceptron neural network method performed best among MLM’s. Input features based on spectral derivatives or SVI’s improved MLM’s performance compared to inputting reflectance data. Spectral reflectance data and SVI’s involving red edge radiation were the most important inputs to MLM’s for estimation of cotton leaf chlorophyll. Because MLM’s struggled to perform beyond the constraints of their training data, SVI’s should not be overlooked as practical plant trait estimators for high-throughput phenotyping, whereas MLM’s offer great opportunity for data mining to develop more robust indices.

## 1 Introduction

Optimizing leaf chlorophyll is an important cotton (*Gossypium hirsutum* L.) breeding goal (Turley and Pettigrew, 2011; Thompson et al., 2022). As the primary light-harvesting pigment in plants, chlorophyll is responsible for driving photosynthesis and producing photosynthate for plant respiration. Naturally, reduced chlorophyll content limits the energy available for plant growth and marketable yield. On the other hand, excessive chlorophyll can lead to excess energy production in environments where light absorption exceeds capacity for photosynthesis. To avoid harmful effects, the plant must dissipate the excess energy using non-photochemical quenching. Breeding cotton cultivars with focus on leaf chlorophyll can potentially increase cotton production through increased photosynthetic efficiency and greater tolerance to environmental stress. However, progress is currently limited by lack of appropriate screening tools to rapidly evaluate cultivars in the field (Singh et al., 2007).

Traditionally, plant scientists have measured leaf chlorophyll via tissue sampling procedures coupled with analytical methods in the laboratory (Porra et al., 1989). However, these measurements are often laborious and time-consuming, which limits their effectiveness for rapid estimation of chlorophyll content among entries in large breeding populations. In the last decade, high-throughput phenotyping (HTP) technologies have been developed, which couple the deployment of electronic sensing systems and use of automated data workflows to enable rapid estimation of plant traits in the field (White et al., 2012; Pabuayon et al., 2019; Gill et al., 2022). Not only do HTP technologies offer a screening tool for plant breeding decisions but also the data can be used in genomic analyses to better elucidate the links between plant genetics and expression of plant traits in field environments. Solving the latter problem is considered a fundamental step toward enabling agricultural production to feed a growing human population with the complication of climate uncertainty in the current century (White et al., 2012).

Leaf chlorophyll, among other leaf characteristics, is known to influence the reflectance of light from plant tissue, due to the absorbance of visible light radiation by the chlorophyll molecule (Knipling, 1970). Therefore, HTP methods for estimating leaf chlorophyll have typically utilized hyperspectral sensing technology (Grybowski et al., 2021; Sarić et al., 2021), which provides measurements of reflected radiation from plant leaves or plant canopies in narrow, contiguous wavebands. These efforts in HTP build on a half century of research in remote sensing science and more than three decades of research in precision agriculture, where chlorophyll estimation was also relevant for diverse applications in ecosystem monitoring (Zarco-Tejada et al., 2001; Le Maire et al., 2008; Kalacska et al., 2015; Inoue et al., 2016) and crop nutrient management (Daughtry et al., 2000; Gitelson et al., 2005; Schlemmer et al., 2005; Zhu et al., 2020). Continued efforts are needed to advance the science of chlorophyll estimation using modern proximal and remote sensing equipment and by developing reliable data analysis pipelines.

Considerable research effort has been expended to develop spectral vegetation indices and machine learning methods to estimate chlorophyll from vegetative spectral reflectance measurements. A concurrent literature search (Thorp, 2024) identified 148 spectral vegetation indices reported in scientific literature from 1968 to the present time, many of which were developed specifically to estimate chlorophyll (**Supplementary Tables S.1, S.2**). Routinely, researchers consider only a small subset of available indices, but more comprehensive assessments of indices are needed. Some spectral vegetation indices are simple mathematical formulae that combine spectral data from two or three wavebands (Chappelle et al., 1992; Carter, 1994), while others are more complex and require, for example, derivative transformations of hyperspectral data prior to index computation (Filella and Peñuelas, 1994; Elvidge and Chen, 1995). Machine learning is often considered a novel and modern technology, although remote sensing literature suggests that principal component analyses were being used to evaluate spectral reflectance data more than forty years ago (Jackson, 1983; Heute et al., 1984). Nonetheless, modern computational paradigms that provide accessible machine learning algorithms via open-source packages in Python and R (Mevik and Wehrens, 2007; Pedregosa et al., 2011) have enabled novel capabilities for construction of advanced models to relate spectral reflectance data to leaf chlorophyll and other plant traits. A common complaint, however, is that machine learning algorithms are “black box” methods that know nothing of physiological mechanisms (Grybowski et al., 2021), while spectral vegetation indices often have a physiological basis. Further research is needed to expand the development and evaluation of machine learning methods and to identify the advantages and disadvantages of such algorithms as compared to simpler spectral vegetation index approaches.

Recent literature has demonstrated many HTP studies based on combining hyperspectral sensing, spectral vegetation indices, and machine learning methods. For example, Thorp et al. (2015) used a field spectroradiometer to measure reflected radiation from the canopies of 25 cotton cultivars grown under well-watered and water-limited conditions in Arizona. Among several data analysis methodologies including spectral vegetation indices, partial least squares regression (PLSR), and Prosail model inversion, PLSR estimated leaf chlorophyll best with a root mean squared error of 13.1%. Similarly, Yendrek et al. (2017) used a field spectroradiometer with a leaf clipping device to measure *in situ* leaf reflectance for greenhouse- and field-grown maize (*Zea mays* L.) in Illinois. The PLSR method outperformed spectral vegetation indices in the estimation of chlorophyll content with coefficients of determination (r^2^) of 0.81–0.85 for PLSR as compared to <0.75 for spectral indices. Also, Meacham-Hensold et al. (2020) installed a hyperspectral imaging system on a manual field cart to measure reflected radiation from field-grown tobacco (*Nicotiana tabacum* L.) plants in Illinois. The spectral data were used to estimate photosynthetic parameters and leaf pigment contents via PLSR, and chlorophyll content was estimated with an r^2^ of 0.87. Bhadra et al. (2020) used the Transportation Energy Resource from Renewable Agriculture, Phenotyping Reference Platform (TERRA-REF) field scanner at Maricopa, Arizona to measure reflected radiation from a sorghum (*Sorghum bicolor* (L.) Moench) population and used several spectral vegetation indices and machine learning methods to estimate leaf chlorophyll. Their results demonstrated that support vector regression (SVR) with reflectance inputs and extreme learning regression (ELR) with spectral index inputs were the best performing models. Grybowski et al. (2021) described several current challenges for advancing hyperspectral and machine learning technologies for HTP: 1) assessing the ability of trained regression models to perform adequately for estimating plant traits in different years or locations, 2) evaluating additional machine learning algorithms to limit overreliance on PLSR modeling, and 3) using feature selection methodologies to improve understanding of physiological mechanisms for vegetative reflectance of radiation. The present study aimed to address all three of these issues.

A common complaint against HTP is its primary focus on technology development without adequate feedback from breeders on whether the technology is useful and practical (Deery and Jones, 2021). Research activities that directly pair technology experts with breeding experts are needed for development of practical approaches to incorporate HTP technologies into breeding programs. In the present study, the two primary authors represent this dichotomy of expertise, and they jointly pursued field-based cotton HTP research at Maricopa, Arizona for 7+ years. The last four years of effort has led to the research described elsewhere and herein, where the overall vision was to evaluate a cart-based hyperspectral sensing system (Thompson et al., 2023) for field phenotyping of leaf chlorophyll among cotton varieties to provide data for breeding selection and genomic analysis. The main goal of the present study was to evaluate data processing methodologies to estimate cotton leaf chlorophyll from hyperspectral data. Specific objectives were to 1) collect cotton leaf spectral reflectance data and corresponding leaf tissue samples for chlorophyll extraction over four growing seasons of cotton field trials, 2) evaluate existing spectral vegetation indices and machine learning methods for estimating cotton leaf chlorophyll from the leaf spectral reflectance data, and 3) determine the importance of various spectral indices and reflectance wavebands for estimating cotton leaf chlorophyll using machine learning.

## 2 Materials and methods

### 2.1 Field experiments

Two cotton field experiments were conducted at the Maricopa Agricultural Center (MAC) in Maricopa, Arizona, USA (33.079°N, 111.977°W, 360 m above sea level) over four cotton growing seasons from 2019 to 2022. In the first two years (2019–2020), the field trial was arranged in a (0-1) alpha lattice design with six cotton entries, two planting dates, three irrigation treatments, and three replicates per entry for each irrigation by planting date treatment. Five entries were chosen from the National Cotton Variety Test (NCVT) program and the sixth was a red cotton variety. Cotton was planted into raised beds with row spacing of 1.02 m on 16 April (DOY 106) and 17 May (DOY 137) in 2019 and 23 April (DOY 114) and 14 May (DOY 135) in 2020. A subsurface drip irrigation system was installed under the raised beds at a depth of 20 cm with an emitter spacing of 30 cm. After establishing the crop with furrow flood irrigation, three irrigation rates were initiated using the drip irrigation system on 6 May 2019 (DOY 126) and 14 May 2020 (DOY 135) for the first planting and 4 June 2019 (DOY 155) and 29 May 2020 (DOY 150) for the second planting. Irrigation was applied at 60%, 80%, and 100% of the recommended irrigation rate for full production, based on calculations from an irrigation scheduling tool that estimated evapotranspiration and soil water balance (Hunsaker et al., 2005). Plots were 10.6 m in length with 2.3 m alleys between plots. The total experiment area was 0.46 ha and located in Field 119 at MAC.

In the latter two years (2021–2022), the field trial was arranged in an augmented block design with 379 cotton entries and two replicates per entry. The entries were from a recombinant inbred line population with parents that differed in chlorophyll content in wet and dry environments. Cotton was planted into conventionally tilled and planed soil with row spacing of 1.02 m on 22 April 2021 and 18–19 April 2022. Plots were 6.1 m long with 1.2 m alleys. The total experiment area was 0.92 ha and located in Field 13 Bench 4 at MAC. An overhead lateral-move sprinkler irrigation system was used to irrigate the field, with irrigation rates calculated using a newer formulation of the irrigation scheduling tool (Thorp, 2022). The 2021–2022 field site was adjacent to another cotton field trial described by Thorp (2023), and further details on the experimental conditions and irrigation system were provided there.

The soil at both field sites was classified as a Casa Grande sandy loam. Typical agronomic practices for nitrogen fertilizer, insecticide, and defoliant applications were used, but plot areas were manually weeded due to glyphosate non-resistance among the cotton varieties.

### 2.2 Field data collections

**Figure 1:**
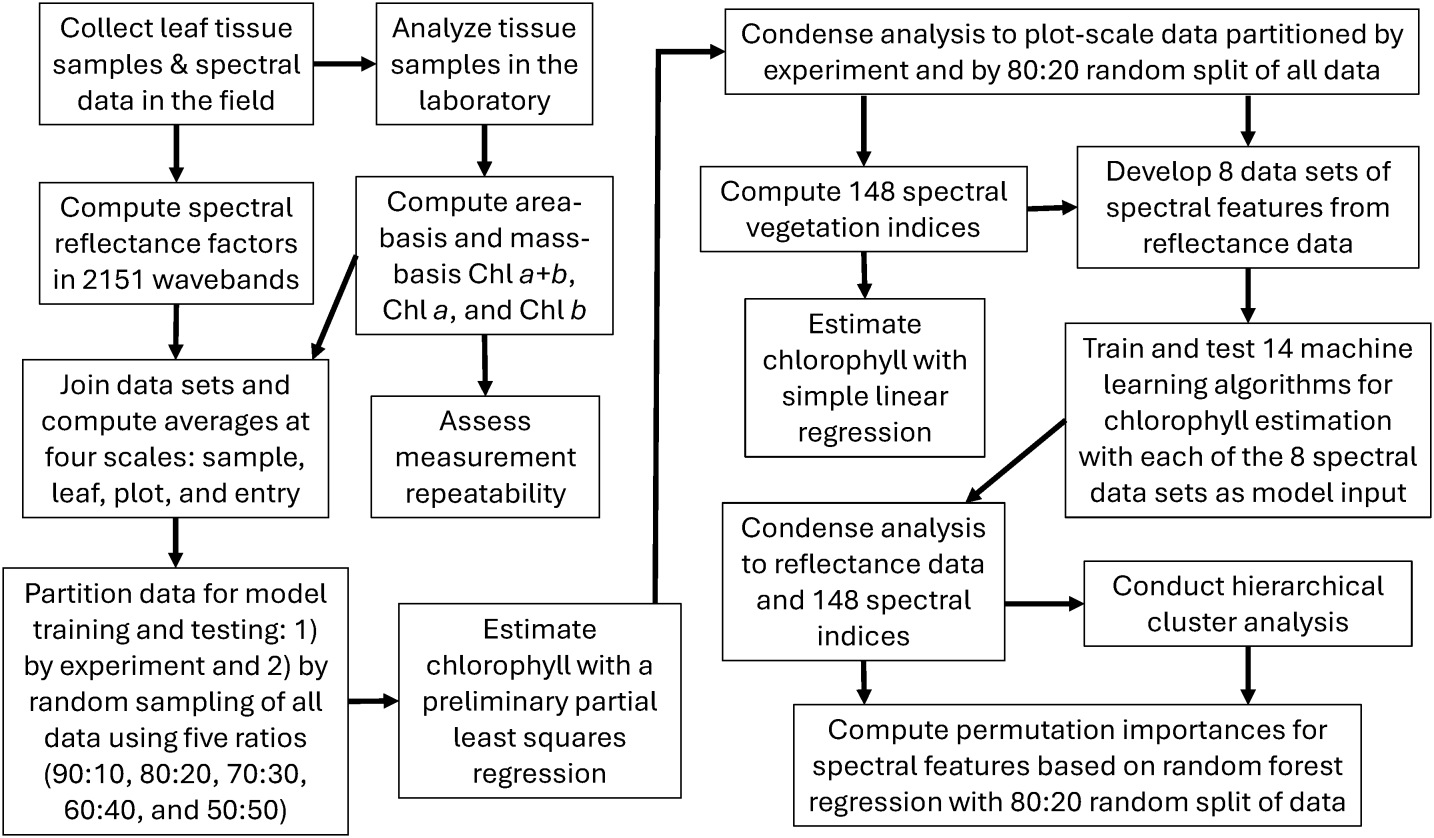
Flow chart of data collection and analysis activities.

Field data collections were scheduled monthly to measure cotton leaf spectral reflectance and to obtain leaf tissue for chlorophyll extraction (**Table 1**; **Figure 1**). Nine collections were conducted during the 2019–2020 field study (four in 2019 and five in 2020), and six collections were conducted during the 2021–2022 field study (three per season). In 2019–2020, each collection was completed in one day; however, two days were typically required to complete collections in 2021–2022. Leaves for sampling were identified from uppermost fully expanded leaves in the canopy on each collection date. Leaves were pre-marked with paint markers ahead of the data collection crew, and flags were temporarily placed in the soil to help the crew quickly find the leaves previously chosen for sampling.

**Table 1:** Schedule for field data collections during cotton field experiments in 2019–2020 and 2021–2022 at Maricopa, Arizona, USA, including the day of year (DOY), the days after planting for the first and second cotton plantings (DAP1 and DAP2, respectively), and the cumulative heat units (°C d) since the first and second cotton plantings (HUP1 and HUP2, respectively).

Collection	Date	DOY	DAP1	DAP2	HUP1	HUP2	Growth Stage
2019A	7/5/2019	186	80	49	941	627	First square/flower
2019B	7/26/2019	207	101	70	1275	961	Peak bloom
2019C	8/16/2019	228	122	91	1615	1301	First open boll
2019D	9/6/2019	249	143	112	1952	1639	Maturity
2020A	6/19/2020	171	57	36	757	491	First square
2020B	7/10/2020	192	78	57	1081	815	First flower
2020C	7/31/2020	213	99	78	1432	1165	Peak bloom
2020D	8/21/2020	234	120	99	1779	1512	First open boll
2020E	9/11/2020	255	141	120	2104	1837	Maturity
2021A	5/26/2021 5/27/2021	146 147	34 35	NA	380 391	NA	First leaf/square
2021B	6/30/2021 7/1/2021	181 182	69 70	NA	900 916	NA	First flower
2021C	7/28/2021 7/29/2021	209 210	97 98	NA	1346 1362	NA	Peak bloom
2022A	6/15/2022	166	57	NA	712	NA	First square/flower
2022B	7/13/2022 7/21/2022	194 202	85 93	NA	1157 1293	NA	Peak bloom
2022C	8/24/2022 8/25/2022	236 237	127 128	NA	1835 1851	NA	First open boll

#### 2.2.1 Leaf spectral reflectance

Radiometric measurements of cotton leaves were collected using a portable field spectroradiometer (ASD FieldSpec 3, Malvern Panalytical Ltd., Malvern, United Kingdom). Radiometric information was reported in 2151 narrow wavebands from 350 to 2500 nm with bandwidth of 1 nm. The instrument’s 1.0 m fiber optic cable was fitted with a contact probe and a leaf clipping device for non-destructive, *in situ* radiometric measurements of cotton leaves. The probe featured a 4.5-W halogen light source and measured leaf radiance independent of external lighting conditions. Two reflectance standards (one made of white polytetrafluoroethylene material and the other made of black painted vinyl) were incorporated with the leaf clip and were easily interchanged to alternate between white reference measurements and dark background for leaf measurements. Thompson et al. (2023) described a novel proximal sensing cart for spectroradiometer transport through the field. The cart also included a customized cooling device for maintaining the spectroradiometer at 27°C in field conditions. As compared to manually transporting the spectroradiometer in the backpack provided by the manufacturer, the cart-based system facilitated data collections, enhanced spectroradiometer performance, and improved operator safety for the collections required in this study. It also permitted increases in the number of collection dates and the number of sampled leaves per collection, as compared to previous phenomics studies at MAC (Thorp et al., 2015; Melandri et al., 2021).

For the 2019–2020 study, spectral measurements were collected from three leaves per plot, but only one leaf per plot was measured in the 2021–2022 study due to the increased number of plots for the mapping population. Five spectral scans were collected from each leaf and averaged by the instrument’s control software, and the averaged spectra were saved to one file per leaf. Five to ten spectral measurements of the white reference panel were frequently collected to characterize the light provided by the halogen bulb, bookending the leaf spectral measurements in groups of 18 or less leaves. Following spectral data collection, leaf reflectance factors at each wavelength were computed as the ratio of leaf radiance and the average radiance from the previous set of white reference scans.

#### 2.2.2 Leaf chlorophyll

Immediately following leaf spectral data collection, leaf tissue was extracted from the same leaf using a 0.283 cm^2^ leaf punch. To avoid possible damage previously caused by the spectroradiometer’s leaf clipping device, leaf tissue sampling occurred on a neighboring leaf lobe. For the 2019–2020 field study, two samples containing two 0.283 cm^2^ leaf disks per sample were collected from each leaf, which permitted an analysis of repeatability for the chlorophyll extraction data at leaf scale. For the 2021-2022 field study, only one sample containing two 0.283 cm^2^ leaf disks per sample was collected per leaf. Samples were collected in 1.5 mL safe-lock tubes (Eppendorf, Enfield, CT, USA) and were kept in the dark on ice in the field prior to transport for storage at -80°C in the laboratory. Chlorophyll was extracted using methanol and measured using a fluorescence plate reader as described by Thompson et al. (2022). Chlorophyll concentration (µg mL^-1^) from each sample was calculated following Porra et al. (1989). The data were then adjusted to both area-basis and mass-basis chlorophyll estimates, which were expected to be most applicable for comparison to hyperspectral remote sensing and for quantifying leaf chlorophyll content, respectively. Dividing the chlorophyll concentration by the total leaf area of the sample (0.566 cm^2^) provided chlorophyll on a leaf area basis (µg cm^-2^). After extraction, the leaf discs were dried at room temperature (22.2°C) for 48 hours and then weighed on an analytical balance. Chlorophyll content was then computed as the ratio of chlorophyll concentration and tissue mass (mg g^-1^). The procedure resulted in six chlorophyll metrics for each leaf sample, including area-basis and mass-basis estimates of chlorophyll *a* (Chl *a*), chlorophyll *b* (Chl *b*), and chlorophyll *a+b* (Chl *a+b*) in units of µg cm^-2^ and mg g^-1^, respectively (**Figure 1**). Thompson et al. (2022) described the outlier removal approach used to remove outliers from the chlorophyll data sets.

### 2.3 Field data analysis

An important question for the data analysis involved the choice of data scale (i.e., the level of data averaging prior to analysis). For the 2019–2020 data set, there were four choices for data scaling: sample, leaf, plot, and entry. At sample scale, no averaging was conducted, and chlorophyll data from the two tissue samples per leaf were each paired with identical spectral reflectance data as collected from the leaf. The size (*n*) of the 2019-2020 data set at sample scale was 5832, including data from all nine data collections in two growing seasons. At leaf scale, the chlorophyll data from the two samples per leaf were averaged and paired with the spectral data for that leaf (*n*=2916). At plot scale, chlorophyll data from six leaf tissue samples among three leaves per plot were averaged and paired with average spectral reflectance data from the same three leaves (*n*=972). At entry scale, data were averaged according to the genotype, which eliminated replication in the study (*n=*54). As the choice of scale was expected to affect the modeling results, all four scales were preliminarily examined based on the performance of partial least squares regression (PLSR) models that related each of the six chlorophyll metrics to the leaf reflectance data in 2151 wavebands (**Figure 1**). For these preliminary evaluations, data from the 2019–2020 field study were averaged accordingly and used for model training based on a cross validation approach that divided the data into 10 segments (Mevik and Wehrens, 2007), and data from the 2021–2022 field study was used for model testing. No averaging from sample to leaf and plot scales was required for the 2021–2022 data, because only one chlorophyll sample was collected per leaf and only one leaf was evaluated per plot, which rendered sample, leaf, and plot scales identical in the test data set (*n*=572). At entry scale, data from the 2021–2022 study were averaged according to genotype, which eliminated replication in the study (*n*=513).

Field data from the 2019–2020 study were originally intended for training of models to relate leaf spectral reflectance and chlorophyll, whereas data from the 2021–2022 study were intended for chlorophyll model testing and application to genetic mapping of chlorophyll traits. However, the preliminary PLSR results demonstrated poor performance of model tests, and an alternative strategy was devised as follows. At each of the four scales, data from all four growing seasons were combined, and samples for model training and testing were drawn randomly without replacement. Different fractions of training and testing data were drawn to identify the optimal choice for data partitioning, including 90%, 80%, 70%, 60%, and 50% of samples for training data and 10%, 20%, 30%, 40%, and 50% for testing, respectively. Additional PLSR models were fit to these data using 10-segment cross validation, which revealed the 80% and 20% split for model training and testing data at plot scale as the optimal choice for further analysis.

#### 2.3.1 Spectral data transformations

Remote sensing literature over the past decades has documented several spectral transformations used to condition spectral reflectance (ρ) data prior to analysis. Examples include 1) the first and second derivatives of spectral reflectance (ρ′ and ρ″, respectively; Horler et al., 1983), 2) the base-10 logarithm (log_10_) of the inverse of spectral reflectance and its first and second derivatives (log_10_ ρ^-1^, (log_10_ ρ^-1^)′, and (log_10_ ρ^-1^)″, respectively; Yoder and Pettigrew-Crosby, 1995; Blackburn, 1998), and 3) band depth or continuum removal analysis of reflectance spectra (ρ_CR_; Kokaly and Clark, 1999; Curran et al., 2001; Huang et al., 2004). These six transformations were computed and used along with the original spectral reflectance data for further data analysis. Spectral derivatives were computed using a Savitsky-Golay filter in Python’s ‘SciPy’ package (Virtanen et al., 2020), and continuum removal analysis required use of a convex hull algorithm also in SciPy. Several of the pretreatment calculations were also required for computation of various spectral vegetation indices. Concurrent development of the “vegspec” software package for Python (Thorp, 2024) facilitated the computation of the spectral data transformations for these data sets.

#### 2.3.2 Spectral vegetation indices

As described in the **Supplementary Material** (**Supplementary Tables S.1, S.2**), an exhaustive literature search identified 148 spectral vegetation indices developed in remote sensing science from 1968 to the present time. Of these indices, the normalized difference vegetation index (NDVI; Rouse et al., 1973) has become the most popular and most widely applied spectral index for agricultural applications. Other spectral indices were developed specifically for estimating chlorophyll in plant leaves. For example, Chappelle et al. (1992) developed spectral ratio indices that were highly correlated with Chl *a*, Chl *b*, and carotenoid (Car) concentrations in soybean (*Glycine max* (L.) Merr.) leaves. While many of the indices involve simple waveband ratios or normalized differences, others have more sophisticated formulations for analysis of chlorophyll absorption features in reflectance spectra (Kim et al., 1994; Daughtry et al., 2000; Haboudane et al., 2004). Furthermore, indices by Filella and Peñuelas (1994) and by Gitelson and Merzlyak (1994) involve integrals (i.e., summations) of hyperspectral reflectance (or its derivatives) over a range of wavelengths. Others evaluate reflectance or derivative spectra relative to linear trends between two endpoint wavelengths (Oppelt and Mauser, 2004; Cho and Skidmore, 2006). To comprehensively evaluate the myriad spectral vegetation indices now available, the “vegspec” Python package (Thorp, 2024) was developed and used to compute each of the 148 spectral indices for each measurement of cotton leaf spectral reflectance. Simple linear regression models were developed to estimate each of the six chlorophyll metrics (Chl *a+b*, Chl *a*, and Chl *b* in both µg cm^-2^ and mg g^-1^) using each spectral index as the independent variable (**Figure 1**).

#### 2.3.3 Machine learning

Fourteen machine learning algorithms as implemented in the ‘scikit-learn’ Python package (Pedregosa et al., 2011) were used for supervised regression of the leaf chlorophyll data with spectral reflectance data and its various transformations (**Table 2**). Specifically, the six chlorophyll metrics were each evaluated as dependent variables, and eight spectral data sets were each tested as the independent variables: ρ, ρ′, ρ″, log_10_ ρ^-1^, (log_10_ ρ^-1^)′, (log_10_ ρ^-1^)″, ρ_CR_, and the complete set of 148 spectral indices. Thus, 48 unique model realizations (6 chlorophyll variables × 8 spectral data sets) were established for each of the 14 machine learning methods (**Figure 1**). The 14 regression algorithms (with their corresponding function names in scikit-learn) included the following: 1) ridge regression (Ridge), 2) least absolute shrinkage and selection operator regression (Lasso), 3) Lasso least angle regression (LassoLars), 4) Bayesian ridge regression (BayesianRidge), 5) kernel ridge regression (KernelRidge), 6) support vector machine regression (SVR), 7) nearest neighbors regression (KNeighborsRegressor), 8) Gaussian process regression (GaussianProcessRegressor), 9) partial least squares regression (PLSRegression), 10) decision tree regression (DecisionTreeRegressor), 11) gradient tree boosting regression (GradientBoostingRegressor), 12) random forest regression (RandomForestRegressor), 13) AdaBoost regression (AdaBoostRegressor), and 14) multi-layer perceptron neural network regression (MLPRegressor). Prior to fitting the machine learning models, all data were standardized by removing the mean and scaling to unit variance via the ‘StandardScaler’ method in scikit-learn.

**Table 2:** Hyperparameters (HP) adjusted from default values for the 14 machine learning regression methods in the “scikit-learn” Python package.

Method in “scikit-learn”	Static HP’s	Dynamic HP’s
Ridge	tol=0.0001	alpha
Lasso	none	alpha
LassoLars	eps=0.0001 normalize=False max_iter=1000000	alpha
BayesianRidge	tol=0.0001	none
KernelRidge	none	alpha
SVR	tol=0.0001	C
KNeighborsRegressor	none	n_neighbors
PLSRegression	scale=False	n_components
GaussianProcessRegressor	none	none
DecisionTreeRegressor	none	max_depth min_samples_split min_samples_leaf
GradientBoostingRegressor	none	learning_rate max_depth
RandomForestRegressor	none	n_estimators min_samples_split min_samples_leaf
AdaBoostRegressor	none	n_estimators learning_rate
MLPRegressor	solver=‘lbfgs’	hidden_layer_sizes alpha

Static HP’s were set uniformly among all instances of the machine learning method in this study. Dynamic HP’s were adjusted uniquely for each of 48 trained models with each method. The optimal HP values for each trained model are given in supplementary tables (**Supplementary Tables S.9–S.14**).

Hyperparameter specifications for each machine learning algorithm were achieved using the data sets established for model training. An exploratory approach was used to determine which hyperparameters to adjust and how they were specified. First, the hyperparameters most sensitive for model fitting were identified via manual adjustment of the function arguments to each machine learning method. Some of the sensitive parameters were specified statically among all fitted models for a particular method, and others were dynamically adjusted for each fitted model via further analysis (**Table 2**). For the hyperparameters chosen for dynamic adjustment, the second step involved use of the scikit-learn ‘GridSearchCV’ or ‘RandomSearchCV’ methods with ‘RepeatedKFold’ cross validation to identify optimal hyperparameter values. The *k*-fold cross validation was conducted with 5 splits (*k*=5) of the training data set and repeated 10 times. The objective function for cross validation was based on maximizing the negative root mean squared error between measured and modeled chlorophyll data. After model training was complete, the models were run with optimal hyperparameter values for the data set previously set aside for model testing. All machine learning modeling was conducted on the Ceres high-performance computing infrastructure provided by the USDA-ARS SCINet program.

#### 2.3.4 Statistical analysis

Comparisons among the machine learning model performance results were evaluated by conducting an analysis of variance (ANOVA) on the root mean squared error (RMSE) between measured and modeled leaf chlorophyll for each combination of machine learning algorithm, spectral data transformation approach, and chlorophyll metric. Tukey’s multiple comparisons tests were also conducted to identify which methods performed statistically better than others. The statistical analysis was conducted using the R Project for Statistical Computing software (www.r-project.org).

#### 2.3.5 Feature importance

Assessments of feature importance focused on the spectral reflectance data and the set of 148 spectral indices with a goal to determine which spectral wavebands and which indices were most informative to the machine learning models. The analysis was further simplified by focusing only on the RandomForestRegressor with the 80% and 20% random split of data from both experiments, because this modeling scenario was among the top performers (**Figure 1**) Because the spectral indices represent a half century of research efforts on deriving meaningful features from vegetative spectral reflectance and its derivatives, the analysis of feature importance was expected to further elucidate the best performing indices for chlorophyll estimation. Also, the analysis of reflectance data was expected to elucidate the most important 1 nm wavebands, which could potentially be targeted for development of new indices.

Multicollinearity is known to exist in hyperspectral data sets due to the large correlation between reflectance measurements in neighboring wavebands. Also, many spectral indices have similar formulations, leading to potential for multicollinearity among them. Most machine learning algorithms incorporate techniques to reduce effects of multicollinearity among input features, which improves the robustness of model performance for independent data. For example, Ridge and Lasso regression incorporate a regularization parameter to constrain regression coefficient estimation, avoid overfitting, and improve generalization of models. Likewise, PLSR incorporates a variable reduction strategy similar to principal component analysis to reduce the number of input features and the correlation among them. Also, the random sampling and bagging methodologies of tree-based algorithms like random forest regression tend to limit the chance that correlated features will be considered in the model. These characteristics help machine learning models deal with multicollinearity when used for estimation purposes; however, when the models are used to assess feature importance, multicollinearity remains problematic. When features are colinear, the model receives similar information from each colinear feature, making it difficult to assess the importance of one feature independent from the other one. To address this issue, a hierarchical clustering approach in SciPy was used to develop clusters of similar reflectance data and spectral indices (**Figure 1**). The approach was based on the Ward linkage of a distance matrix from Pearson correlations among the reflectance data (or spectral indices) at plot-level from both field experiments (*n* = 1544), resulting in a dendrogram that demonstrated groups of similar data. New RandomForestRegressor models were then constructed using one random feature from each of the clusters (thereby reducing multicollinearity among input features). Permutation importances were finally computed among the inputted features, which involves computing the reduction in model fit score when values of a feature are permuted (i.e., jumbled randomly). Iterating the feature selection and permutation importance processes 10,000 times finally demonstrated which groups of spectral wavebands or spectral indices were consistently important for estimation of cotton leaf chlorophyll.

## 3 Results

### 3.1 Measurement repeatability

**Figure 2:**
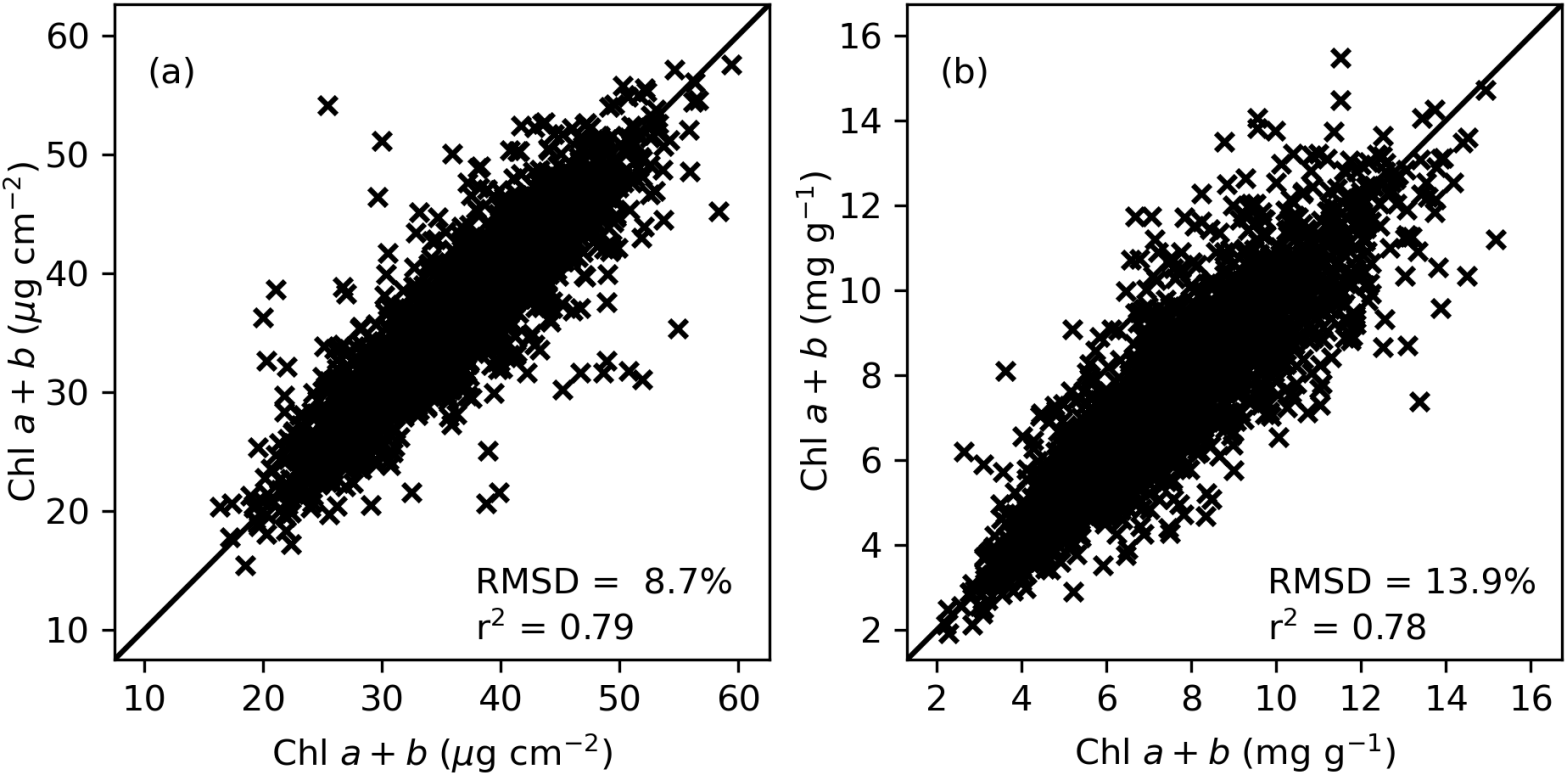
Comparison of cotton leaf chlorophyll *a+b* (Chl *a+b*) extractions among paired tissue samples from the same cotton leaf (*n*=2,916) in units of **(A)** µg cm^-2^ for area-basis estimates and **(B)** mg g^-1^ for mass-basis estimates. Samples were collected during a 2019–2020 field study at Maricopa, Arizona.

Comparisons of Chl *a+b* measurements between paired tissue samples from the same cotton leaf in 2019–2020 revealed coefficients of determination (r^2^) of 0.79 and 0.78 and root mean squared differences (RMSD) of 8.7% and 13.9% for area-basis and mass-basis measurements of Chl *a+b*, respectively (**Figure 2**). The results demonstrated comparisons of paired samples from 2,916 cotton leaves from nine collections in two growing seasons. Similarly, the r^2^ and RMSD statistics for area-basis Chl *a* measurements were 0.80 and 8.4% and for mass-basis Chl *a* were 0.79 and 13.8%, respectively (**Supplementary Figure S.1**). Likewise, the r^2^ and RMSD statistics for area-basis Chl *b* measurements were 0.73 and 15.4% and for mass-basis Chl *b* were 0.71 and 19.7%, respectively (**Supplementary Figure S.2**). These results represent the repeatability of the traditional laboratory-based chlorophyll extraction method for estimating cotton leaf chlorophyll in this study. Furthermore, the results provide important guidance on the expected performance of models developed to estimate chlorophyll from *in-situ* leaf spectral reflectance data. Namely, because the chlorophyll extraction data was used for model fitting and evaluation, the models can likely not be expected to estimate chlorophyll better than the use of a second chlorophyll extraction to estimate the first. Thus, any improvement to model performance reported herein will foremost require improvements in the repeatability of measured data used for model development and evaluation.

**Figure 3:**
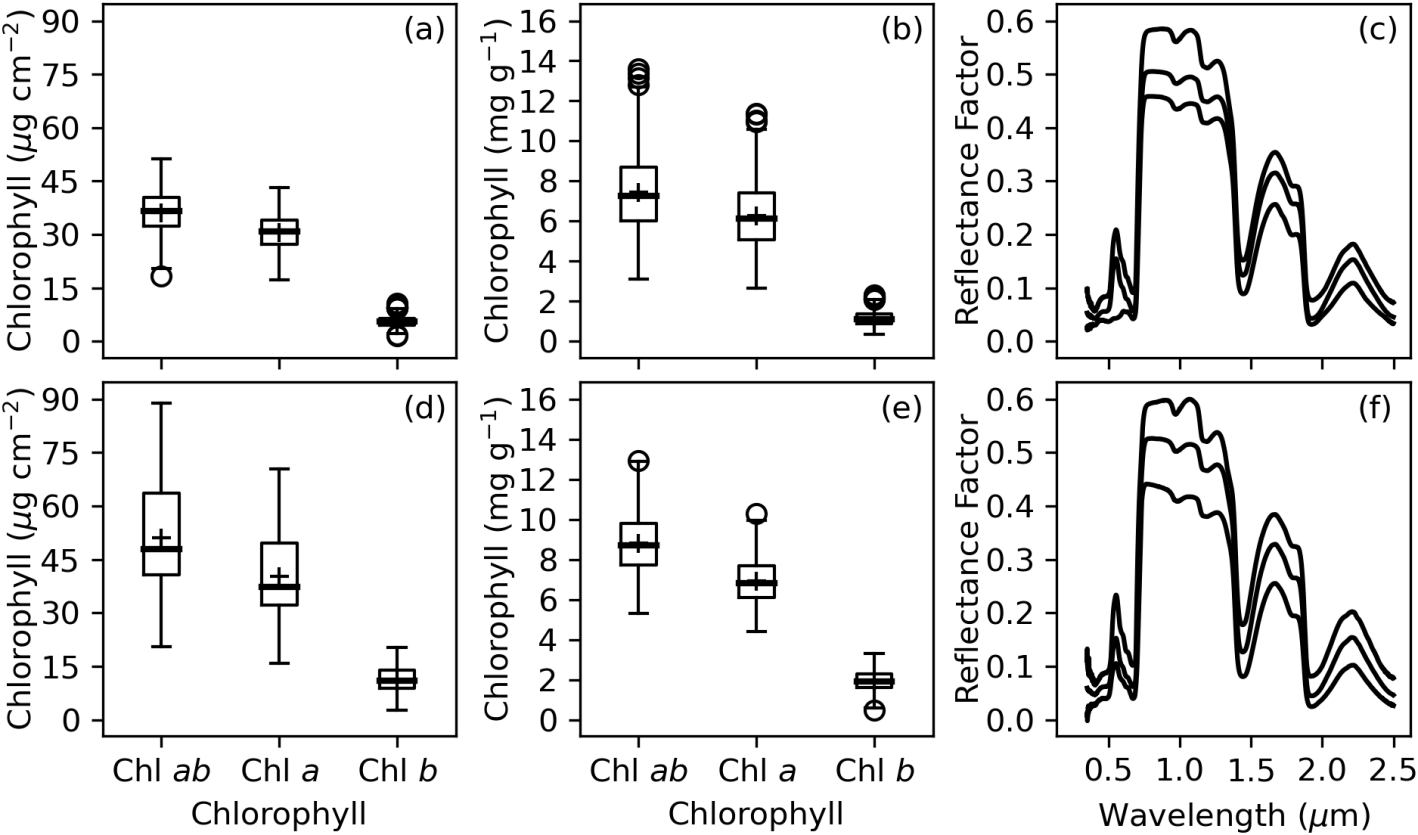
Summary of measured chlorophyll (Chl) and spectral reflectance data collected from cotton leaves during field studies at Maricopa, Arizona, USA, including **(A)** area-basis Chl, **(B)** mass-basis Chl, and **(C)** the minimum, median, and maximum of spectral reflectance data from the 2019–2020 experiment and **(D)** area-basis Chl, **(E)** mass-basis Chl, and **(F)** the minimum, median, and maximum of spectral reflectance data from the 2021–2022 experiment.

Chlorophyll measurements were not particularly similar among the two experiments (**Figure 3**). The interquartile ranges for area-basis Chl *a+b*, Chl *a*, and Chl *b* were 32.3–40.4 µg cm^-2^, 27.3–34.0 µg cm^-2^, and 4.6–6.5 µg cm^-2^, respectively, for the 2019–2020 experiment (**Figure 3A**) and 40.7–63.6 µg cm^-2^, 32.2–49.6 µg cm^-2^, 8.9–13.9 µg cm^-2^ for the 2021–2022 experiment (**Figure 3D**). Similarly, the interquartile ranges for mass-basis Chl *a+b*, Chl *a*, and Chl *b* were 6.0–8.7 mg g^-1^, 5.1–7.4 mg g^-1^, and 0.9–1.4 mg g^-1^, respectively, for the 2019–2020 experiment (**Figure 3B**) and 7.7–9.8 mg g^-1^, 6.1–7.7 mg g^-1^, 1.6–2.3 mg g^-1^ for the 2021–2022 experiment (**Figure 3E**). Welch’s t-tests demonstrated that none of the chlorophyll metrics had equivalent means for the two experiments (*p*<0.05). The leaf spectral reflectance data was more similar for the two experiments (**Figures 3C, F**) with Welch’s t-tests demonstrating equivalent means at 602–613 nm, 691–693 nm, 718–723 nm, 1869–1910 nm, and 2207–2500 nm but unequal means in other wavebands. As discussed later, the differences in chlorophyll data for the two experiments led to performance issues when machine learning models were trained using 2019–2020 data and tested using 2021–2022 data.

### 3.2 Input data considerations

**Figure 4:**
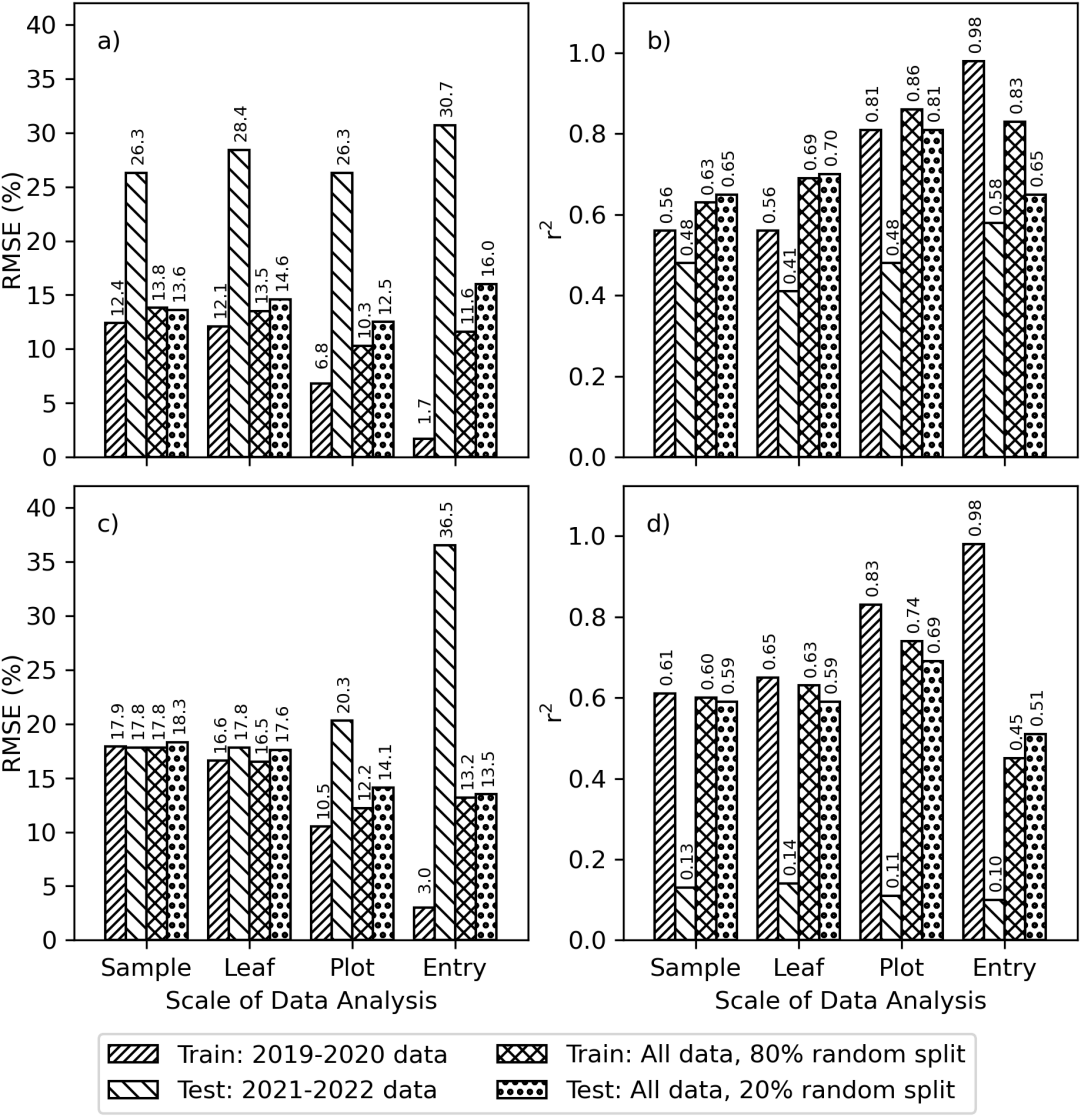
Goodness-of-fit statistics for partial least squares regression (PLSR) models that were fit using cotton leaf chlorophyll *a+b* (Chl *a+b*) and spectral reflectance data at four different scales (i.e., sample, leaf, plot, and entry) and using two methods to split data for training and testing phases (i.e., by experiment and by using an 80% and 20% random split of combined data from both experiments). Results are shown as **(A)** root mean squared errors (RMSE) and **(B)** coefficients of determination (r^2^) for area-basis Chl *a+b* (µg cm^-2^) and **(C)** RMSE and **(D)** r^2^ for mass-basis Chl *a+b* (mg g^-1^).

The r^2^ and RMSE between measured and modeled chlorophyll for PLSR models relating Chl *a+b* and leaf reflectance demonstrated the model performance impacts due to choices for data scaling and for partitioning the training and testing data (**Figure 4**). For area-basis Chl *a+b*, the RMSE’s for model training based on the 2019–2020 data alone improved with additional data averaging: 12.4%, 12.1%, 6.8%, and 1.7% for sample, leaf, plot, and entry scales, respectively (**Figure 4A**). However, the corresponding model tests using data from 2021–2022 alone were poorest with RMSE’s greater than 26.0% no matter the scale. Similarly, the r^2^ for model training based on the 2019–2020 data alone improved with additional averaging: 0.56, 0.56, 0.81, and 0.98 for scale, leaf, plot, and entry data, respectively (**Figure 4B**); however, the r^2^ for model testing using the 2021–2022 data alone were poorest and all less than 0.6. For area-basis Chl *a+b*, the best performing scenario involved plot-scale averaging and an 80% and 20% random split of data from all four years, which provided RMSE’s of 10.3% and 12.5% and r^2^ of 0.86 and 0.81 for training and testing phases, respectively (**Figures 4A, B**). The results were similar for mass-basis Chl *a+b*. When using 2019-2020 data alone for model training, the RMSE’s for mass-basis Chl *a+b* improved with additional data averaging: 17.9%, 16.6%, 10.5%, and 3.0% for sample, leaf, plot, and entry scales respectively (**Figure 4C**). However, model testing performance worsened with these improvements in model training performance. Likewise, the r^2^ for model training based on 2019–2020 data alone improved with additional averaging: 0.61, 0.65, 0.83, and 0.98 for sample, leaf, plot, and entry scales, respectively (**Figure 4D**). However, the model testing performance using 2021-2022 data resulted in r^2^ less than 0.14 no matter the scale. For mass-basis Chl *a+b*, the best performing scenario again involved plot-scale averaging and an 80% and 20% random split of data from all four years, which provided RMSE’s of 12.2% and 14.1% and r^2^ of 0.74 and 0.69 for model training and testing phases, respectively. Model performance results for other fractions of random data splitting (not shown) were often not greatly different from results with the 80% and 20% random split. Also, findings for Chl *a* and Chl *b* (**Supplementary Figures S.3, S.4**) were generally similar to that for Chl *a+b*. The results revealed three considerations for further data analysis: 1) plot-scale data averaging performed optimally in most cases, 2) PLSR models trained using 2019–2020 data were often ineffective for use with the 2021–2022 data, and 3) use of an 80% and 20% random split of data from all years provided consistent and optimal model performance in both training and testing phases. Based on these findings and the reality that most plant breeding and agronomic applications involve plant communities (i.e., crops in field plots), the remainder of the data analysis was performed with plot-scale data averaging. Further comparisons of splitting data by experiment or by using the 80% and 20% random split of data from all years were also performed.

### 3.3 Spectral indices

**Figure 5:**
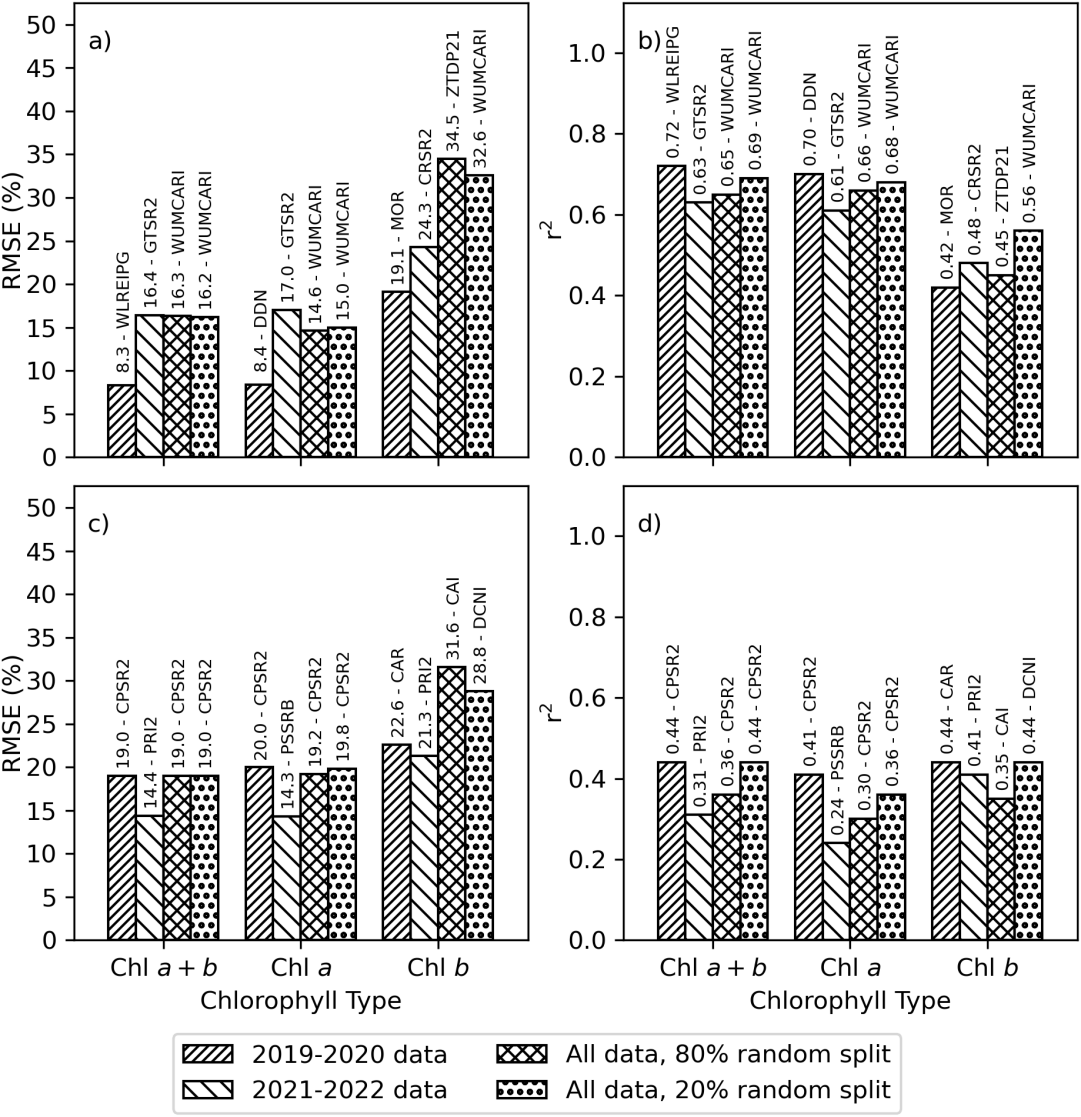
Best-performing spectral vegetation indices for estimating chlorophyll *a+b* (Chl *a+b*), chlorophyll *a* (Chl *a*), or chlorophyll *b* (Chl *b*) using simple linear regression. Data sets were developed from plot-scale averaging of field data and partitioned according to the 2019–2020 experiment, the 2021–2022 experiment, an 80% random split of combined data from 2019–2022, and the remaining 20% random split of combined data from 2019–2022. Results are provided as the **(A)** root mean squared error (RMSE, %) and **(B)** coefficient of determination (r^2^) for estimating area-basis chlorophyll (µg cm^-2^) and **(C)** RMSE and **(D)** r^2^ for estimating mass-basis chlorophyll (mg g^-1^). The spectral index abbreviations are provided in the **Supplementary Material** (**Supplementary Table S.1**).

The best-performing spectral vegetation indices estimated area-basis Chl *a+b* and Chl *a* with RMSE’s less than 17% and r^2^ from 0.61 to 0.72, while area-basis Chl *b* was estimated with RMSE’s less than 35% and r^2^ not exceeding 0.56 (**Figures 5A, B**). Wu’s revision of the modified chlorophyll absorption in reflectance index (WUMCARI; Wu et al., 2008) was notably the best-performing index for area-basis Chl *a+b* and Chl *a* for both the 80% and 20% random splits of the full dataset, as well as for Chl *b* for the 20% random split. For the 2019–2020 data set, the best-performing indices for estimating area-basis Chl *a+b*, Chl *a*, and Chl *b* were, respectively, the wavelength of the red edge inflection point as determined using a Gaussian model fitting approach (WLREIPG; Miller et al., 1990), the new Double Difference index (DDN, Le Maire et al., 2008), and the ratio of the modified chlorophyll absorption in reflectance index and the optimized soil adjusted vegetation index (MOR; Daughtry et al., 2000). For the 2020–2021 data set, a simple ratio of reflectances at 750 and 700 nm (GTSR2 = ρ_750_/ρ_700_; Gitelson and Merzlyak, 1996, 1997) was the best-performing index for estimating Chl *a+b* and Chl *a*, while a simple ratio of reflectances at 605 and 760 nm (CRSR2 = ρ_605_/ρ_760_; Carter, 1994) was the best-performing index for estimating area-basis Chl *b*.

The best-performing spectral vegetation indices estimated mass-basis Chl *a+b* and Chl *a* with RMSE’s less than 20% and mass-basis Chl *b* with RMSE’s less than 32%, while the r^2^ for these estimates ranged from 0.24 to 0.44 (**Figures 5C, D**). Spectral vegetation indices tended to estimate mass-basis chlorophyll more poorly than area-basis chlorophyll. One of Chapelle’s ratio indices (CPSR2 = ρ_675_/(ρ_650_ρ_700_); Chappelle et al., 1992) was notably the best-performing index for estimating mass-basis Chl *a+b* and Chl *a* for the 2019-2020 data set and for both the 80% and 20% random splits of the full data set. For the 2021–2022 data set, best-performing indices for mass-basis Chl *a+b* and Chl *a* were, respectively, Filella’s implementation of the photochemical reflectance index (PRI2 = (ρ_539_-ρ_570_)/(ρ_539_+ρ_570_); Filella et al., 1996) and one of Blackburn’s simple ratio indices (PSSRB = ρ_800_/ρ_635_; Blackburn, 1998). To estimate mass-basis Chl *b*, the best-performing index for the 2019–2020 data set was the chlorophyll absorption in reflectance index (CAR; Kim et al., 1994) and for the 2021–2022 data set was Filella’s PRI2. For the 80% and 20% random splits of the full data set, mass-basis Chl *b* was best estimated by the cellulose absorption integral (CAI) and double-peak canopy nitrogen index (DCNI), neither of which were developed specifically for estimating Chl *b*. The **Supplementary Material** provides a complete listing of simple linear regression performance statistics for all 148 spectral indices for area-basis Chl *a+b* (**Supplementary Table S.3**), area-basis Chl *a* (**Supplementary Table S.4**), area-basis Chl *b* (**Supplementary Table S.5**), mass-basis Chl *a+b* (**Supplementary Table S.6**), mass-basis Chl *a* (**Supplementary Table S.7**), and mass-basis Chl *b* (**Supplementary Table S.8**).

### 3.4 Machine learning

Between zero and three hyperparameters were selected for dynamic adjustment in the training of each machine learning model (**Table 2**). For several of the methods, the regularization parameter (i.e., alpha) was the only adjusted parameter. In fact, only 5 of the 14 methods required adjustment of more than one hyperparameter. For the KNeighborsRegressor and PLSRegression methods, hyperparameters for the number of neighbors and number of components, respectively, were most important. For the DecisionTreeRegressor and RandomForestRegressor methods, hyperparameters that governed the tree size were most sensitive. Likewise, the number of hidden layers was important for the MLPRegressor neural network method. The optimal hyperparameter values for each trained model are provided in the **Supplementary Material** for area-basis Chl *a+b* (**Supplementary Table S.9**), area-basis Chl *a* (**Supplementary Table S.10**), area-basis Chl *b* (**Supplementary Table S.11**), mass-basis Chl *a+b* (**Supplementary Table S.12**), mass-basis Chl *a* (**Supplementary Table S.13**), and mass-basis Chl *b* (**Supplementary Table S.14**). Note that no hyperparameters were dynamically adjusted for the BayesianRidge and GaussianProcessRegressor methodologies.

**Figure 6:**
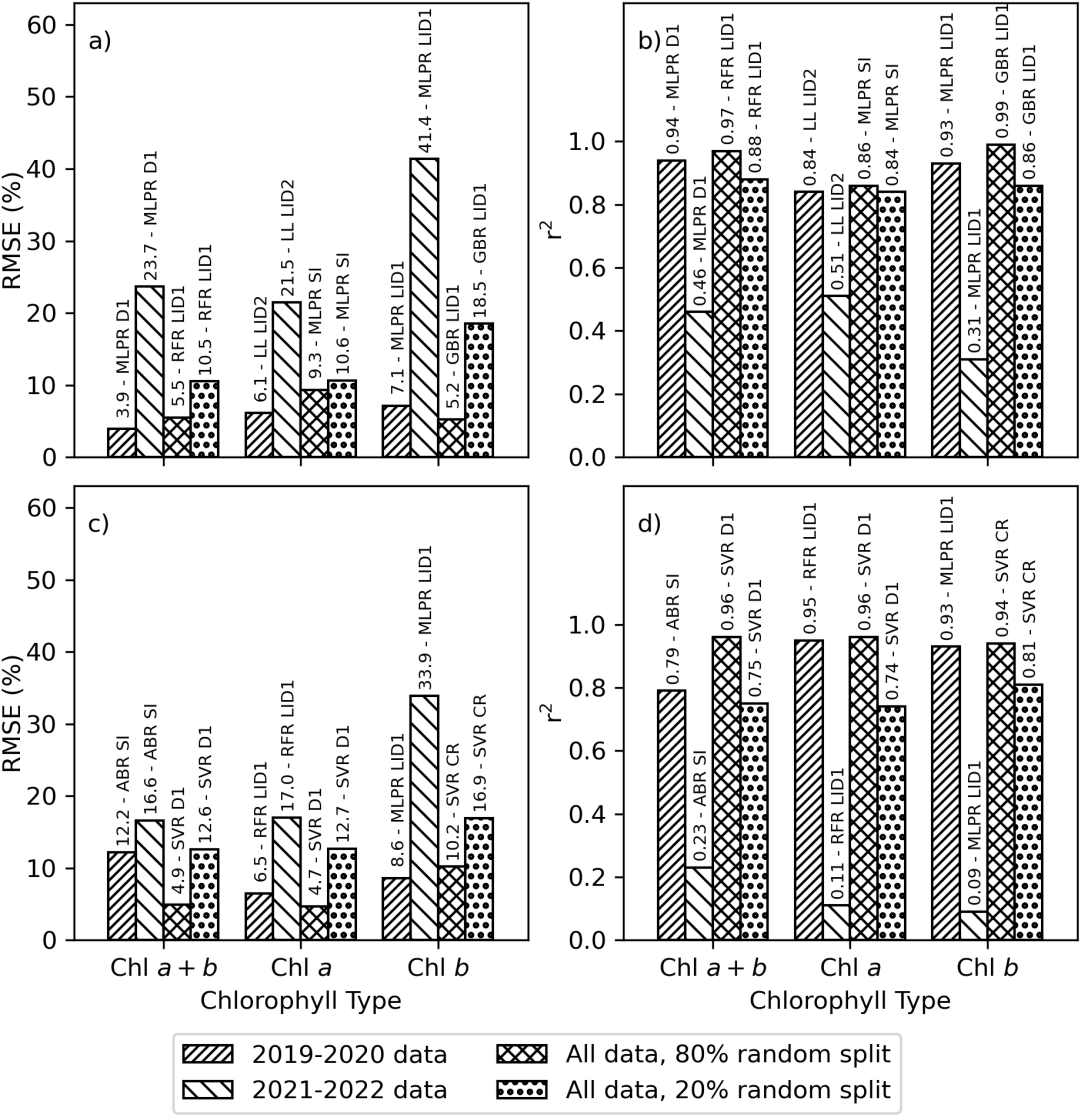
Best-performing machine learning model and spectral data set combinations for estimating chlorophyll *a+b* (Chl *a+b*), chlorophyll *a* (Chl *a*), or chlorophyll *b* (Chl *b*). Data sets were developed from plot-scale averaging of field data and partitioned according to the 2019–2020 experiment, the 2021–2022 experiment, an 80% random split of combined data from 2019–2022, and the remaining 20% random split of combined data from 2019–2022. Results are provided as the **(A)** root mean squared error (RMSE, %) and **(B)** coefficient of determination (r^2^) for estimating area-basis chlorophyll (µg cm^-2^) and **(C)** RMSE and **(D)** r^2^ for estimating mass-basis chlorophyll (mg g^-1^). Best-performing machine learning algorithms included the AdaBoostRegressor (ABR), GradientBoostingRegressor (GBR), LassoLars (LL), MLPRegressor (MLPR), RandomForestRegressor (RFR), and support vector regression (SVR). Best-performing spectral data sets included the first derivative of reflectance (D1), first derivative of log-inverse reflectance (LID1), second derivative of log-inverse reflectance (LID2), continuum removed spectra (CR), and the set of 148 spectral vegetation indices (SI).

The original research plan was to use data from the 2019–2020 experiment for training the machine learning models, while data from the 2021–2022 experiment was reserved for model testing and application. However, poor performance of the machine learning models when tested using 2021–2022 data, likely related to the differences in chlorophyll data among the two experiments (**Figure 3**), demonstrated the fallacy of this initial plan. The best-performing machine learning models estimated area-basis Chl *a+b*, Chl *a*, and Chl *b* with RMSE’s of 3.9%, 6.1%, and 7.1%, respectively, for the 2019–2020 training data, but performance degraded to 23.7%, 21.5%, and 41.4%, respectively, for the 2021–2022 testing data (**Figure 6A**). The r^2^ for these estimates were greater than 0.84 for training data, but less than 0.51 for the testing data (**Figure 6B**). When partitioning data according to the experiments, the best-performing algorithms and spectral data transformations for estimating area-basis Chl *a+b*, Chl *a*, and Chl *b* were, respectively, MLPRegressor with the first derivative of spectral reflectance, LassoLars with the second derivative of the log-inverse of spectral reflectance, and MLPRegressor with the first derivative of the log-inverse of spectral reflectance. Similarly for mass-basis Chl *a+b*, Chl *a*, and Chl *b*, the best performing machine learning models provided estimates with RMSE’s of 12.2%, 6.5%, and 8.6%, respectively, for the 2019–2020 training data, but performance degraded to 16.6%, 17.0%, and 33.9%, respectively, for the 2021–2022 testing data (**Figure 6C**). The r^2^ for these estimates were greater than 0.79 for the training data but less than 0.23 for the testing data (**Figure 6D**). The best-performing algorithms and spectral data transformations for estimating mass-basis Chl *a+b*, Chl *a*, and Chl *b* were, respectively, AdaBoostRegressor with the full set of 148 spectral indices, RandomForestRegressor with the first derivative of log-inverse reflectance, and MLPRegressor with the first derivative of log-inverse reflectance. When comparing results for spectral vegetation indices (**Figure 5**) with that for machine learning algorithms trained with 2019–2020 data and tested with 2021–2022 data (**Figure 6**), the best-performing spectral indices for the 2021–2022 data set each provided better statistical results than the best-performing machine learning algorithms. This means leaf chlorophyll from the 2021–2022 experiment was better estimated using spectral vegetation indices than using machine learning algorithms previously trained with data from the 2019–2020 experiment. As this called into question the original plan for developing the machine learning models, the alternative plan based on random splits of data from both experiments was undertaken.

Improved machine learning model performance was achieved when model training and model testing were based on an 80% and 20% random split, respectively, of all data from both experiments. In this case, the best-performing machine learning models estimated area-basis Chl *a+b*, Chl *a*, and Chl *b* with RMSE’s of 5.5%, 9.3%, and 5.2%, respectively, for the training data, and RMSE’s were 10.5%, 10.6%, and 18.5%, respectively, for the testing data (**Figure 6A**). The r^2^ for these estimates were greater than 0.84 for both the training and testing data sets (**Figure 6B**). When partitioning by the random split of all data, the best-performing algorithms and spectral data transformations for estimating area-basis Chl *a+b*, Chl *a*, and Chl *b* were, respectively, RandomForestRegressor with the first derivative of log-inverse reflectance, MLPRegressor with all 148 spectral indices, and GradientBoostingRegressor with the first derivative of log-inverse reflectance. Similarly for mass-basis Chl *a+b*, Chl *a*, and Chl *b*, the best performing machine learning models provided estimates with RMSE’s of 4.9%, 4.7%, and 10.2%, respectively, for the training data, and RMSE’s were 12.6%, 12.7%, and 16.9%, respectively, for the testing data (**Figure 6C**). The r^2^ for these estimates were greater than 0.74 for both the training and testing data sets. The best-performing algorithm for estimating mass-basis Chl *a+b*, Chl *a*, and Chl *b* was support vector regression (SVR) based on the first derivative of spectral reflectance for Chl *a+b* and Chl *a* and continuum removed spectra for Chl *b*. When comparing results for spectral vegetation indices (**Figure 5**) with that for machine learning algorithms trained and tested via an 80% and 20% random split of data from both experiments (**Figure 6**), the machine learning algorithms outperformed the spectral indices for both the training and testing data sets. While the performance of the machine learning models has improved in this case, the results also raise concern about the robustness of machine learning models when applied even slightly beyond the scope of the data used for their training. The **Supplementary Material** provides a complete listing of performance results for all 14 machine learning algorithms and 8 spectral data sets for estimating area-basis Chl *a+b* (**Supplementary Table S.15**), area-basis Chl *a* (**Supplementary Table S.16**), area-basis Chl *b* (**Supplementary Table S.17**), mass-basis Chl *a+b* (**Supplementary Table S.18**), mass-basis Chl *a* (**Supplementary Table S.19**), and mass-basis Chl *b* (**Supplementary Table S.20**).

**Figure 7:**
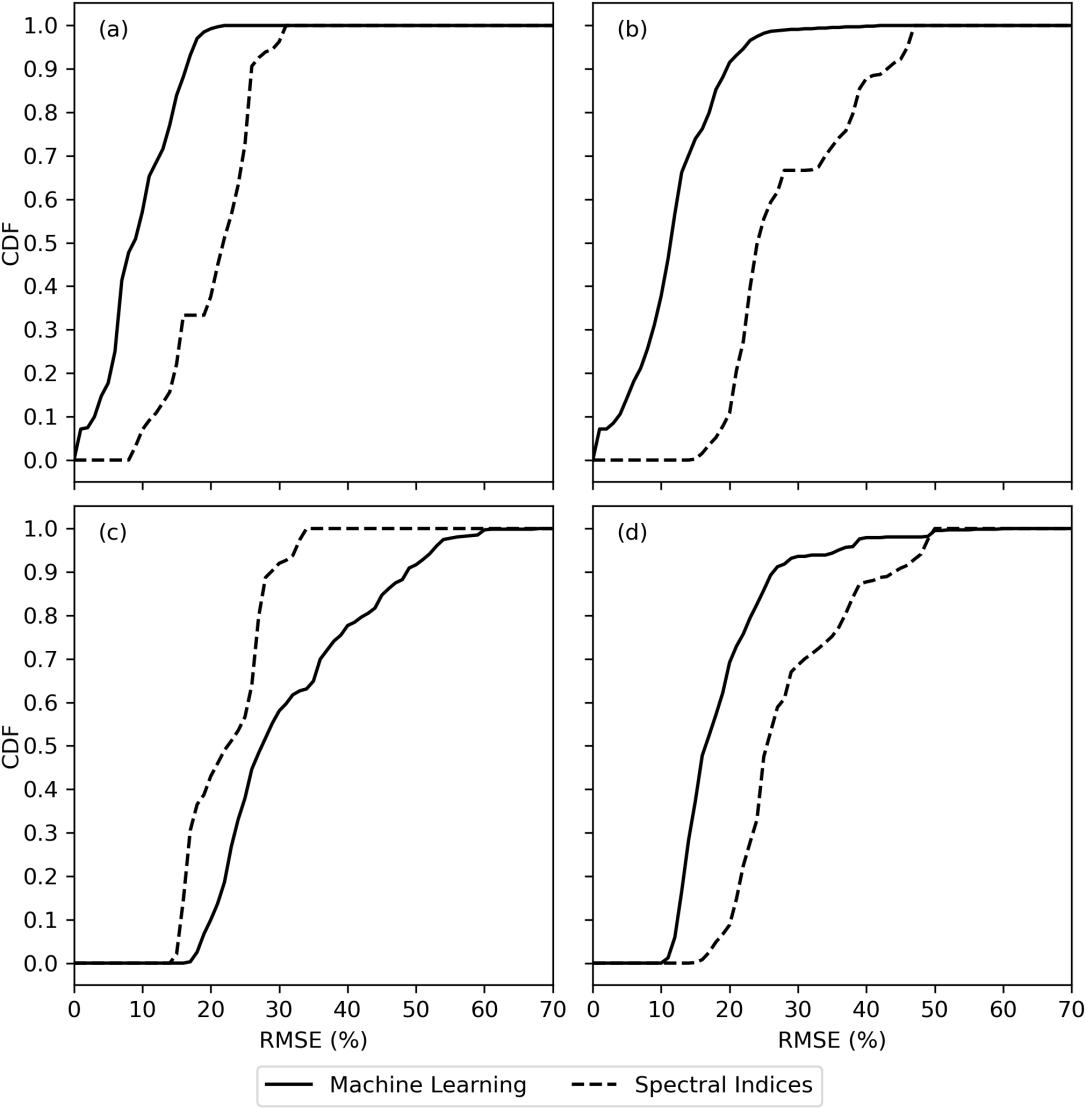
Cumulative distribution functions (CDF) for the root mean square error (RMSE) between measured and modeled cotton leaf chlorophyll among evaluations of machine learning models and spectral vegetation indices for **(A)** model training data from the 2019–2020 field experiment, **(B)** model training data based on an 80% random split of all field data, **(C)** model testing data from the 2021–2022 field experiment, and **(D)** model testing data based on a 20% random split of all field data.

The cumulative distribution functions (CDF’s) of RMSE between measured and modeled cotton leaf chlorophyll among all machine learning models and all spectral vegetation indices further demonstrated the issue with choice of strategy for partitioning the model training and testing data (**Figure 7**). For the data sets used for machine learning model training, the trained models better estimated chlorophyll with lower RMSE as compared to use of spectral vegetation indices and simple linear regression (**Figures 7A, B**). However, for the data sets used for machine learning model testing, results depended on the strategy for partitioning the model training and testing data sets. When partitioning the data according to the 2019–2020 and 2021–2022 field experiments, machine learning models generally did not estimate chlorophyll with lower RMSE than spectral vegetation indices (**Figure 7C**). When partitioning using an 80% and 20% random split of data from both experiments, the opposite result was found (**Figure 7D**). This means machine learning model performance was more dependent on arbitrary choices made by the modeler, while the spectral vegetation indices offered a more consistent performance. For example, the best-performing spectral indices estimated chlorophyll with RMSE from 15% to 20% no matter the data set, while the best-performing machine learning models estimated chlorophyll with RMSE less than 10% for training data sets and from 10%-15% or 20%-25% depending on the choice of data partitioning strategy (**Figure 7**). The results highlight the limitation of machine learning models to perform consistently, particularly for data sets that diverge from data used for model training.

**Figure 8:**
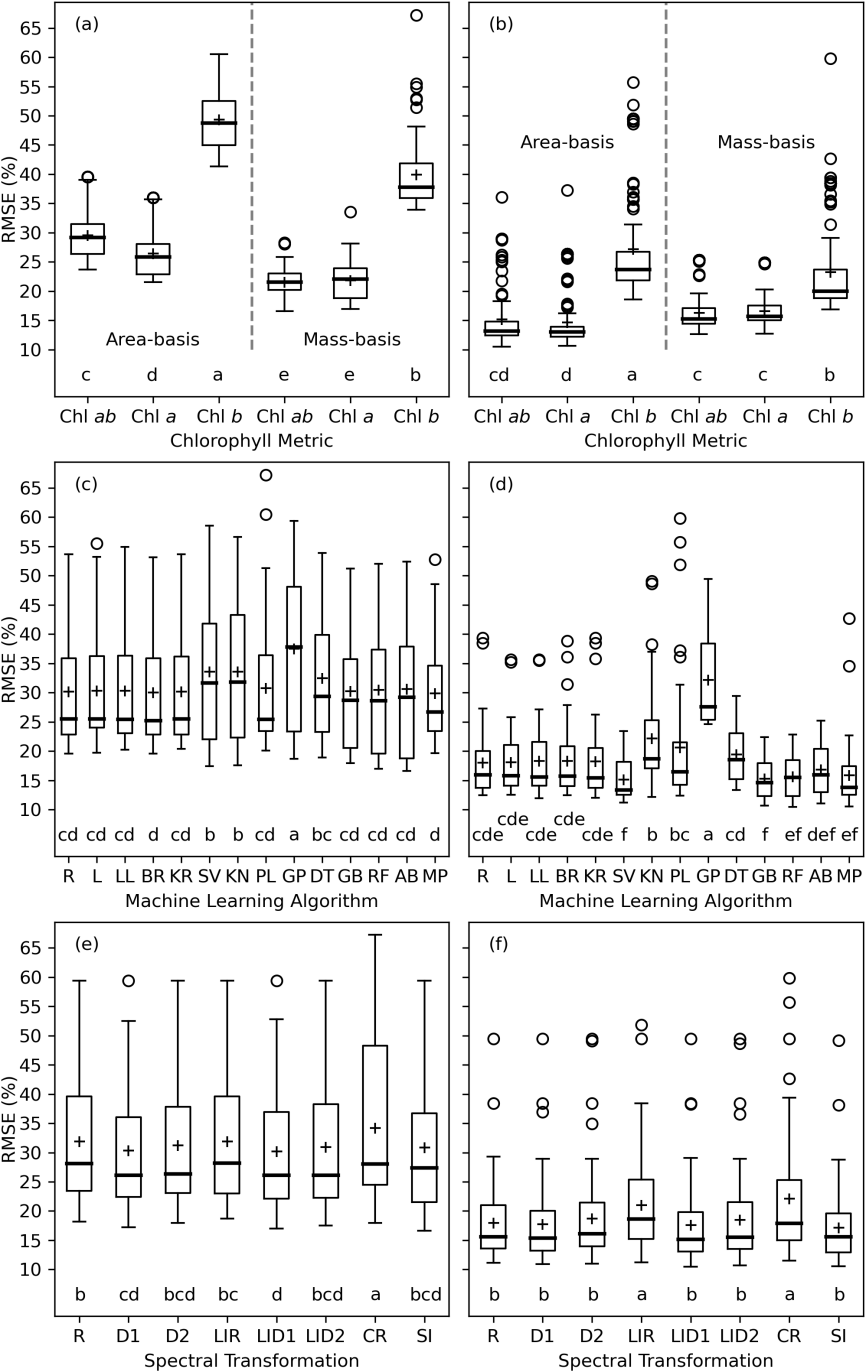
Machine learning model performance expressed as the root mean squared error (RMSE, %) between measured and modeled leaf chlorophyll (Chl) among combinations of 6 chlorophyll metrics **(A, B)**, 14 machine learning algorithms **(C, D)**, and 8 spectral data transformations **(E, F)**. The left column **(A, C, D)** demonstrates model testing results with model training and testing based on data from the 2019–2020 and 2021–2022 field data sets, respectively. The right column **(B, D, F)** demonstrates model testing results with model training and testing based on data from an 80% and 20% random split of data from both field experiments, respectively. Letters below box plots indicate mean separation via Tukey’s multiple comparisons tests. The evaluated machine learning algorithms from Python’s scikit-learn package include Ridge (R), Lasso (L), LassoLars (LL), BayesianRidge (BR), KernelRidge (KR), SVR (SV), KNeighborsRegressor (KN), PLSRegression (PL), GaussianProcessRegressor (GP), DecisionTreeRegressor (DT), GradientBoostingRegressor (GB), RandomForestRegressor (RF), AdaBoostRegressor (AB), and MLPRegressor (MP). The spectral data transformations include reflectance (R), first derivative of reflectance (D1), second derivative of reflectance (D2), log-inverse reflectance (LIR), first derivative of log-inverse reflectance (LID1), second derivative of log-inverse reflectance (LID2), continuum-removed spectra (CR), and 148 spectral vegetation indices (SI).

The analysis of variance for RMSE between measured and modeled cotton leaf chlorophyll indicated that the chlorophyll metric (i.e., area-basis or mass-basis estimates of Chl *a+b*, Chl *a*, and Chl *b*) led most greatly to variability in machine learning model performance, because the *F* statistics were greatest for this factor (**Table 3**). For the training data sets, the machine learning algorithm also contributed substantially to the variability in performance results, but lesser so than the chlorophyll metric. For the testing data sets, the *F* statistic for chlorophyll metric was an order of magnitude greater than that for either the machine learning algorithm or the spectral data transformation. This result was due mainly to the relatively poorer performance of machine learning models to estimate Chl *b* as compared to Chl *a* and Chl *a+b* (**Figure 8**). For machine learning models trained with data from 2019–2020 and tested with data from 2021–2022, model testing demonstrated significantly superior performance for estimating mass-basis Chl *a+b* and Chl *a* as compared to other chlorophyll metrics (**Figure 8A**), followed by area-basis Chl *a*, area-basis Chl *a+b*, mass-basis Chl *b*, and area-basis Chl *b*. However, for machine learning models trained and tested with an 80% and 20% random split of all data, model testing demonstrated superior performance for area-basis Chl *a* and Chl *a+b* (**Figure 8B**), followed by mass-basis Chl *a+b* and Chl *a*, mass-basis Chl *b*, and area-basis Chl *b*. Thus, the performance of machine learning models to estimate area-basis versus mass-basis chlorophyll was also dependent on the arbitrary choice of strategy for determining the model training and testing data sets.

**Table 3:** Analysis of variance results (*F* statistics and *p* values) for the percent root mean squared error between measured and modeled cotton leaf chlorophyll.

		Training Data		Testing Data	
	df	*F*	*p* value	*F*	*p* value
		*————— Split by experiment —————*			
Machine Learning Algorithm	13	229.10	0.0000***	19.46	0.0000***
Spectral Transformation	7	24.54	0.0000***	11.92	0.0000***
Chlorophyll Metric	5	445.86	0.0000***	1179.73	0.0000***
		— *80% and 20% random split of all data* —			
Machine Learning Algorithm	13	202.00	0.0000***	60.45	0.0000***
Spectral Transformation	7	13.20	0.0000***	17.85	0.0000***
Chlorophyll Metric	5	234.40	0.0000***	197.45	0.0000***

Results are provided for two conditions of model training and testing data for machine learning: 1) splitting the data by experiment (i.e., 2019–2020 data for training and 2021–2022 data for testing) and 2) using an 80% and 20% random split of data from both experiments.

Model testing performance among many of the machine learning algorithms were often not statistically different. For models trained with data from 2019–2020 and tested with data from 2021–2022, 10 of 14 algorithms provided statistically similar results among those with the smallest mean RMSE’s, including Ridge, Lasso, LassoLars, BayesianRidge, KernelRidge, PLSRegression, GradientBoostingRegressor, RandomForestRegressor, AdaBoostRegressor, and MLPRegressor (**Figure 6C**). One of the algorithms, GaussianProcessRegression, performed significantly poorer than the others. For models trained and tested with an 80% and 20% random split of all data, 5 of 14 algorithms provided statistically similar results among those with the smallest mean RMSE’s, including SVR, GradientBoostingRegressor, RandomForestRegressor, AdaBoostRegressor, and MLPRegressor (**Figure 8D**). Notably, the methods denoted in scikit-learn as “ensemble” approaches (GradientBoostingRegressor, RandomForestRegressor, and AdaBoostRegressor), which combine the estimates of several base estimators to improve robustness over a single estimator, and also the neural network modeling approach (MLPRegressor) performed well for both ways of specifying model training and testing data. Future analyses could likely be condensed by focusing on these latter four algorithms.

Model testing performance among the 8 spectral data transformations were also often not statistically different. For models trained with data from 2019–2020 and tested with data from 2021–2022, 5 of the 8 spectral transformation provided statistically similar results among those with the smallest mean RMSE’s, including the first and second derivatives of reflectance, the first and second derivatives of log-inverse reflectance, and the set of 148 spectral vegetation indices (**Figure 8E**). Notably, derivative spectra and spectral indices, some of which involve derivative analysis, provided a statistically better performance than raw reflectance or continuum-removed spectra. For models trained and tested with an 80% and 20% random split of all data, log-inverse reflectance and continuum-removed spectra performed significantly more poorly than the other spectral data sets. The favorable performance of the 148 spectral vegetation indices is a positive result due to the large research investment to develop these indices over the past half century. Because the indices theoretically embody a biophysical understanding of light interaction with vegetation, they offer a unique way to synthesize prior knowledge for input to the “black box” machine learning algorithms.

### 3.5 Feature importance

**Figure 9:**
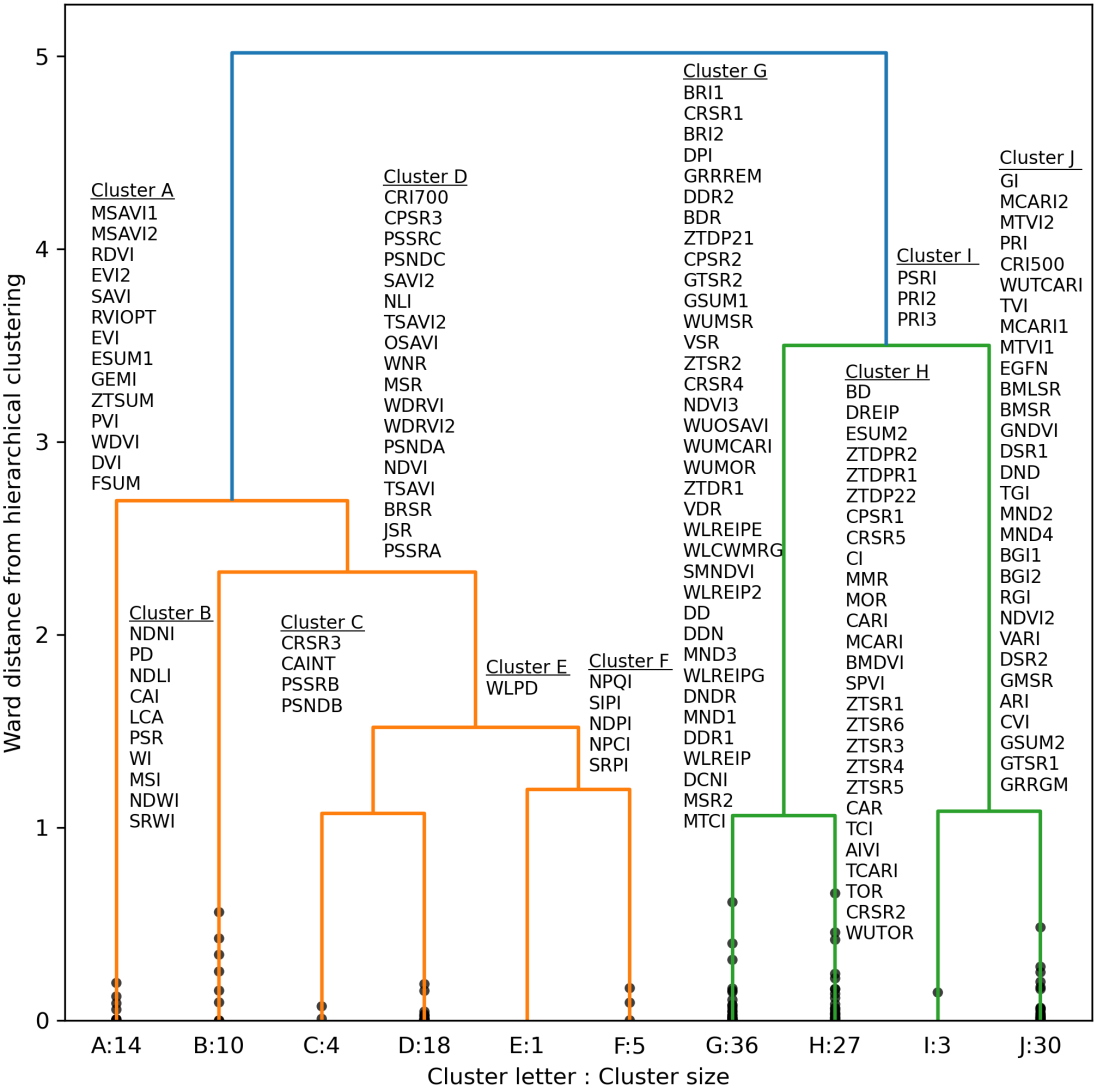
Truncated dendrogram based on hierarchical cluster analysis of 148 spectral vegetation indices computed from cotton leaf reflectance data collected during two field experiments in the 2019–2022 growing seasons at Maricopa, Arizona, USA. To cluster the data, the dendrogram was truncated at a Ward distance of 1.0 and nodes below this value indicate points of further branching for within-cluster indices. Further information on the definition and formulation of each index is provided in the **Supplementary Material** (**Supplementary Tables S.1, S.2**).

A truncated dendrogram from hierarchical clustering of spectral vegetation indices demonstrated 10 groups of similar indices (**Figure 9**). To cluster the indices, the cut-off point was manually selected at a Ward distance of 1.0 with an aim to balance cluster sizes and maximize distance from the cut-off point to the previous node in the tree. Cluster A indices focused on contrasting near-infrared and red radiation at default wavelengths of 670 and 800 nm, respectively, and three indices computed the area of the first derivative peak over a broad 80+ nm waveband straddling the red edge. Cluster B indices used near-infrared and short-wave infrared radiation for estimating contents of water, nitrogen, cellulose, or lignin. Like Cluster A, indices in Clusters C and D focused on contrasts of near-infrared and visible light radiation. Cluster E contained a single index based on the wavelength of the minimum first derivative in the range 900 to 970 nm, developed for plant water status estimation. Cluster F contained indices developed by Peñuelas et al. (1995) for assessing the ratio of carotenoid and Chl *a*. Many indices in Cluster G analyzed the red edge region or included a red edge waveband in the index calculation. For example, all the indices that compute the wavelength of the red edge inflection point were in Cluster G. Also, many of the indices by Wu et al. (2008), which substituted a red edge waveband into the equations of other indices, were in Cluster G. A few additional indices contrast radiation in red and blue visible light wavebands. All the Cluster G indices were developed for the purpose of estimating contents of chlorophyll or nitrogen in plant leaves (**Supplementary Table S.2**). Cluster H indices had a similar focus on chlorophyll estimation, but fewer of them focused squarely on the red edge. Cluster I related to photochemical reflectance indices with focus on xanthophyll cycle pigments. Finally, Cluster J focused on greenness, and many of these indices included a waveband for visible green radiation. Generally, the hierarchical clustering algorithm performed very well at grouping similar indices together, and indices developed by the same researcher with the same data were often contained in the same cluster.

**Figure 10:**
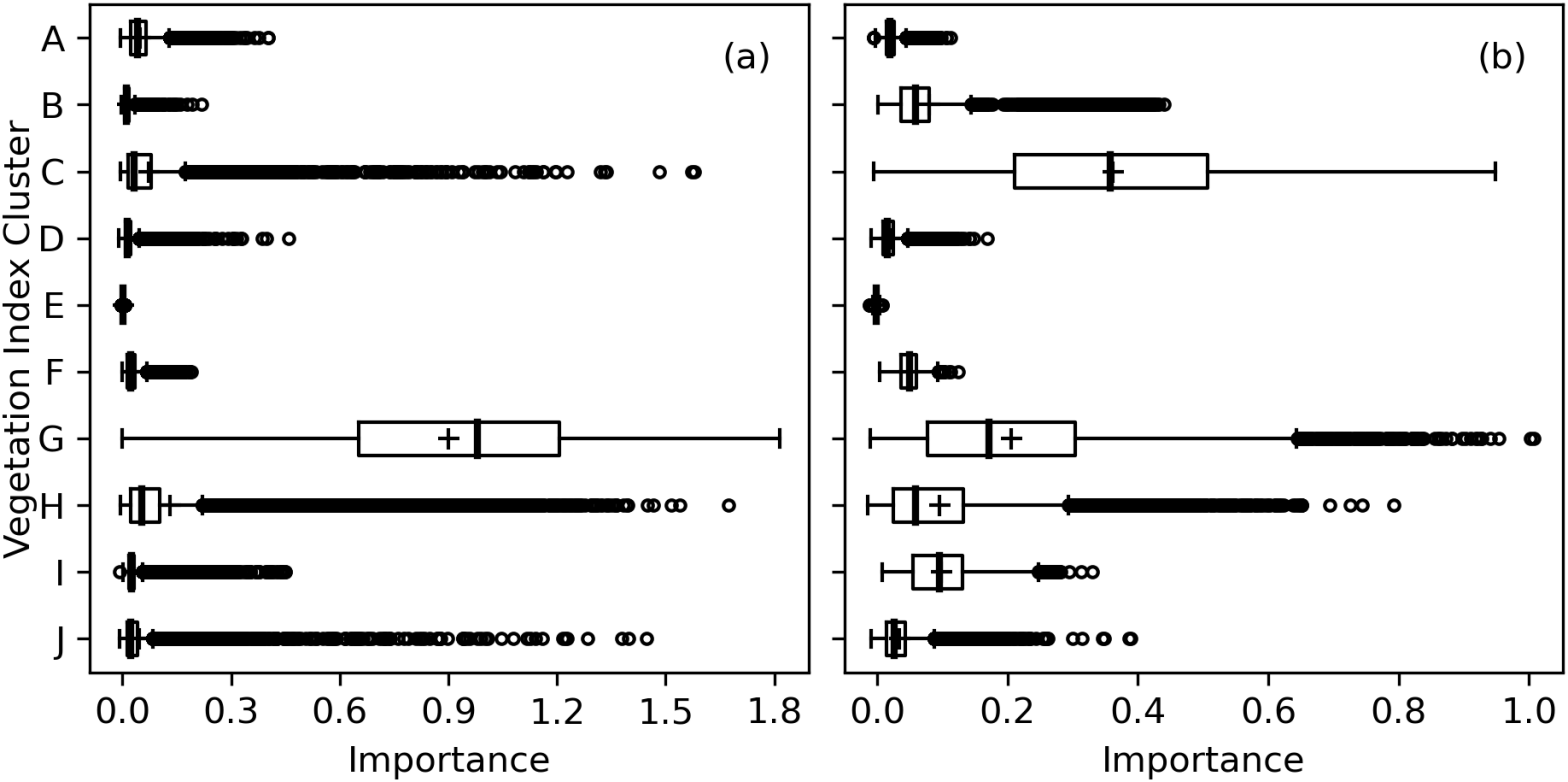
Permutation importances (computed as the reduction in model fit score when values of a feature input to random forest models were permuted) among 10 clusters of 148 spectral vegetation indices for estimation of **(A)** area-basis chlorophyll *a+b* (µg cm^-2^) and **(B)** mass-basis chlorophyll *a+b* (mg g^-1^).

As compared to RandomForestRegressor models with all 148 spectral indices inputted, the RMSE’s for RandomForestRegressor models with 10 inputted indices was typically increased by only 1-3% for Chl *a+b* and Chl *a* and up to 6% for Chl *b*. Indices within Cluster G were typically most important for estimation of area-basis Chl *a+b* (**Figure 10A**). Except for 5 of 36 indices in Cluster G, the mean importance among the 10,000 permutation trials for indices in Cluster G was greater than 0.50, which was substantially greater than the maximum whisker position for box plots of any other cluster (**Figure 10A**). Clusters C, H, and J each had outlier importances greater than 0.5; however, these importances were typically achieved when one of the five poorer indices was selected to represent Cluster G. For estimation of mass-basis Chl *a+b*, the most important indices were more distributed among Clusters C, G, H, and I (**Figure 10B**), indicating the red edge radiation response to chlorophyll may be more dictated by the chlorophyll per leaf area than per leaf mass. Interpretations of permutation importances were similar for Chl *a* (**Supplementary Figure S.5**) and Chl *b* (**Supplementary Figure S.6**). Furthermore, for each of the 148 indices, the mean importance among the 10,000 permutation trials for estimation of all six chlorophyll metrics are given in the **Supplementary Material** (**Supplementary Table S.21**). Overwhelmingly, indices from Clusters G and C were highly important for estimating cotton leaf chlorophyll, followed by selected indices from Clusters H, I, and J.

**Figure 11:**
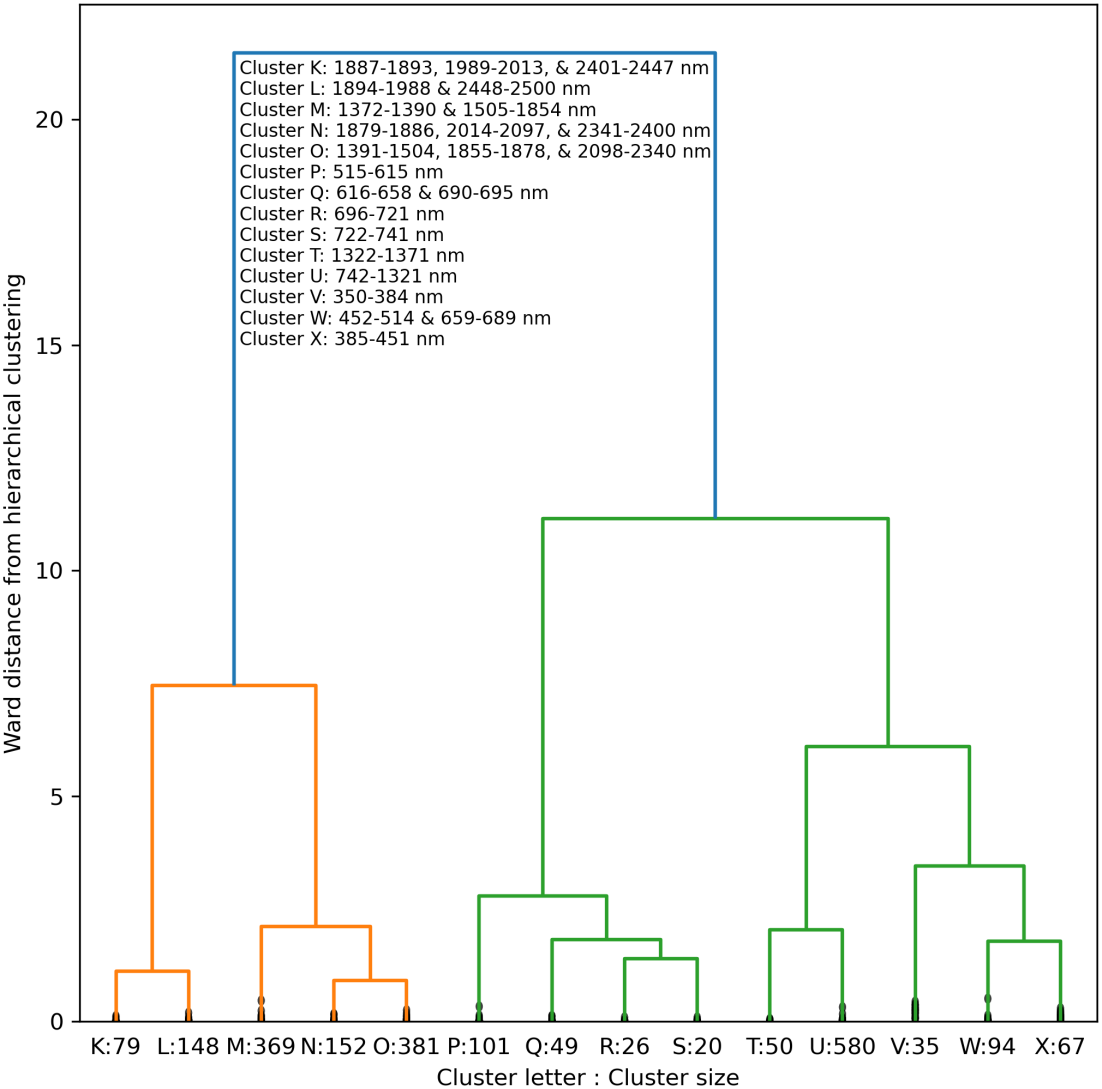
Truncated dendrogram based on hierarchical cluster analysis of cotton leaf spectral reflectance data in 2151 wavebands from 350 to 2500 nm, collected during two field experiments in the 2019–2022 growing seasons at Maricopa, Arizona, USA. To cluster the data, the dendrogram was truncated at a Ward distance of 0.865 and nodes below this value indicate points of further branching for within-cluster wavebands.

A truncated dendrogram from hierarchical clustering of cotton leaf spectral reflectance in 2151 wavebands demonstrated 14 groups of similar data (**Figure 11**). To cluster the data, the cut-off point was manually selected at a Ward distance of 0.865. The reflectance data tended to cluster together among ranges of wavelengths both within and among clusters. For example, Clusters K, L, M, N, O, and P were comprised of 2 or 3 groups of consecutive wavelengths in the short-wave infrared. Clusters P and Q represented green and red visible light, respectively. Clusters R and S contained data from the lower and upper sections of the red edge region, respectively, with a split at 721–722 nm near the red edge inflection point. Clusters T and U contained near-infrared radiation, and Cluster V represented ultraviolet radiation. Clusters W represented a combination of red and blue visible light, and Cluster X corresponded to blue light. The clustering algorithm demonstrated how hyperspectral reflectance data can be grouped into meaningful groups according to their position on the electromagnetic spectrum.

**Figure 12:**
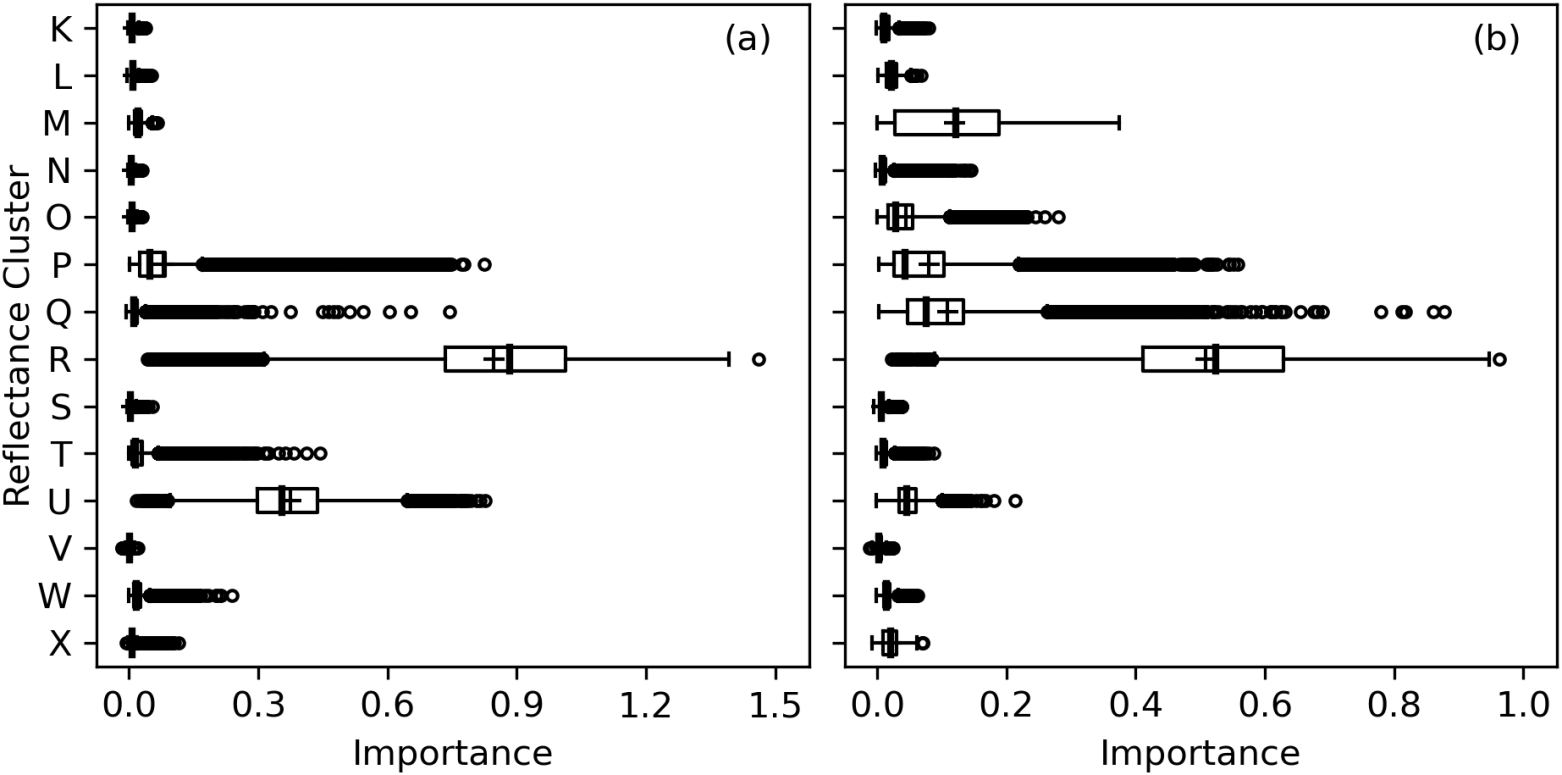
Permutation importances (computed as the reduction in model fit score when values of a feature input to random forest models were permuted) among 14 clusters of 2151 spectral reflectance wavebands at 350–2500 nm for estimation of **(A)** area-basis chlorophyll *a+b* (µg cm^-2^) and **(B)** mass-basis chlorophyll *a+b* (mg g^-1^).

The RMSE’s for RandomForestRegressor models with 14 inputted wavebands was typically very similar and within 0-2% of values with all 2151 wavebands inputted. Cluster R, which represented a narrow set of red edge wavebands at 696–721 nm, was the most important for estimation of area-basis Chl *a+b*, followed by near-infrared wavebands at 742–1321 nm in Cluster U (**Figure 12A**). For mass-basis Chl *a+b*, Cluster R was again most important, but reflectance data in Cluster M (short-wave infrared radiation), Cluster P (visible green light), and Cluster Q (visible red light) had greater importances as compared to area-basis Chl *a+b* (**Figure 12B**). Interpretations of permutation importances were similar for Chl *a* (**Supplementary Figure S.7**) and Chl *b* (**Supplementary Figure S.8**). The results confirm the importance of red edge radiation for estimation of cotton leaf chlorophyll.

## 4 Discussion

Grybowski et al. (2021) suggested a need to assess the ability of trained machine learning models to perform adequately for estimating plant traits in different years or locations. In the present study, the working hypothesis during the four years of data collection was that data from the 2019–2020 experiment could be used for machine learning model training, and those models could be tested and applied using data from the 2021–2022 experiment. However, this strategy ultimately did not provide useful models, because model performance was substantially degraded for 2021–2022 data when trained with 2019–2020 data. The finding was likely due to the unexpected differences in amounts of leaf chlorophyll measured in 2019–2020 compared to 2021–2022. Better model performance was attained when using the more common approach of randomly dividing the complete dataset from both experiments into training and testing sets. While the data from both experiments were gathered using the same methods from cotton plants grown in relatively similar field environments, the reasons for the differences in chlorophyll measurements between experiments, other than obvious differences in the evaluated cultivars, are largely unknown. However, the results suggest major limitations in the use of machine learning models across experiments as originally intended in this study and furthermore suggest that the machine learning models developed in this study were unable to perform adequately beyond the experimental conditions of the data used for their training. This means the models trained using data from the 2019–2020 experiment could likely be useful only for the experimental conditions in those two growing seasons. Likewise, the models trained and tested using an 80% and 20% random split of data from all four growing seasons could be reliably used within the conditions of those four experiments, but likely not applied with data from experiments in other growing seasons or locations. For example, the data collection protocols for the 2021–2022 experiment included collections of cotton leaf spectral reflectance data from additional plots, which were not included in the present study because corresponding leaf tissue samples for chlorophyll extractions were not collected. The plan was to use the modeling results from the present study to rapidly estimate chlorophyll from these additional plots and therefore avoid the labor required to obtain chlorophyll estimates from tissue extractions. The results suggest that the models trained and tested using an 80% and 20% random split of data from all four growing season could be used to make these chlorophyll estimations, but the reliability of the models for any estimation tasks beyond the additional plots from the 2021–2022 experiment would be questionable. As the ideal goal for model development is a model that is transferrable to the conditions of other growing seasons or locations, the results call to question the utility of machine learning models for this purpose, even for studies conducted in different years, in different fields, or with different varieties at the same research station. Improved model generalizability likely requires much more data collected in more diverse conditions than was collected during the four cotton growing seasons presented herein, and machine learning modelers must therefore ensure that their models are not applied beyond the conditions represented in their training data.

A second need expressed by Grybowski et al. (2021) was that machine learning studies should more often evaluate multiple machine learning algorithms. Herein, fourteen regression approaches were compared for estimating cotton leaf chlorophyll from spectral reflectance data. While outcomes were similar for many of the techniques, the results demonstrated significant performance boosts (*p* < 0.05) for “ensemble” methods, including GradientBoostingRegressor, RandomForestRegressor, and AdaBoostRegressor. These methods improve estimation by combining several basic estimators to improve generalizability and robustness as compared to a single estimator. Similarly, the MLPRegressor neural network approach demonstrated significant performance boosts (*p* < 0.05). Preliminary results using deep learning, specifically a one-dimensional convolutional neural network from the ‘keras’ package in Python, did not provide satisfactory results (not presented). One explanation could be the limited size of the data set, as deep learning typically requires many more samples than there are features. Herein, four years of field data collection efforts provided 1544 samples at plot scale while the hyperspectral data included 2151 reflectance measurements (i.e., features). Thus, the present study focused on non-deep learning methods and further refinement of dimensionality reduction and feature selection approaches for the hyperspectral data set. Based on the knowledge gained in the present studies, exploration of deep learning methodologies should be revisited in future research.

Based on the findings reported herein, a primary disadvantage of machine learning is that subjective decisions by the modeler can have substantial effects on modeling results. Foremost, the choice of strategy for splitting data into required training and testing data sets had major implications for modeling outcomes, and the initial plan to train and test models using data from two separate experiments ultimately failed. Using the more traditional approach of training and testing based on random splitting of the complete data set worked better, but the usability of these models with data from other site-years remains questionable. Second, modeling outcomes are affected by choices on standardization of input variables, such as whether it should be done or not and, if so, which method to use. Third, the modeler must choose a machine learning algorithm or try a set of them. Fourth, the modeler must choose how hyperparameters are specified for each algorithm, whether they are evaluated using cross validation techniques, and, if so, which values or ranges of values to evaluate, which cross validation technique to use, and the values for number of splits, repeats and other cross validation options. Altogether, the level of subjectivity required for machine learning may be too great for the practical breeder or agricultural field scientist. On the other hand, machine learning models can be good estimators when used within the constraints of their training data sets.

Biological data sets may present additional challenges for machine learning models due to the potential for uncertainty in biological processes and associated measurement errors. For example, increased canopy temperature and reduced soil water content have previously been associated with changes to leaf morphological traits, such as leaf thickness, that could convolute the estimation of leaf chlorophyll from spectral reflectance measurements (Salem-Fnayou et al., 2011; Khan et al., 2023). Furthermore, leaf spectral reflectance is also influenced by leaf water content (Schlemmer et al., 2005), protein content (Veverka et al., 2021), and pigments other than chlorophyll (Sims and Gamon, 2002). Variation in these other factors can contribute to noise in models relating spectral reflectance to chlorophyll alone. In the present study, performance of machine learning models was also limited by measurement error in the chlorophyll data used for model training. Comparisons of chlorophyll extractions from paired tissue samples from the same cotton leaves in 2019–2020 suggested that one chlorophyll extraction could estimate the other with RMSD ranging 8%–20%. Likewise, the RMSE between measured and modeled chlorophyll for machine learning model testing was also in this range. Results suggested that the improvement of chlorophyll measurement repeatability would assist improvements in model performance. Alternatively, breeders and geneticists could forego the direct estimation of chlorophyll and rely instead on spectral features or indices that demonstrate greatest correlation to chlorophyll for the species of interest. Unlike machine learning, spectral vegetation indices had consistent performance when applied to data from separate field experiments. Furthermore, simple linear regression models relating some indices to chlorophyll could perform with RMSE no more than 6% greater than chlorophyll estimation from a second tissue extraction. Spectral vegetation indices also have greater biophysiological basis than machine learning models, and new tools developed during this study (Thorp, 2024) can speed their computation. Practical breeders and field scientists may prefer to focus on development of rapid and repeatable sensing techniques for computation of spectral vegetation indices as a surrogate for direct laboratory-based estimates of chlorophyll and in lieu of chlorophyll estimation via machine learning modeling. If such indices demonstrated better measurement repeatability in addition to adequate biological heritability (Grybowski et al., 2021), they could potentially be used for both breeding selection and genome-wide association studies (Rufo et al., 2021).

Another research need expressed by Grybowski et al. (2021) was to reduce the “black box” nature of machine learning models by evaluating model input feature importance to better understand the physiological mechanisms that govern reflectance of radiation from plants. However, whereas machine learning models are often robust to multicollinearity for purposes of estimation, they are less robust to multicollinearity when used for interpretation. Herein, a clustering methodology was used to group similar indices and reflectance wavebands to reduce effects of multicollinearity in analyses of feature importance using a permutation methodology. Results clearly indicated the great importance of reflectance information at the red edge, specifically at 696–721 nm, and the importance of existing spectral vegetation indices that incorporate or analyze reflectance information at the red edge. Also, unlike the chlorophyll estimates from laboratory extractions, the means of spectral reflectance data in the red edge region, specifically at 691–693 nm and 718–723 nm, were not significantly different for the 2019–2020 and 2021–2022 experiments (*p* > 0.05), suggesting a measurement that is both highly related to chlorophyll content and repeatable across experiments. Future research should continue to develop and fine-tune spectral indices and related methodologies to utilize red edge radiation to estimate cotton leaf chlorophyll. The real value of machine learning in this study and for hyperspectral data analysis in general is likely in its data mining capabilities to identify informative spectral features or indices with greater biophysical meaning, as compared to its strict use as a trait estimation tool.

## Data Availability

The raw data supporting the conclusions of this article will be made available by the authors, without undue reservation.
