# Supplementary material for: Phenotyping cotton leaf chlorophyll via in situ hyperspectral reflectance sensing, spectral vegetation indices, and machine learning
Source: Front Plant Sci. 2024 Nov 21;15:1495593. doi: 10.3389/fpls.2024.1495593 (PMC11617151; doi:10.3389/fpls.2024.1495593)
Supplement: Supplementary file 1 [file DataSheet1.pdf]

Supplementary material for Thorp et al. (2024). "Phenotyping cotton leaf chlorophyll via *in situ* hyperspectral reflectance sensing, spectral vegetation indices, and machine learning" Submitted to Frontiers in Plant Science.

August 26, 2024

List of Tables

S.1 Chronological summary of 148 spectral vegetation indices developed from 1968 to the present time. Indices are defined using formulas based on reflectance (ρ), the first derivative of reflectance (ρ'), and the second derivative of reflectance (ρ'') with wavelengths (λ) identified either specifically in nanometers (i.e., λ_{675}) or generally as blue (λ_{BLU}), green (λ_{GRN}), red (λ_{RED}), red edge (λ_{RDE}), or near-infrared (λ_{NIR}) wavebands. 5

S.2 Additional information for each spectral vegetation index (indicated by its year and code from Table S.1), including the evaluated plant species, the scale of the remote sensing data, and the evaluated dependent variables. Information was collected only from the original publication(s) for each spectral index as cited, and use of indices for other plant species and dependent variables can likely be found with further literature review. 25

S.3 Simple linear regression statistics, including root mean squared errors (RMSE, %) and coefficients of determination (r^2), for 148 spectral vegetation indices to estimate area-basis cotton leaf chlorophyll $a + b$ (Chl $a + b$; μg cm^{-2}) for data sets collected during field studies at Maricopa, Arizona, USA. Definitions and formulas for each spectral index are given in Table S.1. 34

S.4 Simple linear regression statistics, including root mean squared errors (RMSE, %) and coefficients of determination (r^2), for 148 spectral vegetation indices to estimate area-basis cotton leaf chlorophyll a (Chl a; μg cm^{-2}) for data sets collected during field studies at Maricopa, Arizona, USA. Definitions and formulas for each spectral index are given in Table S.1. 39

S.5 Simple linear regression statistics, including root mean squared errors (RMSE, %) and coefficients of determination (r^2), for 148 spectral vegetation indices to estimate area-basis cotton leaf chlorophyll b (Chl b; μg cm^{-2}) for data sets collected during field studies at Maricopa, Arizona, USA. Definitions and formulas for each spectral index are given in Table S.1. 44

S.6 Simple linear regression statistics, including root mean squared errors (RMSE, %) and coefficients of determination (r^2), for 148 spectral vegetation indices to estimate mass-basis cotton leaf chlorophyll $a + b$ (Chl $a + b$; mg g^{-1}) for data sets collected during field studies at Maricopa, Arizona, USA. Definitions and formulas for each spectral index are given in Table S.1. 49

S.7 Simple linear regression statistics, including root mean squared errors (RMSE, %) and coefficients of determination (r^2), for 148 spectral vegetation indices to estimate mass-basis cotton leaf chlorophyll a (Chl a; mg g^{-1}) for data sets collected during field studies at Maricopa, Arizona, USA. Definitions and formulas for each spectral index are given in Table S.1. 54
S.8 Simple linear regression statistics, including root mean squared errors (RMSE, %) and coefficients of determination (r^2), for 148 spectral vegetation indices to estimate mass-basis cotton leaf chlorophyll b (Chl b; mg g^{-1}) for data sets collected during field studies at Maricopa, Arizona, USA. Definitions and formulas for each spectral index are given in Table S.1. 59
S.9 Optimized machine learning hyperparameters for estimating area-basis cotton leaf chlorophyll $a+b$ (Chl $a+b$, μg cm^{-2}) with spectral reflectance data sets from field trials at Maricopa, Arizona, USA. Twelve machine learning models from Python's "scikit-learn" package were optimized, while two other methods (BayesianRidge and GaussianProcessRegressor) were also tested but required no hyperparameter optimization. Eight spectral data sets were tested, including spectral reflectance (ρ); the first and second derivatives of reflectance (ρ' and ρ'', respectively); the base-10 logarithm of the inverse of reflectance ($\log_{10} \rho^{-1}$) and its first and second derivatives [$(\log_{10} \rho^{-1})'$ and $(\log_{10} \rho^{-1})''$, respectively]; continuum-removed reflectance (ρ_{CR}); and the set of 148 spectral indices from Table S.1. 64
S.10 Optimized machine learning hyperparameters for estimating area-basis cotton leaf chlorophyll a (Chl a, μg cm^{-2}) with spectral reflectance data sets from field trials at Maricopa, Arizona, USA. Twelve machine learning models from Python's "scikit-learn" package were optimized, while two other methods (BayesianRidge and GaussianProcessRegressor) were also tested but required no hyperparameter optimization. Eight spectral data sets were tested, including spectral reflectance (ρ); the first and second derivatives of reflectance (ρ' and ρ'', respectively); the base-10 logarithm of the inverse of reflectance ($\log_{10} \rho^{-1}$) and its first and second derivatives [$(\log_{10} \rho^{-1})'$ and $(\log_{10} \rho^{-1})''$, respectively]; continuum-removed reflectance (ρ_{CR}); and the set of 148 spectral indices from Table S.1. 65
S.11 Optimized machine learning hyperparameters for estimating area-basis cotton leaf chlorophyll b (Chl b, μg cm^{-2}) with spectral reflectance data sets from field trials at Maricopa, Arizona, USA. Twelve machine learning models from Python's "scikit-learn" package were optimized, while two other methods (BayesianRidge and GaussianProcessRegressor) were also tested but required no hyperparameter optimization. Eight spectral data sets were tested, including spectral reflectance (ρ); the first and second derivatives of reflectance (ρ' and ρ'', respectively); the base-10 logarithm of the inverse of reflectance ($\log_{10} \rho^{-1}$) and its first and second derivatives [$(\log_{10} \rho^{-1})'$ and $(\log_{10} \rho^{-1})''$, respectively]; continuum-removed reflectance (ρ_{CR}); and the set of 148 spectral indices from Table S.1. 66
S.12 Optimized machine learning hyperparameters for estimating mass-basis cotton leaf chlorophyll $a+b$ (Chl $a+b$, mg g^{-1}) with spectral reflectance data sets from field trials at Maricopa, Arizona, USA. Twelve machine learning models from Python's "scikit-learn" package were optimized, while two other methods (BayesianRidge and GaussianProcessRegressor) were also tested but required no hyperparameter optimization. Eight spectral data sets were tested, including spectral reflectance (ρ); the first and second derivatives of reflectance (ρ' and ρ'', respectively); the base-10 logarithm of the inverse of reflectance ($\log_{10} \rho^{-1}$) and its first and second derivatives [$(\log_{10} \rho^{-1})'$ and $(\log_{10} \rho^{-1})''$, respectively]; continuum-removed reflectance (ρ_{CR}); and the set of 148 spectral indices from Table S.1. 67
S.13 Optimized machine learning hyperparameters for estimating mass-basis cotton leaf chlorophyll a (Chl a, mg g^{-1}) with spectral reflectance data sets from field trials at Maricopa, Arizona, USA. Twelve machine learning models from Python's "scikit-learn" package were optimized, while two other methods (BayesianRidge and GaussianProcessRegressor) were also tested but required no hyperparameter optimization. Eight spectral data sets were tested, including spectral reflectance (ρ); the first and second derivatives of reflectance (ρ' and ρ'', respectively); the base-10 logarithm of the inverse of reflectance ($\log_{10} \rho^{-1}$) and its first and second derivatives [$(\log_{10} \rho^{-1})'$ and $(\log_{10} \rho^{-1})''$, respectively]; continuum-removed reflectance (ρ_{CR}); and the set of 148 spectral indices from Table S.1. 68

S.14 Optimized machine learning hyperparameters for estimating mass-basis cotton leaf chlorophyll b (Chl b, mg g^{-1}) with spectral reflectance data sets from field trials at Maricopa, Arizona, USA. Twelve machine learning models from Python's "scikit-learn" package were optimized, while two other methods (BayesianRidge and GaussianProcessRegressor) were also tested but required no hyperparameter optimization. Eight spectral data sets were tested, including spectral reflectance (ρ); the first and second derivatives of reflectance (ρ' and ρ'', respectively); the base-10 logarithm of the inverse of reflectance ($\log_{10}\rho^{-1}$) and its first and second derivatives [$(\log_{10}\rho^{-1})'$ and $(\log_{10}\rho^{-1})''$, respectively]; continuum-removed reflectance (ρ_{CR}); and the set of 148 spectral indices from Table S.1. 69
S.15 Goodness-of-fit statistics, including root mean squared errors (%RMSE) and coefficients of determination (r^2) between measured and modeled area-basis cotton leaf chlorophyll $a+b$ (Chl $a+b$, μg cm^{-2}) for training and testing of 14 machine learning methods from Python's "scikit-learn" package with 8 input data sets derived from leaf spectral reflectance (ρ). The 8 input data sets included spectral reflectance (ρ); the first and second derivatives of reflectance (ρ' and ρ'', respectively); the base-10 logarithm of the inverse of reflectance ($\log_{10}\rho^{-1}$) and its first and second derivatives [$(\log_{10}\rho^{-1})'$ and $(\log_{10}\rho^{-1})''$, respectively]; continuum-removed reflectance (ρ_{CR}); and the set of 148 spectral indices from Table S.1. Models were trained and tested by experiment using data from the 2019–2020 and 2021–2022 cotton field studies at Maricopa, Arizona, USA, respectively. Models were also trained and tested using an 80% and 20% random split of all from both experiments. Results are ranked according to the %RMSE of model testing. 70
S.16 Goodness-of-fit statistics, including root mean squared errors (%RMSE) and coefficients of determination (r^2) between measured and modeled area-basis cotton leaf chlorophyll a (Chl a, μg cm^{-2}) for training and testing of 14 machine learning methods from Python's "scikit-learn" package with 8 input data sets derived from leaf spectral reflectance (ρ). The 8 input data sets included spectral reflectance (ρ); the first and second derivatives of reflectance (ρ' and ρ'', respectively); the base-10 logarithm of the inverse of reflectance ($\log_{10}\rho^{-1}$) and its first and second derivatives [$(\log_{10}\rho^{-1})'$ and $(\log_{10}\rho^{-1})''$, respectively]; continuum-removed reflectance (ρ_{CR}); and the set of 148 spectral indices from Table S.1. Models were trained and tested by experiment using data from the 2019–2020 and 2021–2022 cotton field studies at Maricopa, Arizona, USA, respectively. Models were also trained and tested using an 80% and 20% random split of all from both experiments. Results are ranked according to the %RMSE of model testing. 74
S.17 Goodness-of-fit statistics, including root mean squared errors (%RMSE) and coefficients of determination (r^2) between measured and modeled area-basis cotton leaf chlorophyll b (Chl b, μg cm^{-2}) for training and testing of 14 machine learning methods from Python's "scikit-learn" package with 8 input data sets derived from leaf spectral reflectance (ρ). The 8 input data sets included spectral reflectance (ρ); the first and second derivatives of reflectance (ρ' and ρ'', respectively); the base-10 logarithm of the inverse of reflectance ($\log_{10}\rho^{-1}$) and its first and second derivatives [$(\log_{10}\rho^{-1})'$ and $(\log_{10}\rho^{-1})''$, respectively]; continuum-removed reflectance (ρ_{CR}); and the set of 148 spectral indices from Table S.1. Models were trained and tested by experiment using data from the 2019–2020 and 2021–2022 cotton field studies at Maricopa, Arizona, USA, respectively. Models were also trained and tested using an 80% and 20% random split of all from both experiments. Results are ranked according to the %RMSE of model testing. 78
S.18 Goodness-of-fit statistics, including root mean squared errors (%RMSE) and coefficients of determination (r^2) between measured and modeled mass-basis cotton leaf chlorophyll $a+b$ (Chl $a+b$, mg g^{-1}) for training and testing of 14 machine learning methods from Python's "scikit-learn" package with 8 input data sets derived from leaf spectral reflectance (ρ). The 8 input data sets included spectral reflectance (ρ); the first and second derivatives of reflectance (ρ' and ρ'', respectively); the base-10 logarithm of the inverse of reflectance ($\log_{10}\rho^{-1}$) and its first and second derivatives [$(\log_{10}\rho^{-1})'$ and $(\log_{10}\rho^{-1})''$, respectively]; continuum-removed reflectance (ρ_{CR}); and the set of 148 spectral indices from Table S.1. Models were trained and tested by experiment using data from the 2019–2020 and 2021–2022 cotton field studies at Maricopa, Arizona, USA, respectively. Models were also trained and tested using an 80% and 20% random split of all from both experiments. Results are ranked according to the %RMSE of model testing. 82

S.19 Goodness-of-fit statistics, including root mean squared errors (%RMSE) and coefficients of determination (r^2) between measured and modeled mass-basis cotton leaf chlorophyll *a* (Chl *a*, mg g^{-1}) for training and testing of 14 machine learning methods from Python's "scikit-learn" package with 8 input data sets derived from leaf spectral reflectance (ρ). The 8 input data sets included spectral reflectance (ρ); the first and second derivatives of reflectance (ρ' and ρ'', respectively); the base-10 logarithm of the inverse of reflectance ($\log_{10} \rho^{-1}$) and its first and second derivatives [$(\log_{10} \rho^{-1})'$ and $(\log_{10} \rho^{-1})''$, respectively]; continuum-removed reflectance (ρ_{CR}); and the set of 148 spectral indices from Table S.1. Models were trained and tested by experiment using data from the 2019–2020 and 2021–2022 cotton field studies at Maricopa, Arizona, USA, respectively. Models were also trained and tested using an 80% and 20% random split of all from both experiments. Results are ranked according to the %RMSE of model testing. 86
S.20 Goodness-of-fit statistics, including root mean squared errors (%RMSE) and coefficients of determination (r^2) between measured and modeled mass-basis cotton leaf chlorophyll *b* (Chl *b*, mg g^{-1}) for training and testing of 14 machine learning methods from Python's "scikit-learn" package with 8 input data sets derived from leaf spectral reflectance (ρ). The 8 input data sets included spectral reflectance (ρ); the first and second derivatives of reflectance (ρ' and ρ'', respectively); the base-10 logarithm of the inverse of reflectance ($\log_{10} \rho^{-1}$) and its first and second derivatives [$(\log_{10} \rho^{-1})'$ and $(\log_{10} \rho^{-1})''$, respectively]; continuum-removed reflectance (ρ_{CR}); and the set of 148 spectral indices from Table S.1. Models were trained and tested by experiment using data from the 2019–2020 and 2021–2022 cotton field studies at Maricopa, Arizona, USA, respectively. Models were also trained and tested using an 80% and 20% random split of all from both experiments. Results are ranked according to the %RMSE of model testing. 90
S.21 Mean permutation importances for using each of 148 spectral vegetation indices to estimate area-basis and mass-basis chlorophyll $a + b$ (Chl $a + b$), chlorophyll *a* (Chl *a*), and chlorophyll *b* (Chl *b*) with random forest machine learning models. To avoid effects of multicollinearity, indices were grouped into 10 feature sets using hierarchical clustering, and random forest models were fit with one spectral index randomly chosen from each feature set. Iterating over 10,000 unique model fits for each chlorophyll metric ensured each spectral index was evaluated multiple times. Permutation importances were computed as the reduction in model fit score when values of a feature input to random forest models were permuted. The importances of spectral indices are ranked from greatest to least. . . . 94

List of Figures

S.1 Comparison of cotton leaf chlorophyll *a* (Chl *a*) extractions among paired tissue samples from the same cotton leaf (n=2,916) in units of a) μg cm^{-2} for area-basis estimates and b) mg g^{-1} for mass-basis estimates. Samples were collected during a 2019-2020 field study at Maricopa, Arizona. 98
S.2 Comparison of cotton leaf chlorophyll *b* (Chl *b*) extractions among paired tissue samples from the same cotton leaf (n=2,916) in units of a) μg cm^{-2} for area-basis estimates and b) mg g^{-1} for mass-basis estimates. Samples were collected during a 2019-2020 field study at Maricopa, Arizona. 99
S.3 Goodness-of-fit statistics for partial least squares regression (PLSR) models that were fit using cotton leaf chlorophyll *a* (Chl *a*) and spectral reflectance data at four different scales (i.e., sample, leaf, plot, and entry) and using two methods to split the data for training and testing phases (i.e., by experiment and by using an 80% and 20% random split of combined data from both experiments). Results are shown as a) root mean squared errors (RMSE) and b) coefficients of determination (r^2) for area-basis Chl *a* (μg cm^{-2}) and c) RMSE and d) r^2 for mass-basis Chl *a* (mg g^{-1}). 100

S.4 Goodness-of-fit statistics for partial least squares regression (PLSR) models that were fit using cotton leaf chlorophyll *b* (Chl *b*) and spectral reflectance data at four different scales (i.e., sample, leaf, plot, and entry) and using two methods to split the data for training and testing phases (i.e., by experiment and by using an 80% and 20% random split of combined data from both experiments). Results are shown as a) root mean squared errors (RMSE) and b) coefficients of determination (r^2) for area-basis Chl *b* (μg cm^{-2}) and c) RMSE and d) r^2 for mass-basis Chl *b* (mg g^{-1}). 101
S.5 Permutation importances (computed as the reduction in model fit score when values of a feature input to random forest models were permuted) among 10 clusters of 148 spectral vegetation indices for estimation of a) area-basis chlorophyll *a* (μg cm^{-2}) and b) mass-basis chlorophyll *a* (mg g^{-1}). 102
S.6 Permutation importances (computed as the reduction in model fit score when values of a feature input to random forest models were permuted) among 10 clusters of 148 spectral vegetation indices for estimation of a) area-basis chlorophyll *b* (μg cm^{-2}) and b) mass-basis chlorophyll *b* (mg g^{-1}). 103
S.7 Permutation importances (computed as the reduction in model fit score when values of a feature input to random forest models were permuted) among 14 clusters of 2151 spectral reflectance wavebands at 350-2500 nm for estimation of a) area-basis chlorophyll *a* (μg cm^{-2}) and b) mass-basis chlorophyll *a* (mg g^{-1}). 104
S.8 Permutation importances (computed as the reduction in model fit score when values of a feature input to random forest models were permuted) among 14 clusters of 2151 spectral reflectance wavebands at 350-2500 nm for estimation of a) area-basis chlorophyll *b* (μg cm^{-2}) and b) mass-basis chlorophyll *b* (mg g^{-1}). 105

Table S.1: Chronological summary of 148 spectral vegetation indices developed from 1968 to the present time. Indices are defined using formulas based on reflectance (ρ), the first derivative of reflectance (ρ'), and the second derivative of reflectance (ρ'') with wavelengths (λ) identified either specifically in nanometers (i.e., λ_{675}) or generally as blue (λ_{BLU}), green (λ_{GRN}), red (λ_{RED}), red edge (λ_{RDE}), or near-infrared (λ_{NIR}) wavebands.

Year	Code	Description	Type[1]	Formula	Citation
1968	BRSR	Birth simple ratio	SR	$\frac{\rho_{745}}{\rho_{675}}$	Birth and McVey (1968)
1969	JSR	Jordan simple ratio	SR	$\frac{\rho_{800}}{\rho_{675}}$	Jordan (1969)

Continued on next page

Year	Code	Description	Type[1]	Formula	Citation
1973	NDVI	Normalized Difference Vegetation Index (NDVI)	ND	$\frac{\rho_{\text{NIR}} - \rho_{\text{RED}}}{\rho_{\text{NIR}} + \rho_{\text{RED}}}$	Rouse et al. (1973)
1977	PVI	Perpendicular Vegetation Index (PVI)	SA	$\frac{\rho_{\text{NIR}} - a\rho_{\text{RED}} - b}{\sqrt{1+a^2}} : (a = 1.166, b = 0.024)$	Richardson and Wiegand (1977); Jackson et al. (1980); Huete et al. (1984)
1978	WLREIP	Wavelength of red edge inflection point	SF	$\lambda_{\text{RDE}} = \arg\max_{\lambda}(\rho'(\lambda) : 680 \leq \lambda \leq 750)$	Collins (1978); Horler et al. (1983)
1979	DVI	Difference Vegetation Index (DVI)	DF	$\rho_{\text{NIR}} - \rho_{\text{RED}}$	Tucker (1979)
1979	NDVI2	Normalized Difference Vegetation Index 2 (NDVI2)	ND	$\frac{\rho_{\text{GRN}} - \rho_{\text{RED}}}{\rho_{\text{GRN}} + \rho_{\text{RED}}}$	Tucker (1979)
1988	WLREIP2	Wavelength of red edge inflection point 2	SF	$700 + 40\left(\frac{(\rho_{670} + \rho_{780})/2 - \rho_{700}}{\rho_{740} - \rho_{700}}\right)$	Guyot and Baret (1988); Cho and Skidmore (2006)
1988	SAVI	Soil-Adjusted Vegetation Index (SAVI)	SA	$(1+L)\left(\frac{\rho_{\text{NIR}} - \rho_{\text{RED}}}{\rho_{\text{NIR}} + \rho_{\text{RED}} + L}\right) : (L = 0.5)$	Huete (1988)

Continued on next page

Year	Code	Description	Type[1]	Formula	Citation
1989	TSAVI	Transformed Soil-Adjusted Vegetation Index (TSAVI)	SA	$\frac{a(\rho_{\mathrm{NIR}} - a\rho_{\mathrm{RED}} - b)}{\rho_{\mathrm{RED}} + a\rho_{\mathrm{NIR}} - ab} : (a = 1.166, b = 0.024)$	Baret et al. (1989); Huete et al. (1984)
1989	WDVI	Weighted Difference Vegetation Index (WDVI)	SA	$\rho_{\mathrm{NIR}} - C\rho_{\mathrm{RED}} : (C = 1.166)$	Clevers (1989); Huete et al. (1984)
1989	MSI	Moisture Stress Index	SR	$\frac{\rho_{1600}}{\rho_{820}}$	Hunt and Rock (1989)
1990	BD	Boochs derivative	SF	ρ'_{703}	Boochs et al. (1990)
1990	BDR	Boochs derivative ratio	SF	$\frac{\rho'_{703}}{\max(\rho'(\lambda) : 680 \leq \lambda \leq 750)}$	Boochs et al. (1990)
1990	SAVI2	Soil-Adjusted Vegetation Index 2 (SAVI2)	SA	$\frac{\rho_{\mathrm{NIR}}}{\rho_{\mathrm{RED}} + b/a} : (a = 1.166, b = 0.024)$	Major et al. (1990); Huete et al. (1984)
1990	WLREIPG	Wavelength of red edge inflection point, Gaussian fit	SF	$\lambda_{\mathrm{RDE}} = \lambda_0 + \sigma : \rho(\lambda) = \rho_{\mathrm{s}} - (\rho_{\mathrm{s}} - \rho_0)\exp\left(\frac{-(\lambda_0 - \lambda)^2}{2\sigma^2}\right)$	Miller et al. (1990)
1990	WLCWMRG	Wavelength of chlorophyll-well minimum reflectance, Gaussian fit	SF	$\lambda_0 : \rho(\lambda) = \rho_{\mathrm{s}} - (\rho_{\mathrm{s}} - \rho_0)\exp\left(\frac{-(\lambda_0 - \lambda)^2}{2\sigma^2}\right)$	Miller et al. (1990)

Continued on next page

Table S.1 – Continued from previous page

Year	Code	Description	Type[1]	Formula	Citation
1991	TSAVI2	Transformed Soil-Adjusted Vegetation Index 2 (TSAVI2)	SA	$\frac{a(\rho_{\mathrm{NIR}} - a\rho_{\mathrm{RED}} - b)}{a\rho_{\mathrm{NIR}} + \rho_{\mathrm{RED}} - ab + X(1 + a^2)} : (X = 0.08, a = 1.166, b = 0.024)$	Baret and Guyot (1991); Huete et al. (1984)
1992	CPSR1	Chappelle simple ratio 1	SR	$\frac{\rho_{675}}{\rho_{700}}$	Chappelle et al. (1992)
1992	CPSR2	Chappelle simple ratio 2	SR	$\frac{\rho_{675}}{\rho_{650}\rho_{700}}$	Chappelle et al. (1992)
1992	CPSR3	Chappelle simple ratio 3	SR	$\frac{\rho_{760}}{\rho_{500}}$	Chappelle et al. (1992)
1992	PRI	Photochemical Reflectance Index (PRI)	ND	$\frac{\rho_{550} - \rho_{531}}{\rho_{550} + \rho_{531}}$	Gamon et al. (1992)
1992	GEMI	Global Environment Monitoring Index (GEMI)	EN	$\eta(1 - 0.25\eta) - \frac{\rho_{\mathrm{RED}} - 0.125}{1 - \rho_{\mathrm{RED}}} : (\eta = \frac{2(\rho_{\mathrm{NIR}}^2 - \rho_{\mathrm{RED}}^2) + 1.5\rho_{\mathrm{NIR}} + 0.5\rho_{\mathrm{RED}}}{\rho_{\mathrm{NIR}} + \rho_{\mathrm{RED}} + 0.5}$	Pinty and Verstraete (1992)
1993	BMSR	Buschmann simple ratio	SR	$\frac{\rho_{550}}{\rho_{800}}$	Buschmann and Nagel (1993)
1993	BMLSR	Buschmann log simple ratio	SR	$\log_{10}\left(\frac{\rho_{800}}{\rho_{550}}\right)$	Buschmann and Nagel (1993)

Continued on next page

Year	Code	Description	Type[1]	Formula	Citation
1993	BMDVI	Bushmann difference vegetation index	DF	$\rho_{800} - \rho_{550}$	Buschmann and Nagel (1993)
1993	PSR	Peñuelas simple ratio	SR	$\frac{\rho_{970}}{\rho_{900}}$	Peñuelas et al. (1993)
1993	PD	Peñuelas derivative	SF	$\min(\rho'(\lambda) : 900 \leq \lambda \leq 970)$	Peñuelas et al. (1993)
1993	WLPD	Wavelength of PD	SF	$\arg\min_{\lambda}(\rho'(\lambda) : 900 \leq \lambda \leq 970)$	Peñuelas et al. (1993)
1993	VSR	Vogelmann simple ratio	SR	$\frac{\rho_{740}}{\rho_{720}}$	Vogelmann et al. (1993)
1993	VDR	Vogelmann derivative ratio	SF	$\frac{\rho'_{715}}{\rho'_{705}}$	Vogelmann et al. (1993)
1994	CRSR1	Carter simple ratio 1	SR	$\frac{\rho_{695}}{\rho_{420}}$	Carter (1994)
1994	CRSR2	Carter simple ratio 2	SR	$\frac{\rho_{605}}{\rho_{760}}$	Carter (1994)
1994	CRSR3	Carter simple ratio 3	SR	$\frac{\rho_{695}}{\rho_{760}}$	Carter (1994)
1994	CRSR4	Carter simple ratio 4	SR	$\frac{\rho_{710}}{\rho_{760}}$	Carter (1994)

Continued on next page

Table S.1 – Continued from previous page

Year	Code	Description	Type[1]	Formula	Citation
1994	CRSR5	Carter simple ratio 5	SR	$\frac{\rho_{695}}{\rho_{670}}$	Carter (1994)
1994	FSUM	Area of the first derivative red edge peak from 680 nm to 780 nm	SF	$\sum_{\lambda=680}^{780} \rho'(\lambda) d\lambda$	Filella and Peñuelas (1994); Filella et al. (1995)
1994	DREIP	Amplitude of the first derivative at red edge inflection point	SF	$\max(\rho'(\lambda) : 680 \leq \lambda \leq 780)$	Filella and Peñuelas (1994); Filella et al. (1995)
1994	NDVI3	Normalized Difference Vegetation Index 3 (NDVI3)	ND	$\frac{\rho_{750} - \rho_{705}}{\rho_{750} + \rho_{705}}$	Gitelson and Merzlyak (1994)
1994	GSUM1	Sum of reflectance from 705 nm to 750 nm, normalized by reflectance at 705 nm	SF	$\sum_{\lambda=705}^{750} \left(\frac{\rho(\lambda)}{\rho_{705}} - 1 \right) d\lambda$	Gitelson and Merzlyak (1994)
1994	GSUM2	Sum of reflectance from 705 nm to 750 nm, normalized by reflectance at 555 nm	SF	$\sum_{\lambda=705}^{750} \left(\frac{\rho(\lambda)}{\rho_{555}} - 1 \right) d\lambda$	Gitelson and Merzlyak (1994)
1994	NLI	Nonlinear Index (NLI)	ND	$\frac{\rho_{\mathrm{NIR}}^2 - \rho_{\mathrm{RED}}}{\rho_{\mathrm{NIR}}^2 + \rho_{\mathrm{RED}}}$	Goel and Qin (1994)

Continued on next page

Table S.1 – Continued from previous page

Year	Code	Description	Type[1]	Formula	Citation
1994	CAR	Chlorophyll Absorption in Reflectance (CAR)	SF	$\sqrt{\frac{(b^T b)(a^T a) - (a^T b)^2}{(a^T a)}} : \left(a = \begin{bmatrix} \lambda_{700} - \lambda_{550} \\ \rho_{700} - \rho_{550} \end{bmatrix}, b = \begin{bmatrix} \lambda_{670} - \lambda_{550} \\ \rho_{670} - \rho_{550} \end{bmatrix} \right)$	Kim et al. (1994)
1994	CARI	Chlorophyll Absorption Ratio Index (CARI)	SF	$\text{CAR} \times \left(\frac{\rho_{700}}{\rho_{670}} \right)$	Kim et al. (1994)
1994	NPCI	Normalized Pigments Chlorophyll ratio Index (NPCI)	ND	$\frac{\rho_{680} - \rho_{430}}{\rho_{680} + \rho_{430}}$	Peñuelas et al. (1994)
1994	EGFN	Edge-Green First-derivative Normalised difference index (EGFN)	SF	$\frac{\max(\rho'(\lambda_{\text{RDE}})) - \max(\rho'(\lambda_{\text{GRN}}))}{\max(\rho'(\lambda_{\text{RDE}})) + \max(\rho'(\lambda_{\text{GRN}}))} : (500 \leq \lambda_{\text{GRN}} \leq 600, 680 \leq \lambda_{\text{RDE}} \leq 750)$	Peñuelas et al. (1994)
1994	MSAVI1	Modified Soil-Adjusted Vegetation Index 1 (MSAVI1)	SA	$(1 + L) \left(\frac{\rho_{\text{NIR}} - \rho_{\text{RED}}}{\rho_{\text{NIR}} + \rho_{\text{RED}} + L} \right) : (L = 1 - 2a \times \text{NDVI} \times \text{WDVI}, a = 1.166)$	Qi et al. (1994); Huete et al. (1984)
1994	MSAVI2	Modified Soil-Adjusted Vegetation Index 2 (MSAVI2)	SA	$\frac{2\rho_{\text{NIR}} + 1 - \sqrt{(2\rho_{\text{NIR}} + 1)^2 - 8(\rho_{\text{NIR}} - \rho_{\text{RED}})}}{2}$	Qi et al. (1994)

Continued on next page

Table S.1 – Continued from previous page

Year	Code	Description	Type[1]	Formula	Citation
1995	ESUM1	Area of the first derivative red edge peak from 626 nm to 795 nm	SF	$\sum_{\lambda=626}^{795} \lvert\rho'(\lambda)\rvert d\lambda$	Elvidge and Chen (1995)
1995	ESUM2	Area of the second derivative red edge peaks from 626 nm to 795 nm	SF	$\sum_{\lambda=626}^{795} \lvert\rho''(\lambda)\rvert d\lambda$	Elvidge and Chen (1995)
1995	NDPI	Normalized Difference Pigment Index (NDPI)	ND	$\frac{\rho_{670} - \rho_{420}}{\rho_{670} + \rho_{420}}$	Peñuelas et al. (1995a)
1995	SIPI	Structure Independent Pigment Index (SIPI)	ND	$\frac{\rho_{800} - \rho_{445}}{\rho_{800} - \rho_{680}}$	Peñuelas et al. (1995a)
1995	SRPI	Simple Ratio Pigment Index (SRPI)	SR	$\frac{\rho_{430}}{\rho_{680}}$	Peñuelas et al. (1995b)
1995	NPQI	Normalized Phaeophytinization Index (NPQI)	ND	$\frac{\rho_{415} - \rho_{435}}{\rho_{415} + \rho_{435}}$	Peñuelas et al. (1995b)
1995	RDVI	Renormalized Difference Vegetation Index (RDVI)	ND	$\frac{\rho_{\mathrm{NIR}} - \rho_{\mathrm{RED}}}{\sqrt{\rho_{\mathrm{NIR}} + \rho_{\mathrm{RED}}}}$	Roujean and Breon (1995)
1996	MSR	Modified Simple Ratio (MSR)	EN	$\frac{\rho_{\mathrm{NIR}}/\rho_{\mathrm{RED}} - 1}{\sqrt{\rho_{\mathrm{NIR}}/\rho_{\mathrm{RED}} + 1}}$	Chen (1996); Roujean and Breon (1995)

Continued on next page

Table S.1 – Continued from previous page

Year	Code	Description	Type[1]	Formula	Citation
1996	PRI2	Photochemical Reflectance Index 2 (PRI2)	ND	$\frac{\rho_{539} - \rho_{570}}{\rho_{539} + \rho_{570}}$	Filella et al. (1996)
1996	NDWI	Normalized Difference Water Index (NDWI)	ND	$\frac{\rho_{860} - \rho_{1240}}{\rho_{860} + \rho_{1240}}$	Gao (1996)
1996	GTSR1	Gitelson simple ratio 1	SR	$\frac{\rho_{750}}{\rho_{550}}$	Gitelson and Merzlyak (1996, 1997); Lichtenthaler et al. (1996)
1996	GTSR2	Gitelson simple ratio 2	SR	$\frac{\rho_{750}}{\rho_{700}}$	Gitelson and Merzlyak (1996, 1997); Lichtenthaler et al. (1996)
1996	GNDVI	Green Normalized Difference Vegetation Index (GNDVI)	ND	$\frac{\rho_{\mathrm{NIR}} - \rho_{\mathrm{GRN}}}{\rho_{\mathrm{NIR}} + \rho_{\mathrm{GRN}}}$	Gitelson et al. (1996)
1996	OSAVI	Optimized Soil-Adjusted Vegetation Index (OSAVI)	SA	$(1 + L)\left(\frac{\rho_{\mathrm{NIR}} - \rho_{\mathrm{RED}}}{\rho_{\mathrm{NIR}} + \rho_{\mathrm{RED}} + L}\right) : (L = 0.16)$	Rondeaux et al. (1996)
1997	WI	Water Index (WI)	SR	$\frac{\rho_{900}}{\rho_{970}}$	Peñuelas et al. (1997)

Continued on next page

Table S.1 – Continued from previous page

Year	Code	Description	Type[1]	Formula	Citation
1997	WNR	WI NDVI ratio	EN	$\frac{\mathrm{WI}}{\mathrm{NDVI}}$	Peñuelas et al. (1997)
1998	PSSRA	Pigment Specific Simple Ratio for chlorophyll *a* (PSSRa)	SR	$\frac{\rho_{800}}{\rho_{680}}$	Blackburn (1998a,b)
1998	PSSRB	Pigment Specific Simple Ratio for chlorophyll *b* (PSSRb)	SR	$\frac{\rho_{800}}{\rho_{635}}$	Blackburn (1998a,b)
1998	PSSRC	Pigment Specific Simple Ratio for carotenoid (PSSRc)	SR	$\frac{\rho_{800}}{\rho_{470}}$	Blackburn (1998a,b)
1998	PSNDA	Pigment Specific Normalized Difference for chlorophyll *a* (PSNDa)	ND	$\frac{\rho_{800}-\rho_{680}}{\rho_{800}+\rho_{680}}$	Blackburn (1998a,b)
1998	PSNDB	Pigment Specific Normalized Difference for chlorophyll *b* (PSNDb)	ND	$\frac{\rho_{800}-\rho_{635}}{\rho_{800}+\rho_{635}}$	Blackburn (1998a,b)
1998	PSNDC	Pigment Specific Normalized Difference for carotenoid (PSNDc)	ND	$\frac{\rho_{800}-\rho_{470}}{\rho_{800}+\rho_{470}}$	Blackburn (1998a,b)
1998	DSR1	Datt simple ratio 1	SR	$\frac{\rho_{672}}{\rho_{550}\rho_{708}}$	Datt (1998)

Continued on next page

Table S.1 – Continued from previous page

Year	Code	Description	Type[1]	Formula	Citation
1998	DSR2	Datt simple ratio 2	SR	$\frac{\rho_{672}}{\rho_{550}}$	Datt (1998)
1999	DNDR	Datt normalized difference ratio	ND	$\frac{\rho_{850}-\rho_{710}}{\rho_{850}-\rho_{680}}$	Datt (1999a,b)
1999	DDR1	Datt first derivative ratio	SF	$\frac{\rho'_{754}}{\rho'_{704}}$	Datt (1999b)
1999	DDR2	Datt second derivative ratio	SF	$\frac{\rho''_{712}}{\rho''_{688}}$	Datt (1999b)
1999	GMSR	Gamon simple ratio	SR	$\frac{\rho_{\mathrm{RED}}}{\rho_{\mathrm{GRN}}}$	Gamon and Surfus (1999)
1999	PSRI	Plant Senescence Reflectance Index (PSRI)	ND	$\frac{\rho_{678}-\rho_{500}}{\rho_{750}}$	Merzlyak et al. (1999)
2000	TVI	Triangular Vegetation Index (TVI)	EN	$0.5[120(\rho_{750}-\rho_{550})-200(\rho_{670}-\rho_{550})]$	Broge and Leblanc (2000)
2000	MCARI	Modified Chlorophyll Absorption in Reflectance Index (MCARI)	EN	$[(\rho_{700}-\rho_{670})-0.2(\rho_{700}-\rho_{550})]\left(\frac{\rho_{700}}{\rho_{670}}\right)$	Daughtry et al. (2000)
2000	MOR	MCARI OSAVI ratio	EN	$\frac{\mathrm{MCARI}}{\mathrm{OSAVI}}$	Daughtry et al. (2000)

Continued on next page

Table S.1 – Continued from previous page

Year	Code	Description	Type[1]	Formula	Citation
2000	ZTSR1	Zarco-Tejada simple ratio 1	SR	$\frac{\rho_{685}}{\rho_{655}}$	Zarco-Tejada et al. (2000a,b)
2000	CI	Curvature Index (CI)	EN	$\frac{\rho_{683}^2}{\rho_{675}\rho_{691}}$	Zarco-Tejada et al. (2000a,b)
2000	ZTDR1	Zarco-Tejada derivative ratio 1	SF	$\frac{\rho'_{730}}{\rho'_{706}}$	Zarco-Tejada et al. (2000b)
2000	ZTSR2	Zarco-Tejada simple ratio 2	SR	$\frac{\rho_{750}}{\rho_{710}}$	Zarco-Tejada et al. (2000b)
2001	CAI	Cellulose Absorption Index (CAI)	EN	$0.5(\rho_{2019} + \rho_{2206}) - \rho_{2109}$	Daughtry (2001)
2001	ARI	Anthocyanin Reflectance Index (ARI)	EN	$(\rho_{550})^{-1} - (\rho_{700})^{-1}$	Gitelson et al. (2001)
2001	MND1	Maccioni normalized difference 1	ND	$\frac{\rho_{780} - \rho_{710}}{\rho_{780} - \rho_{680}}$	Maccioni et al. (2001); Datt (1999b)
2001	MND2	Maccioni normalized difference 2	ND	$\frac{\rho_{542} - \min(\rho(\lambda_{\mathrm{RED}}))}{\rho_{750} - \min(\rho(\lambda_{\mathrm{RED}}))} : 660 \leq \lambda_{\mathrm{RED}} \leq 680$	Maccioni et al. (2001)

Continued on next page

Year	Code	Description	Type[1]	Formula	Citation
2001	MND3	Maccioni normalized difference 3	ND	$\frac{\rho_{706} - \min(\rho(\lambda_{\text{RED}}))}{\rho_{750} - \min(\rho(\lambda_{\text{RED}}))} : 660 \leq \lambda_{\text{RED}} \leq 680$	Maccioni et al. (2001)
2001	MND4	Maccioni normalized difference 4	ND	$\frac{\rho_{556} - \min(\rho(\lambda_{\text{RED}}))}{\rho_{750} - \min(\rho(\lambda_{\text{RED}}))} : 660 \leq \lambda_{\text{RED}} \leq 680$	Maccioni et al. (2001)
2001	CAINT	Chlorophyll Absorption INTegral (CAINT)	SF	$\sum_{\lambda=600}^{735} \left(\frac{\rho(\lambda)}{y(\lambda)}\right) d\lambda : y(\lambda) = \frac{\rho_{735} - \rho_{600}}{\lambda_{735} - \lambda_{600}}(\lambda - 600) + \rho_{600}$	Oppelt and Mauser (2001, 2004)
2001	ZTSUM	Area of the first derivative peak from 680 nm to 760 nm	SF	$\sum_{\lambda=680}^{760} \rho'(\lambda) d\lambda$	Zarco-Tejada et al. (2001b)
2001	PRI3	Photochemical Reflectance Index 3 (PRI3)	ND	$\frac{\rho_{531} - \rho_{570}}{\rho_{531} + \rho_{570}}$	Zarco-Tejada et al. (2001b)
2001	ZTDPR1	Zarco-Tejada derivative peak ratio 1	SF	$\frac{\rho'_{\lambda_{RDE}}}{\rho'_{\lambda_{RDE}+12}} : \lambda_{RDE}$ from WLREIPG	Zarco-Tejada et al. (2001b); Miller et al. (1990)
2001	ZTDPR2	Zarco-Tejada derivative peak ratio 2	SF	$\frac{\rho'_{\lambda_{RDE}}}{\rho'_{\lambda_{RDE}+22}} : \lambda_{RDE}$ from WLREIPG	Zarco-Tejada et al. (2001b); Miller et al. (1990)

Continued on next page

Table S.1 – Continued from previous page

Year	Code	Description	Type[1]	Formula	Citation
2001	ZTDP21	Zarco-Tejada derivative peak ratio 21	SF	$\frac{\rho'_{\lambda_{RDE}}}{\rho'_{703}}$: λ_{RDE} from WLREIPG	Zarco-Tejada et al. (2001b); Miller et al. (1990)
2001	ZTDP22	Zarco-Tejada derivative peak ratio 22	SF	$\frac{\rho'_{\lambda_{RDE}}}{\rho'_{720}}$: λ_{RDE} from WLREIPG	Zarco-Tejada et al. (2001b); Miller et al. (1990)
2001	GI	Greenness Index	SR	$\frac{\rho_{554}}{\rho_{677}}$	Zarco-Tejada et al. (2001b)
2001	ZTSR3	Zarco-Tejada simple ratio 3	SR	$\frac{\rho_{680}}{\rho_{630}}$	Zarco-Tejada et al. (2001a)
2001	ZTSR4	Zarco-Tejada simple ratio 4	SR	$\frac{\rho_{685}}{\rho_{630}}$	Zarco-Tejada et al. (2001a)
2001	ZTSR5	Zarco-Tejada simple ratio 5	SR	$\frac{\rho_{687}}{\rho_{630}}$	Zarco-Tejada et al. (2001a)
2001	ZTSR6	Zarco-Tejada simple ratio 6	SR	$\frac{\rho_{690}}{\rho_{630}}$	Zarco-Tejada et al. (2001a)

Continued on next page

Table S.1 – Continued from previous page

Year	Code	Description	Type[1]	Formula	Citation
2002	VARI	Visible Atmospherically Resistant Index (VARI)	ND	$\frac{\rho_{\text{GRN}} - \rho_{\text{RED}}}{\rho_{\text{GRN}} + \rho_{\text{RED}} - \rho_{\text{BLU}}}$	Gitelson et al. (2002a)
2002	CRI500	Carotenoid Reflectance Index (CRI550)	EN	$(\rho_{510})^{-1} - (\rho_{550})^{-1}$	Gitelson et al. (2002b)
2002	CRI700	Carotenoid Reflectance Index (CRI700)	EN	$(\rho_{510})^{-1} - (\rho_{700})^{-1}$	Gitelson et al. (2002b)
2002	TCARI	Transformed Chlorophyll Absorption Ratio Index (TCARI)	EN	$3\left[(\rho_{700} - \rho_{670}) - 0.2(\rho_{700} - \rho_{550})\left(\frac{\rho_{700}}{\rho_{670}}\right)\right]$	Haboudane et al. (2002)
2002	TOR	TCARI OSAVI ratio	EN	$\frac{\text{TCARI}}{\text{OSAVI}}$	Haboudane et al. (2002)
2002	EVI	Enhanced Vegetation Index (EVI)	EN	$2.5\left(\frac{\rho_{\text{NIR}} - \rho_{\text{RED}}}{\rho_{\text{NIR}} + 6\rho_{\text{RED}} - 7.5\rho_{\text{BLU}} + 1}\right)$	Huete et al. (2002)
2002	NDNI	Normalized Difference Nitrogen Index (NDNI)	EN	$\frac{\log_{10}(\rho_{1510}^{-1}) - \log_{10}(\rho_{1680}^{-1})}{\log_{10}(\rho_{1510}^{-1}) + \log_{10}(\rho_{1680}^{-1})}$	Serrano et al. (2002)
2002	NDLI	Normalized Difference Lignin Index (NDLI)	EN	$\frac{\log_{10}(\rho_{1754}^{-1}) - \log_{10}(\rho_{1680}^{-1})}{\log_{10}(\rho_{1754}^{-1}) + \log_{10}(\rho_{1680}^{-1})}$	Serrano et al. (2002)

Continued on next page

Year	Code	Description	Type[1]	Formula	Citation
2002	MSR2	Modified Simple Ratio 2	ND	$\frac{\rho_{750} - \rho_{445}}{\rho_{705} - \rho_{445}}$	Sims and Gamon (2002)
2002	SMNDVI	Sims Modified Normalized Difference Vegetation Index	ND	$\frac{\rho_{750} - \rho_{705}}{\rho_{750} + \rho_{705} - 2\rho_{445}}$	Sims and Gamon (2002)
2003	GRRGM	Gitelson Reciprocal Reflectance Green Model	SR	$\left(\frac{\rho_{\mathrm{NIR}}}{\rho_{\mathrm{GRN}}}\right) - 1$	Gitelson et al. (2003, 2005)
2003	GRRREM	Gitelson Reciprocal Reflectance Red Edge Model	SR	$\left(\frac{\rho_{\mathrm{NIR}}}{\rho_{\mathrm{RDE}}}\right) - 1$	Gitelson et al. (2003, 2005)
2003	DPI	Double-Peak Index (DPI)	SF	$\frac{\rho'_{688}\rho'_{710}}{\rho'^{2}_{697}}$	Zarco-Tejada et al. (2003a)
2003	SRWI	Simple Ratio Water Index (SRWI)	SR	$\frac{\rho_{860}}{\rho_{1240}}$	Zarco-Tejada et al. (2003b)
2004	MTCI	MERIS Terrestrial Chlorophyll Index (MTCI)	ND	$\frac{\rho_{754} - \rho_{709}}{\rho_{709} - \rho_{681}}$	Dash and Curran (2004)
2004	WDRVI	Wide Dynamic Range Vegetation Index (WDRVI)	EN	$\frac{a\rho_{\mathrm{NIR}} - \rho_{\mathrm{RED}}}{a\rho_{\mathrm{NIR}} + \rho_{\mathrm{RED}}} : (a = 0.15)$	Gitelson (2004)

Continued on next page

Year	Code	Description	Type[1]	Formula	Citation
2004	MCARI1	Modified Chlorophyll Absorption in Reflectance Index 1 (MCARI1)	EN	$1.2[2.5(\rho_{800} - \rho_{670}) - 1.3(\rho_{800} - \rho_{550})]$	Haboudane et al. (2004)
2004	MCARI2	Modified Chlorophyll Absorption in Reflectance Index 2 (MCARI2)	EN	$\frac{1.5[2.5(\rho_{800} - \rho_{670}) - 1.3(\rho_{800} - \rho_{550})]}{\sqrt{(2\rho_{800} + 1)^2 - (6\rho_{800} - 5\sqrt{\rho_{670}}) - 0.5}}$	Haboudane et al. (2004)
2004	MTVI1	Modified Triangular Vegetation Index 1 (MTVI1)	EN	$1.2[1.2(\rho_{800} - \rho_{550}) - 2.5(\rho_{670} - \rho_{550})]$	Haboudane et al. (2004)
2004	MTVI2	Modified Triangular Vegetation Index 2 (MTVI2)	EN	$\frac{1.5[1.2(\rho_{800} - \rho_{550}) - 2.5(\rho_{670} - \rho_{550})]}{\sqrt{(2\rho_{800} + 1)^2 - (6\rho_{800} - 5\sqrt{\rho_{670}}) - 0.5}}$	Haboudane et al. (2004)
2004	DD	Double Difference index (DD)	DF	$(\rho_{749} - \rho_{720}) - (\rho_{701} - \rho_{672})$	Le Maire et al. (2004)
2005	LCA	Lignin Cellulose Absorption Index (LCA)	EN	$100[(\rho_{2205} - \rho_{2165}) + (\rho_{2205} - \rho_{2330})]$	Daughtry et al. (2005)
2005	RGI	Red Green Pigment Index (RGI)	SR	$\frac{\rho_{690}}{\rho_{550}}$	Zarco-Tejada et al. (2005)
2005	BGI1	Blue Green Pigment Index 1 (BGI1)	SR	$\frac{\rho_{400}}{\rho_{550}}$	Zarco-Tejada et al. (2005)

Continued on next page

Table S.1 – Continued from previous page

Year	Code	Description	Type[1]	Formula	Citation
2005	BGI2	Blue Green Pigment Index 2 (BGI2)	SR	$\frac{\rho_{450}}{\rho_{550}}$	Zarco-Tejada et al. (2005)
2005	BRI1	Blue Red Pigment Index 1 (BRI1)	SR	$\frac{\rho_{400}}{\rho_{690}}$	Zarco-Tejada et al. (2005)
2005	BRI2	Blue Red Pigment Index 2 (BRI2)	SR	$\frac{\rho_{450}}{\rho_{690}}$	Zarco-Tejada et al. (2005)
2006	WLREIPE	Wavelength of red edge inflection point, extrapolation method	SF	$\lambda_{\mathrm{RDE}} = \frac{-(c_1 - c_2)}{m_1 - m_2} : \rho'_1(\lambda) = m_1\lambda + c_1, \rho'_2(\lambda) = m_2\lambda + c_2, m_1 = \frac{\rho'_{700} - \rho'_{680}}{\lambda_{700} - \lambda_{680}}, m_2 = \frac{\rho'_{760} - \rho'_{725}}{\lambda_{760} - \lambda_{725}}$	Cho and Skidmore (2006)
2006	RVIOPT	Reyniers VIopt	EN	$(1 + 0.45)\frac{\rho^2_{\mathrm{NIR}} + 1}{\rho_{\mathrm{RED}} + 0.45}$	Reyniers et al. (2006)
2006	SPVI	Spectral Polygon Vegetation Index (SPVI)	EN	$0.4[3.7(\rho_{800} - \rho_{670}) - 1.2\|\rho_{550} - \rho_{670}\|]$	Vincini et al. (2006)
2007	MMR	MCARI MTVI2 ratio	EN	$\frac{\mathrm{MCARI}}{\mathrm{MTVI2}}$	Eitel et al. (2007)
2008	TCI	Triangular Chlorophyll Index (TCI)	EN	$1.2(\rho_{700} - \rho_{550}) - 1.5(\rho_{670} - \rho_{550})\sqrt{\frac{\rho_{700}}{\rho_{670}}}$	Haboudane et al. (2008)

Continued on next page

Table S.1 – Continued from previous page

Year	Code	Description	Type[1]	Formula	Citation
2008	EVI2	Enhanced Vegetation Index 2 (EVI2)	EN	$\frac{2.5(\rho_{\text{NIR}} - \rho_{\text{RED}})}{\rho_{\text{NIR}} + 2.4\rho_{\text{RED}} + 1}$	Jiang et al. (2008)
2008	DDN	New Double Difference index (DDN)	DF	$2\rho_{710} - \rho_{660} - \rho_{760}$	Le Maire et al. (2008)
2008	CVI	Chlorophyll Vegetation Index (CVI)	EN	$\frac{\rho_{\text{NIR}}\rho_{\text{RED}}}{\rho_{\text{GRN}}^2}$	Vincini et al. (2008)
2008	WUTCARI	Transformed Chlorophyll Absorption Ratio Index [705, 750]	EN	$3\left[(\rho_{750} - \rho_{705}) - 0.2(\rho_{750} - \rho_{550})\left(\frac{\rho_{750}}{\rho_{705}}\right)\right]$	Wu et al. (2008)
2008	WUOSAVI	Optimized Soil-Adjusted Vegetation Index [705, 750]	SA	$(1 + L)\left(\frac{\rho_{750} - \rho_{705}}{\rho_{750} + \rho_{705} + L}\right) : (L = 0.16)$	Wu et al. (2008)
2008	WUMCARI	Modified Chlorophyll Absorption in Reflectance Index [705, 750]	EN	$[(\rho_{750} - \rho_{705}) - 0.2(\rho_{750} - \rho_{550})]\left(\frac{\rho_{750}}{\rho_{705}}\right)$	Wu et al. (2008)
2008	WUMSR	Modified Simple Ratio [705, 750]	EN	$\frac{\rho_{750}/\rho_{705} - 1}{\sqrt{\rho_{750}/\rho_{705} + 1}}$	Wu et al. (2008)
2008	WUTOR	TCARI OSAVI ratio [705, 750]	EN	$\frac{\text{WUTCARI}}{\text{WUOSAVI}}$	Wu et al. (2008)
2008	WUMOR	MCARI OSAVI ratio [705, 750]	EN	$\frac{\text{WUMCARI}}{\text{WUOSAVI}}$	Wu et al. (2008)

Continued on next page

Table S.1 – Continued from previous page

Year	Code	Description	Type[1]	Formula	Citation
2010	DCNI	Double-peak Canopy Nitrogen Index (DCNI)	SF	$\frac{\left(\frac{\rho_{720}-\rho_{700}}{\rho_{700}-\rho_{670}}\right)}{\rho_{720}-\rho_{670}+0.03}$	Chen et al. (2010)
2011	TGI	Triangular Greenness Index (TGI)	EN	$-0.5[(\lambda_{\mathrm{RED}}-\lambda_{\mathrm{BLU}})(\rho_{\mathrm{RED}}-\rho_{\mathrm{GRN}})-(\lambda_{\mathrm{RED}}-\lambda_{\mathrm{GRN}})(\rho_{\mathrm{RED}}-\rho_{\mathrm{BLU}})]$	Hunt et al. (2011)
2011	WDRVI2	Wide Dynamic Range Vegetation Index 2 (WDRVI2)	EN	$\frac{\alpha\rho_{\mathrm{NIR}}-\rho_{\mathrm{RED}}}{\alpha\rho_{\mathrm{NIR}}+\rho_{\mathrm{RED}}}+\frac{1-\alpha}{1+\alpha}:(\alpha=0.2)$	Peng and Gitelson (2011)
2016	AIVI	Angular Insensitivity Vegetation Index (AIVI)	EN	$\frac{\rho_{445}(\rho_{720}+\rho_{735})-\rho_{573}(\rho_{720}-\rho_{735})}{\rho_{720}(\rho_{573}+\rho_{445})}$	He et al. (2016)
2017	DND	Derivative Normalized Difference	SF	$\frac{\rho'_{522}-\rho'_{728}}{\rho'_{522}+\rho'_{728}}$	Sonobe and Wang (2017)

1 Types of spectral vegetation indices include the following: simple ratio (SR), difference (DF), normalized difference (ND), soil-adjusted (SA), spectral feature (SF), and enhanced (EN).

Table S.2: Additional information for each spectral vegetation index (indicated by its year and code from Table S.1), including the evaluated plant species, the scale of the remote sensing data, and the evaluated dependent variables. Information was collected only from the original publication(s) for each spectral index as cited, and use of indices for other plant species and dependent variables can likely be found with further literature review.

Year	Code	Plant Species[1]	Scale	Dependent Variables[2]	Citation
1968	BRSR	Kentucky bluegrass, tall fescue, colonial bentgrass	canopy	visual color scores	Birth and McVey (1968)
1969	JSR	forest canopy	canopy	LAI, Chl *a*	Jordan (1969)
1973	NDVI	*Stipa* and *Bouteloua* genera, rangeland grasses including warm-season grasses (blue grama, buffalograss, sideoats grama, big and little bluestem) and cool-season grasses (western wheatgrass, needle-and-thread, Texas wintergrass)	canopy	green and dry biomass	Rouse et al. (1973)
1977	PVI	sorghum	canopy	crop cover and height, LAI	Richardson and Wiegand (1977)
1978	WLREIP	wheat, alfalfa, cotton, sugar beet, sudan grass, milo, pea, maize, sunflower, silver birch, ash, hawthorn, penduculate oak, winter and spring barley, winter wheat	canopy	Chl	Collins (1978); Horler et al. (1983)
1979	DVI, NDVI2	blue grama grass	canopy	wet and dry biomass, leaf water content, Chl	Tucker (1979)
1988	WLREIP2	maize, rye, mixed grass (*Brachypodium genuense*, quaking-grass, erect brome, *Festuca* species) and herb (snow carpet, *Cirsium creticum*, pygmy hawksbeard, *Lamium garganicum*, common sainfoin, feverfew, red clover)	leaf, canopy	leaf N	Guyot and Baret (1988); Cho and Skidmore (2006)

Continued on next page

Table S.2 – Continued from previous page

Year	Code	Plant Species[1]	Scale	Dependent Variables[2]	Citation
1988	SAVI	cotton, Lehmann lovegrass	canopy	LAI	Huete (1988)
1989	TSAVI	wheat	canopy	LAI, APAR	Baret et al. (1989)
1989	WDVI	barley	canopy	LAI	Clevers (1989)
1989	MSI	California live oak, blue spruce, sweetgum, red spruce, soybean	leaf	leaf relative water content, equivalent water thickness	Hunt and Rock (1989)
1990	BD, BDR	sugar beet, wheat	canopy	plant species, cultivar, N fertilizer rate, sowing date	Boochs et al. (1990)
1990	SAVI2	wheat	canopy	LAI	Major et al. (1990)
1990	WLREIPG, WLCWMRG	burr oak, sugar maple, balsom fir, American beech, black spruce	leaf	none	Miller et al. (1990)
1991	TSAVI2	none	canopy	LAI	Baret and Guyot (1991)
1992	CPSR1	soybean	leaf	Chl *a*	Chappelle et al. (1992)
1992	CPSR2	soybean	leaf	Chl *b*	Chappelle et al. (1992)
1992	CPSR3	soybean	leaf	carotenoid	Chappelle et al. (1992)
1992	PRI	sunflower	leaf, canopy	xanthophyll epoxidation state, photosynthetic efficiency	Gamon et al. (1992)
1992	GEMI	none	canopy	none	Pinty and Verstraete (1992)

Continued on next page

Table S.2 – Continued from previous page

Year	Code	Plant Species[1]	Scale	Dependent Variables[2]	Citation
1993	BMSR, BMLSR, BMDVI	bean	leaf	Chl $a+b$	Buschmann and Nagel (1993)
1993	PSR, PD, WLPD	gerbera, pepper, bean	canopy	leaf relative water content, leaf water potential, leaf conductance, photosynthetic rate	Peñuelas et al. (1993)
1993	VSR, VDR	sugar maple	leaf	Chl $a+b$	Vogelmann et al. (1993)
1994	CRSR1, CRSR2, CRSR3, CRSR4, CRSR5	persimmon, loblolly pine, slash pine, switchcane, golden euonymus, live oak	leaf	physiochemical & biological stress	Carter (1994)
1994	FSUM, DREIP	gerbera, pepper, bean, wheat	canopy	LAI, Chl	Filella and Peñuelas (1994); Filella et al. (1995)
1994	NDVI3, GSUM1, GSUM2	horse chestnut, Norway maple	leaf	Chl a	Gitelson and Merzlyak (1994)
1994	NLI	aspen, corn	canopy	LAI, fPAR	Goel and Qin (1994)
1994	CAR	soybean	leaf	Chl a	Kim et al. (1994)
1994	CARI	soybean	canopy	fPAR, LAI	Kim et al. (1994)
1994	NPCI	sunflower	leaf	Chl, leaf N, net CO_2 uptake, light use efficiency, leaf thickness, leaf starch	Peñuelas et al. (1994)
1994	EGFN	sunflower	leaf	Chl, leaf N	Peñuelas et al. (1994)

Continued on next page

Table S.2 – Continued from previous page

Year	Code	Plant Species[1]	Scale	Dependent Variables[2]	Citation
1994	MSAVI1, MSAVI2	cotton	canopy	% green cover	Qi et al. (1994)
1995	ESUM1, ESUM2	pinyon pine	canopy	LAI, % green cover	Elvidge and Chen (1995)
1995	NDPI, SIPI	maize, wheat, tomato, soybean, sunflower, sugar beet, oak, boxelder maple, succulent	leaf	carotenoid:Chl a (ratio)	Peñuelas et al. (1995a)
1995	SRPI	apple	canopy	carotenoid:Chl a (ratio)	Peñuelas et al. (1995b)
1995	NPQI	apple	canopy	Chl	Peñuelas et al. (1995b)
1995	RDVI	none	canopy	fPAR	Roujean and Breon (1995)
1996	MSR	jack pine, black spruce	canopy	LAI, fPAR	Chen (1996)
1996	PRI2	barley	canopy	xanthophyll epoxidation state, zeaxanthin, photosynthetic efficiency	Filella et al. (1996)
1996	NDWI	unspecified woodland, grassland, and crop species	canopy	vegetation liquid water	Gao (1996)
1996	GTSR1, GTSR2	horse chestnut, Norway maple, tobacco, fig, oleander, hibiscus, common grape vine, rose	leaf	Chl $a+b$, Chl a	Gitelson and Merzlyak (1996, 1997); Lichtenthaler et al. (1996)
1996	GNDVI	horse chestnut, Norway maple	leaf	Chl $a+b$, Chl a	Gitelson et al. (1996)
1996	OSAVI	none	canopy	foliage cover	Rondeaux et al. (1996)

Continued on next page

Table S.2 – Continued from previous page

Year	Code	Plant Species[1]	Scale	Dependent Variables[2]	Citation
1997	WI, WNR	kermes oak, strawberry tree, grey-leaved cistus, Montpellier cistus, Mediterranean false brome, Aleppo pine, evergreen oak, narrow-leaved mock privet, mastic tree	canopy	plant water concentration	Peñuelas et al. (1997)
1998	PSSRA, PSNDA	bracken, beech, oak, boxelder maple, sweet chestnut	leaf	Chl a	Blackburn (1998a,b)
1998	PSSRB, PSNDB	bracken, beech, oak, boxelder maple, sweet chestnut	leaf	Chl b	Blackburn (1998a,b)
1998	PSSRC, PSNDC	bracken, beech, oak, boxelder maple, sweet chestnut	leaf	carotenoid	Blackburn (1998a,b)
1998	DSR1, DSR2	*Eucalyptus* species	leaf	Chl a, Chl b, Chl $a+b$, carotenoid	Datt (1998)
1999	DNDR, DDR1, DDR2	*Eucalyptus* species	leaf	Chl a, Chl $a+b$	Datt (1999a,b)
1999	GMSR	Douglas fir, coast live oak, sunflower	leaf	anthocyanin	Gamon and Surfus (1999)
1999	PSRI	Norway maple, horse chestnut, potato, coleus	leaf	Chl, carotenoid:Chl (ratio)	Merzlyak et al. (1999)
2000	TVI	none	canopy	Chl $a+b$, LAI	Broge and Leblanc (2000)
2000	MCARI, MOR	corn	leaf, canopy	Chl $a+b$, LAI	Daughtry et al. (2000)
2000	ZTSR1, ZTSR2, CI, ZTDR1	sugar maple	leaf, canopy	fluorescence	Zarco-Tejada et al. (2000a,b)
2001	CAI	corn, soybean, wheat	canopy	residue cover	Daughtry (2001)

Continued on next page

Year	Code	Plant Species[1]	Scale	Dependent Variables[2]	Citation
2001	ARI	Norway maple, cotoneaster, dogwood, pelargonium	leaf	anthocyanin	Gitelson et al. (2001)
2001	MND1	croton, spotted elaeagnus, Japanese pittosporum, Benjamin fig	leaf	Chl $a+b$, Chl a, Chl b	Maccioni et al. (2001)
2001	MND2	croton, spotted elaeagnus, Japanese pittosporum, Benjamin fig	leaf	Chl $a+b$	Maccioni et al. (2001)
2001	MND3	croton, spotted elaeagnus, Japanese pittosporum, Benjamin fig	leaf	Chl a	Maccioni et al. (2001)
2001	MND4	croton, spotted elaeagnus, Japanese pittosporum, Benjamin fig	leaf	Chl b	Maccioni et al. (2001)
2001	CAINT	maize, wheat	canopy	leaf N, Chl $a+b$, Chl a, Chl b	Oppelt and Mauser (2001, 2004)
2001	ZTSUM, ZTDPR1, ZTDPR2, ZTDP21, ZTDP22, PRI3, GI	sugar maple	leaf, canopy	Chl $a+b$	Zarco-Tejada et al. (2001b)
2001	ZTSR3, ZTSR4, ZTSR5, ZTSR6	sugar maple	leaf, canopy	fluorescence	Zarco-Tejada et al. (2001a)
2002	VARI	wheat	canopy	vegetation fraction	Gitelson et al. (2002a)
2002	CRI500, CRI700	Norway maple, horse chestnut, beech	leaf	Chl, carotenoid	Gitelson et al. (2002b)
2002	TCARI, TOR	corn	leaf, canopy	Chl	Haboudane et al. (2002)
2002	EVI	grass/shrub, savanna, and tropical forest biomes	canopy	LAI	Huete et al. (2002)

Continued on next page

Table S.2 – Continued from previous page

Year	Code	Plant Species[1]	Scale	Dependent Variables[2]	Citation
2002	NDNI	ceanothus chapparal (*Ceanothus* species), chamise chaparral, coastal sage scrub (*Salvia* species, *Eriogonum* species, California sagebrush)	canopy	leaf and canopy N	Serrano et al. (2002)
2002	NDLI	ceanothus chapparal (*Ceanothus* species), chamise chaparral, coastal sage scrub (*Salvia* species, *Eriogonum* species, California sagebrush)	canopy	leaf and canopy lignin	Serrano et al. (2002)
2002	MSR2, SMNDVI	53 plant species	leaf	Chl	Sims and Gamon (2002)
2003	GRRGM, GRRREM	Norway maple, horse chestnut, beech, wild vine shrub, maize, soybean	leaf	Chl	Gitelson et al. (2003, 2005)
2003	DPI	boxelder maple	canopy	fluorescence	Zarco-Tejada et al. (2003a)
2003	SRWI	various chapparal species (chamise, redshanks, California sagebrush, bigpod ceanothus, greenbark, San Luis purple sage, Californian black sage)	canopy	leaf water content	Zarco-Tejada et al. (2003b)
2004	MTCI	Douglas fir, bigleaf maple	canopy	Chl	Dash and Curran (2004)
2004	WDRVI	wheat, soybean, maize	canopy	LAI, vegetation fraction	Gitelson (2004)
2004	MCARI1, MCARI2, MTVI1, MTVI2	corn, wheat, soybean	leaf, canopy	LAI	Haboudane et al. (2004)
2004	DD	sycamore, *Betula species*, European beech, ash, wild cherry, oak, evergreen oak, *Salix* species	leaf	Chl	Le Maire et al. (2004)

Continued on next page

Table S.2 – Continued from previous page

Year	Code	Plant Species[1]	Scale	Dependent Variables[2]	Citation
2005	LCA	corn, soybean, wheat, tall fescue, alfalfa	canopy	residue cover	Daughtry et al. (2005)
2005	RGI, BGI1, BGI2, BRI1, BRI2	common grape vine	leaf, canopy	Chl $a + b$, Chl a, Chl b	Zarco-Tejada et al. (2005)
2006	WLREIPE	maize, rye, mixed grass (*Brachypodium genuense*, quaking-grass, erect brome, *Festuca* species) and herb (snow carpet, *Cirsium creticum*, pygmy hawksbeard, *Lamium garganicum*, common sainfoin, feverfew, red clover)	leaf, canopy	leaf N	Cho and Skidmore (2006)
2006	RVIOPT	winter wheat	canopy	plant N	Reyniers et al. (2006)
2006	SPVI	maize, sugar beet	canopy	Chl, LAI	Vincini et al. (2006)
2007	MMR	spring wheat	canopy	SPAD meter, leaf N	Eitel et al. (2007)
2008	TCI	corn, wheat, bean, pea	canopy	Chl, LAI	Haboudane et al. (2008)
2008	EVI2	none	canopy	none	Jiang et al. (2008)
2008	DDN	oak, sessile oak, Scots pine, beech	canopy	Chl	Le Maire et al. (2008)
2008	CVI	sugar beet	canopy	Chl $a + b$	Vincini et al. (2008)
2008	WUTCARI, WUOSAVI, WUMCARI, WUMSR, WUTOR, WUMOR	wheat, corn	canopy	Chl, LAI	Wu et al. (2008)
2010	DCNI	wheat, corn	canopy	plant N, LAI	Chen et al. (2010)
2011	TGI	corn, soybean, sorghum, dandelion, sweetgum, tuliptree, small-leaf linden, wheat	leaf, canopy	Chl $a + b$, SPAD meter, LAI	Hunt et al. (2011)

Continued on next page

Table S.2 – Continued from previous page

Year	Code	Plant Species[1]	Scale	Dependent Variables[2]	Citation
2011	WDRVI2	maize, soybean, wheat, oat	canopy	gross primary productivity	Peng and Gitelson (2011)
2016	AIVI	winter wheat	canopy	leaf N	He et al. (2016)
2017	DND	29+ deciduous species	leaf	Chl	Sonobe and Wang (2017)

1 Scientific names for plant species are as follows: Aleppo pine, *Pinus halepensis*; alfalfa, *Medicago sativa*; American beech, *Fagus grandifolia*; apple, *Malus domestica*; ash, *Fraxinus excelsior*; aspen, *Populus tremula*; balsom fir, *Abies balsamea*; barley, *Hordeum vulgare*; bean, *Phaseolus vulgaris*; beech, *Fagas sylvatica*; Benjamin fig, *Ficus benjamina*; big bluestem, *Andropogon gerardi*; bigleaf maple, *Acer macrophyllum*; bigpod ceanothus, *Ceanothus megacarpus*; black spruce, *Picea mariana*; blue grama grass, *Bouteloua gracilis*; blue spruce, *Picea pungens*; boxelder maple, *Acer negundo*; bracken, *Pteridium aquilinum*; buffalograss, *Bouteloua dactyloides*; burr oak, *Quercus macrocarpa*; California live oak, *Quercus agrifolia*; Californian black sage, *Salvia mellifera*; California sagebrush, *Artemisia californica*; chamise chaparral, *Adenostoma fasciculatum*; coast live oak, *Quercus agrifolia*; coleus, *Coleus blumei*; colonial bentgrass, *Argrostis tenuis*; common grape vine, *Vitis vinifera*; common sainfoin, *Onobrychis viciifolia*; corn, *Zea mays*; cotoneaster, *Cotoneaster alaunica*; cotton, *Gossypium hirsutum*; croton, *Codiaeum variegatum*; dandelion, *Taraxacum officinale*; dogwood, *Cornus alba*; Douglas fir, *Pseudotsuga menziesii*; erect brome, *Bromus erectus*; European beech, *Fagus sylvatica*; evergreen oak, *Quercus ilex*; feverfew, *Tanacetum parthenium*; fig, *Ficus carica*; gerbera, *Gerbera jamesonii*; golden euonymus, *Euonymus japonica*; greenbark, *Ceanothus spinosus*; grey-leaved cistus, *Cistus albidus*; hawthorn, *Crataegus monogyna*; hibiscus, *Hibiscus esculentus*; horse chestnut, *Aesculus hippocastanum*; jack pine, *Pinus banksiana*; Japanese pittosporum, *Pittosporum tobira*; Kentucky bluegrass, *Poa protensis*; kermes oak, *Quercus coccifera*; Lehmann lovegrass, *Eragrostis lehmanniana*; little bluestem, *Schizachyrium scoparium*; live oak, *Quercus virginiana*; loblolly pine, *Pinus taeda*; maize, *Zea mays*; mastic tree, *Pistacia lentiscus*; Mediterranean false brome, *Brachypodium retusum*; milo, *Sorghum bicolor*; Montpellier cistus, *Cistus monspeliensis*; narrow-leaved mock privet, *Phillyrea angustifolia*; needle-and-thread, *Hesperostipa comata*; Norway maple, *Acer platanoides*; oak, *Quercus rober*; oleander, *Netrium oleander*; pea, *Pisum sativum*; pelargonium, *Pelargonium zonale*; pendunculate oak, *Quercus robur*; pepper, *Capsicum annuum*; persimmon, *Diospyros virginiana*; pinyon pine, *Pinus edulis*; potato, *Solanum tuberosum*; pygmy hawksbeard, *Crepis pygmaea*; quaking-grass, *Briza media*; red clover, *Trifolium pratense*; redshanks, *Adenostoma sparsifolium*; red spruce, *Picea rubens*; rose, *Rosa rugosa*; rye, *Secale cereale*; San Luis purple sage, *Salvia leucophylla*; Scots pine, *Pinus sylvestris*; sessile oak, *Quercus petraea*; sideoats grama, *Bouteloua curtipendula*; silver birch, *Betula pendula*; slash pine, *Pinus elliotti*; small-leaf linden, *Tilia cordata*; snow carpet, *Anthemis carpatica*; sorghum, *Sorghum bicolor*; soybean, *Glycine max*; spring barley, *Hordeum vulgare*; spring wheat, *Triticum aestivum*; spotted elaeagnus, *Elaeagnus pungens*; strawberry tree, *Arbutus unedo*; succulent, *Othonnopsis cheirifolia*; sudan grass, *Sorghum* × *drummondii*; sugar beet, *Beta vulgaris*; sugar maple, *Acer saccharum*; sunflower, *Helianthus annuus*; sweet chestnut, *Castanea sativa*; sweetgum, *Liquidambar styraciflua*; switchcane, *Arundinaria gigantea*; sycamore, *Acer pseudoplatanus*; tall fescue, *Lolium arundinaceum*; Texas wintergrass, *Nassella leucotricha*; tobacco, *Nicotiana tabacum*; tomato, *Lycopersicon esculentum*; tuliptree, *Liriodendron tulipifera*; western wheatgrass, *Pascopyrum smithii*; wheat, *Triticum aestivum*; wild cherry, *Prunus avium*; wild vine shrub, *Parthenocissus tricuspidata*; winter barley, *Hordeum vulgare*; winter wheat, *Triticum aestivum*

2 Abbreviations for dependent variables are as follows: absorbed photosynthetically active radiation, APAR; chlorophyll, Chl; fraction of photosynthetically active radiation absorbed, fPAR; leaf area index, LAI; nitrogen, N.

Table S.3: Simple linear regression statistics, including root mean squared errors (RMSE, %) and coefficients of determination (r^2), for 148 spectral vegetation indices to estimate area-basis cotton leaf chlorophyll $a + b$ (Chl $a + b$; μg cm^{-2}) for data sets collected during field studies at Maricopa, Arizona, USA. Definitions and formulas for each spectral index are given in Table S.1.

	2019-2020 data			2021-2022 data			All data, 80% random split			All data, 20% random split		
Rank	Index	%RMSE	r^2	Index	%RMSE	r^2	Index	%RMSE	r^2	Index	%RMSE	r^2
1	WLREIPG	8.33	0.7203	GTSR2	16.44	0.6277	WUMCARI	16.27	0.6513	WUMCARI	16.22	0.6852
2	DDN	8.36	0.7184	GSUM1	16.93	0.6054	WUMOR	16.59	0.6375	MSR2	16.53	0.6731
3	DNDR	8.40	0.7157	GRRGM	17.20	0.5927	MSR2	16.84	0.6265	WUMOR	16.82	0.6614
4	DD	8.43	0.7138	WUTOR	17.21	0.5923	WLCWMRG	17.39	0.6018	DCNI	17.11	0.6497
5	MND1	8.46	0.7114	GTSR1	17.25	0.5904	MTCI	17.40	0.6010	GSUM1	17.27	0.6430
6	WLREIP2	8.56	0.7048	WUMSR	17.27	0.5895	DCNI	17.48	0.5976	ZTSR2	17.29	0.6421
7	MND3	8.57	0.7045	GSUM2	17.35	0.5854	ZTSR2	17.54	0.5949	GTSR2	17.33	0.6405
8	MTCI	8.75	0.6916	BMLSR	17.44	0.5812	DDR1	17.57	0.5931	WLCWMRG	17.48	0.6344
9	WLREIPE	8.85	0.6842	ZTSR2	17.58	0.5743	GSUM1	17.68	0.5883	WUMSR	17.50	0.6337
10	VDR	8.87	0.6830	NDVI3	17.60	0.5737	SMNDVI	17.75	0.5851	MTCI	17.58	0.6304
11	WLCWMRG	8.88	0.6824	GNDVI	17.65	0.5709	WUMSR	17.75	0.5850	SMNDVI	17.80	0.6209
12	MMR	8.95	0.6774	CRSR3	17.69	0.5690	WUOSAVI	17.82	0.5817	WUOSAVI	17.89	0.6168
13	ZTDR1	8.99	0.6743	CPSR2	17.70	0.5688	GTSR2	17.85	0.5801	VSR	17.90	0.6165
14	ZTDP22	9.03	0.6716	WUMCARI	17.70	0.5686	ZTDP21	17.90	0.5781	DDR1	17.94	0.6148
15	VSR	9.03	0.6714	WUOSAVI	17.80	0.5635	VSR	17.90	0.5780	NDVI3	18.20	0.6037
16	DDR1	9.05	0.6699	BMSR	17.89	0.5592	WLREIP	17.94	0.5758	WLREIP	18.30	0.5992
17	CRSR4	9.14	0.6637	VSR	18.10	0.5491	WLREIPG	18.09	0.5690	WLREIPG	18.48	0.5912
18	SMNDVI	9.17	0.6614	MSR2	18.11	0.5483	DD	18.11	0.5678	CRSR4	18.53	0.5889
19	WUMCARI	9.28	0.6534	CRSR4	18.16	0.5458	DDN	18.16	0.5656	DDN	18.68	0.5825
20	WUOSAVI	9.28	0.6533	WUMOR	18.29	0.5393	NDVI3	18.22	0.5625	DD	18.70	0.5816
21	ZTSR2	9.32	0.6498	CRSR2	18.44	0.5317	WLREIPE	18.35	0.5565	ZTDP21	18.71	0.5811
22	MSR2	9.36	0.6470	SMNDVI	18.50	0.5287	CRSR4	18.39	0.5545	WLREIPE	18.78	0.5780
23	MOR	9.44	0.6411	DCNI	18.67	0.5200	MND3	18.48	0.5502	MND3	18.92	0.5716
24	ZTDPR1	9.61	0.6281	WLCWMRG	18.75	0.5159	MND1	18.50	0.5490	MND1	18.94	0.5709
25	MCARI	9.63	0.6264	MTCI	18.85	0.5110	DNDR	18.51	0.5485	DNDR	18.94	0.5707
26	CARI	9.63	0.6262	ARI	18.97	0.5043	ZTDR1	18.57	0.5460	VDR	19.10	0.5636
27	NDVI3	9.68	0.6226	WLREIP	19.13	0.4963	VDR	18.59	0.5450	ZTDR1	19.19	0.5595
28	WUMSR	9.72	0.6196	WLREIPG	19.16	0.4947	BDR	18.59	0.5447	WLREIP2	19.54	0.5430
29	DCNI	9.78	0.6147	DNDR	19.27	0.4890	WLREIP2	18.78	0.5354	BDR	19.57	0.5417
30	WLREIP	9.79	0.6139	TOR	19.28	0.4884	DDR2	19.52	0.4980	CPSR2	19.74	0.5335

Continued on next page

Table S.3 – Continued from previous page

Rank	2019-2020 data			2021-2022 data			All data, 80% random split			All data, 20% random split		
	Index	%RMSE	r^2	Index	%RMSE	r^2	Index	%RMSE	r^2	Index	%RMSE	r^2
31	CI	9.84	0.6103	WLREIPE	19.28	0.4883	CRSR1	19.89	0.4790	CRSR1	19.93	0.5249
32	CRSR5	10.07	0.5912	DD	19.30	0.4870	CPSR2	20.06	0.4698	BRI2	19.98	0.5221
33	GSUM1	10.11	0.5880	VDR	19.32	0.4860	CPSR1	20.29	0.4579	DDR2	20.65	0.4895
34	ZTSR3	10.28	0.5740	DDN	19.34	0.4849	MMR	20.32	0.4560	CI	21.12	0.4664
35	WUMOR	10.37	0.5670	MND3	19.35	0.4847	CI	20.42	0.4505	MMR	21.19	0.4627
36	CAR	10.54	0.5528	MND1	19.35	0.4844	GRRREM	20.49	0.4469	CPSR1	21.46	0.4490
37	ZTDPR2	10.55	0.5514	DDR1	19.36	0.4838	CRSR5	20.80	0.4300	CRSR5	21.54	0.4448
38	ZTSR4	10.65	0.5435	ZTDR1	19.40	0.4819	CAR	21.20	0.4082	CRSR3	21.56	0.4437
39	GTSR2	10.71	0.5384	EGFN	19.46	0.4786	MOR	21.29	0.4031	CAR	21.77	0.4327
40	CPSR1	10.83	0.5275	AIVI	19.56	0.4731	CRSR3	21.29	0.4028	ZTSR3	21.94	0.4242
41	DDR2	10.98	0.5143	WLREIP2	19.71	0.4653	ZTSR3	21.30	0.4023	GRRREM	22.08	0.4165
42	ZTSR5	11.00	0.5127	DND	19.81	0.4596	BRI2	21.47	0.3927	MOR	22.27	0.4064
43	CRSR1	11.16	0.4982	CAR	19.92	0.4538	MCARI	21.62	0.3842	MCARI	22.65	0.3863
44	CPSR2	11.29	0.4868	ZTDP21	19.98	0.4504	CARI	21.62	0.3840	CARI	22.65	0.3862
45	TCI	11.31	0.4848	MND4	20.05	0.4468	ZTDP22	21.95	0.3651	ZTSR4	22.70	0.3835
46	ZTDP21	11.43	0.4734	PSSRB	20.05	0.4464	ZTSR4	21.97	0.3643	AIVI	22.74	0.3810
47	BD	11.69	0.4496	TCARI	20.11	0.4433	AIVI	22.35	0.3420	ZTDP22	23.30	0.3501
48	ESUM2	11.87	0.4321	TGI	20.13	0.4419	ZTSR5	22.50	0.3333	ZTSR5	23.31	0.3496
49	AIVI	12.20	0.4007	MND2	20.24	0.4362	BD	22.52	0.3318	BD	23.45	0.3420
50	TCARI	12.30	0.3910	BRI2	20.30	0.4326	ZTDPR1	22.66	0.3234	TCI	23.67	0.3296
51	ZTSR6	12.32	0.3883	PSNDB	20.35	0.4298	TCI	22.88	0.3104	ZTDPR1	23.81	0.3214
52	ZTSR1	12.33	0.3876	BDR	20.49	0.4221	TOR	23.39	0.2795	TOR	23.93	0.3146
53	TOR	12.35	0.3860	BMDVI	20.58	0.4171	TCARI	23.56	0.2687	WUTOR	23.99	0.3116
54	GRRREM	12.36	0.3842	CVI	20.78	0.4053	WUTOR	23.57	0.2680	TCARI	24.21	0.2989
55	DREIP	12.47	0.3733	DSR1	20.83	0.4023	SPVI	23.70	0.2599	CRSR2	24.28	0.2948
56	CRSR3	12.73	0.3469	DDR2	20.88	0.4000	CRSR2	23.92	0.2466	BRI1	24.34	0.2912
57	BDR	12.84	0.3361	MMR	21.06	0.3895	ZTDPR2	24.01	0.2406	PSSRB	24.50	0.2819
58	WUTOR	12.93	0.3262	BD	21.07	0.3887	BRI1	24.08	0.2359	SPVI	24.62	0.2745
59	CRSR2	12.95	0.3247	CAINT	21.08	0.3882	BMDVI	24.30	0.2223	PSNDB	24.76	0.2664
60	DND	13.26	0.2920	TCI	21.13	0.3853	PSSRB	24.37	0.2174	BMDVI	25.08	0.2475
61	BMDVI	13.45	0.2712	CRSR1	21.15	0.3839	NDLI	24.39	0.2162	CAINT	25.47	0.2237
62	GI	13.54	0.2611	MOR	21.37	0.3712	PSNDB	24.46	0.2118	ZTDPR2	25.48	0.2228
63	MND4	13.64	0.2505	CI	21.53	0.3618	ZTSR6	24.68	0.1979	ZTSR1	25.64	0.2132

Continued on next page

Table S.3 – Continued from previous page

Rank	2019-2020 data			2021-2022 data			All data, 80% random split			All data, 20% random split		
	Index	%RMSE	r^2	Index	%RMSE	r^2	Index	%RMSE	r^2	Index	%RMSE	r^2
64	MND2	13.70	0.2442	ZTDP22	21.59	0.3582	EVI	24.71	0.1955	ZTSR6	25.69	0.2102
65	TGI	13.74	0.2397	ZTDPR1	21.62	0.3563	ZTSR1	24.83	0.1881	EVI	26.03	0.1895
66	PSSRB	13.79	0.2336	CARI	21.78	0.3468	PVI	25.13	0.1683	DND	26.11	0.1842
67	BMSR	13.80	0.2325	MCARI	21.78	0.3468	WDVI	25.13	0.1683	PRI3	26.47	0.1616
68	PSNDB	13.82	0.2303	WUTCARI	21.84	0.3433	DND	25.13	0.1678	PVI	26.52	0.1582
69	MCARI2	13.97	0.2141	GRRREM	21.85	0.3430	DVI	25.14	0.1676	WDVI	26.52	0.1582
70	MTVI2	13.97	0.2141	ZTSR3	21.98	0.3351	CAINT	25.14	0.1672	GEMI	26.56	0.1561
71	GNDVI	14.04	0.2063	ZTSR4	22.00	0.3335	FSUM	25.23	0.1614	DVI	26.58	0.1549
72	DSR1	14.09	0.2001	ZTSR5	22.08	0.3287	GEMI	25.28	0.1578	NDLI	26.65	0.1498
73	SPVI	14.20	0.1874	BGI1	22.12	0.3262	PRI3	25.62	0.1353	FSUM	26.68	0.1480
74	NDVI2	14.36	0.1698	BGI2	22.36	0.3116	DPI	25.65	0.1334	ESUM2	26.78	0.1415
75	BMLSR	14.39	0.1656	CRSR5	22.37	0.3113	ZTSUM	25.71	0.1296	MND4	26.82	0.1396
76	NDNI	14.41	0.1640	CPSR1	22.61	0.2962	MND4	25.79	0.1237	BMSR	26.87	0.1360
77	MCARI1	14.57	0.1450	ZTDPR2	22.62	0.2958	ESUM2	25.80	0.1234	TGI	26.94	0.1313
78	MTVI1	14.57	0.1450	ZTSR6	23.02	0.2704	GI	25.88	0.1180	MND2	26.97	0.1296
79	EGFN	14.57	0.1444	PRI3	23.03	0.2699	BMSR	25.91	0.1153	CAI	26.97	0.1294
80	PSRI	14.61	0.1404	SPVI	23.18	0.2600	MND2	25.93	0.1145	EVI2	27.14	0.1189
81	BGI2	14.62	0.1387	ESUM2	23.32	0.2511	EVI2	25.93	0.1144	RDVI	27.18	0.1159
82	VARI	14.63	0.1376	BRI1	23.42	0.2445	TGI	25.94	0.1135	ZTSUM	27.22	0.1132
83	DSR2	14.64	0.1362	GI	23.76	0.2227	RDVI	25.96	0.1120	GI	27.23	0.1130
84	GMSR	14.66	0.1349	NDVI2	23.93	0.2113	RVIOPT	26.04	0.1065	RVIOPT	27.27	0.1100
85	TVI	14.71	0.1289	DSR2	24.01	0.2062	DSR1	26.12	0.1014	GNDVI	27.28	0.1097
86	BGI1	14.80	0.1178	GMSR	24.02	0.2054	SAVI	26.16	0.0988	DSR1	27.28	0.1095
87	NDLI	14.84	0.1134	EVI	24.12	0.1993	GNDVI	26.23	0.0938	SAVI	27.35	0.1047
88	PRI	14.84	0.1130	PRI2	24.21	0.1933	PD	26.29	0.0893	SRPI	27.39	0.1021
89	GRRGM	14.85	0.1119	PRI	24.36	0.1832	CRI700	26.50	0.0751	NPCI	27.43	0.0999
90	BRI2	14.86	0.1101	DREIP	24.49	0.1740	BMLSR	26.71	0.0602	PRI2	27.84	0.0723
91	BRI1	14.87	0.1099	DPI	24.67	0.1618	EGFN	26.72	0.0595	SIPI	27.87	0.0707
92	GTSR1	14.88	0.1082	ZTSR1	24.68	0.1616	DREIP	26.75	0.0576	BMLSR	27.88	0.0697
93	CAINT	14.92	0.1037	VARI	24.89	0.1468	BGI2	26.75	0.0575	MSAVI2	27.89	0.0695
94	DPI	14.95	0.0998	GEMI	24.99	0.1403	MSAVI2	26.75	0.0573	DREIP	27.94	0.0656
95	WUTCARI	14.97	0.0979	EVI2	25.10	0.1323	CAI	26.76	0.0570	NDPI	27.95	0.0655
96	RGI	15.00	0.0943	RGI	25.11	0.1322	NDVI2	26.76	0.0567	MSAVI1	27.96	0.0643

Continued on next page

Table S.3 – Continued from previous page

Rank	2019-2020 data			2021-2022 data			All data, 80% random split			All data, 20% random split		
	Index	%RMSE	r^2	Index	%RMSE	r^2	Index	%RMSE	r^2	Index	%RMSE	r^2
97	GSUM2	15.04	0.0886	MCARI1	25.12	0.1315	MCARI2	26.82	0.0524	BGI2	28.01	0.0612
98	CVI	15.07	0.0853	MTVI1	25.12	0.1315	MTVI2	26.82	0.0524	CRI700	28.02	0.0604
99	PD	15.19	0.0711	RDVI	25.13	0.1307	MSAVI1	26.86	0.0498	EGFN	28.06	0.0581
100	LCA	15.20	0.0698	SAVI	25.16	0.1282	PRI	26.86	0.0495	NDVI2	28.12	0.0535
101	CRI500	15.23	0.0661	SIPI	25.17	0.1278	NDNI	26.90	0.0469	MCARI2	28.20	0.0481
102	NPQI	15.28	0.0599	RVIOPT	25.19	0.1262	WUTCARI	26.97	0.0419	MTVI2	28.20	0.0481
103	SIPI	15.37	0.0479	CRI700	25.32	0.1174	CRI500	26.98	0.0410	WUTCARI	28.31	0.0412
104	ARI	15.47	0.0361	PVI	25.36	0.1147	SRPI	26.99	0.0403	BGI1	28.35	0.0381
105	DVI	15.52	0.0299	WDVI	25.36	0.1147	NPCI	27.00	0.0397	PRI	28.42	0.0335
106	PVI	15.53	0.0286	MSAVI2	25.46	0.1076	BGI1	27.00	0.0395	DSR2	28.45	0.0312
107	WDVI	15.53	0.0286	DVI	25.49	0.1055	PRI2	27.02	0.0383	GMSR	28.46	0.0306
108	CPSR3	15.58	0.0224	TVI	25.54	0.1022	DSR2	27.09	0.0330	PD	28.47	0.0299
109	BRSR	15.58	0.0219	FSUM	25.57	0.0998	GMSR	27.10	0.0327	TSAVI2	28.53	0.0263
110	FSUM	15.59	0.0216	MSAVI1	25.57	0.0995	NDPI	27.18	0.0271	GRRGM	28.54	0.0251
111	GEMI	15.59	0.0216	MCARI2	25.58	0.0989	VARI	27.18	0.0269	VARI	28.57	0.0230
112	CAI	15.59	0.0214	MTVI2	25.58	0.0989	SIPI	27.18	0.0269	DPI	28.58	0.0224
113	ESUM1	15.59	0.0205	TSAVI2	25.86	0.0795	GRRGM	27.23	0.0230	OSAVI	28.59	0.0217
114	MSI	15.65	0.0136	NDLI	25.87	0.0782	BRSR	27.28	0.0200	GTSR1	28.59	0.0216
115	PRI2	15.67	0.0113	OSAVI	25.93	0.0746	PSNDC	27.28	0.0199	NDNI	28.60	0.0214
116	EVI2	15.67	0.0113	NLI	25.98	0.0706	GTSR1	27.28	0.0198	ARI	28.62	0.0201
117	ZTSUM	15.67	0.0110	ZTSUM	26.01	0.0689	PSSRC	27.29	0.0188	NLI	28.62	0.0199
118	RDVI	15.67	0.0110	PSRI	26.04	0.0665	ESUM1	27.30	0.0179	MCARI1	28.70	0.0143
119	RVIOPT	15.68	0.0100	CRI500	26.28	0.0491	WI	27.33	0.0164	MTVI1	28.70	0.0143
120	PSNDA	15.68	0.0093	SAVI2	26.30	0.0474	PSR	27.33	0.0163	CRI500	28.71	0.0138
121	PSSRA	15.69	0.0089	NPCI	26.31	0.0468	TSAVI2	27.34	0.0155	PSSRC	28.74	0.0119
122	JSR	15.69	0.0086	SRPI	26.36	0.0431	NPQI	27.35	0.0146	PSNDC	28.74	0.0119
123	SAVI	15.69	0.0084	PSNDA	26.44	0.0378	OSAVI	27.39	0.0115	NPQI	28.74	0.0118
124	WLPD	15.70	0.0069	TSAVI	26.49	0.0338	ARI	27.40	0.0108	TVI	28.75	0.0111
125	NDPI	15.70	0.0068	NDVI	26.53	0.0307	LCA	27.41	0.0103	SAVI2	28.79	0.0082
126	PRI3	15.70	0.0067	WNR	26.57	0.0283	NLI	27.42	0.0097	SRWI	28.80	0.0077
127	PSSRC	15.70	0.0066	WDRVI2	26.58	0.0275	MCARI1	27.43	0.0089	NDWI	28.80	0.0077
128	WNR	15.71	0.0062	PSSRA	26.59	0.0267	MTVI1	27.43	0.0089	GSUM2	28.80	0.0077
129	NDVI	15.71	0.0056	WDRVI	26.59	0.0266	CVI	27.44	0.0083	CVI	28.80	0.0076

Continued on next page

Table S.3 – Continued from previous page

	2019-2020 data			2021-2022 data			All data, 80% random split			All data, 20% random split		
Rank	Index	%RMSE	r^2	Index	%RMSE	r^2	Index	%RMSE	r^2	Index	%RMSE	r^2
130	WDRVI2	15.71	0.0054	MSR	26.64	0.0232	GSUM2	27.45	0.0075	BRSR	28.83	0.0057
131	WDRVI	15.71	0.0053	JSR	26.69	0.0194	RGI	27.46	0.0066	ESUM1	28.85	0.0040
132	NPCI	15.72	0.0053	MSI	26.80	0.0113	TVI	27.47	0.0061	WLPD	28.85	0.0040
133	SRPI	15.72	0.0051	NDPI	26.84	0.0082	JSR	27.47	0.0057	RGI	28.87	0.0030
134	MSR	15.72	0.0051	PD	26.86	0.0069	MSI	27.47	0.0056	MSI	28.88	0.0017
135	TSAVI	15.72	0.0050	WLPD	26.87	0.0061	WNR	27.48	0.0052	LCA	28.90	0.0008
136	PSNDC	15.72	0.0047	NDNI	26.88	0.0050	CPSR3	27.49	0.0043	PSRI	28.90	0.0006
137	CRI700	15.72	0.0045	NPQI	26.90	0.0041	MSR	27.50	0.0040	PSNDA	28.90	0.0003
138	MSAVI2	15.74	0.0027	LCA	26.90	0.0038	WDRVI	27.50	0.0036	TSAVI	28.90	0.0002
139	MSAVI1	15.74	0.0020	BRSR	26.91	0.0031	WDRVI2	27.50	0.0035	PSSRA	28.91	0.0002
140	PSR	15.75	0.0014	SRWI	26.92	0.0023	PSSRA	27.51	0.0033	CPSR3	28.91	0.0001
141	WI	15.75	0.0014	NDWI	26.92	0.0022	NDVI	27.51	0.0031	JSR	28.91	0.0001
142	SAVI2	15.75	0.0014	CPSR3	26.93	0.0012	WLPD	27.52	0.0023	NDVI	28.91	0.0000
143	NDWI	15.75	0.0007	PSSRC	26.94	0.0008	PSNDA	27.52	0.0022	WNR	28.91	0.0000
144	SRWI	15.75	0.0007	CAI	26.94	0.0005	TSAVI	27.53	0.0019	WDRVI2	28.91	0.0000
145	EVI	15.76	0.0002	WI	26.95	0.0001	SAVI2	27.54	0.0011	WDRVI	28.91	0.0000
146	OSAVI	15.76	0.0001	PSR	26.95	0.0001	PSRI	27.55	0.0001	MSR	28.91	0.0000
147	NLI	15.76	0.0001	ESUM1	26.95	0.0000	NDWI	27.55	0.0000	PSR	28.91	0.0000
148	TSAVI2	15.76	0.0000	PSNDC	26.95	0.0000	SRWI	27.55	0.0000	WI	28.91	0.0000

Table S.4: Simple linear regression statistics, including root mean squared errors (RMSE, %) and coefficients of determination (r^2), for 148 spectral vegetation indices to estimate area-basis cotton leaf chlorophyll *a* (Chl *a*; μg cm^{-2}) for data sets collected during field studies at Maricopa, Arizona, USA. Definitions and formulas for each spectral index are given in Table S.1.

	2019-2020 data			2021-2022 data			All data, 80% random split			All data, 20% random split		
Rank	Index	%RMSE	r^2	Index	%RMSE	r^2	Index	%RMSE	r^2	Index	%RMSE	r^2
1	DDN	8.40	0.7019	GTSR2	16.98	0.6057	WUMCARI	14.62	0.6604	WUMCARI	15.00	0.6762
2	DD	8.47	0.6974	GSUM1	17.35	0.5884	WUMOR	14.98	0.6435	MSR2	15.41	0.6582
3	WLREIPG	8.51	0.6940	GRRGM	17.43	0.5845	MSR2	15.24	0.6310	WUMOR	15.62	0.6486
4	DNDR	8.56	0.6903	GTSR1	17.51	0.5807	ZTSR2	15.54	0.6161	ZTSR2	15.68	0.6458
5	MND1	8.57	0.6898	WUMSR	17.61	0.5759	WLCWMRG	15.59	0.6136	GSUM1	15.68	0.6458
6	MND3	8.64	0.6851	WUTOR	17.63	0.5751	MTCI	15.61	0.6126	GTSR2	15.72	0.6440
7	WLREIP2	8.78	0.6743	BMLSR	17.71	0.5713	GSUM1	15.75	0.6056	WUMSR	15.86	0.6380
8	VDR	8.79	0.6738	GSUM2	17.72	0.5708	WUMSR	15.76	0.6051	DCNI	16.07	0.6282
9	MTCI	8.82	0.6717	ZTSR2	17.81	0.5662	VSR	15.77	0.6045	WLCWMRG	16.14	0.6249
10	WLREIPE	8.82	0.6713	GNDVI	17.93	0.5604	WUOSAVI	15.80	0.6030	VSR	16.16	0.6239
11	WLCWMRG	8.82	0.6712	NDVI3	17.93	0.5603	SMNDVI	15.85	0.6005	WUOSAVI	16.22	0.6213
12	ZTDP22	8.85	0.6689	WUMCARI	17.99	0.5574	DDR1	15.88	0.5991	MTCI	16.23	0.6205
13	WUMCARI	8.86	0.6682	WUOSAVI	18.12	0.5511	DCNI	15.94	0.5963	SMNDVI	16.33	0.6162
14	ZTDR1	8.89	0.6665	BMSR	18.17	0.5484	GTSR2	15.96	0.5954	NDVI3	16.43	0.6111
15	VSR	8.92	0.6642	VSR	18.23	0.5458	WLREIPG	16.07	0.5895	DDR1	16.62	0.6024
16	CRSR4	9.05	0.6542	CRSR4	18.37	0.5387	DDN	16.09	0.5887	CRSR4	16.69	0.5988
17	WUOSAVI	9.14	0.6475	MSR2	18.38	0.5381	DD	16.10	0.5882	WLREIPG	16.85	0.5910
18	SMNDVI	9.15	0.6462	CRSR3	18.43	0.5356	NDVI3	16.14	0.5859	DDN	16.97	0.5851
19	ZTSR2	9.22	0.6412	CPSR2	18.44	0.5349	WLREIP	16.15	0.5855	WLREIP	16.98	0.5849
20	MMR	9.23	0.6401	WUMOR	18.53	0.5304	CRSR4	16.22	0.5820	DD	17.03	0.5823
21	ZTDPR1	9.23	0.6401	SMNDVI	18.75	0.5194	WLREIPE	16.25	0.5805	WLREIPE	17.07	0.5804
22	MSR2	9.35	0.6312	WLCWMRG	19.00	0.5064	ZTDR1	16.32	0.5765	DNDR	17.20	0.5740
23	DDR1	9.48	0.6205	MTCI	19.03	0.5048	VDR	16.36	0.5745	MND1	17.21	0.5737
24	NDVI3	9.54	0.6156	DCNI	19.04	0.5041	DNDR	16.39	0.5729	MND3	17.22	0.5731
25	WUMSR	9.58	0.6121	WLREIPG	19.26	0.4925	MND1	16.40	0.5727	VDR	17.25	0.5715
26	WUMOR	9.72	0.6008	WLREIP	19.29	0.4909	MND3	16.41	0.5722	ZTDR1	17.31	0.5684
27	MOR	9.84	0.5910	VDR	19.32	0.4898	ZTDP21	16.62	0.5610	WLREIP2	17.75	0.5462
28	WLREIP	9.86	0.5894	ZTDR1	19.33	0.4891	WLREIP2	16.72	0.5558	ZTDP21	17.80	0.5437
29	DCNI	9.89	0.5871	DDR1	19.35	0.4882	DDR2	17.34	0.5224	CPSR2	18.00	0.5336
30	CI	9.94	0.5826	WLREIPE	19.35	0.4882	BDR	17.34	0.5222	CRSR1	18.56	0.5043

Continued on next page

Table S.4 – Continued from previous page

Rank	2019-2020 data			2021-2022 data			All data, 80% random split			All data, 20% random split		
	Index	%RMSE	r^2	Index	%RMSE	r^2	Index	%RMSE	r^2	Index	%RMSE	r^2
31	GSUM1	9.95	0.5823	DNDR	19.36	0.4874	MMR	18.05	0.4820	BRI2	18.57	0.5033
32	MCARI	10.04	0.5748	DDN	19.41	0.4850	CPSR2	18.09	0.4796	BDR	18.65	0.4991
33	CARI	10.04	0.5745	DD	19.44	0.4831	CRSR1	18.31	0.4674	DDR2	18.70	0.4968
34	ZTDPR2	10.10	0.5691	CRSR2	19.46	0.4823	CI	18.43	0.4600	MMR	19.19	0.4700
35	CRSR5	10.29	0.5532	MND1	19.46	0.4819	CPSR1	18.50	0.4558	CRSR3	19.37	0.4598
36	GTSR2	10.49	0.5352	EGFN	19.50	0.4799	GRRREM	18.65	0.4471	CI	19.43	0.4563
37	DDR2	10.51	0.5334	MND3	19.51	0.4798	CRSR5	18.67	0.4458	CRSR5	19.74	0.4390
38	ZTSR3	10.67	0.5189	TOR	19.54	0.4780	MOR	18.93	0.4304	CAR	19.76	0.4380
39	CAR	10.87	0.5007	AIVI	19.68	0.4705	CAR	18.98	0.4277	CPSR1	20.04	0.4220
40	ZTSR4	11.03	0.4865	DND	19.73	0.4680	CRSR3	19.07	0.4222	MOR	20.12	0.4169
41	CPSR1	11.04	0.4853	WLREIP2	19.83	0.4621	ZTDP22	19.19	0.4145	ZTSR3	20.34	0.4044
42	CPSR2	11.28	0.4629	ARI	19.88	0.4594	MCARI	19.24	0.4115	MCARI	20.47	0.3966
43	ZTSR5	11.38	0.4536	CAR	20.11	0.4472	CARI	19.25	0.4113	CARI	20.47	0.3964
44	CRSR1	11.40	0.4514	MND4	20.16	0.4441	ZTSR3	19.42	0.4004	GRRREM	20.55	0.3919
45	ZTDP21	11.44	0.4478	TGI	20.21	0.4416	BRI2	19.73	0.3812	ZTDP22	20.71	0.3823
46	BD	11.64	0.4275	ZTDP21	20.23	0.4402	ZTDPR1	19.73	0.3810	AIVI	20.84	0.3749
47	TCI	11.71	0.4210	MND2	20.26	0.4386	BD	19.95	0.3677	ZTSR4	20.98	0.3665
48	ESUM2	11.85	0.4075	TCARI	20.27	0.4384	ZTSR4	20.01	0.3639	BD	21.05	0.3617
49	CRSR3	12.34	0.3572	BRI2	20.57	0.4217	AIVI	20.22	0.3500	ZTDPR1	21.09	0.3594
50	DREIP	12.35	0.3559	BMDVI	20.65	0.4169	ZTSR5	20.49	0.3329	TCI	21.47	0.3361
51	GRRREM	12.38	0.3525	DDR2	20.67	0.4160	TCI	20.53	0.3302	ZTSR5	21.52	0.3331
52	ZTSR1	12.43	0.3481	BDR	20.74	0.4119	ZTDPR2	21.00	0.2990	WUTOR	21.68	0.3235
53	AIVI	12.53	0.3366	CVI	20.77	0.4103	TOR	21.08	0.2937	TOR	21.74	0.3197
54	TCARI	12.58	0.3320	DSR1	20.87	0.4046	WUTOR	21.23	0.2837	CRSR2	21.99	0.3035
55	TOR	12.60	0.3301	BD	20.95	0.3996	TCARI	21.23	0.2834	TCARI	22.00	0.3033
56	ZTSR6	12.60	0.3298	PSSRB	21.00	0.3972	CRSR2	21.66	0.2546	PSSRB	22.08	0.2982
57	BDR	12.71	0.3177	MMR	21.05	0.3941	SPVI	21.69	0.2520	PSNDB	22.37	0.2795
58	CRSR2	12.93	0.2944	TCI	21.13	0.3894	BMDVI	22.08	0.2250	BRI1	22.51	0.2701
59	WUTOR	13.06	0.2803	MOR	21.33	0.3778	PSSRB	22.08	0.2249	ZTDPR2	22.60	0.2648
60	DND	13.47	0.2338	PSNDB	21.34	0.3771	PSNDB	22.17	0.2191	SPVI	22.65	0.2612
61	PSSRB	13.49	0.2314	ZTDPR1	21.35	0.3769	NDLI	22.21	0.2159	BMDVI	22.91	0.2443
62	PSNDB	13.50	0.2307	CRSR1	21.35	0.3767	BRI1	22.37	0.2046	CAINT	22.93	0.2430
63	BMDVI	13.56	0.2232	ZTDP22	21.42	0.3729	ZTSR6	22.51	0.1949	ZTSR6	23.62	0.1967

Continued on next page

Table S.4 – Continued from previous page

Rank	2019-2020 data			2021-2022 data			All data, 80% random split			All data, 20% random split		
	Index	%RMSE	r^2	Index	%RMSE	r^2	Index	%RMSE	r^2	Index	%RMSE	r^2
64	GI	13.75	0.2021	CARI	21.70	0.3559	DND	22.70	0.1809	ZTSR1	23.64	0.1951
65	MND4	13.80	0.1960	MCARI	21.70	0.3559	EVI	22.72	0.1795	DND	23.70	0.1914
66	MND2	13.85	0.1901	CI	21.83	0.3484	CAINT	22.73	0.1786	EVI	23.97	0.1727
67	TGI	13.89	0.1857	GRRREM	21.86	0.3467	ZTSR1	22.79	0.1747	ESUM2	24.04	0.1681
68	BMSR	13.90	0.1842	CAINT	21.88	0.3452	ESUM2	23.02	0.1579	NDLI	24.27	0.1519
69	NDNI	13.92	0.1821	WUTCARI	22.07	0.3338	PVI	23.05	0.1556	GEMI	24.34	0.1472
70	SPVI	14.04	0.1672	ZTDPR2	22.25	0.3233	WDVI	23.05	0.1556	PRI3	24.35	0.1463
71	GNDVI	14.10	0.1606	CRSR5	22.29	0.3205	DVI	23.06	0.1546	PVI	24.36	0.1454
72	MCARI2	14.13	0.1566	BGI2	22.33	0.3182	DPI	23.07	0.1541	WDVI	24.36	0.1454
73	MTVI2	14.13	0.1566	BGI1	22.36	0.3165	FSUM	23.15	0.1484	BMSR	24.40	0.1430
74	DSR1	14.19	0.1495	ZTSR3	22.41	0.3134	GEMI	23.18	0.1462	MND4	24.41	0.1423
75	CAINT	14.39	0.1250	ZTSR4	22.48	0.3089	MND4	23.39	0.1305	DVI	24.43	0.1408
76	BMLSR	14.40	0.1247	ZTSR5	22.59	0.3021	GI	23.46	0.1251	TGI	24.50	0.1360
77	NDVI2	14.42	0.1214	CPSR1	22.66	0.2978	BMSR	23.48	0.1235	FSUM	24.52	0.1342
78	BRI2	14.45	0.1185	ESUM2	23.26	0.2604	PRI3	23.50	0.1221	MND2	24.54	0.1327
79	PSRI	14.46	0.1167	SPVI	23.30	0.2579	TGI	23.51	0.1218	EVI2	24.74	0.1184
80	MCARI1	14.56	0.1042	GI	23.62	0.2372	MND2	23.51	0.1216	RDVI	24.78	0.1158
81	MTVI1	14.56	0.1042	ZTSR6	23.69	0.2328	ZTSUM	23.57	0.1174	GNDVI	24.78	0.1157
82	EGFN	14.61	0.0988	NDVI2	23.82	0.2242	EVI2	23.70	0.1075	GI	24.84	0.1117
83	VARI	14.64	0.0951	DSR2	23.94	0.2165	RDVI	23.72	0.1055	RVIOPT	24.84	0.1113
84	NDLI	14.64	0.0950	GMSR	23.94	0.2164	DSR1	23.73	0.1053	DSR1	24.88	0.1090
85	BGI2	14.65	0.0940	BRI1	24.02	0.2109	PD	23.73	0.1049	SAVI	24.92	0.1061
86	DSR2	14.65	0.0933	PRI3	24.08	0.2068	RVIOPT	23.79	0.1008	ZTSUM	24.98	0.1013
87	GMSR	14.66	0.0921	EVI	24.26	0.1956	GNDVI	23.79	0.1005	DREIP	25.12	0.0913
88	TVI	14.67	0.0912	DREIP	24.26	0.1951	SAVI	23.89	0.0933	SRPI	25.15	0.0891
89	BRI1	14.69	0.0889	DPI	24.34	0.1899	DREIP	23.94	0.0889	NPCI	25.18	0.0872
90	LCA	14.72	0.0856	PRI	24.79	0.1597	CRI700	24.17	0.0714	CAI	25.30	0.0784
91	PD	14.75	0.0816	VARI	24.83	0.1567	BMLSR	24.26	0.0647	BMLSR	25.36	0.0743
92	GRRGM	14.77	0.0790	RGI	25.01	0.1449	NDNI	24.26	0.0647	MSAVI2	25.36	0.0742
93	GTSR1	14.79	0.0762	MCARI1	25.20	0.1317	EGFN	24.30	0.0616	MSAVI1	25.42	0.0696
94	BGI1	14.79	0.0761	MTVI1	25.20	0.1317	NDVI2	24.33	0.0589	PRI2	25.46	0.0664
95	DPI	14.81	0.0744	PRI2	25.22	0.1301	MCARI2	24.35	0.0580	SIPI	25.47	0.0661
96	PRI	14.81	0.0740	GEMI	25.22	0.1300	MTVI2	24.35	0.0580	BGI2	25.57	0.0589

Continued on next page

Table S.4 – Continued from previous page

Rank	2019-2020 data			2021-2022 data			All data, 80% random split			All data, 20% random split		
	Index	%RMSE	r^2	Index	%RMSE	r^2	Index	%RMSE	r^2	Index	%RMSE	r^2
97	WUTCARI	14.87	0.0665	CRI700	25.28	0.1263	BGI2	24.36	0.0572	EGFN	25.58	0.0580
98	GSUM2	14.92	0.0595	SIPI	25.37	0.1197	MSAVI2	24.39	0.0544	NDPI	25.58	0.0576
99	RGI	14.93	0.0593	EVI2	25.40	0.1178	MSAVI1	24.49	0.0470	NDVI2	25.66	0.0517
100	CVI	14.96	0.0550	RDVI	25.42	0.1162	PRI	24.50	0.0459	CRI700	25.67	0.0513
101	SIPI	15.08	0.0393	NDLI	25.45	0.1141	WUTCARI	24.51	0.0449	MCARI2	25.72	0.0476
102	DVI	15.12	0.0345	ZTSR1	25.46	0.1136	CRI500	24.60	0.0384	MTVI2	25.72	0.0476
103	PVI	15.12	0.0341	SAVI	25.47	0.1128	BGI1	24.62	0.0368	PD	25.74	0.0460
104	WDVI	15.12	0.0341	RVIOPT	25.48	0.1123	PRI2	24.66	0.0339	WUTCARI	25.75	0.0449
105	CRI500	15.13	0.0339	PVI	25.50	0.1108	DSR2	24.66	0.0338	NDNI	25.86	0.0375
106	NPQI	15.16	0.0294	WDVI	25.50	0.1108	GMSR	24.66	0.0335	BGI1	25.88	0.0359
107	GEMI	15.17	0.0278	MCARI2	25.51	0.1099	SRPI	24.72	0.0291	TSAVI2	25.93	0.0322
108	FSUM	15.18	0.0265	MTVI2	25.51	0.1099	CAI	24.72	0.0288	DSR2	25.96	0.0299
109	MSI	15.21	0.0234	TVI	25.60	0.1039	NPCI	24.72	0.0288	GMSR	25.96	0.0294
110	CPSR3	15.22	0.0219	DVI	25.61	0.1033	VARI	24.73	0.0279	DPI	25.97	0.0291
111	EVI2	15.25	0.0183	FSUM	25.68	0.0983	LCA	24.76	0.0259	PRI	25.97	0.0291
112	RDVI	15.25	0.0182	MSAVI2	25.78	0.0913	GRRGM	24.77	0.0250	GRRGM	25.99	0.0276
113	ARI	15.25	0.0177	MSAVI1	25.89	0.0836	SIPI	24.79	0.0233	OSAVI	25.99	0.0274
114	RVIOPT	15.25	0.0174	PSRI	26.06	0.0711	WI	24.81	0.0221	NLI	26.01	0.0257
115	ZTSUM	15.27	0.0156	ZTSUM	26.12	0.0669	PSR	24.81	0.0220	GTSR1	26.04	0.0240
116	SAVI	15.27	0.0149	TSAVI2	26.15	0.0647	GTSR1	24.81	0.0216	VARI	26.06	0.0221
117	ESUM1	15.28	0.0147	OSAVI	26.22	0.0602	MSI	24.82	0.0205	ARI	26.10	0.0188
118	BRSR	15.28	0.0138	CRI500	26.24	0.0583	BRSR	24.85	0.0187	MCARI1	26.12	0.0178
119	PRI3	15.28	0.0135	NLI	26.27	0.0561	NDPI	24.87	0.0168	MTVI1	26.12	0.0178
120	CAI	15.29	0.0122	MSI	26.47	0.0418	PSNDC	24.87	0.0167	TVI	26.16	0.0145
121	WLPD	15.32	0.0094	NPCI	26.49	0.0403	PSSRC	24.88	0.0166	SAVI2	26.18	0.0129
122	PSSRC	15.32	0.0084	SRPI	26.54	0.0370	TSAVI2	24.89	0.0157	CRI500	26.23	0.0096
123	MSAVI2	15.33	0.0071	SAVI2	26.56	0.0355	ESUM1	24.91	0.0135	GSUM2	26.23	0.0090
124	PSNDC	15.34	0.0069	PSNDA	26.65	0.0292	MCARI1	24.93	0.0126	LCA	26.24	0.0089
125	NPCI	15.34	0.0062	PD	26.70	0.0249	MTVI1	24.93	0.0126	CVI	26.25	0.0080
126	SRPI	15.34	0.0061	TSAVI	26.72	0.0241	OSAVI	24.94	0.0118	PSSRC	26.27	0.0064
127	MSAVI1	15.34	0.0058	NDVI	26.75	0.0215	ARI	24.95	0.0105	NPQI	26.27	0.0062
128	PSR	15.35	0.0053	LCA	26.77	0.0204	NLI	24.96	0.0101	PSNDC	26.27	0.0062
129	WI	15.35	0.0053	PSSRA	26.78	0.0191	TVI	24.96	0.0095	WLPD	26.29	0.0050

Continued on next page

Table S.4 – Continued from previous page

	2019-2020 data			2021-2022 data			All data, 80% random split			All data, 20% random split		
Rank	Index	%RMSE	r^2	Index	%RMSE	r^2	Index	%RMSE	r^2	Index	%RMSE	r^2
130	PSNDA	15.35	0.0045	WDRVI2	26.79	0.0187	CVI	24.98	0.0086	RGI	26.32	0.0029
131	PSSRA	15.35	0.0044	WDRVI	26.80	0.0179	GSUM2	24.98	0.0084	BRSR	26.32	0.0023
132	NDWI	15.36	0.0043	WNR	26.81	0.0174	RGI	25.00	0.0070	ESUM1	26.32	0.0023
133	JSR	15.36	0.0042	MSR	26.84	0.0150	WLPD	25.02	0.0050	PSNDA	26.33	0.0019
134	SRWI	15.36	0.0042	JSR	26.87	0.0126	JSR	25.02	0.0048	PSSRA	26.33	0.0018
135	NDPI	15.36	0.0036	NPQI	26.89	0.0110	WNR	25.03	0.0046	TSAVI	26.33	0.0017
136	WNR	15.36	0.0033	WLPD	26.95	0.0068	NPQI	25.03	0.0043	PSR	26.33	0.0016
137	PRI2	15.37	0.0023	NDPI	26.98	0.0044	CPSR3	25.04	0.0035	WI	26.33	0.0016
138	NDVI	15.37	0.0022	CAI	26.99	0.0042	MSR	25.04	0.0032	MSI	26.34	0.0012
139	WDRVI2	15.37	0.0020	PSSRC	26.99	0.0037	WDRVI	25.05	0.0028	WDRVI2	26.34	0.0010
140	WDRVI	15.37	0.0020	PSR	27.02	0.0021	WDRVI2	25.05	0.0027	NDVI	26.34	0.0010
141	CRI700	15.37	0.0019	WI	27.02	0.0020	PSSRA	25.05	0.0027	WDRVI	26.34	0.0010
142	MSR	15.37	0.0019	NDWI	27.02	0.0018	SRWI	25.05	0.0026	MSR	26.34	0.0009
143	TSAVI	15.38	0.0017	SRWI	27.02	0.0017	NDWI	25.05	0.0025	SRWI	26.34	0.0008
144	TSAVI2	15.38	0.0009	PSNDC	27.03	0.0009	NDVI	25.06	0.0022	NDWI	26.34	0.0008
145	NLI	15.38	0.0005	BRSR	27.03	0.0008	PSNDA	25.07	0.0014	PSRI	26.35	0.0005
146	OSAVI	15.39	0.0005	NDNI	27.04	0.0002	TSAVI	25.07	0.0013	WNR	26.35	0.0004
147	EVI	15.39	0.0002	ESUM1	27.04	0.0002	SAVI2	25.07	0.0012	JSR	26.35	0.0004
148	SAVI2	15.39	0.0000	CPSR3	27.04	0.0000	PSRI	25.08	0.0001	CPSR3	26.35	0.0002

Table S.5: Simple linear regression statistics, including root mean squared errors (RMSE, %) and coefficients of determination (r^2), for 148 spectral vegetation indices to estimate area-basis cotton leaf chlorophyll b (Chl b; μg cm^{-2}) for data sets collected during field studies at Maricopa, Arizona, USA. Definitions and formulas for each spectral index are given in Table S.1.

	2019-2020 data			2021-2022 data			All data, 80% random split			All data, 20% random split		
Rank	Index	%RMSE	r^2	Index	%RMSE	r^2	Index	%RMSE	r^2	Index	%RMSE	r^2
1	MOR	19.18	0.4246	CRSR2	24.35	0.4799	ZTDP21	34.49	0.4544	WUMCARI	32.64	0.5595
2	CARI	19.20	0.4237	CPSR2	24.55	0.4714	BDR	34.75	0.4461	MSR2	32.74	0.5567
3	MCARI	19.20	0.4237	GTSR2	24.62	0.4684	WUMCARI	34.91	0.4411	WUMOR	33.01	0.5494
4	DDR1	19.21	0.4233	CRSR3	24.83	0.4592	DCNI	34.98	0.4388	DCNI	33.10	0.5470
5	ZTSR3	19.35	0.4149	ARI	24.95	0.4538	MSR2	34.99	0.4385	ZTDP21	33.52	0.5355
6	MMR	19.48	0.4065	GSUM1	25.21	0.4424	WUMOR	35.07	0.4360	BDR	34.13	0.5183
7	ZTSR4	19.52	0.4041	WUTOR	25.27	0.4400	DDR1	35.77	0.4130	WLCWMRG	34.29	0.5137
8	WLREIP2	19.65	0.3965	WUMSR	25.62	0.4244	WLCWMRG	35.88	0.4096	DDR1	34.43	0.5099
9	ZTSR5	19.65	0.3962	GSUM2	25.68	0.4213	MTCI	35.92	0.4084	MTCI	34.46	0.5090
10	TCI	19.66	0.3955	PSSRB	25.70	0.4204	WLREIP	36.23	0.3979	GSUM1	34.62	0.5045
11	WLREIPG	19.72	0.3918	PSNDB	25.72	0.4196	SMNDVI	36.50	0.3890	GTSR2	34.62	0.5045
12	CAR	19.75	0.3902	NDVI3	25.82	0.4152	GSUM1	36.62	0.3850	WLREIP	34.79	0.4995
13	DNDR	19.82	0.3858	GTSR1	25.86	0.4133	ZTSR2	36.69	0.3825	ZTSR2	34.84	0.4981
14	CRSR1	19.87	0.3825	GRRGM	25.90	0.4114	GTSR2	36.70	0.3822	SMNDVI	34.91	0.4962
15	MND1	19.97	0.3765	BMLSR	25.93	0.4099	WUMSR	36.84	0.3775	WUMSR	35.03	0.4927
16	CRSR5	20.01	0.3738	GNDVI	26.02	0.4059	CRSR1	37.00	0.3722	WUOSAVI	35.53	0.4780
17	MND3	20.09	0.3692	WUOSAVI	26.07	0.4037	WUOSAVI	37.03	0.3712	BRI2	35.71	0.4726
18	DD	20.14	0.3661	ZTSR2	26.16	0.3997	WLREIPG	37.11	0.3682	VSR	35.86	0.4684
19	DDN	20.14	0.3660	BMSR	26.17	0.3993	DD	37.19	0.3656	NDVI3	36.05	0.4627
20	MTCI	20.17	0.3639	WUMCARI	26.25	0.3956	VSR	37.33	0.3608	WLREIPG	36.09	0.4614
21	AIVI	20.18	0.3636	DCNI	26.41	0.3883	DDN	37.39	0.3587	CRSR1	36.22	0.4574
22	CPSR1	20.34	0.3532	CRSR4	26.53	0.3826	NDVI3	37.48	0.3557	DD	36.38	0.4526
23	CI	20.35	0.3528	MSR2	26.53	0.3825	WLREIPE	37.56	0.3529	DDN	36.47	0.4500
24	DCNI	20.36	0.3519	SMNDVI	26.70	0.3746	MND3	37.57	0.3526	CRSR4	36.67	0.4441
25	WLREIPE	20.50	0.3432	PRI3	26.74	0.3727	MND1	37.70	0.3482	WLREIPE	36.68	0.4438
26	ZTSR6	20.50	0.3429	VSR	26.75	0.3723	DNDR	37.77	0.3457	MND3	36.73	0.4421
27	SMNDVI	20.51	0.3423	WLCWMRG	26.84	0.3681	WLREIP2	37.80	0.3447	MND1	36.84	0.4388
28	TCARI	20.51	0.3422	WUMOR	26.84	0.3680	CRSR4	37.86	0.3425	DNDR	36.89	0.4374
29	WLCWMRG	20.52	0.3414	CAINT	26.99	0.3609	CPSR1	37.86	0.3425	VDR	37.38	0.4223
30	MSR2	20.59	0.3368	TOR	27.05	0.3583	VDR	38.11	0.3338	WLREIP2	37.51	0.4181

Continued on next page

Table S.5 – Continued from previous page

	2019-2020 data			2021-2022 data			All data, 80% random split			All data, 20% random split		
Rank	Index	%RMSE	r^2	Index	%RMSE	r^2	Index	%RMSE	r^2	Index	%RMSE	r^2
31	WLREIP	20.60	0.3368	MTCI	27.14	0.3536	ZTDR1	38.17	0.3317	CPSR2	37.54	0.4174
32	VDR	20.64	0.3336	WLREIP	27.22	0.3501	GRRREM	38.18	0.3313	ZTDR1	37.57	0.4164
33	TOR	20.71	0.3291	MND3	27.45	0.3391	CPSR2	38.36	0.3250	CPSR1	38.32	0.3929
34	ZTDR1	20.76	0.3260	WLREIPG	27.47	0.3382	CI	38.81	0.3091	GRRREM	38.67	0.3816
35	CRSR4	20.77	0.3257	PRI2	27.49	0.3370	BRI2	38.85	0.3078	CI	38.97	0.3721
36	VSR	20.80	0.3239	DD	27.54	0.3345	DDR2	38.88	0.3068	DDR2	39.28	0.3619
37	ZTSR2	20.90	0.3172	DNDR	27.56	0.3337	ZTSR3	39.12	0.2979	ZTSR3	39.61	0.3513
38	ZTDP22	20.93	0.3147	MND1	27.58	0.3326	CRSR5	39.52	0.2839	CRSR5	39.62	0.3510
39	WUOSAVI	20.98	0.3118	CAR	27.68	0.3277	MMR	39.60	0.2807	MMR	39.84	0.3436
40	NDVI3	21.14	0.3015	WLREIPE	27.69	0.3273	ZTSR4	39.98	0.2668	CRSR3	40.24	0.3306
41	WUMSR	21.14	0.3012	DDN	27.76	0.3240	CAR	40.17	0.2600	CAR	40.40	0.3251
42	DND	21.19	0.2979	WLREIP2	27.78	0.3231	CRSR3	40.53	0.2466	ZTSR4	40.73	0.3141
43	GI	21.19	0.2977	ZTDP21	27.85	0.3196	ZTSR5	40.60	0.2439	AIVI	41.00	0.3049
44	ZTSR1	21.21	0.2968	AIVI	27.85	0.3196	MOR	40.69	0.2407	BRI1	41.15	0.2999
45	CPSR2	21.30	0.2905	TCARI	27.92	0.3163	BRI1	40.86	0.2343	MOR	41.35	0.2929
46	MCARI2	21.36	0.2865	VDR	27.92	0.3159	AIVI	40.99	0.2296	ZTSR5	41.54	0.2865
47	MTVI2	21.36	0.2865	EGFN	27.95	0.3146	MCARI	41.02	0.2281	MCARI	41.82	0.2767
48	GSUM1	21.42	0.2825	DDR1	27.99	0.3129	CARI	41.03	0.2280	CARI	41.83	0.2766
49	ZTDP21	21.48	0.2786	MND4	28.08	0.3082	TCI	42.07	0.1882	SPVI	42.62	0.2490
50	MND4	21.52	0.2757	ZTDR1	28.18	0.3033	BD	42.19	0.1837	TCI	42.90	0.2389
51	WUTOR	21.55	0.2736	TGI	28.18	0.3032	SPVI	42.29	0.1796	TOR	43.05	0.2338
52	TGI	21.57	0.2723	BDR	28.20	0.3024	TOR	42.42	0.1748	BD	43.12	0.2312
53	MND2	21.59	0.2713	BRI2	28.21	0.3017	ZTDP22	42.47	0.1727	WUTOR	43.27	0.2258
54	ESUM2	21.64	0.2680	ZTSR5	28.43	0.2910	ZTSR1	42.59	0.1682	TCARI	43.41	0.2206
55	BD	21.67	0.2658	DND	28.44	0.2905	TCARI	42.60	0.1678	CRSR2	43.49	0.2181
56	WUMCARI	21.67	0.2658	MND2	28.46	0.2892	CAI	42.65	0.1656	EVI	43.62	0.2131
57	ZTDPR1	21.72	0.2621	ZTSR4	28.47	0.2890	WUTOR	42.72	0.1629	ZTDP22	43.64	0.2126
58	GTSR2	21.84	0.2541	ZTSR6	28.54	0.2855	CRSR2	42.86	0.1575	CAI	43.64	0.2124
59	BMDVI	21.88	0.2513	ZTSR3	28.61	0.2817	EVI	42.94	0.1542	PSSRB	43.91	0.2027
60	GRRREM	21.93	0.2484	CI	28.66	0.2795	ZTSR6	43.04	0.1503	BMDVI	44.01	0.1990
61	DSR1	21.95	0.2467	CRSR1	28.84	0.2704	BMDVI	43.23	0.1427	PSNDB	44.14	0.1944
62	BMSR	21.97	0.2453	DSR1	28.85	0.2700	PRI3	43.39	0.1363	ZTDPR1	44.55	0.1795
63	NDVI2	22.15	0.2328	WUTCARI	29.00	0.2622	PSSRB	43.44	0.1345	ZTSR1	44.55	0.1795

Continued on next page

Table S.5 – Continued from previous page

Rank	2019-2020 data			2021-2022 data			All data, 80% random split			All data, 20% random split		
	Index	%RMSE	r^2	Index	%RMSE	r^2	Index	%RMSE	r^2	Index	%RMSE	r^2
64	CRSR2	22.21	0.2290	BMDVI	29.03	0.2605	NDLI	43.45	0.1342	ZTSR6	44.77	0.1714
65	GNDVI	22.22	0.2280	ZTSR1	29.05	0.2598	PSNDB	43.51	0.1317	PRI3	44.89	0.1668
66	DREIP	22.33	0.2204	CVI	29.05	0.2596	ZTDPR1	43.52	0.1315	PVI	44.97	0.1636
67	EGFN	22.38	0.2169	MMR	29.11	0.2565	DVI	43.66	0.1256	WDVI	44.97	0.1636
68	ZTDPR2	22.41	0.2149	TCI	29.12	0.2561	PVI	43.69	0.1243	DVI	45.01	0.1623
69	BGI2	22.41	0.2147	BD	29.26	0.2491	WDVI	43.69	0.1243	FSUM	45.16	0.1568
70	MCARI1	22.55	0.2052	MOR	29.42	0.2405	FSUM	43.75	0.1223	GEMI	45.16	0.1568
71	MTVI1	22.55	0.2052	GRRREM	29.52	0.2353	GEMI	43.97	0.1132	CAINT	45.19	0.1558
72	BGI1	22.55	0.2051	BGI1	29.72	0.2252	ZTSUM	44.32	0.0993	NDLI	45.78	0.1336
73	DSR2	22.56	0.2044	BRI1	29.74	0.2242	DND	44.46	0.0935	DND	45.92	0.1283
74	GMSR	22.57	0.2038	CARI	29.84	0.2190	CAINT	44.48	0.0927	ZTSUM	46.04	0.1237
75	VARI	22.58	0.2026	MCARI	29.84	0.2189	ZTDPR2	44.87	0.0768	SRPI	46.04	0.1235
76	BDR	22.61	0.2004	DDR2	29.85	0.2185	EVI2	44.92	0.0746	NPCI	46.10	0.1211
77	DDR2	22.63	0.1990	ZTDP22	29.96	0.2123	MND4	44.93	0.0743	EVI2	46.36	0.1112
78	BMLSR	22.64	0.1987	CPSR1	30.22	0.1986	RDVI	44.98	0.0720	RDVI	46.46	0.1076
79	NPQI	22.70	0.1942	ZTDPR1	30.24	0.1979	GI	44.99	0.0716	RVIOPT	46.63	0.1007
80	TVI	22.75	0.1910	CRSR5	30.33	0.1932	CRI700	45.10	0.0673	ZTDPR2	46.65	0.1000
81	WUMOR	22.78	0.1885	PRI	30.37	0.1907	MND2	45.10	0.0669	MND4	46.68	0.0991
82	PRI	22.83	0.1853	BGI2	30.38	0.1906	RVIOPT	45.11	0.0668	SAVI	46.76	0.0959
83	RGI	23.08	0.1670	ESUM2	30.80	0.1679	TGI	45.13	0.0658	BMSR	46.86	0.0920
84	CRI500	23.16	0.1615	SPVI	31.17	0.1475	DSR1	45.14	0.0657	TGI	46.90	0.0905
85	GRRGM	23.25	0.1549	ZTDPR2	31.23	0.1446	BMSR	45.17	0.0644	MND2	46.90	0.0903
86	WUTCARI	23.29	0.1521	GI	31.64	0.1220	SAVI	45.22	0.0623	SIPI	47.08	0.0834
87	GTSR1	23.30	0.1510	DSR2	31.68	0.1195	DPI	45.25	0.0609	NDPI	47.11	0.0822
88	CVI	23.41	0.1429	NDNI	31.70	0.1182	SRPI	45.35	0.0566	GI	47.14	0.0811
89	CRSR3	23.46	0.1391	NDVI2	31.71	0.1178	NPCI	45.38	0.0557	DSR1	47.15	0.0806
90	BRI1	23.47	0.1389	GMSR	31.72	0.1174	GNDVI	45.47	0.0518	PRI2	47.28	0.0755
91	GSUM2	23.50	0.1369	MCARI1	31.86	0.1094	NDPI	45.61	0.0457	CRI700	47.33	0.0738
92	PSRI	23.54	0.1334	MTVI1	31.86	0.1094	PRI	45.66	0.0438	GNDVI	47.34	0.0733
93	SPVI	23.64	0.1259	EVI	31.89	0.1078	NPQI	45.66	0.0438	ESUM2	47.62	0.0623
94	DPI	23.81	0.1136	SRWI	32.08	0.0971	PRI2	45.69	0.0426	MSAVI2	47.69	0.0596
95	PSSRB	23.82	0.1131	NDWI	32.10	0.0960	ESUM2	45.73	0.0409	MSAVI1	47.81	0.0547
96	PSNDB	23.88	0.1081	SAVI	32.10	0.0958	BGI2	45.74	0.0403	BGI2	47.96	0.0487

Continued on next page

Table S.5 – Continued from previous page

	2019-2020 data			2021-2022 data			All data, 80% random split			All data, 20% random split		
Rank	Index	%RMSE	r^2	Index	%RMSE	r^2	Index	%RMSE	r^2	Index	%RMSE	r^2
97	ARI	24.07	0.0945	EVI2	32.12	0.0948	EGFN	45.82	0.0369	BMLSR	48.07	0.0445
98	NDLI	24.11	0.0910	RDVI	32.14	0.0936	NDVI2	45.85	0.0359	EGFN	48.08	0.0442
99	PRI2	24.49	0.0626	DREIP	32.17	0.0923	CRI500	45.89	0.0343	NDWI	48.18	0.0401
100	CAI	24.62	0.0519	MSAVI2	32.17	0.0919	MSAVI2	45.92	0.0330	SRWI	48.18	0.0401
101	BRI2	24.73	0.0442	CAI	32.18	0.0918	BMLSR	45.94	0.0322	NDVI2	48.21	0.0389
102	BRSR	24.74	0.0431	RVIOPT	32.20	0.0905	BGI1	45.95	0.0317	PRI	48.33	0.0342
103	SIPI	24.79	0.0391	SIPI	32.22	0.0892	MCARI2	45.96	0.0311	BGI1	48.36	0.0330
104	NDNI	24.82	0.0372	MSAVI1	32.24	0.0883	MTVI2	45.96	0.0311	MSI	48.40	0.0315
105	ESUM1	24.83	0.0362	GEMI	32.28	0.0860	MSAVI1	46.02	0.0288	NPQI	48.40	0.0315
106	NDPI	24.95	0.0266	TVI	32.28	0.0859	PSNDC	46.07	0.0264	MCARI2	48.42	0.0304
107	PSNDA	24.95	0.0266	VARI	32.35	0.0817	SIPI	46.07	0.0264	MTVI2	48.42	0.0304
108	JSR	24.96	0.0259	TSAVI2	32.41	0.0782	WUTCARI	46.10	0.0254	WUTCARI	48.53	0.0263
109	PSSRA	24.96	0.0259	OSAVI	32.46	0.0754	PD	46.11	0.0250	PSNDC	48.56	0.0251
110	NDVI	25.01	0.0218	NLI	32.50	0.0735	PSSRC	46.15	0.0230	PSSRC	48.57	0.0245
111	TSAVI	25.02	0.0216	RGI	32.64	0.0655	NDWI	46.18	0.0221	ARI	48.60	0.0232
112	WDRVI2	25.02	0.0215	SAVI2	32.75	0.0591	SRWI	46.18	0.0220	DSR2	48.63	0.0221
113	WDRVI	25.02	0.0215	CRI700	32.75	0.0590	BRSR	46.19	0.0215	GMSR	48.64	0.0217
114	MSR	25.02	0.0211	PVI	32.76	0.0582	DSR2	46.20	0.0212	CRI500	48.70	0.0192
115	SAVI2	25.06	0.0177	WDVI	32.76	0.0582	GMSR	46.20	0.0209	TSAVI2	48.72	0.0184
116	NLI	25.07	0.0172	DPI	32.79	0.0568	MSI	46.29	0.0171	VARI	48.81	0.0149
117	WNR	25.09	0.0159	WNR	32.82	0.0553	VARI	46.30	0.0168	OSAVI	48.82	0.0144
118	OSAVI	25.09	0.0158	MCARI2	32.86	0.0530	ESUM1	46.34	0.0151	DREIP	48.84	0.0139
119	TSAVI2	25.10	0.0149	MTVI2	32.86	0.0530	GRRGM	46.44	0.0111	GRRGM	48.84	0.0137
120	CRI700	25.11	0.0142	DVI	32.91	0.0501	ARI	46.45	0.0106	NLI	48.87	0.0126
121	CAINT	25.11	0.0140	TSAVI	32.95	0.0477	GTSR1	46.48	0.0092	DPI	48.88	0.0120
122	PD	25.13	0.0124	MSI	32.96	0.0471	JSR	46.49	0.0089	ESUM1	48.89	0.0117
123	EVI	25.14	0.0119	PSNDA	32.97	0.0462	CPSR3	46.52	0.0074	GTSR1	48.90	0.0113
124	CPSR3	25.16	0.0106	FSUM	32.98	0.0457	MSR	46.53	0.0071	BRSR	48.93	0.0100
125	LCA	25.17	0.0094	NDVI	32.99	0.0452	WDRVI	46.53	0.0070	PD	48.96	0.0089
126	MSAVI1	25.19	0.0078	WDRVI2	33.02	0.0433	WDRVI2	46.53	0.0070	LCA	48.97	0.0086
127	SRWI	25.20	0.0075	WDRVI	33.03	0.0427	NDVI	46.53	0.0068	CPSR3	49.08	0.0039
128	MSAVI2	25.20	0.0074	MSR	33.08	0.0398	WNR	46.54	0.0067	SAVI2	49.09	0.0037
129	NDWI	25.20	0.0074	PSSRA	33.09	0.0395	DREIP	46.55	0.0063	MCARI1	49.09	0.0037

Continued on next page

Table S.5 – Continued from previous page

Rank	2019-2020 data			2021-2022 data			All data, 80% random split			All data, 20% random split		
	Index	%RMSE	r^2	Index	%RMSE	r^2	Index	%RMSE	r^2	Index	%RMSE	r^2
130	WI	25.21	0.0062	WI	33.09	0.0392	PSSRA	46.55	0.0063	MTVI1	49.09	0.0037
131	PSR	25.21	0.0061	PSR	33.10	0.0386	PSNDA	46.55	0.0060	CVI	49.09	0.0036
132	SAVI	25.25	0.0032	NPCI	33.12	0.0378	TSAVI2	46.56	0.0056	WLPD	49.09	0.0034
133	DVI	25.25	0.0031	SRPI	33.18	0.0344	TSAVI	46.57	0.0053	GSUM2	49.11	0.0028
134	RVIOPT	25.25	0.0030	JSR	33.19	0.0338	LCA	46.58	0.0048	PSR	49.11	0.0027
135	RDVI	25.26	0.0022	LCA	33.20	0.0331	CVI	46.60	0.0042	WI	49.11	0.0026
136	PVI	25.26	0.0020	PD	33.23	0.0315	OSAVI	46.61	0.0035	TVI	49.12	0.0023
137	WDVI	25.26	0.0020	PSRI	33.24	0.0304	RGI	46.62	0.0033	JSR	49.14	0.0016
138	PRI3	25.27	0.0019	ZTSUM	33.26	0.0297	GSUM2	46.63	0.0028	NDNI	49.14	0.0016
139	EVI2	25.27	0.0018	CRI500	33.51	0.0150	NLI	46.63	0.0026	RGI	49.15	0.0012
140	MSI	25.27	0.0015	NDPI	33.53	0.0139	MCARI1	46.63	0.0025	MSR	49.16	0.0009
141	FSUM	25.28	0.0010	BRSR	33.53	0.0135	MTVI1	46.63	0.0025	WDRVI	49.16	0.0008
142	GEMI	25.29	0.0003	CPSR3	33.54	0.0131	NDNI	46.65	0.0021	WDRVI2	49.16	0.0008
143	WLPD	25.29	0.0003	ESUM1	33.65	0.0067	TVI	46.66	0.0014	NDVI	49.16	0.0008
144	PSSRC	25.29	0.0001	PSNDC	33.66	0.0062	WI	46.67	0.0011	PSRI	49.17	0.0005
145	ZTSUM	25.29	0.0001	PSSRC	33.69	0.0042	PSR	46.67	0.0011	PSSRA	49.17	0.0003
146	PSNDC	25.29	0.0001	WLPD	33.74	0.0015	WLPD	46.69	0.0001	TSAVI	49.17	0.0003
147	SRPI	25.29	0.0000	NDLI	33.75	0.0008	PSRI	46.69	0.0000	PSNDA	49.17	0.0002
148	NPCI	25.29	0.0000	NPQI	33.76	0.0004	SAVI2	46.69	0.0000	WNR	49.17	0.0002

Table S.6: Simple linear regression statistics, including root mean squared errors (RMSE, %) and coefficients of determination (r^2), for 148 spectral vegetation indices to estimate mass-basis cotton leaf chlorophyll $a + b$ (Chl $a + b$; mg g^{-1}) for data sets collected during field studies at Maricopa, Arizona, USA. Definitions and formulas for each spectral index are given in Table S.1.

	2019-2020 data			2021-2022 data			All data, 80% random split			All data, 20% random split		
Rank	Index	%RMSE	r^2	Index	%RMSE	r^2	Index	%RMSE	r^2	Index	%RMSE	r^2
1	CPSR2	19.05	0.4441	PRI2	14.45	0.3053	CPSR2	18.99	0.3628	CPSR2	18.96	0.4373
2	NDVI3	19.25	0.4320	PSSRB	14.54	0.2970	GTSR2	19.56	0.3238	GTSR2	19.39	0.4118
3	WUMSR	19.27	0.4312	PSNDB	14.61	0.2900	CRSR3	19.67	0.3158	GSUM1	19.59	0.3994
4	CRSR4	19.30	0.4294	CRSR2	14.80	0.2712	GSUM1	19.75	0.3105	WUMSR	19.69	0.3935
5	GTSR2	19.31	0.4286	CAINT	14.85	0.2661	WUMSR	19.83	0.3051	NDVI3	19.78	0.3877
6	GSUM1	19.32	0.4277	PRI3	14.96	0.2554	NDVI3	19.83	0.3048	ZTSR2	19.87	0.3822
7	WUOSAVI	19.37	0.4249	CRSR3	15.27	0.2241	PSSRB	19.90	0.2998	WUOSAVI	19.96	0.3767
8	ZTSR2	19.39	0.4236	CPSR2	15.28	0.2231	WUOSAVI	19.95	0.2965	CRSR4	20.02	0.3726
9	WLCWMRG	19.47	0.4194	WUTCARI	15.46	0.2048	CRSR2	19.96	0.2958	CRSR3	20.05	0.3707
10	WLREIP2	19.48	0.4186	WUTOR	15.49	0.2020	ZTSR2	20.03	0.2907	VSR	20.09	0.3682
11	VSR	19.56	0.4136	GSUM2	15.51	0.1999	CRSR4	20.04	0.2902	WLCWMRG	20.26	0.3579
12	DCNI	19.58	0.4128	BRI1	15.51	0.1998	PSNDB	20.08	0.2870	DCNI	20.26	0.3578
13	WLREIPG	19.60	0.4110	GTSR2	15.59	0.1917	ZTSR1	20.16	0.2816	SMNDVI	20.26	0.3578
14	MND3	19.62	0.4103	GTSR1	15.70	0.1800	VSR	20.19	0.2795	MSR2	20.35	0.3518
15	DD	19.63	0.4096	GRRGM	15.74	0.1755	WLCWMRG	20.22	0.2771	CRSR2	20.40	0.3489
16	MND1	19.70	0.4053	GSUM1	15.77	0.1722	ZTSR3	20.26	0.2743	WLREIPG	20.47	0.3440
17	DNDR	19.73	0.4037	BMLSR	15.80	0.1692	SMNDVI	20.29	0.2722	CRSR1	20.48	0.3435
18	CAR	19.79	0.3997	ZTSR1	15.82	0.1672	DCNI	20.29	0.2720	PSSRB	20.49	0.3429
19	SMNDVI	19.81	0.3987	GNDVI	15.84	0.1647	CAR	20.31	0.2707	MTCI	20.51	0.3420
20	MTCI	19.84	0.3967	ARI	15.87	0.1624	MND3	20.36	0.2671	MND3	20.54	0.3397
21	DDN	19.89	0.3936	BMSR	15.88	0.1605	WLREIP2	20.37	0.2662	DNDR	20.55	0.3389
22	MSR2	19.96	0.3895	WUMSR	15.90	0.1593	WLREIPG	20.39	0.2652	WUMCARI	20.57	0.3380
23	MMR	20.13	0.3788	NDVI3	15.96	0.1521	DD	20.40	0.2646	CAR	20.57	0.3380
24	MOR	20.17	0.3763	ZTSR2	16.00	0.1481	ZTSR4	20.42	0.2632	MND1	20.57	0.3377
25	WLREIPE	20.31	0.3679	MSR2	16.05	0.1428	MND1	20.42	0.2627	DD	20.59	0.3368
26	CRSR3	20.37	0.3643	WUOSAVI	16.07	0.1409	DNDR	20.43	0.2623	WLREIP2	20.67	0.3316
27	WUMCARI	20.42	0.3613	DCNI	16.09	0.1387	CI	20.44	0.2614	WUTOR	20.68	0.3304
28	ZTDP22	20.46	0.3584	WUMCARI	16.14	0.1330	MTCI	20.45	0.2604	PSNDB	20.77	0.3250
29	VDR	20.46	0.3584	VSR	16.14	0.1329	MSR2	20.46	0.2600	DDN	20.78	0.3242
30	CARI	20.48	0.3575	CRSR4	16.15	0.1321	WUMCARI	20.46	0.2599	WLREIPE	20.82	0.3213

Continued on next page

Table S.6 – Continued from previous page

	2019-2020 data			2021-2022 data			All data, 80% random split			All data, 20% random split		
Rank	Index	%RMSE	r^2	Index	%RMSE	r^2	Index	%RMSE	r^2	Index	%RMSE	r^2
31	MCARI	20.48	0.3575	NDWI	16.15	0.1320	CRSR1	20.52	0.2557	ZTSR3	20.94	0.3136
32	CI	20.49	0.3566	SRWI	16.16	0.1314	WLREIP	20.53	0.2553	VDR	20.96	0.3123
33	ZTDR1	20.57	0.3514	WNR	16.18	0.1291	WUTOR	20.59	0.2510	DDR1	20.97	0.3115
34	DDR1	20.58	0.3513	BRI2	16.19	0.1273	ZTSR5	20.59	0.2509	WLREIP	20.98	0.3112
35	ZTSR3	20.64	0.3474	SMNDVI	16.20	0.1263	DDN	20.60	0.2501	ZTDR1	20.99	0.3107
36	CRSR2	20.81	0.3363	TOR	16.22	0.1241	WLREIPE	20.67	0.2451	AIVI	21.11	0.3026
37	WLREIP	20.89	0.3311	ZTDP21	16.24	0.1229	VDR	20.77	0.2372	TOR	21.12	0.3017
38	ZTSR4	20.97	0.3262	WLREIP	16.25	0.1218	CAI	20.80	0.2351	CI	21.14	0.3008
39	PSSRB	20.98	0.3258	BDR	16.26	0.1198	ZTDR1	20.80	0.2350	ZTSR4	21.20	0.2967
40	TCI	21.03	0.3223	PSR	16.28	0.1184	TOR	20.84	0.2324	MMR	21.37	0.2855
41	ESUM2	21.10	0.3176	MTCI	16.28	0.1183	MMR	20.85	0.2313	WUMOR	21.38	0.2847
42	PSNDB	21.17	0.3130	WI	16.28	0.1180	DDR1	20.91	0.2269	TCARI	21.41	0.2828
43	ZTDPR1	21.18	0.3128	WLCWMRG	16.30	0.1156	MOR	20.94	0.2250	ZTSR5	21.45	0.2802
44	TOR	21.27	0.3067	ZTSR6	16.31	0.1148	AIVI	21.00	0.2203	ZTSR1	21.54	0.2738
45	WUTOR	21.27	0.3066	EGFN	16.32	0.1139	WUMOR	21.02	0.2191	MOR	21.54	0.2737
46	ZTSR1	21.30	0.3050	SIPI	16.33	0.1128	TCARI	21.03	0.2184	TCI	21.61	0.2695
47	ZTSR5	21.31	0.3042	WUMOR	16.34	0.1112	TCI	21.10	0.2134	CAI	21.78	0.2579
48	TCARI	21.38	0.2993	CRSR1	16.36	0.1096	CARI	21.14	0.2097	CARI	21.81	0.2558
49	BD	21.43	0.2962	TGI	16.37	0.1087	MCARI	21.14	0.2097	MCARI	21.81	0.2558
50	CRSR1	21.73	0.2765	AIVI	16.38	0.1074	ZTSR6	21.17	0.2079	BD	21.91	0.2487
51	AIVI	21.85	0.2682	CAR	16.38	0.1073	CAINT	21.18	0.2068	ZTDP21	21.92	0.2480
52	DREIP	21.93	0.2632	TSAVI	16.41	0.1034	ZTDP21	21.32	0.1967	CAINT	21.95	0.2461
53	ZTDPR2	21.97	0.2602	BGI1	16.42	0.1034	ZTDP22	21.38	0.1919	BRI1	22.00	0.2428
54	CRSR5	21.98	0.2594	NDVI	16.42	0.1032	BD	21.43	0.1879	BRI2	22.04	0.2397
55	WUMOR	22.06	0.2541	WDRVI2	16.42	0.1027	BDR	21.58	0.1766	DDR2	22.16	0.2313
56	ZTDP21	22.15	0.2480	WLREIPE	16.42	0.1025	CRSR5	21.63	0.1733	ZTDP22	22.19	0.2293
57	DDR2	22.18	0.2459	WDRVI	16.42	0.1024	ZTDPR1	21.65	0.1713	ZTSR6	22.28	0.2235
58	ZTSR6	22.24	0.2423	MND3	16.43	0.1017	DDR2	21.67	0.1697	CRSR5	22.29	0.2223
59	CAI	22.39	0.2315	TCARI	16.44	0.1012	GRRREM	21.84	0.1568	ZTDPR1	22.31	0.2208
60	DND	22.74	0.2074	WLREIPG	16.44	0.1007	BRI1	21.86	0.1553	BDR	22.42	0.2134
61	BMSR	22.79	0.2040	ZTDR1	16.44	0.1006	CPSR1	21.92	0.1506	BMSR	22.62	0.1995
62	TGI	22.92	0.1948	MSR	16.44	0.1005	BMSR	21.94	0.1490	DND	22.66	0.1966
63	CAINT	22.92	0.1946	DD	16.45	0.1001	DND	21.97	0.1464	CPSR1	22.73	0.1915

Continued on next page

Table S.6 – Continued from previous page

	2019-2020 data			2021-2022 data			All data, 80% random split			All data, 20% random split		
Rank	Index	%RMSE	r^2	Index	%RMSE	r^2	Index	%RMSE	r^2	Index	%RMSE	r^2
64	MND4	22.97	0.1912	VDR	16.45	0.1000	ESUM2	21.99	0.1449	BMDVI	22.78	0.1876
65	CPSR1	22.99	0.1900	DNDR	16.45	0.1000	MND4	22.10	0.1367	ESUM2	22.84	0.1839
66	GNDVI	23.00	0.1890	SAVI2	16.45	0.0995	GNDVI	22.12	0.1351	TGI	22.84	0.1838
67	MND2	23.07	0.1847	DDR2	16.45	0.0994	TGI	22.13	0.1345	MND4	22.87	0.1818
68	BDR	23.11	0.1818	PSNDA	16.45	0.0991	BMDVI	22.14	0.1332	GNDVI	22.89	0.1798
69	MCARI1	23.22	0.1739	MND1	16.46	0.0989	MND2	22.22	0.1276	MND2	23.00	0.1721
70	MTVI1	23.22	0.1739	CAI	16.48	0.0962	ZTDPR2	22.22	0.1275	DSR1	23.19	0.1584
71	DSR1	23.23	0.1729	MND4	16.48	0.0960	BRI2	22.23	0.1268	ZTDPR2	23.21	0.1566
72	TVI	23.31	0.1675	OSAVI	16.49	0.0955	DSR1	22.24	0.1254	GRRREM	23.30	0.1501
73	BMLSR	23.34	0.1654	PSSRA	16.49	0.0952	BMLSR	22.43	0.1109	BMLSR	23.37	0.1449
74	PSRI	23.48	0.1551	NLI	16.49	0.0951	WUTCARI	22.50	0.1049	WUTCARI	23.64	0.1254
75	BMDVI	23.51	0.1530	WLREIP2	16.49	0.0951	GI	22.64	0.0940	GI	23.77	0.1159
76	GI	23.54	0.1507	DDN	16.50	0.0938	DREIP	22.77	0.0837	DREIP	23.80	0.1132
77	WUTCARI	23.64	0.1436	TSAVI2	16.51	0.0935	EGFN	22.80	0.0815	SPVI	23.91	0.1052
78	MCARI2	23.71	0.1386	ZTSR5	16.51	0.0926	GRRGM	22.86	0.0763	EGFN	23.94	0.1034
79	MTVI2	23.71	0.1386	JSR	16.52	0.0924	SPVI	22.87	0.0756	BGI2	23.97	0.1007
80	CPSR3	23.77	0.1344	PD	16.52	0.0915	PRI	22.87	0.0753	NDPI	24.01	0.0976
81	GRRGM	23.81	0.1314	DDR1	16.53	0.0906	BGI2	22.87	0.0752	MCARI1	24.06	0.0942
82	GTSR1	23.84	0.1294	BD	16.54	0.0900	BGI1	22.89	0.0739	MTVI1	24.06	0.0942
83	GRRREM	23.91	0.1241	ZTSR4	16.55	0.0884	GTSR1	22.90	0.0733	GRRGM	24.06	0.0939
84	NDVI2	23.95	0.1213	DND	16.58	0.0858	MCARI1	22.91	0.0724	BGI1	24.07	0.0936
85	EGFN	23.96	0.1203	PRI	16.60	0.0830	MTVI1	22.91	0.0724	GTSR1	24.12	0.0898
86	BGI2	24.05	0.1139	NPCI	16.60	0.0830	NDVI2	22.93	0.0704	NDVI2	24.16	0.0867
87	GSUM2	24.06	0.1132	MND2	16.60	0.0829	TVI	23.00	0.0653	TVI	24.17	0.0858
88	VARI	24.08	0.1114	SRPI	16.61	0.0822	MCARI2	23.02	0.0636	SRPI	24.24	0.0807
89	DSR2	24.09	0.1110	ZTSR3	16.61	0.0819	MTVI2	23.02	0.0636	NPCI	24.25	0.0793
90	GMSR	24.11	0.1095	DSR1	16.61	0.0818	PRI3	23.03	0.0628	MCARI2	24.28	0.0775
91	BGI1	24.14	0.1068	NDPI	16.62	0.0806	DSR2	23.06	0.0603	MTVI2	24.28	0.0775
92	PSSRC	24.17	0.1051	MSI	16.64	0.0784	GMSR	23.07	0.0591	DSR2	24.34	0.0725
93	CVI	24.22	0.1009	NDNI	16.65	0.0777	VARI	23.11	0.0561	GMSR	24.37	0.0709
94	PSNDC	24.29	0.0961	MSAVI1	16.65	0.0774	GSUM2	23.11	0.0557	NDWI	24.41	0.0673
95	PRI	24.30	0.0955	CI	16.67	0.0755	CPSR3	23.13	0.0539	SRWI	24.42	0.0671
96	BRI1	24.36	0.0904	MSAVI2	16.68	0.0742	NDPI	23.18	0.0499	CPSR3	24.43	0.0657

Continued on next page

Table S.6 – Continued from previous page

Rank	2019-2020 data			2021-2022 data			All data, 80% random split			All data, 20% random split		
	Index	%RMSE	r^2	Index	%RMSE	r^2	Index	%RMSE	r^2	Index	%RMSE	r^2
97	ESUM1	24.42	0.0862	CVI	16.68	0.0740	CVI	23.20	0.0489	GSUM2	24.43	0.0656
98	RGI	24.45	0.0839	BRSR	16.73	0.0688	NDWI	23.20	0.0485	PRI3	24.45	0.0644
99	CRI700	24.59	0.0731	TCI	16.73	0.0686	SRWI	23.20	0.0482	VARI	24.45	0.0643
100	BRI2	24.77	0.0599	MCARI1	16.75	0.0669	PSRI	23.21	0.0476	PRI	24.46	0.0638
101	ARI	24.78	0.0593	MTVI1	16.75	0.0669	MSI	23.23	0.0458	MSI	24.47	0.0633
102	SPVI	24.85	0.0537	GRRREM	16.75	0.0664	RVIOPT	23.37	0.0348	PSRI	24.55	0.0567
103	NDNI	24.89	0.0509	MMR	16.76	0.0652	MSAVI2	23.37	0.0343	CVI	24.57	0.0550
104	EVI	24.91	0.0491	ESUM2	16.77	0.0638	MSAVI1	23.37	0.0342	MSAVI1	24.72	0.0439
105	MSI	25.07	0.0370	MOR	16.79	0.0615	NPQI	23.38	0.0339	RVIOPT	24.72	0.0435
106	SRWI	25.12	0.0330	ZTDP22	16.83	0.0580	RGI	23.38	0.0335	MSAVI2	24.73	0.0429
107	NDWI	25.12	0.0328	CPSR3	16.83	0.0578	SAVI	23.39	0.0330	SAVI	24.75	0.0411
108	WNR	25.18	0.0284	LCA	16.83	0.0575	RDVI	23.39	0.0327	RDVI	24.76	0.0408
109	MSR	25.19	0.0279	TVI	16.83	0.0573	EVI2	23.41	0.0317	NPQI	24.76	0.0404
110	NPQI	25.20	0.0271	BMDVI	16.85	0.0557	NLI	23.41	0.0311	EVI2	24.77	0.0399
111	WDRVI	25.20	0.0265	SAVI	16.85	0.0550	PSSRC	23.42	0.0305	PSR	24.77	0.0397
112	WDRVI2	25.21	0.0261	ZTDPR1	16.86	0.0542	TSAVI2	23.42	0.0301	WI	24.77	0.0395
113	TSAVI	25.23	0.0246	CARI	16.88	0.0523	OSAVI	23.44	0.0290	NLI	24.78	0.0387
114	NDVI	25.23	0.0246	MCARI	16.88	0.0523	SAVI2	23.48	0.0256	TSAVI2	24.80	0.0374
115	SAVI2	25.24	0.0237	RVIOPT	16.90	0.0493	PSNDC	23.49	0.0245	OSAVI	24.82	0.0363
116	NLI	25.25	0.0227	EVI2	16.92	0.0469	GEMI	23.54	0.0206	SIPI	24.83	0.0349
117	NDPI	25.26	0.0221	RDVI	16.92	0.0469	SRPI	23.54	0.0201	SAVI2	24.84	0.0347
118	JSR	25.27	0.0217	BGI2	16.94	0.0455	WNR	23.55	0.0197	RGI	24.84	0.0341
119	PSSRA	25.28	0.0205	PSNDC	16.96	0.0426	NPCI	23.55	0.0193	ESUM1	24.89	0.0303
120	WI	25.29	0.0201	PSSRC	16.98	0.0406	MSR	23.56	0.0186	WNR	24.90	0.0297
121	PSR	25.29	0.0201	ZTDPR2	17.01	0.0367	TSAVI	23.57	0.0182	PSSRC	24.94	0.0268
122	OSAVI	25.30	0.0190	CRSR5	17.05	0.0325	WDRVI	23.57	0.0180	MSR	24.94	0.0266
123	TSAVI2	25.31	0.0181	CPSR1	17.06	0.0316	WDRVI2	23.57	0.0178	GEMI	24.94	0.0264
124	PSNDA	25.32	0.0178	PSRI	17.07	0.0307	ESUM1	23.57	0.0176	WDRVI	24.96	0.0252
125	PRI2	25.37	0.0139	DREIP	17.08	0.0290	NDVI	23.58	0.0171	PSSRA	24.96	0.0251
126	SIPI	25.38	0.0126	ESUM1	17.09	0.0286	PSSRA	23.60	0.0152	WDRVI2	24.96	0.0247
127	MSAVI1	25.40	0.0116	NPQI	17.09	0.0277	PVI	23.60	0.0151	TSAVI	24.97	0.0244
128	BRSR	25.41	0.0109	NDLI	17.10	0.0273	WDVI	23.60	0.0151	NDVI	24.98	0.0231
129	MSAVI2	25.41	0.0107	EVI	17.12	0.0242	PSR	23.61	0.0148	JSR	25.00	0.0223

Continued on next page

Table S.6 – Continued from previous page

	2019-2020 data			2021-2022 data			All data, 80% random split			All data, 20% random split		
Rank	Index	%RMSE	r^2	Index	%RMSE	r^2	Index	%RMSE	r^2	Index	%RMSE	r^2
130	LCA	25.43	0.0090	GEMI	17.17	0.0189	WI	23.61	0.0146	PSNDC	25.01	0.0214
131	ZTSUM	25.45	0.0073	SPVI	17.19	0.0172	JSR	23.61	0.0143	PSNDA	25.01	0.0213
132	WLPD	25.47	0.0062	DSR2	17.23	0.0122	PSNDA	23.62	0.0139	PVI	25.04	0.0188
133	RVIOPT	25.47	0.0062	GI	17.23	0.0120	NDNI	23.63	0.0128	WDVI	25.04	0.0188
134	CRI500	25.47	0.0060	GMSR	17.24	0.0115	DVI	23.63	0.0126	DVI	25.08	0.0153
135	SAVI	25.48	0.0052	NDVI2	17.24	0.0108	NDLI	23.65	0.0109	NDNI	25.12	0.0127
136	RDVI	25.49	0.0044	DPI	17.28	0.0068	FSUM	23.66	0.0102	FSUM	25.12	0.0125
137	EVI2	25.50	0.0035	CRI500	17.30	0.0043	ARI	23.68	0.0088	PRI2	25.14	0.0113
138	FSUM	25.50	0.0032	VARI	17.31	0.0031	PRI2	23.72	0.0055	EVI	25.14	0.0110
139	DPI	25.51	0.0029	MCARI2	17.31	0.0030	ZTSUM	23.72	0.0054	BRSR	25.16	0.0092
140	PRI3	25.52	0.0022	MTVI2	17.31	0.0030	BRSR	23.72	0.0053	NDLI	25.19	0.0070
141	DVI	25.53	0.0015	RGI	17.31	0.0027	CRI700	23.73	0.0044	ZTSUM	25.21	0.0057
142	PVI	25.54	0.0007	PVI	17.31	0.0027	EVI	23.73	0.0043	ARI	25.21	0.0053
143	WDVI	25.54	0.0007	WDVI	17.31	0.0027	CRI500	23.74	0.0038	CRI700	25.21	0.0053
144	NDLI	25.54	0.0003	DVI	17.33	0.0006	SIPI	23.74	0.0036	PD	25.25	0.0026
145	PD	25.54	0.0003	WLPD	17.33	0.0003	DPI	23.76	0.0019	LCA	25.25	0.0023
146	GEMI	25.54	0.0001	CRI700	17.33	0.0003	WLPD	23.77	0.0014	WLPD	25.26	0.0012
147	SRPI	25.54	0.0000	FSUM	17.33	0.0002	LCA	23.78	0.0006	DPI	25.27	0.0010
148	NPCI	25.54	0.0000	ZTSUM	17.34	0.0000	PD	23.78	0.0000	CRI500	25.28	0.0002

Table S.7: Simple linear regression statistics, including root mean squared errors (RMSE, %) and coefficients of determination (r^2), for 148 spectral vegetation indices to estimate mass-basis cotton leaf chlorophyll *a* (Chl *a*; mg g^{-1}) for data sets collected during field studies at Maricopa, Arizona, USA. Definitions and formulas for each spectral index are given in Table S.1.

	2019-2020 data			2021-2022 data			All data, 80% random split			All data, 20% random split		
Rank	Index	%RMSE	r^2	Index	%RMSE	r^2	Index	%RMSE	r^2	Index	%RMSE	r^2
1	CPSR2	19.96	0.4084	PSSRB	14.34	0.2358	CPSR2	19.15	0.3039	CPSR2	19.76	0.3638
2	NDVI3	20.01	0.4056	PSNDB	14.45	0.2245	PSSRB	19.51	0.2780	GTSR2	20.27	0.3306
3	WUMSR	20.03	0.4046	CAINT	14.53	0.2152	CRSR3	19.52	0.2771	GSUM1	20.43	0.3203
4	GTSR2	20.03	0.4043	CRSR2	14.57	0.2112	CRSR2	19.64	0.2679	WUMSR	20.47	0.3176
5	GSUM1	20.07	0.4022	PRI2	14.71	0.1955	PSNDB	19.67	0.2656	NDVI3	20.47	0.3172
6	CRSR4	20.07	0.4020	WUTCARI	14.76	0.1900	GTSR2	19.71	0.2630	CRSR3	20.48	0.3167
7	WUOSAVI	20.12	0.3991	CRSR3	14.82	0.1833	NDVI3	19.87	0.2506	CRSR4	20.62	0.3074
8	ZTSR2	20.16	0.3969	CPSR2	14.85	0.1800	GSUM1	19.87	0.2504	ZTSR2	20.62	0.3070
9	WLCWMRG	20.24	0.3918	GSUM2	14.89	0.1759	WUMSR	19.92	0.2471	CRSR2	20.64	0.3059
10	VSR	20.30	0.3880	WUTOR	14.89	0.1757	WUOSAVI	20.01	0.2400	WUOSAVI	20.68	0.3034
11	WLREIP2	20.38	0.3834	BRI1	14.97	0.1674	CRSR4	20.01	0.2399	PSSRB	20.69	0.3023
12	DD	20.43	0.3806	PRI3	14.98	0.1666	ZTSR1	20.08	0.2348	VSR	20.73	0.2998
13	MND3	20.43	0.3801	GTSR2	15.02	0.1620	ZTSR2	20.10	0.2334	WUTOR	20.81	0.2944
14	DCNI	20.45	0.3789	GTSR1	15.03	0.1600	CAR	20.16	0.2288	CAR	20.90	0.2885
15	WLREIPG	20.47	0.3780	GRRGM	15.06	0.1566	VSR	20.18	0.2272	PSNDB	20.93	0.2865
16	MND1	20.53	0.3745	BMLSR	15.14	0.1485	WUTOR	20.20	0.2257	SMNDVI	21.00	0.2816
17	DNDR	20.57	0.3718	GSUM1	15.15	0.1467	ZTSR3	20.32	0.2166	WLCWMRG	21.05	0.2781
18	SMNDVI	20.59	0.3704	GNDVI	15.18	0.1434	MND3	20.35	0.2139	WLREIPG	21.09	0.2753
19	MTCI	20.65	0.3670	BMSR	15.22	0.1391	WLREIP2	20.37	0.2130	DNDR	21.10	0.2750
20	DDN	20.67	0.3655	WUMSR	15.24	0.1364	WLCWMRG	20.37	0.2130	MND3	21.11	0.2741
21	MSR2	20.74	0.3614	ZTSR2	15.30	0.1301	SMNDVI	20.37	0.2124	MND1	21.12	0.2734
22	CAR	20.85	0.3547	NDVI3	15.31	0.1292	DNDR	20.39	0.2112	DCNI	21.16	0.2706
23	CRSR3	20.91	0.3507	MSR2	15.34	0.1255	MND1	20.39	0.2110	WLREIP2	21.17	0.2697
24	WUMCARI	20.98	0.3469	WUOSAVI	15.39	0.1194	ZTSR4	20.39	0.2109	DD	21.18	0.2695
25	MMR	21.03	0.3434	VSR	15.40	0.1188	DD	20.40	0.2100	MSR2	21.23	0.2660
26	WLREIPE	21.04	0.3427	ARI	15.41	0.1177	WLREIPG	20.41	0.2097	MTCI	21.25	0.2645
27	ZTDP22	21.13	0.3375	SIPI	15.43	0.1156	CI	20.42	0.2085	TOR	21.26	0.2635
28	MOR	21.16	0.3355	DCNI	15.43	0.1151	TOR	20.49	0.2036	DDN	21.32	0.2594
29	VDR	21.17	0.3349	WUMCARI	15.43	0.1147	ZTSR5	20.50	0.2024	CRSR1	21.33	0.2589
30	ZTDR1	21.26	0.3289	CRSR4	15.44	0.1143	DCNI	20.53	0.2006	WLREIPE	21.34	0.2582

Continued on next page

Table S.7 – Continued from previous page

	2019-2020 data			2021-2022 data			All data, 80% random split			All data, 20% random split		
Rank	Index	%RMSE	r^2	Index	%RMSE	r^2	Index	%RMSE	r^2	Index	%RMSE	r^2
31	CI	21.29	0.3271	BRI2	15.44	0.1137	DDN	20.56	0.1982	VDR	21.39	0.2545
32	MCARI	21.45	0.3173	SMNDVI	15.49	0.1079	MTCI	20.56	0.1982	ZTDR1	21.40	0.2541
33	CARI	21.45	0.3173	DDR2	15.50	0.1068	CAINT	20.57	0.1967	WUMCARI	21.41	0.2532
34	DDR1	21.52	0.3125	ZTDP21	15.51	0.1065	WUMCARI	20.60	0.1948	AIVI	21.48	0.2483
35	PSSRB	21.61	0.3068	TOR	15.53	0.1041	WLREIPE	20.60	0.1944	TCARI	21.49	0.2480
36	ZTSR3	21.64	0.3048	WLREIP	15.53	0.1041	MSR2	20.63	0.1928	ZTSR3	21.51	0.2464
37	WLREIP	21.66	0.3034	BDR	15.53	0.1037	WLREIP	20.63	0.1926	CI	21.61	0.2394
38	CRSR2	21.70	0.3008	EGFN	15.53	0.1037	VDR	20.64	0.1914	MMR	21.63	0.2380
39	ZTDPR1	21.72	0.2996	MTCI	15.54	0.1028	TCARI	20.65	0.1911	ZTSR4	21.66	0.2360
40	PSNDB	21.77	0.2962	WNR	15.57	0.0988	MOR	20.66	0.1904	DDR1	21.67	0.2351
41	ESUM2	21.86	0.2906	TGI	15.58	0.0974	MMR	20.66	0.1900	TCI	21.67	0.2349
42	ZTSR4	21.96	0.2844	WLCWMRG	15.59	0.0968	ZTDR1	20.66	0.1900	MOR	21.68	0.2346
43	TCI	22.07	0.2767	CRSR1	15.60	0.0957	CRSR1	20.71	0.1860	WLREIP	21.70	0.2330
44	BD	22.15	0.2719	AIVI	15.60	0.0956	TCI	20.73	0.1847	BD	21.78	0.2273
45	ZTSR1	22.19	0.2687	WUMOR	15.60	0.0955	AIVI	20.79	0.1799	ZTSR1	21.82	0.2246
46	WUTOR	22.24	0.2660	ZTDR1	15.60	0.0951	ZTDP22	20.81	0.1786	ZTSR5	21.83	0.2235
47	TOR	22.28	0.2631	ZTSR1	15.61	0.0939	CARI	20.82	0.1775	CARI	21.88	0.2199
48	ZTSR5	22.29	0.2627	BGI1	15.63	0.0926	MCARI	20.82	0.1775	MCARI	21.88	0.2199
49	TCARI	22.40	0.2551	VDR	15.63	0.0918	ZTSR6	20.87	0.1736	CAINT	21.88	0.2198
50	ZTDPR2	22.45	0.2521	WLREIPE	15.64	0.0911	ZTDPR1	20.92	0.1693	ZTDP22	21.97	0.2134
51	WUMOR	22.46	0.2514	CAR	15.64	0.0908	BD	20.95	0.1672	ZTDPR1	21.98	0.2131
52	DREIP	22.59	0.2428	BD	15.67	0.0878	DDR1	20.98	0.1651	WUMOR	22.10	0.2043
53	CRSR1	22.64	0.2390	WLREIPG	15.67	0.0872	ESUM2	21.06	0.1580	ESUM2	22.18	0.1988
54	DDR2	22.66	0.2380	DNDR	15.68	0.0868	WUMOR	21.07	0.1575	DDR2	22.37	0.1850
55	CRSR5	22.75	0.2315	DD	15.68	0.0864	CAI	21.11	0.1546	BMSR	22.37	0.1845
56	ZTDP21	22.86	0.2242	TCARI	15.68	0.0864	BMSR	21.28	0.1411	ZTSR6	22.39	0.1831
57	AIVI	22.86	0.2240	MND3	15.68	0.0863	ZTDPR2	21.32	0.1372	DND	22.46	0.1779
58	CAI	23.13	0.2058	MND1	15.69	0.0854	DND	21.36	0.1339	BRI1	22.53	0.1731
59	ZTSR6	23.16	0.2036	TSAVI	15.69	0.0847	CRSR5	21.39	0.1316	CRSR5	22.54	0.1725
60	CAINT	23.22	0.1996	PSNDA	15.69	0.0847	DDR2	21.41	0.1303	CAI	22.55	0.1712
61	DND	23.65	0.1695	NDVI	15.69	0.0846	GNDVI	21.42	0.1292	TGI	22.57	0.1703
62	BMSR	23.67	0.1680	NPCI	15.70	0.0837	ZTDP21	21.44	0.1281	GNDVI	22.60	0.1680
63	CPSR1	23.71	0.1658	SRPI	15.70	0.0834	TGI	21.47	0.1254	BRI2	22.62	0.1662

Continued on next page

Table S.7 – Continued from previous page

Rank	2019-2020 data			2021-2022 data			All data, 80% random split			All data, 20% random split		
	Index	%RMSE	r^2	Index	%RMSE	r^2	Index	%RMSE	r^2	Index	%RMSE	r^2
64	BDR	23.73	0.1642	WDRVI2	15.70	0.0834	MND4	21.47	0.1250	ZTDPR2	22.63	0.1658
65	TGI	23.82	0.1574	WDRVI	15.71	0.0829	MND2	21.56	0.1177	ZTDP21	22.63	0.1657
66	GNDVI	23.86	0.1546	DDN	15.71	0.0829	DSR1	21.62	0.1130	MND4	22.65	0.1642
67	MND4	23.87	0.1545	MND4	15.71	0.0826	BDR	21.66	0.1097	MND2	22.75	0.1566
68	MND2	23.95	0.1487	DDR1	15.71	0.0824	BMDVI	21.67	0.1090	BMDVI	22.86	0.1488
69	MCARI1	24.06	0.1405	WLREIP2	15.72	0.0811	BMLSR	21.67	0.1085	DSR1	22.93	0.1438
70	MTVI1	24.06	0.1405	MSR	15.73	0.0807	DREIP	21.68	0.1080	DREIP	22.95	0.1423
71	DSR1	24.11	0.1374	SAVI2	15.73	0.0801	GRRREM	21.70	0.1065	BMLSR	22.99	0.1387
72	TVI	24.13	0.1356	DND	15.73	0.0799	WUTCARI	21.75	0.1024	BDR	23.03	0.1356
73	BMLSR	24.16	0.1336	PSSRA	15.74	0.0790	BRI1	21.75	0.1020	CPSR1	23.05	0.1342
74	PSRI	24.17	0.1326	OSAVI	15.75	0.0782	CPSR1	21.77	0.1004	WUTCARI	23.19	0.1237
75	CPSR3	24.26	0.1264	NLI	15.76	0.0773	GI	21.99	0.0825	MCARI1	23.41	0.1076
76	BMDVI	24.29	0.1241	TSAVI2	15.76	0.0765	MCARI1	22.00	0.0812	MTVI1	23.41	0.1076
77	GI	24.38	0.1174	JSR	15.78	0.0752	MTVI1	22.00	0.0812	GI	23.47	0.1028
78	WUTCARI	24.42	0.1145	NDPI	15.78	0.0741	BRI2	22.01	0.0805	TVI	23.50	0.1005
79	GRRREM	24.52	0.1076	MND2	15.79	0.0732	GRRGM	22.04	0.0779	GRRREM	23.57	0.0952
80	MCARI2	24.54	0.1058	DSR1	15.80	0.0725	GTSR1	22.07	0.0756	GRRGM	23.58	0.0943
81	MTVI2	24.54	0.1058	ZTSR6	15.82	0.0704	EGFN	22.08	0.0750	EGFN	23.58	0.0941
82	GRRGM	24.57	0.1041	CVI	15.84	0.0673	TVI	22.09	0.0744	GTSR1	23.62	0.0910
83	PSSRC	24.57	0.1040	PSR	15.87	0.0636	CPSR3	22.14	0.0699	BGI2	23.64	0.0897
84	GTSR1	24.59	0.1024	WI	15.88	0.0632	BGI2	22.18	0.0661	BGI1	23.72	0.0836
85	PSNDC	24.67	0.0967	MSAVI1	15.88	0.0623	BGI1	22.20	0.0648	NDVI2	23.77	0.0791
86	NDVI2	24.73	0.0918	ESUM2	15.89	0.0621	NDVI2	22.21	0.0639	CPSR3	23.78	0.0787
87	EGFN	24.75	0.0907	ZTSR5	15.89	0.0621	PRI	22.22	0.0632	MCARI2	23.82	0.0759
88	GSUM2	24.78	0.0882	TCI	15.89	0.0612	GSUM2	22.26	0.0596	MTVI2	23.82	0.0759
89	BGI2	24.83	0.0848	MCARI1	15.90	0.0605	MCARI2	22.26	0.0595	GSUM2	23.90	0.0691
90	VARI	24.84	0.0840	MTVI1	15.90	0.0605	MTVI2	22.26	0.0595	NDPI	23.90	0.0691
91	DSR2	24.85	0.0833	ZTSR4	15.90	0.0600	DSR2	22.30	0.0564	DSR2	23.92	0.0679
92	GMSR	24.87	0.0820	MSAVI2	15.90	0.0600	GMSR	22.31	0.0553	GMSR	23.94	0.0665
93	BGI1	24.92	0.0782	ZTDP22	15.92	0.0586	VARI	22.33	0.0534	SPVI	23.97	0.0643
94	CVI	24.94	0.0766	ZTDPR1	15.92	0.0581	PSRI	22.34	0.0534	PSRI	23.99	0.0625
95	ESUM1	24.94	0.0763	MMR	15.92	0.0579	CVI	22.37	0.0504	VARI	24.00	0.0618
96	BRI1	25.00	0.0720	ZTSR3	15.93	0.0566	SPVI	22.37	0.0502	CVI	24.06	0.0566

Continued on next page

Table S.7 – Continued from previous page

Rank	2019-2020 data			2021-2022 data			All data, 80% random split			All data, 20% random split		
	Index	%RMSE	r^2	Index	%RMSE	r^2	Index	%RMSE	r^2	Index	%RMSE	r^2
97	PRI	25.01	0.0712	GRRREM	15.94	0.0563	PSSRC	22.42	0.0465	PRI	24.10	0.0538
98	CRI700	25.13	0.0622	CI	15.94	0.0562	PSNDC	22.49	0.0402	SRPI	24.14	0.0506
99	RGI	25.15	0.0607	PRI	15.94	0.0561	PRI3	22.52	0.0377	NPCI	24.15	0.0499
100	BRI2	25.18	0.0585	MOR	15.94	0.0560	RGI	22.56	0.0345	ESUM1	24.18	0.0473
101	NDNI	25.24	0.0544	BRSR	15.95	0.0545	NLI	22.56	0.0338	PSSRC	24.28	0.0399
102	SPVI	25.38	0.0439	TVI	15.96	0.0530	NDPI	22.59	0.0319	NDWI	24.28	0.0398
103	ARI	25.41	0.0419	NDWI	15.99	0.0496	TSAVI2	22.60	0.0312	SRWI	24.28	0.0398
104	EVI	25.41	0.0417	SRWI	16.00	0.0491	OSAVI	22.60	0.0310	PRI3	24.30	0.0379
105	MSR	25.54	0.0320	CARI	16.00	0.0488	SAVI2	22.61	0.0302	RGI	24.33	0.0357
106	WNR	25.55	0.0312	MCARI	16.00	0.0488	MSAVI1	22.62	0.0290	NLI	24.34	0.0350
107	WDRVI	25.56	0.0306	BMDVI	16.00	0.0487	MSAVI2	22.63	0.0286	MSI	24.35	0.0343
108	WDRVI2	25.56	0.0302	ZTDPR2	16.02	0.0457	NDWI	22.64	0.0277	SAVI2	24.35	0.0343
109	MSI	25.57	0.0296	SAVI	16.03	0.0449	SRWI	22.64	0.0276	PSNDC	24.36	0.0334
110	TSAVI	25.58	0.0286	BGI2	16.05	0.0429	ESUM1	22.64	0.0271	WNR	24.36	0.0333
111	NDVI	25.58	0.0285	RVIOPT	16.07	0.0408	WNR	22.65	0.0268	OSAVI	24.37	0.0321
112	SAVI2	25.59	0.0278	CPSR3	16.07	0.0402	MSR	22.65	0.0267	TSAVI2	24.38	0.0321
113	NLI	25.60	0.0273	PSRI	16.08	0.0396	WDRVI	22.65	0.0261	MSR	24.38	0.0319
114	SRWI	25.61	0.0266	RDVI	16.09	0.0383	TSAVI	22.66	0.0260	MSAVI1	24.39	0.0308
115	NDWI	25.61	0.0263	EVI2	16.09	0.0382	WDRVI2	22.66	0.0259	PSR	24.39	0.0305
116	JSR	25.62	0.0255	DREIP	16.11	0.0359	NDVI	22.67	0.0251	WDRVI	24.40	0.0304
117	PSSRA	25.64	0.0242	PD	16.12	0.0341	RVIOPT	22.67	0.0250	WI	24.40	0.0304
118	OSAVI	25.66	0.0230	CRSR5	16.16	0.0294	SAVI	22.68	0.0236	WDRVI2	24.40	0.0299
119	TSAVI2	25.67	0.0220	PSNDC	16.17	0.0287	MSI	22.69	0.0228	PSSRA	24.41	0.0295
120	PSNDA	25.68	0.0213	CAI	16.18	0.0272	RDVI	22.70	0.0224	MSAVI2	24.41	0.0294
121	NDPI	25.74	0.0162	PSSRC	16.19	0.0259	NDNI	22.70	0.0224	TSAVI	24.42	0.0289
122	WI	25.76	0.0149	CPSR1	16.19	0.0255	PSSRA	22.70	0.0221	NDVI	24.42	0.0282
123	PSR	25.76	0.0149	EVI	16.20	0.0243	JSR	22.71	0.0216	JSR	24.43	0.0275
124	MSAVI1	25.77	0.0145	ESUM1	16.23	0.0209	EVI2	22.71	0.0211	PSNDA	24.46	0.0257
125	BRSR	25.77	0.0144	NPQI	16.26	0.0173	PSNDA	22.71	0.0210	RVIOPT	24.46	0.0254
126	MSAVI2	25.78	0.0135	NDNI	16.26	0.0170	NPQI	22.76	0.0166	SAVI	24.48	0.0238
127	NPQI	25.79	0.0129	MSI	16.27	0.0159	ARI	22.80	0.0135	RDVI	24.50	0.0225
128	LCA	25.81	0.0113	GEMI	16.28	0.0151	CRI700	22.80	0.0132	NPQI	24.50	0.0221
129	SIPI	25.82	0.0101	SPVI	16.28	0.0149	PSR	22.82	0.0116	EVI2	24.51	0.0214

Continued on next page

Table S.7 – Continued from previous page

	2019-2020 data			2021-2022 data			All data, 80% random split			All data, 20% random split		
Rank	Index	%RMSE	r^2	Index	%RMSE	r^2	Index	%RMSE	r^2	Index	%RMSE	r^2
130	RVIOPT	25.84	0.0085	DPI	16.29	0.0135	WI	22.82	0.0115	SIPI	24.52	0.0207
131	SAVI	25.86	0.0072	LCA	16.29	0.0133	BRSR	22.83	0.0110	NDNI	24.54	0.0189
132	PRI2	25.87	0.0067	GI	16.30	0.0127	GEMI	22.85	0.0092	CRI700	24.60	0.0143
133	WLPD	25.87	0.0067	DSR2	16.31	0.0114	SRPI	22.86	0.0086	BRSR	24.60	0.0138
134	RDVI	25.87	0.0062	GMSR	16.31	0.0110	NPCI	22.86	0.0082	ARI	24.65	0.0099
135	DPI	25.88	0.0058	NDVI2	16.31	0.0108	PVI	22.90	0.0051	GEMI	24.67	0.0088
136	ZTSUM	25.88	0.0057	NDLI	16.35	0.0066	WDVI	22.90	0.0051	PVI	24.72	0.0043
137	EVI2	25.89	0.0050	MCARI2	16.37	0.0037	NDLI	22.91	0.0037	WDVI	24.72	0.0043
138	PRI3	25.90	0.0043	MTVI2	16.37	0.0037	DVI	22.92	0.0035	PRI2	24.73	0.0040
139	FSUM	25.92	0.0025	RGI	16.38	0.0034	WLPD	22.92	0.0032	DVI	24.74	0.0026
140	CRI500	25.94	0.0012	VARI	16.38	0.0028	FSUM	22.93	0.0022	PD	24.75	0.0018
141	DVI	25.94	0.0011	CRI500	16.39	0.0022	PRI2	22.94	0.0014	FSUM	24.76	0.0016
142	PVI	25.95	0.0004	PVI	16.39	0.0022	SIPI	22.94	0.0010	NDLI	24.76	0.0012
143	WDVI	25.95	0.0004	WDVI	16.39	0.0022	LCA	22.95	0.0010	EVI	24.77	0.0006
144	SRPI	25.95	0.0002	CRI700	16.40	0.0005	CRI500	22.95	0.0010	DPI	24.77	0.0005
145	NPCI	25.95	0.0002	DVI	16.40	0.0005	ZTSUM	22.95	0.0005	WLPD	24.77	0.0004
146	PD	25.95	0.0001	FSUM	16.40	0.0002	DPI	22.95	0.0005	LCA	24.77	0.0003
147	NDLI	25.95	0.0000	ZTSUM	16.40	0.0001	EVI	22.96	0.0000	CRI500	24.77	0.0001
148	GEMI	25.95	0.0000	WLPD	16.40	0.0000	PD	22.96	0.0000	ZTSUM	24.77	0.0001

Table S.8: Simple linear regression statistics, including root mean squared errors (RMSE, %) and coefficients of determination (r^2), for 148 spectral vegetation indices to estimate mass-basis cotton leaf chlorophyll *b* (Chl *b*; mg g^{-1}) for data sets collected during field studies at Maricopa, Arizona, USA. Definitions and formulas for each spectral index are given in Table S.1.

	2019-2020 data			2021-2022 data			All data, 80% random split			All data, 20% random split		
Rank	Index	%RMSE	r^2	Index	%RMSE	r^2	Index	%RMSE	r^2	Index	%RMSE	r^2
1	CAR	22.65	0.4392	PRI2	21.30	0.4113	CAI	31.59	0.3454	DCNI	28.76	0.4391
2	CPSR2	23.03	0.4199	PRI3	22.64	0.3352	DCNI	32.48	0.3081	GTSR2	28.83	0.4364
3	TCI	23.28	0.4073	NDWI	22.86	0.3218	CPSR2	32.51	0.3069	MSR2	28.95	0.4317
4	ZTSR3	23.31	0.4059	SRWI	22.87	0.3215	CRSR1	32.64	0.3011	GSUM1	29.11	0.4255
5	MOR	23.36	0.4032	MSI	23.46	0.2859	GTSR2	32.89	0.2903	CPSR2	29.13	0.4246
6	ZTSR4	23.57	0.3924	CAI	23.49	0.2841	MSR2	32.90	0.2900	CRSR1	29.18	0.4228
7	CARI	23.60	0.3911	PSNDB	23.54	0.2813	ZTDP21	32.91	0.2897	WUMCARI	29.18	0.4225
8	MCARI	23.60	0.3909	NDNI	23.66	0.2736	BDR	33.04	0.2838	WUMSR	29.42	0.4131
9	TCARI	23.61	0.3906	PSSRB	23.68	0.2727	GSUM1	33.06	0.2829	WLCWMRG	29.44	0.4123
10	WLREIP2	23.62	0.3900	ZTSR1	23.73	0.2698	WLCWMRG	33.09	0.2818	ZTSR2	29.60	0.4061
11	TOR	23.62	0.3898	CRSR2	23.93	0.2568	WUMCARI	33.15	0.2794	CAI	29.71	0.4016
12	DCNI	23.69	0.3866	CAINT	24.25	0.2371	WLREIP	33.25	0.2748	SMNDVI	29.74	0.4001
13	ZTSR5	23.81	0.3804	PD	24.42	0.2263	WUMSR	33.26	0.2745	MTCI	29.77	0.3992
14	AIVI	23.85	0.3779	WI	24.81	0.2015	MTCI	33.28	0.2737	WUOSAVI	29.82	0.3971
15	WLREIPG	23.88	0.3768	PSR	24.81	0.2014	ZTSR3	33.36	0.2701	WLREIP	29.90	0.3937
16	WUTOR	23.93	0.3739	LCA	24.90	0.1956	ZTSR2	33.40	0.2682	ZTDP21	29.91	0.3936
17	MMR	23.93	0.3739	CPSR2	24.92	0.1943	SMNDVI	33.41	0.2677	NDVI3	29.91	0.3932
18	DDR1	24.03	0.3689	CRSR3	25.03	0.1875	WUOSAVI	33.47	0.2651	WUMOR	30.00	0.3896
19	DNDR	24.05	0.3675	ARI	25.34	0.1669	NDVI3	33.53	0.2627	DDR1	30.18	0.3825
20	MND3	24.11	0.3644	ZTSR6	25.41	0.1625	DDR1	33.56	0.2613	VSR	30.27	0.3787
21	MND1	24.12	0.3641	BRI1	25.41	0.1623	WUMOR	33.69	0.2555	BDR	30.46	0.3710
22	CRSR4	24.16	0.3618	WUTOR	25.60	0.1498	ZTSR1	33.73	0.2538	CRSR4	30.48	0.3700
23	WUMSR	24.19	0.3602	GTSR2	25.61	0.1489	ZTSR4	33.79	0.2512	WLREIPG	30.50	0.3694
24	ZTSR1	24.19	0.3600	GSUM2	25.68	0.1443	WLREIPG	33.79	0.2510	DD	30.68	0.3618
25	CRSR2	24.20	0.3599	WUTCARI	25.79	0.1374	VSR	33.83	0.2493	MND3	30.78	0.3577
26	NDVI3	24.21	0.3593	GSUM1	25.88	0.1311	DD	33.85	0.2483	ZTSR3	30.89	0.3531
27	DD	24.22	0.3587	WNR	25.90	0.1296	CRSR4	33.91	0.2459	MND1	30.89	0.3529
28	WLCWMRG	24.25	0.3573	GTSR1	25.96	0.1258	MND3	33.93	0.2447	DNDR	30.91	0.3521
29	ZTSR2	24.25	0.3571	GRRGM	26.03	0.1212	CI	33.93	0.2447	BRI2	30.96	0.3501
30	MTCI	24.26	0.3568	BMLSR	26.03	0.1209	WLREIP2	33.98	0.2426	DDN	31.00	0.3485

Continued on next page

Table S.8 – Continued from previous page

Rank	2019-2020 data			2021-2022 data			All data, 80% random split			All data, 20% random split		
	Index	%RMSE	r^2	Index	%RMSE	r^2	Index	%RMSE	r^2	Index	%RMSE	r^2
31	GSUM1	24.31	0.3538	GNDVI	26.05	0.1197	MND1	34.06	0.2391	WLREIPE	31.12	0.3432
32	WUOSAVI	24.36	0.3514	WUMSR	26.06	0.1193	ZTSR5	34.11	0.2368	WLREIP2	31.17	0.3411
33	SMNDVI	24.37	0.3505	BMSR	26.08	0.1176	DNDR	34.11	0.2367	BRI1	31.34	0.3342
34	GTSR2	24.41	0.3486	NDVI3	26.11	0.1155	DDN	34.16	0.2347	CRSR3	31.40	0.3317
35	CRSR1	24.44	0.3472	DCNI	26.15	0.1129	WLREIPE	34.21	0.2325	CI	31.45	0.3293
36	VSR	24.44	0.3469	ZTSR5	26.17	0.1113	BRI1	34.22	0.2319	ZTSR4	31.54	0.3256
37	MSR2	24.47	0.3456	WUOSAVI	26.24	0.1068	CRSR3	34.30	0.2284	VDR	31.62	0.3219
38	DDN	24.49	0.3442	ZTSR2	26.27	0.1048	CAR	34.50	0.2193	ZTDR1	31.73	0.3173
39	ZTSR6	24.57	0.3403	ZTSR4	26.29	0.1037	VDR	34.59	0.2152	CAR	31.98	0.3067
40	CI	24.58	0.3395	PRI	26.29	0.1032	ZTDR1	34.64	0.2131	ZTSR5	31.99	0.3060
41	WLREIPE	24.93	0.3207	MSR2	26.31	0.1018	GRRREM	34.67	0.2114	AIVI	32.04	0.3038
42	DND	25.02	0.3159	TOR	26.38	0.0974	CPSR1	34.78	0.2068	CRSR2	32.36	0.2899
43	WLREIP	25.09	0.3121	WUMCARI	26.38	0.0973	CRSR2	34.85	0.2033	ZTSR1	32.59	0.2799
44	TGI	25.11	0.3109	CRSR4	26.40	0.0956	AIVI	34.92	0.2001	PSSRB	32.60	0.2792
45	ESUM2	25.14	0.3091	ZTDP21	26.42	0.0944	MMR	35.01	0.1962	CPSR1	32.64	0.2778
46	VDR	25.18	0.3066	NDLI	26.42	0.0941	PSSRB	35.15	0.1895	MMR	32.67	0.2761
47	MND4	25.23	0.3040	SMNDVI	26.43	0.0941	BRI2	35.17	0.1888	GRRREM	32.89	0.2664
48	BMSR	25.25	0.3029	BDR	26.44	0.0930	ZTSR6	35.25	0.1848	PSNDB	32.92	0.2651
49	ZTDR1	25.32	0.2992	WLREIP	26.44	0.0929	PSNDB	35.28	0.1837	WUTOR	32.96	0.2636
50	MND2	25.36	0.2971	ZTSR3	26.45	0.0924	DDR2	35.39	0.1784	CRSR5	33.12	0.2565
51	ZTDP22	25.43	0.2929	WDRVI	26.45	0.0924	CRSR5	35.40	0.1781	TOR	33.13	0.2557
52	DSR1	25.48	0.2904	WDRVI2	26.45	0.0922	MOR	35.41	0.1774	DDR2	33.28	0.2488
53	GNDVI	25.52	0.2881	MSR	26.46	0.0920	WUTOR	35.45	0.1755	MOR	33.36	0.2452
54	BD	25.58	0.2847	TSAVI	26.47	0.0911	TOR	35.50	0.1735	TCARI	33.54	0.2373
55	MCARI1	25.58	0.2844	NDVI	26.47	0.0909	MCARI	35.67	0.1652	TCI	33.70	0.2298
56	MTVI1	25.58	0.2844	VSR	26.48	0.0901	CARI	35.68	0.1652	ZTSR6	33.71	0.2297
57	CRSR5	25.76	0.2747	SAVI2	26.48	0.0901	TCARI	35.70	0.1638	MCARI	33.76	0.2273
58	TVI	25.81	0.2720	WLCWMRG	26.49	0.0898	TCI	35.74	0.1622	CARI	33.76	0.2273
59	PSSRB	25.81	0.2716	MTCI	26.54	0.0859	BD	36.35	0.1335	BMDVI	34.30	0.2022
60	GI	25.84	0.2699	NLI	26.57	0.0842	BMDVI	36.64	0.1194	CAINT	34.50	0.1929
61	MCARI2	25.87	0.2683	OSAVI	26.59	0.0831	CAINT	36.65	0.1187	SPVI	34.59	0.1889
62	MTVI2	25.87	0.2683	BRI2	26.59	0.0829	ZTDP22	36.83	0.1103	BD	34.61	0.1879
63	CRSR3	25.91	0.2662	JSR	26.59	0.0826	PRI3	36.93	0.1055	ZTDP22	35.19	0.1603

Continued on next page

Table S.8 – Continued from previous page

Rank	2019-2020 data			2021-2022 data			All data, 80% random split			All data, 20% random split		
	Index	%RMSE	r^2	Index	%RMSE	r^2	Index	%RMSE	r^2	Index	%RMSE	r^2
64	WUMCARI	25.95	0.2637	CAR	26.60	0.0818	SPVI	36.98	0.1029	DND	35.42	0.1493
65	BMLSR	25.97	0.2629	TSAVI2	26.61	0.0813	MSI	36.99	0.1024	BMSR	35.61	0.1401
66	PSNDB	26.09	0.2559	PSSRA	26.61	0.0812	DND	37.11	0.0969	SRPI	35.63	0.1392
67	CAI	26.12	0.2543	WUMOR	26.62	0.0810	MND4	37.20	0.0924	MND4	35.65	0.1384
68	ZTDP21	26.15	0.2525	PSNDA	26.63	0.0797	BMSR	37.25	0.0898	ZTDPR1	35.67	0.1374
69	DREIP	26.25	0.2469	TCARI	26.69	0.0762	DSR1	37.26	0.0895	MSI	35.68	0.1370
70	WUTCARI	26.32	0.2426	MND3	26.69	0.0761	NPQI	37.29	0.0881	NDPI	35.69	0.1363
71	EGFN	26.37	0.2399	CRSR1	26.70	0.0752	TGI	37.33	0.0860	NPCI	35.69	0.1361
72	NDVI2	26.41	0.2372	EGFN	26.70	0.0750	NDWI	37.35	0.0850	PRI3	35.80	0.1310
73	ZTDPR1	26.45	0.2352	TGI	26.72	0.0737	SRWI	37.36	0.0845	TGI	35.83	0.1293
74	BGI2	26.45	0.2350	CI	26.73	0.0733	MND2	37.37	0.0838	NDWI	35.85	0.1287
75	BMDVI	26.46	0.2343	AIVI	26.73	0.0730	NDPI	37.38	0.0837	SRWI	35.86	0.1283
76	BGI1	26.48	0.2336	WLREIPG	26.74	0.0722	ZTDPR1	37.46	0.0796	MND2	35.87	0.1277
77	DSR2	26.65	0.2233	DD	26.74	0.0721	GNDVI	37.47	0.0791	GNDVI	35.99	0.1220
78	GRRGM	26.66	0.2228	MND1	26.75	0.0714	GI	37.62	0.0717	DSR1	36.05	0.1187
79	CPSR1	26.68	0.2217	BGI1	26.76	0.0710	PRI	37.69	0.0680	GEMI	36.52	0.0955
80	GMSR	26.68	0.2217	DNDR	26.76	0.0710	BMLSR	37.85	0.0605	GI	36.56	0.0937
81	VARI	26.69	0.2211	MND4	26.76	0.0707	WUTCARI	37.85	0.0603	EVI	36.59	0.0925
82	GTSR1	26.72	0.2195	WLREIPE	26.77	0.0701	BGI1	37.86	0.0596	BMLSR	36.63	0.0902
83	PSRI	27.00	0.2028	WLREIP2	26.78	0.0697	BGI2	37.88	0.0589	PVI	36.64	0.0899
84	GSUM2	27.01	0.2023	CPSR3	26.79	0.0691	SRPI	37.93	0.0562	WDVI	36.64	0.0899
85	PRI	27.07	0.1988	MSAVI1	26.79	0.0687	EGFN	37.94	0.0557	NPQI	36.66	0.0885
86	CVI	27.12	0.1957	MSAVI2	26.84	0.0652	NPCI	37.97	0.0545	EVI2	36.72	0.0856
87	BDR	27.24	0.1890	BRSR	26.85	0.0648	NDVI2	38.06	0.0499	DVI	36.73	0.0854
88	RGI	27.26	0.1879	DDN	26.86	0.0638	GEMI	38.11	0.0473	RDVI	36.75	0.0842
89	ZTDPR2	27.29	0.1857	VDR	26.87	0.0632	ESUM2	38.13	0.0465	RVIOPT	36.79	0.0823
90	DDR2	27.38	0.1802	ZTDR1	26.93	0.0591	PVI	38.14	0.0460	FSUM	36.84	0.0796
91	BRI1	27.71	0.1604	DDR1	26.94	0.0586	WDVI	38.14	0.0460	BGI2	36.84	0.0795
92	NPQI	27.83	0.1531	DSR1	26.96	0.0572	DVI	38.17	0.0444	SAVI	36.86	0.0787
93	WUMOR	27.83	0.1531	MND2	26.96	0.0571	EVI	38.20	0.0430	EGFN	36.90	0.0768
94	GRRREM	27.91	0.1487	SIPI	26.96	0.0569	FSUM	38.23	0.0415	BGI1	36.98	0.0730
95	ARI	28.03	0.1413	NDPI	26.97	0.0563	MCARI2	38.23	0.0414	ESUM2	36.98	0.0729
96	CPSR3	28.48	0.1132	PSSRC	26.98	0.0555	MTVI2	38.23	0.0414	WUTCARI	37.03	0.0704

Continued on next page

Table S.8 – Continued from previous page

	2019-2020 data			2021-2022 data			All data, 80% random split			All data, 20% random split		
Rank	Index	%RMSE	r^2	Index	%RMSE	r^2	Index	%RMSE	r^2	Index	%RMSE	r^2
97	ESUM1	28.60	0.1055	PSNDC	27.02	0.0532	EVI2	38.24	0.0410	ZTDPR2	37.08	0.0677
98	CAINT	28.72	0.0983	GRRREM	27.03	0.0520	ZTDPR2	38.25	0.0405	SIPI	37.11	0.0662
99	CRI700	28.74	0.0973	DND	27.05	0.0511	RDVI	38.25	0.0403	NDVI2	37.16	0.0637
100	SPVI	29.08	0.0753	BD	27.06	0.0501	DSR2	38.27	0.0394	PRI	37.20	0.0614
101	EVI	29.14	0.0718	CVI	27.08	0.0485	RVIOPT	38.27	0.0392	MSAVI2	37.21	0.0613
102	CRI500	29.17	0.0698	MCARI1	27.09	0.0479	GMSR	38.28	0.0387	MSAVI1	37.24	0.0599
103	PRI2	29.18	0.0688	MTVI1	27.09	0.0479	SAVI	38.31	0.0373	ZTSUM	37.25	0.0590
104	PSSRC	29.22	0.0664	SAVI	27.11	0.0465	GRRGM	38.34	0.0360	DSR2	37.46	0.0485
105	MSI	29.38	0.0563	TCI	27.13	0.0452	VARI	38.36	0.0346	GRRGM	37.47	0.0482
106	PSNDC	29.39	0.0555	NPCI	27.15	0.0440	GTSR1	38.39	0.0334	GMSR	37.48	0.0474
107	NDPI	29.40	0.0553	SRPI	27.16	0.0428	NDLI	38.41	0.0325	PSR	37.51	0.0462
108	SRWI	29.58	0.0434	MMR	27.17	0.0424	ZTSUM	38.44	0.0308	MCARI2	37.51	0.0460
109	NDWI	29.59	0.0431	DDR2	27.18	0.0418	LCA	38.50	0.0278	MTVI2	37.51	0.0460
110	BRI2	29.61	0.0414	NPQI	27.18	0.0416	MSAVI2	38.51	0.0273	WI	37.51	0.0459
111	WI	29.72	0.0344	RVIOPT	27.20	0.0399	MSAVI1	38.53	0.0261	GTSR1	37.54	0.0444
112	PSR	29.72	0.0343	EVI2	27.21	0.0392	MCARI1	38.58	0.0237	LCA	37.56	0.0437
113	NDNI	29.95	0.0194	TVI	27.22	0.0391	MTVI1	38.58	0.0237	PRI2	37.59	0.0420
114	SIPI	29.96	0.0189	RDVI	27.22	0.0390	CVI	38.60	0.0228	NDLI	37.64	0.0394
115	ZTSUM	30.00	0.0161	MOR	27.24	0.0377	GSUM2	38.61	0.0220	VARI	37.64	0.0393
116	FSUM	30.12	0.0083	BMDVI	27.24	0.0377	PRI2	38.62	0.0215	TSAVI2	37.80	0.0314
117	WNR	30.12	0.0080	ESUM2	27.28	0.0349	TVI	38.66	0.0197	OSAVI	37.86	0.0279
118	NDLI	30.16	0.0058	ESUM1	27.30	0.0333	RGI	38.73	0.0162	MCARI1	37.87	0.0278
119	MSR	30.17	0.0051	CARI	27.35	0.0298	PSRI	38.73	0.0160	MTVI1	37.87	0.0278
120	DVI	30.17	0.0048	MCARI	27.35	0.0298	CRI500	38.74	0.0158	NLI	37.87	0.0275
121	WDRVI	30.17	0.0046	BGI2	27.39	0.0264	SIPI	38.76	0.0146	GSUM2	37.88	0.0273
122	WDRVI2	30.18	0.0045	ZTDP22	27.40	0.0262	PSR	38.77	0.0141	CVI	37.91	0.0254
123	PVI	30.18	0.0043	CPSR1	27.40	0.0261	WI	38.77	0.0140	TVI	37.98	0.0220
124	WDVI	30.18	0.0043	ZTDPR1	27.48	0.0201	TSAVI2	38.81	0.0118	PSRI	38.05	0.0184
125	NDVI	30.18	0.0040	CRSR5	27.50	0.0192	OSAVI	38.85	0.0100	DREIP	38.05	0.0184
126	TSAVI	30.19	0.0038	GEMI	27.55	0.0153	NLI	38.85	0.0097	SAVI2	38.05	0.0183
127	DPI	30.19	0.0038	SPVI	27.61	0.0110	DREIP	38.88	0.0085	RGI	38.12	0.0148
128	GEMI	30.19	0.0033	EVI	27.61	0.0108	DPI	38.91	0.0070	CPSR3	38.16	0.0126
129	SAVI2	30.20	0.0030	CRI500	27.65	0.0080	CPSR3	38.92	0.0063	WNR	38.23	0.0089

Continued on next page

Table S.8 – Continued from previous page

	2019-2020 data			2021-2022 data			All data, 80% random split			All data, 20% random split		
Rank	Index	%RMSE	r^2	Index	%RMSE	r^2	Index	%RMSE	r^2	Index	%RMSE	r^2
130	JSR	30.20	0.0027	DSR2	27.67	0.0071	CRI700	38.93	0.0059	CRI700	38.26	0.0074
131	PD	30.20	0.0027	ZTDPR2	27.67	0.0069	SAVI2	38.94	0.0052	CRI500	38.28	0.0066
132	PRI3	30.20	0.0027	GMSR	27.68	0.0063	BRSR	39.02	0.0013	PSSRA	38.30	0.0053
133	PSSRA	30.21	0.0024	DREIP	27.68	0.0063	WNR	39.02	0.0012	MSR	38.31	0.0051
134	SRPI	30.21	0.0021	PSRI	27.68	0.0058	WLPD	39.02	0.0012	TSAVI	38.31	0.0051
135	NPCI	30.21	0.0021	NDVI2	27.69	0.0050	NDNI	39.03	0.0006	WLPD	38.31	0.0047
136	NLI	30.22	0.0017	GI	27.70	0.0046	TSAVI	39.03	0.0005	WDRVI	38.31	0.0046
137	PSNDA	30.22	0.0017	VARI	27.74	0.0018	MSR	39.04	0.0005	WDRVI2	38.32	0.0045
138	OSAVI	30.22	0.0013	WLPD	27.74	0.0018	WDRVI	39.04	0.0004	NDVI	38.33	0.0040
139	TSAVI2	30.23	0.0010	PVI	27.74	0.0017	WDRVI2	39.04	0.0004	PSNDA	38.33	0.0039
140	LCA	30.23	0.0009	WDVI	27.74	0.0017	PD	39.04	0.0003	PD	38.33	0.0036
141	WLPD	30.23	0.0008	RGI	27.76	0.0006	NDVI	39.04	0.0003	JSR	38.34	0.0034
142	EVI2	30.24	0.0006	MCARI2	27.76	0.0006	ESUM1	39.04	0.0003	DPI	38.36	0.0020
143	RDVI	30.24	0.0005	MTVI2	27.76	0.0006	PSSRA	39.04	0.0003	ARI	38.39	0.0006
144	SAVI	30.24	0.0003	DVI	27.76	0.0002	PSSRC	39.04	0.0002	PSSRC	38.40	0.0001
145	RVIOPT	30.24	0.0002	ZTSUM	27.76	0.0001	ARI	39.04	0.0002	ESUM1	38.40	0.0001
146	MSAVI1	30.24	0.0001	DPI	27.76	0.0001	PSNDA	39.04	0.0001	NDNI	38.40	0.0000
147	BRSR	30.24	0.0000	CRI700	27.76	0.0000	JSR	39.04	0.0001	BRSR	38.40	0.0000
148	MSAVI2	30.24	0.0000	FSUM	27.76	0.0000	PSNDC	39.04	0.0000	PSNDC	38.40	0.0000

Table S.9: Optimized machine learning hyperparameters for estimating area-basis cotton leaf chlorophyll $a+b$ (Chl $a+b$, μg cm^{-2}) with spectral reflectance data sets from field trials at Maricopa, Arizona, USA. Twelve machine learning models from Python's "scikit-learn" package were optimized, while two other methods (BayesianRidge and GaussianProcessRegressor) were also tested but required no hyperparameter optimization. Eight spectral data sets were tested, including spectral reflectance (ρ); the first and second derivatives of reflectance (ρ' and ρ'', respectively); the base-10 logarithm of the inverse of reflectance ($\log_{10}\rho^{-1}$) and its first and second derivatives [$(\log_{10}\rho^{-1})'$ and $(\log_{10}\rho^{-1})''$, respectively]; continuum-removed reflectance (ρ_{CR}); and the set of 148 spectral indices from Table S.1.

Method[1]	Parameter	ρ	ρ'	ρ''	$\log_{10}\rho^{-1}$	$(\log_{10}\rho^{-1})'$	$(\log_{10}\rho^{-1})''$	ρ_{CR}	Indices
		Training data from 2019–2020 experiment							
Ridge	alpha	2.1233e+1	1.0216e+3	3.7536e+3	3.5271e+1	9.6066e+2	3.1864e+3	4.7402e+2	2.5682
Lasso	alpha	1.2788e−3	9.3515e−3	2.2428e−2	1.0741e−3	9.7062e−3	2.3613e−2	6.1786e−3	3.2759e−5
LL	alpha	1.0676e−3	9.3227e−3	2.2427e−2	1.0337e−3	9.6425e−3	2.3639e−2	6.1993e−3	2.6476e−5
KR	alpha	2.4851e+1	1.0243e+3	3.7505e+3	1.3818e+1	9.6101e+2	3.1847e+3	4.8013e+2	1.3287e−2
SVR	C	8.9281	2.0270	1.4075	8.3574	2.0515	1.2452	2.9361	1.6430
KNR	n_neighbors	7	6	10	9	6	10	9	19
PLSR	n_components	12	9	4	16	8	5	10	43
DTR	max_depth	23	3	3	20	7	20	3	4
	min_samples_split	15	95	56	9	96	88	80	15
	min_samples_leaf	18	13	69	81	24	29	42	37
GBR	learning_rate	8.3962e−2	1.5042e−1	5.7794e−2	5.9188e−2	1.2151e−1	6.1419e−2	1.5161e−1	9.7607e−2
	max_depth	4	2	4	4	3	4	2	3
RFR	n_estimators	148	152	156	178	149	179	145	157
	min_samples_split	2	4	6	4	4	12	6	13
	min_samples_leaf	4	4	2	1	2	5	4	6
ABR	n_estimators	120	199	190	145	185	198	178	167
	learning_rate	2.8835	2.9885	3.0770	3.0906	2.8976	3.0788	2.6411	2.8735
MLPR	hidden_layer_sizes	16	200	117	21	158	64	197	113
	alpha	2.8441e+1	9.2163e+1	1.6468e+2	2.9438e+1	8.7139e+1	1.6479e+2	8.0923e+1	1.3636e+1
		Training data from 80% random split off all data from 2019–2020 and 2021–2022 experiments							
Ridge	alpha	1.0058e+1	7.3014e+2	1.2680e+3	4.4239	7.5889e+2	1.3042e+3	7.2081e+2	3.2763
Lasso	alpha	1.0025e−3	1.2567e−2	8.8014e−3	1.0613e−3	1.3877e−2	7.9739e−3	1.9930e−2	8.2580e−5
LL	alpha	2.8277e−4	1.2592e−2	8.7822e−3	8.0236e−5	1.3886e−2	7.9466e−3	2.0087e−2	2.9333e−5
KR	alpha	1.1944	7.2350e+2	1.2309e+3	5.4315e−1	7.4673e+2	1.2762e+3	7.1582e+2	4.3558e−3
SVR	C	1.2964e+1	2.7439	2.2587	5.7037	2.9720	2.1605	4.8950	2.6507
KNR	n_neighbors	8	13	7	8	6	4	6	5
PLSR	n_components	24	8	6	31	8	7	9	50
DTR	max_depth	13	15	4	19	17	27	20	10
	min_samples_split	50	5	69	51	93	91	71	22
	min_samples_leaf	14	39	13	14	25	17	41	17
GBR	learning_rate	5.3776e−2	7.5217e−2	7.7772e−2	4.3479e−2	1.1472e−1	9.8624e−2	8.8367e−2	6.6244e−2
	max_depth	4	3	4	4	3	3	4	5
RFR	n_estimators	121	154	200	194	145	103	185	199
	min_samples_split	4	2	10	5	9	8	2	5
	min_samples_leaf	1	2	5	3	2	3	2	2
ABR	n_estimators	161	167	181	196	190	197	182	135
	learning_rate	3.1450	2.1962	2.3175	2.8454	1.9115	2.5721	3.0486	2.9293
MLPR	hidden_layer_sizes	11	203	236	10	140	198	234	65
	alpha	3.6833e+1	8.4195e+1	1.1791e+2	1.4084e+1	9.7559e+1	1.2826e+2	1.0328e+2	1.2770e+1

[1] AdaBoostRegressor, ABR; DecisionTreeRegressor, DTR; GradientBoostingRegressor, GBR; KernelRidge, KR; KNeighborsRegressor, KNR; LassoLars, LL; MLPRegressor, MLPR; PLSRegression, PLSR; RandomForestRegressor, RFR; and support vector regression, SVR.

Table S.10: Optimized machine learning hyperparameters for estimating area-basis cotton leaf chlorophyll a (Chl a, μg cm^{-2}) with spectral reflectance data sets from field trials at Maricopa, Arizona, USA. Twelve machine learning models from Python's "scikit-learn" package were optimized, while two other methods (BayesianRidge and GaussianProcessRegressor) were also tested but required no hyperparameter optimization. Eight spectral data sets were tested, including spectral reflectance (ρ); the first and second derivatives of reflectance (ρ' and ρ'', respectively); the base-10 logarithm of the inverse of reflectance ($\log_{10}\rho^{-1}$) and its first and second derivatives [$(\log_{10}\rho^{-1})'$ and $(\log_{10}\rho^{-1})''$, respectively]; continuum-removed reflectance (ρ_{CR}); and the set of 148 spectral indices from Table S.1.

Method[1]	Parameter	ρ	ρ'	ρ''	$\log_{10}\rho^{-1}$	$(\log_{10}\rho^{-1})'$	$(\log_{10}\rho^{-1})''$	ρ_{CR}	Indices
		Training data from 2019–2020 experiment							
Ridge	alpha	4.3758	7.6047e + 2	2.8270e + 3	7.5204e − 1	7.2848e + 2	2.4551e + 3	3.5428e + 2	3.1491e + 1
Lasso	alpha	1.1717e − 3	7.9082e − 3	1.9336e − 2	1.0801e − 3	8.1020e − 3	1.7111e − 2	5.5708e − 3	2.9155e − 5
LL	alpha	6.1306e − 4	7.8700e − 3	1.9362e − 2	4.3280e − 4	8.2228e − 3	1.7102e − 2	5.4784e − 3	2.0253e − 5
KR	alpha	4.1811	7.6054e + 2	2.8048e + 3	2.7782	7.2358e + 2	2.4563e + 3	3.5625e + 2	2.6500e − 2
SVR	C	8.5499	2.4174	1.7638	8.4880	2.3668	1.5490	2.5167	2.0815
KNR	n_neighbors	8	5	10	9	7	9	7	9
PLSR	n_components	19	10	5	19	9	5	11	43
DTR	max_depth	25	3	11	6	17	6	4	4
	min_samples_split	40	95	110	40	89	77	107	24
	min_samples_leaf	31	68	38	20	53	37	46	26
GBR	learning_rate	6.4643e − 2	7.9405e − 2	1.1270e − 1	7.6343e − 2	1.4973e − 1	9.1264e − 2	1.3042e − 1	6.3665e − 2
	max_depth	4	3	3	4	3	3	3	3
RFR	n_estimators	130	171	195	183	173	132	157	152
	min_samples_split	2	7	4	10	3	11	3	7
	min_samples_leaf	3	2	1	2	1	4	2	3
ABR	n_estimators	192	160	195	193	176	195	194	153
	learning_rate	3.0357	2.9059	3.1185	2.3167	2.8246	3.1147	2.9733	3.0172
MLPR	hidden_layer_sizes	57	153	70	33	145	130	245	172
	alpha	3.8149e + 1	8.1006e + 1	1.4281e + 2	2.9250e + 1	8.4347e + 1	1.6326e + 2	7.2452e + 1	1.2053e + 1
		Training data from 80% random split off all data from 2019–2020 and 2021–2022 experiments							
Ridge	alpha	6.9434	7.3173e + 2	1.3349e + 3	2.0867	8.4280e + 2	1.5238e + 3	6.4864e + 2	1.5052
Lasso	alpha	1.1099e − 3	1.1124e − 2	8.5521e − 3	1.1276e − 3	1.2054e − 2	8.0147e − 3	1.9130e − 2	7.1667e − 5
LL	alpha	3.1953e − 4	1.1201e − 2	8.6519e − 3	9.2384e − 5	1.1973e − 2	8.0258e − 3	1.8771e − 2	3.1019e − 5
KR	alpha	1.5314	7.2594e + 2	1.3135e + 3	8.0840e − 1	8.3849e + 2	1.5116e + 3	6.4124e + 2	2.1696e − 2
SVR	C	6.1861	3.8943	3.2787	6.5348	2.9149	3.3243	4.5995	2.8691
KNR	n_neighbors	7	13	7	8	6	7	5	6
PLSR	n_components	23	8	5	31	7	5	9	50
DTR	max_depth	12	21	4	11	16	27	16	11
	min_samples_split	9	66	47	16	19	87	87	23
	min_samples_leaf	31	20	11	31	29	35	14	24
GBR	learning_rate	9.9576e − 2	1.2147e − 1	9.1384e − 2	9.1952e − 2	7.2795e − 2	8.5930e − 2	9.7176e − 2	6.4430e − 2
	max_depth	4	3	4	4	4	4	3	4
RFR	n_estimators	187	167	123	135	186	127	198	194
	min_samples_split	7	5	10	6	5	2	4	3
	min_samples_leaf	1	2	3	2	4	1	1	2
ABR	n_estimators	153	186	134	169	184	184	194	193
	learning_rate	2.9957	2.0234	1.4537	3.1222	2.2469	2.8122	2.8097	3.0435
MLPR	hidden_layer_sizes	17	192	217	11	217	212	209	194
	alpha	2.6073e + 1	7.0779e + 1	7.5004e + 1	3.0450e + 1	8.6691e + 1	1.4779e + 2	9.2568e + 1	1.6873e + 1

[1] AdaBoostRegressor, ABR; DecisionTreeRegressor, DTR; GradientBoostingRegressor, GBR; KernelRidge, KR; KNeighborsRegressor, KNR; LassoLars, LL; MLPRegressor, MLPR; PLSRegression, PLSR; RandomForestRegressor, RFR; and support vector regression, SVR.

Table S.11: Optimized machine learning hyperparameters for estimating area-basis cotton leaf chlorophyll b (Chl b, μg cm^{-2}) with spectral reflectance data sets from field trials at Maricopa, Arizona, USA. Twelve machine learning models from Python's "scikit-learn" package were optimized, while two other methods (BayesianRidge and GaussianProcessRegressor) were also tested but required no hyperparameter optimization. Eight spectral data sets were tested, including spectral reflectance (ρ); the first and second derivatives of reflectance (ρ' and ρ'', respectively); the base-10 logarithm of the inverse of reflectance ($\log_{10}\rho^{-1}$) and its first and second derivatives [$(\log_{10}\rho^{-1})'$ and $(\log_{10}\rho^{-1})''$, respectively]; continuum-removed reflectance (ρ_{CR}); and the set of 148 spectral indices from Table S.1.

Method[1]	Parameter	ρ	ρ'	ρ''	$\log_{10}\rho^{-1}$	$(\log_{10}\rho^{-1})'$	$(\log_{10}\rho^{-1})''$	ρ_{CR}	Indices
		Training data from 2019–2020 experiment							
Ridge	alpha	2.1000e+1	1.0600e+3	3.1822e+3	1.8958e+1	1.1699e+3	3.0498e+3	5.4796e+2	2.5899e+1
Lasso	alpha	1.8056e−3	1.4403e−2	2.7565e−2	1.8995e−3	1.4485e−2	2.6787e−2	1.0874e−2	3.4427e−4
LL	alpha	1.5160e−3	1.4416e−2	2.7559e−2	1.5420e−3	1.4450e−2	2.6765e−2	1.0835e−2	1.6982e−4
KR	alpha	1.9749e+1	1.0541e+3	3.1565e+3	1.6192e+1	1.1611e+3	3.0559e+3	5.4701e+2	2.6780e−2
SVR	C	3.9538	2.8740	2.2818	4.0420	4.0385	2.0921	2.9312	2.3755
KNR	n_neighbors	11	8	15	12	8	15	12	12
PLSR	n_components	17	8	5	17	7	4	9	38
DTR	max_depth	5	20	8	16	19	6	6	29
	min_samples_split	53	114	87	58	30	33	61	52
	min_samples_leaf	36	59	65	37	46	67	27	56
GBR	learning_rate	1.0919e−1	7.3961e−2	8.9049e−2	5.0590e−2	6.6735e−2	1.0507e−1	1.0317e−1	5.4121e−2
	max_depth	3	3	4	4	5	3	4	4
RFR	n_estimators	197	114	126	168	162	159	133	150
	min_samples_split	12	12	8	11	3	9	5	9
	min_samples_leaf	5	7	3	5	3	5	1	4
ABR	n_estimators	147	161	174	187	185	196	157	155
	learning_rate	3.1022	2.4941	2.6619	3.1360	2.0309	2.7390	3.0017	4.2698e−2
MLPR	hidden_layer_sizes	8	228	151	8	231	206	166	215
	alpha	5.2820e+1	1.1242e+2	1.4427e+2	3.5024e+1	9.5292e+1	1.3070e+2	8.9453e+1	2.2428e+1
		Training data from 80% random split off all data from 2019–2020 and 2021–2022 experiments							
Ridge	alpha	5.9104	5.0946e+2	1.5135e+3	2.8684e+1	5.3362e+2	1.2811e+3	5.6996e+2	8.1207
Lasso	alpha	1.0375e−3	4.4216e−3	1.5610e−2	1.0281e−3	4.9919e−3	8.7195e−3	1.0324e−2	9.4485e−5
LL	alpha	2.9443e−4	4.7676e−3	1.5653e−2	1.6998e−4	5.0725e−3	9.0661e−3	1.0307e−2	3.0182e−5
KR	alpha	9.4380e−1	5.0665e+2	1.4808e+3	5.8498e−1	5.1805e+2	1.2645e+3	5.7581e+2	2.1685e−1
SVR	C	7.6974	2.2163	2.1851	7.5634	2.1855	2.1316	4.4018	2.7631
KNR	n_neighbors	11	11	7	9	17	10	4	6
PLSR	n_components	24	11	6	30	11	7	10	41
DTR	max_depth	27	12	11	8	27	8	22	23
	min_samples_split	34	60	146	47	110	114	88	52
	min_samples_leaf	29	20	33	28	6	3	11	39
GBR	learning_rate	1.1733e−1	1.0620e−1	6.1558e−2	6.9144e−2	8.1892e−2	9.5978e−2	7.7113e−2	5.2769e−2
	max_depth	4	3	4	5	4	4	4	5
RFR	n_estimators	155	184	182	198	151	200	194	199
	min_samples_split	6	14	8	12	9	3	2	3
	min_samples_leaf	4	5	1	4	4	1	3	4
ABR	n_estimators	88	146	196	169	175	197	167	135
	learning_rate	1.8095e−1	1.4877e−1	3.1107	5.4709e−2	1.5819e−1	2.9824	3.0269	1.0485e−1
MLPR	hidden_layer_sizes	10	242	161	14	156	172	180	231
	alpha	2.6476e+1	9.6744e+1	1.9307e+2	2.8042e+1	1.0438e+2	1.4048e+2	1.2637e+2	1.2051e+1

[1] AdaBoostRegressor, ABR; DecisionTreeRegressor, DTR; GradientBoostingRegressor, GBR; KernelRidge, KR; KNeighborsRegressor, KNR; LassoLars, LL; MLPRegressor, MLPR; PLSRegression, PLSR; RandomForestRegressor, RFR; and support vector regression, SVR.

Table S.12: Optimized machine learning hyperparameters for estimating mass-basis cotton leaf chlorophyll $a+b$ (Chl $a+b$, mg g^{-1}) with spectral reflectance data sets from field trials at Maricopa, Arizona, USA. Twelve machine learning models from Python's "scikit-learn" package were optimized, while two other methods (BayesianRidge and GaussianProcessRegressor) were also tested but required no hyperparameter optimization. Eight spectral data sets were tested, including spectral reflectance (ρ); the first and second derivatives of reflectance (ρ' and ρ'', respectively); the base-10 logarithm of the inverse of reflectance ($\log_{10}\rho^{-1}$) and its first and second derivatives [$(\log_{10}\rho^{-1})'$ and $(\log_{10}\rho^{-1})''$, respectively]; continuum-removed reflectance (ρ_{CR}); and the set of 148 spectral indices from Table S.1.

Method[1]	Parameter	ρ	ρ'	ρ''	$\log_{10}\rho^{-1}$	$(\log_{10}\rho^{-1})'$	$(\log_{10}\rho^{-1})''$	ρ_{CR}	Indices
		Training data from 2019–2020 experiment							
Ridge	alpha	2.6129e + 1	4.3979e + 2	1.6125e + 3	4.3128	5.1013e + 2	1.7987e + 3	1.4293e + 2	7.6813
Lasso	alpha	1.1351e − 3	7.7622e − 3	1.5344e − 2	1.1479e − 3	8.0023e − 3	1.3856e − 2	4.0268e − 3	3.0989e − 5
LL	alpha	1.9087e − 4	7.7464e − 3	1.5335e − 2	2.9895e − 4	7.9361e − 3	1.3814e − 2	4.2427e − 3	1.4819e − 4
KR	alpha	6.8197e − 1	4.3653e + 2	1.6253e + 3	8.4039e − 1	4.9114e + 2	1.7977e + 3	1.4396e + 2	6.9133e − 2
SVR	C	8.8849	3.4902	4.1198e + 1	8.5284	2.6529	1.9665e + 1	4.4018	3.3968
KNR	n_neighbors	11	7	10	9	8	7	9	8
PLSR	n_components	22	11	6	21	11	5	12	37
DTR	max_depth	4	27	22	4	22	12	6	23
	min_samples_split	107	2	79	98	21	37	57	71
	min_samples_leaf	2	57	33	22	35	35	18	34
GBR	learning_rate	1.4662e − 1	1.1175e − 1	8.6736e − 2	1.3519e − 1	9.4376e − 2	1.2222e − 1	9.2454e − 2	1.0102e − 1
	max_depth	3	3	3	3	3	3	4	4
RFR	n_estimators	115	175	182	117	132	149	171	198
	min_samples_split	7	4	3	7	2	3	4	7
	min_samples_leaf	3	3	1	2	5	5	3	1
ABR	n_estimators	175	196	168	163	166	162	184	180
	learning_rate	2.9440	3.2051	2.7508	3.1452	3.0695	2.9912	3.1275	2.9370
MLPR	hidden_layer_sizes	48	110	101	8	136	211	137	233
	alpha	3.6983e + 1	9.3092e + 1	1.6500e + 2	3.1073e + 1	8.7836e + 1	1.5384e + 2	8.1955e + 1	1.7580e + 1
		Training data from 80% random split off all data from 2019–2020 and 2021–2022 experiments							
Ridge	alpha	6.7766	8.1319e + 2	1.4494e + 3	2.0396	8.4783e + 2	1.4048e + 3	3.7399e + 2	1.4252
Lasso	alpha	1.3701e − 3	1.2750e − 2	1.8399e − 2	1.8995e − 3	1.1311e − 2	1.7871e − 2	6.5785e − 3	6.2852e − 6
LL	alpha	5.5598e − 4	1.2761e − 2	1.8426e − 2	2.3493e − 4	1.1333e − 2	1.8057e − 2	6.7235e − 3	1.1344e − 4
KR	alpha	2.3473	8.0477e + 2	1.4378e + 3	1.8286	8.5268e + 2	1.3990e + 3	3.6964e + 2	1.7291e − 1
SVR	C	1.2979e + 1	2.8112	2.1457	8.8202	3.1717	2.2318	3.9550	3.1172
KNR	n_neighbors	12	11	19	18	9	18	8	8
PLSR	n_components	24	9	6	23	8	7	12	33
DTR	max_depth	25	14	14	14	21	5	21	21
	min_samples_split	95	97	116	21	96	108	90	24
	min_samples_leaf	68	66	17	48	34	41	62	44
GBR	learning_rate	1.1924e − 1	7.3169e − 2	7.7613e − 2	8.3652e − 2	9.7670e − 2	9.1053e − 2	6.5196e − 2	7.2181e − 2
	max_depth	4	4	4	5	3	4	4	4
RFR	n_estimators	196	188	179	153	115	166	112	113
	min_samples_split	14	2	2	6	10	3	3	2
	min_samples_leaf	1	1	5	3	4	2	1	2
ABR	n_estimators	196	171	199	188	165	184	198	196
	learning_rate	3.2213	3.1982	2.5561	2.9736	2.5508	1.5234	3.0150	3.1093
MLPR	hidden_layer_sizes	51	214	184	16	202	147	151	221
	alpha	4.6644e + 1	1.5093e + 2	2.0849e + 2	4.2591e + 1	1.3682e + 2	2.2152e + 2	9.5274e + 1	2.1356e + 1

[1] AdaBoostRegressor, ABR; DecisionTreeRegressor, DTR; GradientBoostingRegressor, GBR; KernelRidge, KR; KNeighborsRegressor, KNR; LassoLars, LL; MLPRegressor, MLPR; PLSRegression, PLSR; RandomForestRegressor, RFR; and support vector regression, SVR.

Table S.13: Optimized machine learning hyperparameters for estimating mass-basis cotton leaf chlorophyll a (Chl a, mg g^{-1}) with spectral reflectance data sets from field trials at Maricopa, Arizona, USA. Twelve machine learning models from Python's "scikit-learn" package were optimized, while two other methods (BayesianRidge and GaussianProcessRegressor) were also tested but required no hyperparameter optimization. Eight spectral data sets were tested, including spectral reflectance (ρ); the first and second derivatives of reflectance (ρ' and ρ'', respectively); the base-10 logarithm of the inverse of reflectance ($\log_{10}\rho^{-1}$) and its first and second derivatives [$(\log_{10}\rho^{-1})'$ and $(\log_{10}\rho^{-1})''$, respectively]; continuum-removed reflectance (ρ_{CR}); and the set of 148 spectral indices from Table S.1.

Method[1]	Parameter	ρ	ρ'	ρ''	$\log_{10}\rho^{-1}$	$(\log_{10}\rho^{-1})'$	$(\log_{10}\rho^{-1})''$	ρ_{CR}	Indices
		Training data from 2019–2020 experiment							
Ridge	alpha	1.1260e + 1	3.3964e + 2	1.2518e + 3	1.3474e − 1	3.9476e + 2	1.4489e + 3	8.4244e + 1	3.1247e + 1
Lasso	alpha	1.2233e − 3	6.9750e − 3	1.3931e − 2	1.2115e − 3	6.6075e − 3	1.3243e − 2	4.1425e − 3	7.6826e − 5
LL	alpha	1.6655e − 4	6.8952e − 3	1.3895e − 2	2.5767e − 4	6.5750e − 3	1.3133e − 2	4.1169e − 3	3.5077e − 5
KR	alpha	3.6560e − 1	3.4710e + 2	1.2548e + 3	4.9950e − 1	3.9510e + 2	1.4465e + 3	8.5069e + 1	1.8500e − 2
SVR	C	1.2643e + 1	3.7824	3.9257e + 1	1.1883e + 1	2.4085	9.3746e + 1	3.9439	3.5025
KNR	n_neighbors	13	6	10	13	7	7	8	6
PLSR	n_components	29	11	6	21	10	6	12	37
DTR	max_depth	25	20	26	19	7	24	25	23
	min_samples_split	97	79	21	144	70	16	83	51
	min_samples_leaf	88	30	31	88	35	40	17	23
GBR	learning_rate	1.4911e − 1	1.1156e − 1	9.6497e − 2	1.4617e − 1	1.3842e − 1	9.6663e − 2	8.8162e − 2	9.4260e − 2
	max_depth	3	3	3	3	3	3	4	4
RFR	n_estimators	144	184	193	162	198	175	195	190
	min_samples_split	7	4	9	4	10	12	6	4
	min_samples_leaf	3	1	3	2	3	6	3	3
ABR	n_estimators	187	187	181	199	196	191	198	174
	learning_rate	2.9465	2.8500	3.0868	3.1813	2.8368	3.0287	3.1210	3.0574
MLPR	hidden_layer_sizes	6	145	221	6	237	162	85	217
	alpha	2.6457e + 1	7.8411e + 1	1.5446e + 2	2.4704e + 1	8.1916e + 1	1.5750e + 2	7.4018e + 1	1.7152e + 1
		Training data from 80% random split off all data from 2019–2020 and 2021–2022 experiments							
Ridge	alpha	6.9719	7.4751e + 2	1.5188e + 3	1.4612e + 1	8.0264e + 2	1.5501e + 3	3.4253e + 2	8.4517
Lasso	alpha	1.4564e − 3	1.2180e − 2	1.6591e − 2	1.6401e − 3	1.1726e − 2	1.9916e − 2	7.4246e − 3	5.7278e − 5
LL	alpha	3.6613e − 4	1.2211e − 2	1.6598e − 2	2.6352e − 4	1.1688e − 2	2.0004e − 2	7.2085e − 3	5.9673e − 5
KR	alpha	2.2449	7.3053e + 2	1.4939e + 3	1.8227	7.9351e + 2	1.5446e + 3	3.4917e + 2	1.7609e − 1
SVR	C	9.7411	3.3488	2.5432	8.5889	3.2820	2.3579	3.7928	3.5626
KNR	n_neighbors	15	11	18	18	9	13	7	8
PLSR	n_components	24	10	6	22	9	7	12	33
DTR	max_depth	10	24	19	22	9	8	10	25
	min_samples_split	141	31	9	88	96	18	69	20
	min_samples_leaf	99	63	51	100	35	51	46	29
GBR	learning_rate	7.8052e − 2	1.1019e − 1	8.5373e − 2	1.4701e − 1	5.2501e − 2	8.2877e − 2	8.0359e − 2	6.1827e − 2
	max_depth	5	3	4	4	4	3	4	5
RFR	n_estimators	169	167	153	133	95	145	194	198
	min_samples_split	11	9	3	8	6	2	4	3
	min_samples_leaf	2	3	2	2	5	5	5	2
ABR	n_estimators	194	191	195	179	184	190	183	168
	learning_rate	2.7530	3.0000	2.5993	3.0479	2.7329	2.5803	2.7827	3.1507
MLPR	hidden_layer_sizes	27	230	191	12	236	109	94	94
	alpha	4.0155e + 1	1.5392e + 2	2.3210e + 2	5.2987e + 1	1.6992e + 2	2.3008e + 2	9.3366e + 1	1.7640e + 1

[1] AdaBoostRegressor, ABR; DecisionTreeRegressor, DTR; GradientBoostingRegressor, GBR; KernelRidge, KR; KNeighborsRegressor, KNR; LassoLars, LL; MLPRegressor, MLPR; PLSRegression, PLSR; RandomForestRegressor, RFR; and support vector regression, SVR.

Table S.14: Optimized machine learning hyperparameters for estimating mass-basis cotton leaf chlorophyll b (Chl b, mg g^{-1}) with spectral reflectance data sets from field trials at Maricopa, Arizona, USA. Twelve machine learning models from Python's "scikit-learn" package were optimized, while two other methods (BayesianRidge and GaussianProcessRegressor) were also tested but required no hyperparameter optimization. Eight spectral data sets were tested, including spectral reflectance (ρ); the first and second derivatives of reflectance (ρ' and ρ'', respectively); the base-10 logarithm of the inverse of reflectance ($\log_{10}\rho^{-1}$) and its first and second derivatives [$(\log_{10}\rho^{-1})'$ and $(\log_{10}\rho^{-1})''$, respectively]; continuum-removed reflectance (ρ_{CR}); and the set of 148 spectral indices from Table S.1.

Method[1]	Parameter	ρ	ρ'	ρ''	$\log_{10}\rho^{-1}$	$(\log_{10}\rho^{-1})'$	$(\log_{10}\rho^{-1})''$	ρ_{CR}	Indices
		Training data from 2019–2020 experiment							
Ridge	alpha	2.3671e+1	1.1463e+3	3.9293e+3	1.1867e+1	1.3110e+3	3.7199e+3	5.8067e+2	1.4865e+1
Lasso	alpha	1.4807e−3	1.1173e−2	3.0667e−2	1.4743e−3	1.5368e−2	2.8467e−2	8.8840e−3	1.3931e−3
LL	alpha	8.4717e−4	1.1196e−2	3.0644e−2	7.8102e−4	1.5381e−2	2.8456e−2	9.0877e−3	3.0715e−4
KR	alpha	1.3976e+1	1.1373e+3	3.9242e+3	1.2382e+1	1.3047e+3	3.7154e+3	5.7661e+2	2.1995e−1
SVR	C	4.5823	3.2409	2.7727e+1	4.1724	4.4293	4.5985e+1	6.6085	2.3404
KNR	n_neighbors	15	13	16	15	8	16	15	15
PLSR	n_components	13	8	4	21	6	4	9	34
DTR	max_depth	5	22	18	28	4	15	9	12
	min_samples_split	107	75	75	99	6	115	72	49
	min_samples_leaf	41	45	71	40	33	35	44	31
GBR	learning_rate	1.1400e−1	9.4971e−2	6.7435e−2	1.4792e−1	1.0260e−1	9.8423e−2	1.3833e−1	4.8788e−2
	max_depth	3	3	4	3	4	3	3	4
RFR	n_estimators	199	142	159	177	123	136	190	110
	min_samples_split	3	3	3	3	5	10	3	12
	min_samples_leaf	6	3	1	2	2	7	7	3
ABR	n_estimators	149	147	199	163	189	194	179	146
	learning_rate	3.1429	3.0624	2.8891	3.1647	2.8853	2.8924	2.6621	2.9741
MLPR	hidden_layer_sizes	11	169	100	4	244	142	147	239
	alpha	3.6332e+1	1.1946e+2	2.0202e+2	2.9950e+1	9.6175e+1	1.9712e+2	9.2657e+1	2.2360e+1
		Training data from 80% random split off all data from 2019–2020 and 2021–2022 experiments							
Ridge	alpha	4.5884	8.3607e+2	1.6742e+3	3.8493	8.2044e+2	1.3825e+3	4.6445e+2	1.3736e−1
Lasso	alpha	1.4760e−3	9.1928e−3	1.5619e−2	2.1849e−3	9.9070e−3	1.2516e−2	9.7035e−3	1.4450e−4
LL	alpha	4.7921e−4	9.2035e−3	1.5600e−2	2.7687e−4	9.9180e−3	1.2732e−2	9.7582e−3	1.2097e−4
KR	alpha	2.7646	8.3278e+2	1.6555e+3	1.7423	8.1474e+2	1.3771e+3	4.6630e+2	1.6070e−1
SVR	C	1.2359e+1	3.3789	2.4352	1.0020e+1	2.7695	1.4231e+1	3.6037	3.2888
KNR	n_neighbors	11	17	23	13	19	27	8	7
PLSR	n_components	24	8	5	24	8	6	10	35
DTR	max_depth	8	14	7	18	19	19	28	9
	min_samples_split	49	61	149	51	84	69	49	61
	min_samples_leaf	37	17	6	29	8	50	36	24
GBR	learning_rate	8.8682e−2	6.9291e−2	5.4730e−2	1.5621e−1	1.1233e−1	1.0882e−1	9.1702e−2	6.1642e−2
	max_depth	5	3	4	4	2	3	3	4
RFR	n_estimators	159	111	170	108	155	121	200	196
	min_samples_split	11	2	3	2	11	4	8	14
	min_samples_leaf	2	3	1	1	2	3	4	2
ABR	n_estimators	167	174	193	179	159	184	181	159
	learning_rate	3.1855	2.8480	3.0627	3.1661	3.1124	3.1076	2.8220	3.0519
MLPR	hidden_layer_sizes	11	182	231	11	217	238	142	221
	alpha	3.1257e+1	1.2600e+2	1.6015e+2	4.2054e+1	1.0121e+2	1.3643e+2	9.2929e+1	1.9572e+1

[1] AdaBoostRegressor, ABR; DecisionTreeRegressor, DTR; GradientBoostingRegressor, GBR; KernelRidge, KR; KNeighborsRegressor, KNR; LassoLars, LL; MLPRegressor, MLPR; PLSRegression, PLSR; RandomForestRegressor, RFR; and support vector regression, SVR.

Table S.15: Goodness-of-fit statistics, including root mean squared errors (%RMSE) and coefficients of determination (r^2) between measured and modeled area-basis cotton leaf chlorophyll $a + b$ (Chl $a + b$, μg cm^{-2}) for training and testing of 14 machine learning methods from Python's "scikit-learn" package with 8 input data sets derived from leaf spectral reflectance (ρ). The 8 input data sets included spectral reflectance (ρ); the first and second derivatives of reflectance (ρ' and ρ'', respectively); the base-10 logarithm of the inverse of reflectance ($\log_{10}\rho^{-1}$) and its first and second derivatives [$(\log_{10}\rho^{-1})'$ and $(\log_{10}\rho^{-1})''$, respectively]; continuum-removed reflectance (ρ_{CR}); and the set of 148 spectral indices from Table S.1. Models were trained and tested by experiment using data from the 2019–2020 and 2021–2022 cotton field studies at Maricopa, Arizona, USA, respectively. Models were also trained and tested using an 80% and 20% random split of all from both experiments. Results are ranked according to the %RMSE of model testing.

	Training on 2019–2020 data and testing on 2021–2022 data						Training and testing based on 80%-20% random split of all data					
Rank	Method[1]	Input data	Training %RMSE	Testing %RMSE	Training r^2	Testing r^2	Method[1]	Input data	Training %RMSE	Testing %RMSE	Training r^2	Testing r^2
1	MLPR	ρ'	3.90	23.69	0.9414	0.4649	RFR	$[\log_{10}(\rho^{-1})]'$	5.47	10.47	0.9653	0.8762
2	MLPR	$[\log_{10}(\rho^{-1})]'$	3.75	24.44	0.9460	0.4613	MLPR	148 indices	9.46	10.55	0.8829	0.8682
3	PLSR	$[\log_{10}(\rho^{-1})]'$	6.67	24.54	0.8207	0.5067	GBR	$[\log_{10}(\rho^{-1})]'$	4.45	10.63	0.9750	0.8661
4	PLSR	ρ'	6.57	24.55	0.8260	0.4680	GBR	ρ'	5.72	10.92	0.9592	0.8575
5	Lasso	$[\log_{10}(\rho^{-1})]''$	6.80	24.83	0.8159	0.4966	RFR	ρ'	4.93	10.94	0.9717	0.8594
6	LL	$[\log_{10}(\rho^{-1})]''$	6.80	24.83	0.8158	0.4967	GBR	ρ''	2.92	11.01	0.9895	0.8571
7	BR	ρ'	6.08	24.95	0.8521	0.4863	ABR	$[\log_{10}(\rho^{-1})]'$	9.42	11.01	0.8889	0.8655
8	Ridge	ρ'	6.23	25.03	0.8449	0.4887	RFR	$[\log_{10}(\rho^{-1})]''$	5.55	11.04	0.9650	0.8617
9	KR	ρ'	6.23	25.03	0.8448	0.4887	RFR	ρ''	6.20	11.14	0.9546	0.8569
10	LL	ρ''	6.77	25.10	0.8174	0.4750	MLPR	ρ	10.26	11.15	0.8621	0.8579
11	BR	$[\log_{10}(\rho^{-1})]'$	6.02	25.11	0.8554	0.5067	GBR	$[\log_{10}(\rho^{-1})]''$	4.65	11.17	0.9730	0.8530
12	Lasso	ρ''	6.77	25.12	0.8174	0.4747	SVR	148 indices	9.55	11.28	0.8816	0.8543
13	BR	$[\log_{10}(\rho^{-1})]''$	5.97	25.13	0.8581	0.4789	SVR	ρ'	3.23	11.41	0.9875	0.8498
14	Ridge	$[\log_{10}(\rho^{-1})]'$	6.15	25.18	0.8489	0.5110	SVR	$[\log_{10}(\rho^{-1})]'$	3.09	11.51	0.9885	0.8518
15	KR	$[\log_{10}(\rho^{-1})]'$	6.15	25.18	0.8488	0.5110	SVR	ρ	8.20	11.57	0.9138	0.8458
16	BR	ρ''	6.05	25.21	0.8540	0.4679	ABR	ρ'	8.90	11.57	0.9045	0.8482
17	Lasso	ρ'	6.34	25.23	0.8391	0.4957	ABR	$[\log_{10}(\rho^{-1})]''$	9.65	11.59	0.8898	0.8577
18	LL	ρ'	6.34	25.24	0.8392	0.4959	SVR	ρ_{CR}	4.61	11.67	0.9736	0.8400
19	PLSR	$[\log_{10}(\rho^{-1})]''$	6.74	25.29	0.8171	0.4946	ABR	ρ''	9.37	12.00	0.8938	0.8384
20	MLPR	ρ''	4.16	25.32	0.9341	0.4737	KR	148 indices	11.19	12.03	0.8351	0.8279
21	MLPR	$[\log_{10}(\rho^{-1})]''$	4.10	25.36	0.9363	0.4969	LL	148 indices	11.24	12.04	0.8336	0.8280
22	KR	$[\log_{10}(\rho^{-1})]''$	6.31	25.42	0.8425	0.4817	MLPR	$[\log_{10}(\rho^{-1})]''$	4.94	12.06	0.9699	0.8270
23	Ridge	$[\log_{10}(\rho^{-1})]''$	6.31	25.42	0.8425	0.4817	MLPR	$[\log_{10}(\rho^{-1})]'$	5.35	12.09	0.9643	0.8252
24	KR	ρ''	6.39	25.51	0.8379	0.4713	MLPR	ρ'	4.96	12.13	0.9694	0.8240

Continued on next page

Table S.15 – Continued from previous page

	Training on 2019–2020 data and testing on 2021–2022 data						Training and testing based on 80%-20% random split of all data					
Rank	Method[1]	Input data	Training %RMSE	Testing %RMSE	Training r^2	Testing r^2	Method[1]	Input data	Training %RMSE	Testing %RMSE	Training r^2	Testing r^2
25	Ridge	ρ''	6.39	25.51	0.8379	0.4713	SVR	$\log_{10}(\rho^{-1})$	9.99	12.16	0.8718	0.8365
26	LL	$[\log_{10}(\rho^{-1})]'$	6.35	25.67	0.8389	0.5169	RFR	148 indices	5.56	12.22	0.9623	0.8249
27	Lasso	$[\log_{10}(\rho^{-1})]'$	6.35	25.68	0.8386	0.5170	PLSR	ρ'	11.25	12.34	0.8333	0.8197
28	PLSR	ρ''	7.04	25.86	0.8005	0.4744	GBR	ρ_{CR}	3.67	12.37	0.9836	0.8221
29	MLPR	148 indices	5.90	26.50	0.8605	0.4766	MLPR	$\log_{10}(\rho^{-1})$	9.51	12.45	0.8813	0.8151
30	BR	148 indices	7.63	26.86	0.7656	0.5126	RFR	ρ_{CR}	5.49	12.53	0.9667	0.8229
31	MLPR	ρ	5.95	26.95	0.8585	0.4977	KNR	148 indices	10.73	12.58	0.8486	0.8110
32	KR	$\log_{10}(\rho^{-1})$	6.73	26.97	0.8175	0.5073	MLPR	ρ''	4.70	12.59	0.9728	0.8104
33	Lasso	$\log_{10}(\rho^{-1})$	6.90	27.29	0.8086	0.5132	BR	148 indices	12.39	12.62	0.7979	0.8130
34	PLSR	$\log_{10}(\rho^{-1})$	6.86	27.32	0.8103	0.4949	RFR	ρ	5.67	12.76	0.9626	0.8075
35	Ridge	$\log_{10}(\rho^{-1})$	6.90	27.37	0.8085	0.5130	PLSR	$[\log_{10}(\rho^{-1})]'$	11.55	12.77	0.8242	0.8066
36	LL	$\log_{10}(\rho^{-1})$	6.89	27.40	0.8091	0.5105	Lasso	148 indices	12.49	12.79	0.7946	0.8073
37	Ridge	ρ	6.85	27.48	0.8112	0.4875	GBR	ρ	7.31	12.83	0.9353	0.8061
38	BR	$\log_{10}(\rho^{-1})$	6.98	27.52	0.8038	0.5171	BR	ρ	10.01	12.85	0.8687	0.8046
39	KR	ρ	6.87	27.55	0.8098	0.4879	LL	ρ'	10.91	12.87	0.8459	0.8062
40	LL	ρ	6.92	27.56	0.8073	0.4883	ABR	ρ_{CR}	10.69	12.87	0.8621	0.8202
41	Lasso	ρ	6.98	27.67	0.8039	0.4842	RFR	$\log_{10}(\rho^{-1})$	6.77	12.88	0.9457	0.8046
42	MLPR	$\log_{10}(\rho^{-1})$	5.90	27.72	0.8604	0.4976	GBR	$\log_{10}(\rho^{-1})$	7.91	12.88	0.9255	0.8062
43	BR	ρ	7.01	27.84	0.8026	0.4925	Lasso	ρ'	10.90	12.88	0.8460	0.8059
44	Ridge	148 indices	7.36	27.89	0.7819	0.5010	KR	ρ	9.69	12.89	0.8769	0.8024
45	Lasso	ρ_{CR}	6.57	27.95	0.8270	0.4684	Ridge	$[\log_{10}(\rho^{-1})]'$	9.37	12.95	0.8866	0.8006
46	LL	ρ_{CR}	6.57	27.97	0.8269	0.4696	Ridge	148 indices	12.86	12.95	0.7825	0.8032
47	BR	ρ_{CR}	6.35	27.97	0.8386	0.5131	PLSR	148 indices	11.45	12.95	0.8272	0.7994
48	Ridge	ρ_{CR}	6.37	28.00	0.8373	0.5108	KR	$[\log_{10}(\rho^{-1})]'$	9.35	12.96	0.8871	0.8003
49	KR	ρ_{CR}	6.38	28.01	0.8371	0.5104	ABR	148 indices	11.03	12.96	0.8414	0.8009
50	PLSR	ρ	7.14	28.25	0.7948	0.4775	Lasso	$[\log_{10}(\rho^{-1})]'$	11.22	13.05	0.8368	0.8027
51	MLPR	ρ_{CR}	4.67	28.35	0.9148	0.3912	LL	$[\log_{10}(\rho^{-1})]'$	11.22	13.05	0.8367	0.8027
52	Lasso	148 indices	7.21	28.36	0.7908	0.4857	ABR	$\log_{10}(\rho^{-1})$	12.59	13.06	0.8153	0.8207
53	LL	148 indices	6.63	28.53	0.8229	0.4685	Ridge	ρ'	9.21	13.07	0.8905	0.7966
54	KR	148 indices	6.71	28.87	0.8187	0.4472	KR	ρ'	9.20	13.07	0.8908	0.7964
55	PLSR	148 indices	6.80	29.05	0.8138	0.4197	GBR	148 indices	3.92	13.12	0.9809	0.7980
56	GBR	$[\log_{10}(\rho^{-1})]'$	3.09	29.12	0.9641	0.4803	Ridge	$[\log_{10}(\rho^{-1})]''$	8.94	13.13	0.8975	0.7946

Continued on next page

	Training on 2019–2020 data and testing on 2021–2022 data						Training and testing based on 80%-20% random split of all data					
Rank	Method[1]	Input data	Training %RMSE	Testing %RMSE	Training r^2	Testing r^2	Method[1]	Input data	Training %RMSE	Testing %RMSE	Training r^2	Testing r^2
57	DTR	$[\log_{10}(\rho^{-1})]''$	7.03	29.20	0.8012	0.4386	KR	$[\log_{10}(\rho^{-1})]''$	8.91	13.14	0.8981	0.7944
58	DTR	$[\log_{10}(\rho^{-1})]'$	7.12	29.24	0.7958	0.4527	SVR	ρ''	2.75	13.16	0.9913	0.8181
59	RFR	ρ'	3.60	29.24	0.9523	0.4355	Ridge	ρ	11.47	13.21	0.8277	0.7968
60	DTR	ρ'	7.97	29.40	0.7438	0.4273	PLSR	ρ	10.81	13.28	0.8460	0.7920
61	RFR	ρ''	3.22	29.47	0.9636	0.4414	ABR	ρ	12.66	13.31	0.8166	0.8157
62	GBR	ρ'	4.34	29.50	0.9269	0.4643	DTR	$[\log_{10}(\rho^{-1})]'$	11.54	13.32	0.8246	0.7882
63	GBR	148 indices	4.81	29.53	0.9091	0.4456	BR	$[\log_{10}(\rho^{-1})]''$	7.74	13.35	0.9226	0.7869
64	RFR	148 indices	4.81	29.54	0.9103	0.4338	SVR	$[\log_{10}(\rho^{-1})]''$	2.81	13.41	0.9909	0.8103
65	GBR	ρ''	2.71	29.57	0.9736	0.4664	LL	ρ	10.04	13.54	0.8678	0.7829
66	DTR	ρ_{CR}	8.38	29.73	0.7175	0.4511	Lasso	$[\log_{10}(\rho^{-1})]''$	8.85	13.62	0.8990	0.7781
67	RFR	$[\log_{10}(\rho^{-1})]''$	3.83	29.74	0.9470	0.4570	BR	$[\log_{10}(\rho^{-1})]'$	8.07	13.63	0.9155	0.7780
68	ABR	ρ'	6.06	29.79	0.8585	0.4602	LL	$[\log_{10}(\rho^{-1})]''$	8.84	13.63	0.8992	0.7780
69	DTR	148 indices	7.73	29.79	0.7595	0.4196	Ridge	ρ''	8.81	13.73	0.9004	0.7745
70	RFR	$[\log_{10}(\rho^{-1})]'$	3.10	29.98	0.9663	0.4549	KR	ρ''	8.77	13.73	0.9013	0.7744
71	RFR	ρ_{CR}	3.80	30.09	0.9474	0.4311	BR	ρ'	7.82	13.92	0.9208	0.7690
72	GBR	ρ_{CR}	4.78	30.12	0.9118	0.4938	BR	ρ''	7.55	13.93	0.9262	0.7687
73	ABR	ρ''	5.90	30.39	0.8666	0.4474	PLSR	$[\log_{10}(\rho^{-1})]''$	10.22	13.99	0.8623	0.7664
74	ABR	148 indices	6.76	30.57	0.8176	0.4167	DTR	ρ''	12.24	14.01	0.8027	0.7663
75	GBR	$[\log_{10}(\rho^{-1})]''$	2.58	30.59	0.9765	0.4504	Lasso	ρ_{CR}	13.49	14.08	0.7627	0.7726
76	PLSR	ρ_{CR}	6.62	30.64	0.8236	0.2909	LL	ρ_{CR}	13.50	14.10	0.7624	0.7718
77	ABR	$[\log_{10}(\rho^{-1})]''$	5.92	30.69	0.8684	0.4429	Lasso	ρ''	8.99	14.12	0.8957	0.7620
78	DTR	ρ''	8.05	30.73	0.7389	0.4068	LL	ρ''	8.98	14.13	0.8958	0.7619
79	ABR	$[\log_{10}(\rho^{-1})]'$	6.14	30.75	0.8550	0.4643	Lasso	ρ	12.44	14.14	0.7966	0.7668
80	ABR	ρ_{CR}	6.48	30.87	0.8375	0.4498	PLSR	ρ''	10.65	14.25	0.8507	0.7574
81	KNR	148 indices	7.96	30.99	0.7506	0.4603	Lasso	$\log_{10}(\rho^{-1})$	12.64	14.28	0.7901	0.7616
82	SVR	148 indices	6.30	31.01	0.8416	0.3742	DTR	$[\log_{10}(\rho^{-1})]''$	12.53	14.34	0.7932	0.7544
83	SVR	$\log_{10}(\rho^{-1})$	5.43	31.23	0.8829	0.4745	KNR	ρ	14.05	14.55	0.7480	0.7772
84	SVR	ρ	5.39	31.45	0.8845	0.4720	BR	ρ_{CR}	10.69	14.79	0.8506	0.7393
85	GBR	$\log_{10}(\rho^{-1})$	4.23	31.56	0.9355	0.5033	DTR	$\log_{10}(\rho^{-1})$	12.06	14.85	0.8083	0.7369
86	KNR	ρ'	7.53	31.59	0.7840	0.3632	KNR	$\log_{10}(\rho^{-1})$	14.23	14.99	0.7425	0.7615
87	RFR	ρ	4.29	31.59	0.9345	0.5317	DTR	ρ_{CR}	13.99	15.06	0.7423	0.7302
88	RFR	$\log_{10}(\rho^{-1})$	3.33	31.63	0.9624	0.5206	DTR	ρ	12.03	15.22	0.8094	0.7237

Continued on next page

Table S.15 – Continued from previous page

Rank	Training on 2019–2020 data and testing on 2021–2022 data						Training and testing based on 80%-20% random split of all data					
	Method[1]	Input data	Training %RMSE	Testing %RMSE	Training r^2	Testing r^2	Method[1]	Input data	Training %RMSE	Testing %RMSE	Training r^2	Testing r^2
89	DTR	$\log_{10}(\rho^{-1})$	7.37	31.68	0.7814	0.5197	DTR	ρ'	12.59	15.37	0.7911	0.7210
90	DTR	ρ	7.37	31.69	0.7814	0.5224	DTR	148 indices	11.19	15.63	0.8350	0.7097
91	GBR	ρ	3.43	31.73	0.9576	0.5085	KR	ρ_{CR}	12.00	15.94	0.8124	0.6974
92	KNR	$[\log_{10}(\rho^{-1})]'$	7.75	32.00	0.7694	0.4231	Ridge	ρ_{CR}	12.00	15.94	0.8122	0.6972
93	ABR	$\log_{10}(\rho^{-1})$	7.38	32.01	0.7947	0.5169	MLPR	ρ_{CR}	8.07	16.25	0.9177	0.6955
94	KNR	ρ''	8.23	32.10	0.7539	0.4129	KNR	ρ_{CR}	13.37	17.59	0.7873	0.6780
95	KNR	$[\log_{10}(\rho^{-1})]''$	8.48	32.33	0.7366	0.4486	Ridge	$\log_{10}(\rho^{-1})$	10.87	18.31	0.8453	0.6352
96	ABR	ρ	7.56	32.35	0.7822	0.5114	KNR	$[\log_{10}(\rho^{-1})]'$	14.79	19.23	0.7522	0.6102
97	KNR	ρ_{CR}	8.70	34.06	0.7188	0.4814	KNR	ρ'	16.30	19.56	0.6583	0.5564
98	SVR	$[\log_{10}(\rho^{-1})]'$	3.03	34.28	0.9649	0.5647	PLSR	ρ_{CR}	13.13	21.77	0.7730	0.5224
99	SVR	ρ_{CR}	3.59	34.61	0.9500	0.4326	BR	$\log_{10}(\rho^{-1})$	9.84	23.44	0.8732	0.5157
100	SVR	ρ'	3.04	34.84	0.9646	0.5143	KNR	ρ''	20.07	24.99	0.6754	0.4469
101	KNR	ρ	9.12	35.80	0.6844	0.4433	KNR	$[\log_{10}(\rho^{-1})]''$	17.81	25.30	0.7602	0.4031
102	KNR	$\log_{10}(\rho^{-1})$	9.60	35.96	0.6606	0.4339	LL	$\log_{10}(\rho^{-1})$	7.96	25.85	0.9171	0.4694
103	SVR	ρ''	2.52	38.54	0.9765	0.4513	KR	$\log_{10}(\rho^{-1})$	8.98	26.23	0.8944	0.4627
104	SVR	$[\log_{10}(\rho^{-1})]''$	2.80	39.03	0.9710	0.4618	GPR	148 indices	0.00	28.75	1.0000	0.1493
105	GPR	148 indices	0.00	39.56	1.0000	0.0000	GPR	ρ	0.00	28.93	1.0000	0.0002
106	GPR	ρ	0.00	39.56	1.0000	0.0000	GPR	ρ'	0.00	28.93	1.0000	0.0000
107	GPR	ρ'	0.00	39.56	1.0000	0.0000	GPR	ρ''	0.00	28.93	1.0000	0.0000
108	GPR	ρ''	0.00	39.56	1.0000	0.0000	GPR	$\log_{10}(\rho^{-1})$	0.00	28.93	1.0000	0.0002
109	GPR	$\log_{10}(\rho^{-1})$	0.00	39.56	1.0000	0.0000	GPR	$[\log_{10}(\rho^{-1})]'$	0.00	28.93	1.0000	0.0000
110	GPR	$[\log_{10}(\rho^{-1})]'$	0.00	39.56	1.0000	0.0000	GPR	$[\log_{10}(\rho^{-1})]''$	0.00	28.93	1.0000	0.0000
111	GPR	$[\log_{10}(\rho^{-1})]''$	0.00	39.56	1.0000	0.0000	GPR	ρ_{CR}	0.00	28.93	1.0000	0.0000
112	GPR	ρ_{CR}	0.00	39.56	1.0000	0.0000	PLSR	$\log_{10}(\rho^{-1})$	10.04	36.07	0.8673	0.3240

[1] AdaBoostRegressor, ABR; BayesianRidge, BR; DecisionTreeRegressor, DTR; GaussianProcessRegressor, GPR; GradientBoostingRegressor, GBR; KernelRidge, KR; KNeighborsRegressor, KNR; LassoLars, LL; MLPRegressor, MLPR; PLSRegression, PLSR; RandomForestRegressor, RFR; and support vector regression, SVR.

Table S.16: Goodness-of-fit statistics, including root mean squared errors (%RMSE) and coefficients of determination (r^2) between measured and modeled area-basis cotton leaf chlorophyll a (Chl a, μg cm^{-2}) for training and testing of 14 machine learning methods from Python's "scikit-learn" package with 8 input data sets derived from leaf spectral reflectance (ρ). The 8 input data sets included spectral reflectance (ρ); the first and second derivatives of reflectance (ρ' and ρ'', respectively); the base-10 logarithm of the inverse of reflectance ($\log_{10}\rho^{-1}$) and its first and second derivatives [$(\log_{10}\rho^{-1})'$ and $(\log_{10}\rho^{-1})''$, respectively]; continuum-removed reflectance (ρ_{CR}); and the set of 148 spectral indices from Table S.1. Models were trained and tested by experiment using data from the 2019–2020 and 2021–2022 cotton field studies at Maricopa, Arizona, USA, respectively. Models were also trained and tested using an 80% and 20% random split of all from both experiments. Results are ranked according to the %RMSE of model testing.

	Training on 2019–2020 data and testing on 2021–2022 data						Training and testing based on 80%-20% random split of all data					
Rank	Method[1]	Input data	Training %RMSE	Testing %RMSE	Training r^2	Testing r^2	Method[1]	Input data	Training %RMSE	Testing %RMSE	Training r^2	Testing r^2
1	LL	$[\log_{10}(\rho^{-1})]''$	6.12	21.55	0.8436	0.5089	MLPR	148 indices	9.34	10.62	0.8623	0.8397
2	Lasso	$[\log_{10}(\rho^{-1})]''$	6.12	21.55	0.8436	0.5089	GBR	$[\log_{10}(\rho^{-1})]''$	2.54	10.71	0.9906	0.8392
3	MLPR	ρ'	3.45	21.55	0.9523	0.4521	GBR	ρ''	2.40	11.00	0.9915	0.8286
4	PLSR	$[\log_{10}(\rho^{-1})]'$	6.12	21.93	0.8416	0.4820	RFR	$[\log_{10}(\rho^{-1})]'$	5.47	11.03	0.9584	0.8304
5	MLPR	$[\log_{10}(\rho^{-1})]'$	3.49	22.10	0.9513	0.4543	GBR	$[\log_{10}(\rho^{-1})]'$	3.49	11.06	0.9821	0.8258
6	BR	$[\log_{10}(\rho^{-1})]''$	5.54	22.11	0.8722	0.4815	SVR	148 indices	9.17	11.17	0.8678	0.8267
7	Lasso	ρ''	6.36	22.25	0.8309	0.4698	RFR	$[\log_{10}(\rho^{-1})]''$	4.62	11.19	0.9721	0.8260
8	LL	ρ''	6.37	22.25	0.8309	0.4698	MLPR	$\log_{10}(\rho^{-1})$	9.67	11.23	0.8522	0.8224
9	BR	ρ''	5.64	22.26	0.8672	0.4640	MLPR	ρ	9.06	11.25	0.8706	0.8209
10	LL	ρ'	5.85	22.26	0.8569	0.4696	ABR	$[\log_{10}(\rho^{-1})]'$	9.16	11.31	0.8753	0.8278
11	Lasso	ρ'	5.85	22.27	0.8567	0.4694	SVR	$[\log_{10}(\rho^{-1})]'$	2.84	11.32	0.9885	0.8254
12	MLPR	ρ''	3.62	22.28	0.9480	0.4667	SVR	ρ'	2.50	11.32	0.9910	0.8202
13	MLPR	$[\log_{10}(\rho^{-1})]''$	3.87	22.29	0.9406	0.4944	SVR	ρ_{CR}	4.55	11.52	0.9691	0.8113
14	Ridge	$[\log_{10}(\rho^{-1})]''$	5.82	22.30	0.8596	0.4840	RFR	ρ'	4.89	11.58	0.9669	0.8094
15	KR	$[\log_{10}(\rho^{-1})]''$	5.82	22.30	0.8596	0.4840	RFR	ρ''	5.67	11.64	0.9550	0.8115
16	BR	$[\log_{10}(\rho^{-1})]'$	5.54	22.40	0.8718	0.4778	GBR	ρ'	4.03	11.66	0.9755	0.8045
17	KR	$[\log_{10}(\rho^{-1})]'$	5.66	22.41	0.8663	0.4821	SVR	ρ	9.19	11.78	0.8679	0.8081
18	Ridge	$[\log_{10}(\rho^{-1})]'$	5.66	22.41	0.8661	0.4823	RFR	148 indices	5.18	11.81	0.9617	0.8028
19	BR	ρ'	5.62	22.43	0.8679	0.4539	GBR	$\log_{10}(\rho^{-1})$	5.32	11.85	0.9585	0.8008
20	Ridge	ρ'	5.76	22.46	0.8615	0.4558	SVR	$\log_{10}(\rho^{-1})$	9.23	11.88	0.8665	0.8050
21	KR	ρ'	5.76	22.46	0.8615	0.4558	LL	148 indices	10.88	11.89	0.8120	0.7975
22	KR	ρ''	5.93	22.48	0.8540	0.4649	GBR	ρ_{CR}	5.86	11.99	0.9489	0.7948
23	Ridge	ρ''	5.93	22.48	0.8538	0.4649	KR	148 indices	11.08	12.01	0.8051	0.7940
24	Lasso	$[\log_{10}(\rho^{-1})]'$	5.83	22.49	0.8576	0.4939	MLPR	$[\log_{10}(\rho^{-1})]''$	4.94	12.05	0.9639	0.7919

Continued on next page

Table S.16 – Continued from previous page

Rank	Training on 2019–2020 data and testing on 2021–2022 data						Training and testing based on 80%-20% random split of all data					
	Method[1]	Input data	Training %RMSE	Testing %RMSE	Training r^2	Testing r^2	Method[1]	Input data	Training %RMSE	Testing %RMSE	Training r^2	Testing r^2
25	LL	$[\log_{10}(\rho^{-1})]'$	5.85	22.50	0.8570	0.4944	ABR	ρ'	9.22	12.06	0.8779	0.8025
26	PLSR	$[\log_{10}(\rho^{-1})]''$	6.42	22.61	0.8262	0.4918	GBR	ρ	5.05	12.08	0.9629	0.7927
27	MLPR	148 indices	5.53	22.75	0.8719	0.4974	RFR	ρ_{CR}	4.91	12.11	0.9680	0.7952
28	PLSR	ρ'	6.13	22.83	0.8414	0.4430	KNR	148 indices	10.60	12.14	0.8222	0.7889
29	PLSR	ρ''	6.34	22.92	0.8301	0.4544	RFR	$\log_{10}(\rho^{-1})$	6.15	12.20	0.9470	0.7899
30	BR	148 indices	7.40	23.40	0.7686	0.5112	GBR	148 indices	5.69	12.21	0.9513	0.7869
31	Ridge	148 indices	7.39	23.44	0.7695	0.5108	MLPR	$[\log_{10}(\rho^{-1})]'$	4.55	12.33	0.9691	0.7812
32	KR	$\log_{10}(\rho^{-1})$	6.06	23.72	0.8453	0.4564	MLPR	ρ'	4.17	12.35	0.9739	0.7809
33	KR	ρ	6.23	23.90	0.8362	0.4638	PLSR	ρ'	10.58	12.35	0.8221	0.7807
34	Ridge	ρ	6.24	23.93	0.8356	0.4636	RFR	ρ	6.06	12.37	0.9482	0.7826
35	Ridge	$\log_{10}(\rho^{-1})$	5.66	24.08	0.8652	0.3886	ABR	$[\log_{10}(\rho^{-1})]''$	9.55	12.37	0.8761	0.8098
36	LL	$\log_{10}(\rho^{-1})$	6.22	24.21	0.8368	0.4703	PLSR	$[\log_{10}(\rho^{-1})]'$	11.25	12.39	0.7987	0.7821
37	PLSR	$\log_{10}(\rho^{-1})$	6.41	24.36	0.8265	0.4519	ABR	ρ_{CR}	10.01	12.43	0.8540	0.7931
38	PLSR	ρ	6.46	24.43	0.8236	0.4366	BR	148 indices	12.00	12.45	0.7714	0.7810
39	Lasso	148 indices	6.98	24.68	0.7946	0.4960	Ridge	148 indices	12.02	12.47	0.7705	0.7806
40	Lasso	$\log_{10}(\rho^{-1})$	6.65	24.77	0.8132	0.4939	Lasso	148 indices	11.90	12.50	0.7752	0.7781
41	LL	ρ	6.46	24.84	0.8241	0.4599	ABR	ρ''	9.37	12.59	0.8791	0.7930
42	BR	$\log_{10}(\rho^{-1})$	6.65	24.89	0.8136	0.4825	ABR	148 indices	10.63	12.60	0.8260	0.7799
43	LL	148 indices	6.38	24.91	0.8281	0.4473	ABR	ρ	11.31	12.61	0.8080	0.7892
44	Lasso	ρ	6.72	25.23	0.8098	0.4726	PLSR	148 indices	11.04	12.68	0.8064	0.7687
45	BR	ρ	6.71	25.27	0.8103	0.4640	MLPR	ρ_{CR}	7.21	12.73	0.9206	0.7667
46	MLPR	ρ	6.17	25.38	0.8399	0.4854	MLPR	ρ''	3.15	12.82	0.9854	0.7636
47	MLPR	$\log_{10}(\rho^{-1})$	5.62	25.43	0.8676	0.4543	BR	ρ	9.74	12.83	0.8501	0.7645
48	PLSR	ρ_{CR}	6.36	25.54	0.8294	0.5170	Lasso	$[\log_{10}(\rho^{-1})]'$	10.02	12.84	0.8431	0.7658
49	DTR	148 indices	7.41	25.62	0.7679	0.4199	LL	$[\log_{10}(\rho^{-1})]'$	10.00	12.84	0.8438	0.7656
50	GBR	ρ''	2.67	25.63	0.9722	0.4556	Ridge	ρ	10.51	12.85	0.8253	0.7656
51	KR	148 indices	6.52	25.68	0.8206	0.4358	PLSR	ρ	10.33	12.89	0.8303	0.7630
52	PLSR	148 indices	6.54	25.69	0.8193	0.4107	BR	ρ_{CR}	9.96	12.90	0.8435	0.7620
53	RFR	ρ''	2.89	25.81	0.9701	0.4299	KR	ρ	9.40	12.91	0.8602	0.7609
54	BR	ρ_{CR}	6.09	25.83	0.8442	0.4686	Ridge	$[\log_{10}(\rho^{-1})]''$	8.52	12.94	0.8879	0.7598
55	KR	ρ_{CR}	6.09	25.83	0.8444	0.4681	KR	$[\log_{10}(\rho^{-1})]''$	8.51	12.94	0.8882	0.7597
56	Ridge	ρ_{CR}	6.09	25.84	0.8445	0.4678	SVR	ρ''	2.32	12.97	0.9926	0.7860

Continued on next page

Table S.16 – Continued from previous page

	Training on 2019–2020 data and testing on 2021–2022 data						Training and testing based on 80%-20% random split of all data					
Rank	Method[1]	Input data	Training %RMSE	Testing %RMSE	Training r^2	Testing r^2	Method[1]	Input data	Training %RMSE	Testing %RMSE	Training r^2	Testing r^2
57	DTR	ρ''	7.40	25.90	0.7688	0.4146	Ridge	$[\log_{10}(\rho^{-1})]'$	8.90	13.02	0.8765	0.7566
58	GBR	148 indices	5.41	25.97	0.8804	0.4490	KR	$[\log_{10}(\rho^{-1})]'$	8.90	13.02	0.8767	0.7564
59	RFR	$[\log_{10}(\rho^{-1})]''$	3.54	26.07	0.9533	0.4386	ABR	$\log_{10}(\rho^{-1})$	11.53	13.05	0.8095	0.7837
60	RFR	ρ'	3.18	26.09	0.9629	0.4199	LL	ρ'	9.82	13.15	0.8497	0.7523
61	DTR	$[\log_{10}(\rho^{-1})]''$	7.20	26.09	0.7810	0.4166	Lasso	ρ'	9.80	13.16	0.8500	0.7517
62	Lasso	ρ_{CR}	6.33	26.09	0.8315	0.4143	KR	ρ_{CR}	11.04	13.18	0.8083	0.7526
63	LL	ρ_{CR}	6.32	26.13	0.8320	0.4123	Ridge	ρ_{CR}	11.05	13.19	0.8079	0.7525
64	GBR	ρ'	3.90	26.19	0.9396	0.4285	SVR	$[\log_{10}(\rho^{-1})]''$	2.32	13.23	0.9926	0.7761
65	RFR	148 indices	3.63	26.19	0.9490	0.4206	PLSR	$[\log_{10}(\rho^{-1})]''$	10.74	13.27	0.8167	0.7472
66	GBR	$[\log_{10}(\rho^{-1})]''$	3.31	26.21	0.9578	0.4224	Ridge	ρ'	8.64	13.30	0.8839	0.7453
67	KNR	148 indices	7.25	26.38	0.7820	0.4557	KR	ρ'	8.63	13.31	0.8842	0.7450
68	ABR	148 indices	6.56	26.57	0.8205	0.4323	BR	$[\log_{10}(\rho^{-1})]''$	7.25	13.32	0.9180	0.7452
69	ABR	ρ'	6.07	26.59	0.8479	0.4328	Lasso	$[\log_{10}(\rho^{-1})]''$	8.14	13.33	0.8968	0.7444
70	GBR	$[\log_{10}(\rho^{-1})]'$	2.36	26.60	0.9780	0.4301	LL	$[\log_{10}(\rho^{-1})]''$	8.15	13.33	0.8967	0.7443
71	RFR	$[\log_{10}(\rho^{-1})]'$	2.93	26.79	0.9692	0.4489	Lasso	ρ_{CR}	12.45	13.42	0.7559	0.7480
72	ABR	ρ''	5.64	26.88	0.8729	0.4277	LL	ρ_{CR}	12.43	13.45	0.7568	0.7464
73	DTR	ρ_{CR}	8.08	26.95	0.7243	0.4329	LL	ρ	9.64	13.49	0.8529	0.7387
74	ABR	$[\log_{10}(\rho^{-1})]''$	5.70	26.97	0.8716	0.4287	Ridge	ρ''	8.28	13.59	0.8940	0.7344
75	GBR	ρ_{CR}	3.19	26.98	0.9603	0.4356	KR	ρ''	8.26	13.59	0.8945	0.7343
76	DTR	ρ'	8.06	27.04	0.7259	0.4057	DTR	ρ''	11.65	13.59	0.7843	0.7352
77	ABR	ρ_{CR}	6.42	27.07	0.8330	0.4454	PLSR	ρ''	10.64	13.61	0.8202	0.7340
78	RFR	ρ_{CR}	3.16	27.12	0.9631	0.4179	Lasso	ρ	11.68	13.62	0.7838	0.7367
79	ABR	$[\log_{10}(\rho^{-1})]'$	6.05	27.29	0.8508	0.4488	DTR	ρ	12.76	13.80	0.7414	0.7267
80	MLPR	ρ_{CR}	4.39	27.43	0.9211	0.3084	KNR	ρ	12.94	13.81	0.7420	0.7471
81	SVR	148 indices	5.89	27.85	0.8545	0.3409	Lasso	$\log_{10}(\rho^{-1})$	11.74	13.81	0.7813	0.7277
82	RFR	ρ	3.81	27.92	0.9462	0.4933	DTR	$\log_{10}(\rho^{-1})$	12.76	13.83	0.7414	0.7253
83	RFR	$\log_{10}(\rho^{-1})$	4.13	28.01	0.9367	0.4900	DTR	148 indices	11.00	13.90	0.8078	0.7232
84	KNR	ρ'	7.06	28.02	0.8005	0.3482	LL	ρ''	8.21	13.92	0.8951	0.7222
85	DTR	$[\log_{10}(\rho^{-1})]'$	7.65	28.19	0.7530	0.4128	Lasso	ρ''	8.19	13.93	0.8957	0.7220
86	GBR	ρ	4.15	28.28	0.9350	0.4824	BR	ρ''	7.02	13.97	0.9232	0.7215
87	GBR	$\log_{10}(\rho^{-1})$	3.73	28.29	0.9474	0.4768	BR	$[\log_{10}(\rho^{-1})]'$	7.56	14.04	0.9106	0.7173
88	KNR	ρ''	8.14	28.43	0.7521	0.4027	KNR	$\log_{10}(\rho^{-1})$	13.29	14.21	0.7302	0.7364

Continued on next page

Table S.16 – Continued from previous page

	Training on 2019–2020 data and testing on 2021–2022 data						Training and testing based on 80%-20% random split of all data					
Rank	Method[1]	Input data	Training %RMSE	Testing %RMSE	Training r^2	Testing r^2	Method[1]	Input data	Training %RMSE	Testing %RMSE	Training r^2	Testing r^2
89	KNR	$[\log_{10}(\rho^{-1})]'$	7.79	28.44	0.7606	0.4193	BR	ρ'	7.27	14.43	0.9173	0.7033
90	KNR	$[\log_{10}(\rho^{-1})]''$	8.19	28.52	0.7442	0.4571	DTR	$[\log_{10}(\rho^{-1})]'$	10.46	14.43	0.8262	0.7009
91	SVR	$\log_{10}(\rho^{-1})$	5.20	28.53	0.8871	0.4585	DTR	ρ_{CR}	11.43	14.80	0.7923	0.6865
92	ABR	$\log_{10}(\rho^{-1})$	7.38	28.57	0.7865	0.5085	DTR	$[\log_{10}(\rho^{-1})]''$	11.42	14.91	0.7925	0.6834
93	ABR	ρ	7.27	28.57	0.7919	0.5040	DTR	ρ'	11.13	15.26	0.8032	0.6805
94	SVR	ρ	5.23	28.76	0.8860	0.4575	KNR	ρ_{CR}	11.71	16.22	0.7990	0.6512
95	DTR	$\log_{10}(\rho^{-1})$	7.40	29.46	0.7687	0.4330	KNR	$[\log_{10}(\rho^{-1})]'$	13.00	17.22	0.7685	0.6126
96	KNR	ρ_{CR}	8.27	29.68	0.7351	0.4804	PLSR	ρ_{CR}	12.17	17.50	0.7648	0.5918
97	DTR	ρ	7.83	30.00	0.7414	0.4299	KNR	ρ'	14.93	17.85	0.6523	0.5471
98	SVR	$[\log_{10}(\rho^{-1})]'$	2.44	31.27	0.9763	0.5624	KNR	ρ''	17.35	21.59	0.6825	0.4697
99	SVR	ρ_{CR}	3.66	31.56	0.9457	0.4231	KNR	$[\log_{10}(\rho^{-1})]''$	17.46	21.80	0.6762	0.4524
100	SVR	ρ'	2.39	31.65	0.9772	0.5135	BR	$\log_{10}(\rho^{-1})$	9.66	21.95	0.8524	0.4769
101	KNR	ρ	9.22	32.64	0.6652	0.3697	Ridge	$\log_{10}(\rho^{-1})$	9.64	22.10	0.8532	0.4735
102	KNR	$\log_{10}(\rho^{-1})$	9.50	32.72	0.6517	0.3699	LL	$\log_{10}(\rho^{-1})$	7.73	25.60	0.9056	0.3990
103	SVR	ρ''	1.77	35.21	0.9882	0.4488	KR	$\log_{10}(\rho^{-1})$	8.89	25.88	0.8751	0.3967
104	SVR	$[\log_{10}(\rho^{-1})]''$	1.97	35.66	0.9854	0.4606	GPR	148 indices	0.00	26.21	1.0000	0.1291
105	GPR	148 indices	0.00	36.00	1.0000	0.0005	GPR	ρ	0.00	26.36	1.0000	0.0007
106	GPR	ρ	0.00	36.00	1.0000	0.0000	GPR	ρ'	0.00	26.36	1.0000	0.0000
107	GPR	ρ'	0.00	36.00	1.0000	0.0000	GPR	ρ''	0.00	26.36	1.0000	0.0000
108	GPR	ρ''	0.00	36.00	1.0000	0.0000	GPR	$\log_{10}(\rho^{-1})$	0.00	26.36	1.0000	0.0007
109	GPR	$\log_{10}(\rho^{-1})$	0.00	36.00	1.0000	0.0000	GPR	$[\log_{10}(\rho^{-1})]'$	0.00	26.36	1.0000	0.0000
110	GPR	$[\log_{10}(\rho^{-1})]'$	0.00	36.00	1.0000	0.0000	GPR	$[\log_{10}(\rho^{-1})]''$	0.00	26.36	1.0000	0.0000
111	GPR	$[\log_{10}(\rho^{-1})]''$	0.00	36.00	1.0000	0.0000	GPR	ρ_{CR}	0.00	26.36	1.0000	0.0000
112	GPR	ρ_{CR}	0.00	36.00	1.0000	0.0000	PLSR	$\log_{10}(\rho^{-1})$	9.53	37.22	0.8557	0.2464

[1] AdaBoostRegressor, ABR; BayesianRidge, BR; DecisionTreeRegressor, DTR; GaussianProcessRegressor, GPR; GradientBoostingRegressor, GBR; KernelRidge, KR; KNeighborsRegressor, KNR; LassoLars, LL; MLPRegressor, MLPR; PLSRegression, PLSR; RandomForestRegressor, RFR; and support vector regression, SVR.

Table S.17: Goodness-of-fit statistics, including root mean squared errors (%RMSE) and coefficients of determination (r^2) between measured and modeled area-basis cotton leaf chlorophyll b (Chl b, μg cm^{-2}) for training and testing of 14 machine learning methods from Python's "scikit-learn" package with 8 input data sets derived from leaf spectral reflectance (ρ). The 8 input data sets included spectral reflectance (ρ); the first and second derivatives of reflectance (ρ' and ρ'', respectively); the base-10 logarithm of the inverse of reflectance ($\log_{10}\rho^{-1}$) and its first and second derivatives [$(\log_{10}\rho^{-1})'$ and $(\log_{10}\rho^{-1})''$, respectively]; continuum-removed reflectance (ρ_{CR}); and the set of 148 spectral indices from Table S.1. Models were trained and tested by experiment using data from the 2019–2020 and 2021–2022 cotton field studies at Maricopa, Arizona, USA, respectively. Models were also trained and tested using an 80% and 20% random split of all from both experiments. Results are ranked according to the %RMSE of model testing.

	Training on 2019–2020 data and testing on 2021–2022 data						Training and testing based on 80%-20% random split of all data					
Rank	Method[1]	Input data	Training %RMSE	Testing %RMSE	Training r^2	Testing r^2	Method[1]	Input data	Training %RMSE	Testing %RMSE	Training r^2	Testing r^2
1	MLPR	$[\log_{10}(\rho^{-1})]'$	7.07	41.35	0.9295	0.3138	GBR	$[\log_{10}(\rho^{-1})]'$	5.17	18.55	0.9885	0.8633
2	MLPR	$\log_{10}(\rho^{-1})$	13.02	41.55	0.7364	0.3023	RFR	ρ''	9.60	18.94	0.9636	0.8603
3	MLPR	ρ'	8.01	42.27	0.9085	0.3413	GBR	ρ''	6.55	19.16	0.9820	0.8512
4	Lasso	ρ	14.99	42.68	0.6493	0.3377	RFR	$[\log_{10}(\rho^{-1})]'$	9.84	19.26	0.9610	0.8607
5	Lasso	$\log_{10}(\rho^{-1})$	15.16	42.98	0.6412	0.3499	MLPR	148 indices	16.63	19.40	0.8742	0.8491
6	PLSR	$[\log_{10}(\rho^{-1})]''$	15.10	43.32	0.6435	0.3824	RFR	$[\log_{10}(\rho^{-1})]''$	8.30	19.67	0.9750	0.8542
7	MLPR	ρ''	6.49	43.42	0.9430	0.2997	GBR	$[\log_{10}(\rho^{-1})]''$	4.13	19.71	0.9926	0.8442
8	LL	ρ	14.87	43.46	0.6546	0.3347	MLPR	ρ	17.41	19.85	0.8618	0.8506
9	MLPR	$[\log_{10}(\rho^{-1})]''$	5.91	43.64	0.9532	0.2798	SVR	148 indices	17.62	19.98	0.8595	0.8467
10	LL	$\log_{10}(\rho^{-1})$	15.03	43.77	0.6473	0.3474	GBR	ρ'	7.99	20.19	0.9720	0.8354
11	MLPR	ρ	13.74	43.91	0.7067	0.3386	RFR	ρ'	11.22	20.52	0.9465	0.8311
12	PLSR	ρ'	14.49	44.23	0.6719	0.3891	MLPR	$\log_{10}(\rho^{-1})$	17.52	20.54	0.8603	0.8348
13	KR	ρ	14.68	44.56	0.6636	0.3082	GBR	148 indices	8.35	20.60	0.9705	0.8282
14	Ridge	ρ	14.70	44.57	0.6627	0.3089	MLPR	$[\log_{10}(\rho^{-1})]'$	9.99	20.73	0.9567	0.8261
15	KR	$\log_{10}(\rho^{-1})$	14.72	44.60	0.6617	0.3088	ABR	ρ'	17.72	20.82	0.8835	0.8451
16	Ridge	$\log_{10}(\rho^{-1})$	14.77	44.65	0.6594	0.3109	SVR	ρ'	7.14	20.88	0.9783	0.8294
17	PLSR	$[\log_{10}(\rho^{-1})]'$	14.73	44.68	0.6608	0.3928	SVR	$[\log_{10}(\rho^{-1})]'$	7.19	20.93	0.9782	0.8342
18	PLSR	ρ''	14.49	44.71	0.6715	0.3647	ABR	$[\log_{10}(\rho^{-1})]'$	17.91	20.95	0.8860	0.8596
19	BR	ρ	15.12	44.73	0.6431	0.3308	GBR	ρ	7.66	20.99	0.9756	0.8340
20	KR	ρ'	13.41	44.77	0.7223	0.3859	MLPR	ρ'	9.64	21.04	0.9598	0.8201
21	Ridge	ρ'	13.41	44.77	0.7221	0.3861	PLSR	148 indices	21.16	21.16	0.7947	0.8217
22	BR	ρ'	13.58	44.83	0.7149	0.3906	LL	148 indices	20.44	21.20	0.8085	0.8204
23	Lasso	$[\log_{10}(\rho^{-1})]'$	14.29	44.88	0.6825	0.4008	RFR	148 indices	12.73	21.37	0.9312	0.8187
24	LL	$[\log_{10}(\rho^{-1})]'$	14.29	44.89	0.6828	0.4003	RFR	ρ_{CR}	10.53	21.51	0.9587	0.8350

Continued on next page

Table S.17 – Continued from previous page

Rank	Training on 2019–2020 data and testing on 2021–2022 data						Training and testing based on 80%-20% random split of all data					
	Method[1]	Input data	Training %RMSE	Testing %RMSE	Training r^2	Testing r^2	Method[1]	Input data	Training %RMSE	Testing %RMSE	Training r^2	Testing r^2
25	BR	$\log_{10}(\rho^{-1})$	15.28	44.91	0.6359	0.3456	MLPR	$[\log_{10}(\rho^{-1})]''$	9.30	21.60	0.9631	0.8137
26	PLSR	ρ	14.77	44.94	0.6591	0.2813	GBR	ρ_{CR}	7.28	21.60	0.9778	0.8214
27	KR	$[\log_{10}(\rho^{-1})]'$	13.51	44.98	0.7179	0.3760	SVR	ρ_{CR}	9.28	21.67	0.9620	0.8160
28	Ridge	$[\log_{10}(\rho^{-1})]'$	13.52	44.99	0.7175	0.3762	KR	148 indices	21.71	21.69	0.7840	0.8130
29	LL	ρ'	14.33	44.99	0.6812	0.4125	SVR	ρ	17.43	21.91	0.8645	0.8224
30	Lasso	ρ'	14.33	44.99	0.6812	0.4126	KR	ρ	17.36	22.09	0.8625	0.8037
31	BR	$[\log_{10}(\rho^{-1})]'$	13.69	45.06	0.7099	0.3814	SVR	$\log_{10}(\rho^{-1})$	17.60	22.14	0.8623	0.8205
32	PLSR	$\log_{10}(\rho^{-1})$	14.83	45.20	0.6560	0.2824	BR	148 indices	22.30	22.21	0.7721	0.8033
33	BR	$[\log_{10}(\rho^{-1})]''$	13.08	45.47	0.7380	0.3003	Ridge	ρ'	16.16	22.39	0.8831	0.8014
34	Ridge	$[\log_{10}(\rho^{-1})]''$	13.34	45.49	0.7278	0.3086	KR	ρ'	16.14	22.39	0.8833	0.8013
35	KR	$[\log_{10}(\rho^{-1})]''$	13.34	45.49	0.7277	0.3086	ABR	ρ''	17.86	22.40	0.8878	0.8222
36	KR	ρ''	13.39	45.63	0.7254	0.3356	GBR	$\log_{10}(\rho^{-1})$	7.10	22.41	0.9796	0.8027
37	Ridge	ρ''	13.40	45.63	0.7250	0.3359	BR	ρ	18.57	22.42	0.8427	0.8004
38	BR	ρ''	13.11	45.63	0.7367	0.3255	Lasso	148 indices	22.53	22.49	0.7672	0.7979
39	GBR	ρ'	7.81	45.68	0.9146	0.3269	KNR	148 indices	20.53	22.52	0.8072	0.7979
40	Lasso	$[\log_{10}(\rho^{-1})]''$	14.19	46.42	0.6904	0.3106	MLPR	ρ''	11.37	22.68	0.9446	0.7915
41	LL	$[\log_{10}(\rho^{-1})]''$	14.19	46.42	0.6905	0.3106	Ridge	$[\log_{10}(\rho^{-1})]'$	16.43	22.72	0.8792	0.7950
42	GBR	$[\log_{10}(\rho^{-1})]'$	2.50	46.56	0.9922	0.2892	KR	$[\log_{10}(\rho^{-1})]'$	16.36	22.73	0.8801	0.7946
43	LL	ρ''	14.33	46.62	0.6843	0.3411	RFR	ρ	13.64	22.79	0.9248	0.8009
44	Lasso	ρ''	14.33	46.62	0.6842	0.3412	BR	ρ'	14.56	22.83	0.9046	0.7900
45	BR	148 indices	15.86	47.21	0.6072	0.3573	RFR	$\log_{10}(\rho^{-1})$	14.78	22.84	0.9102	0.7985
46	Ridge	148 indices	15.90	47.27	0.6051	0.3563	ABR	$[\log_{10}(\rho^{-1})]''$	17.74	22.86	0.8889	0.8173
47	Lasso	148 indices	15.40	47.36	0.6294	0.3176	SVR	ρ''	5.05	22.88	0.9895	0.8050
48	MLPR	148 indices	13.35	47.87	0.7240	0.2945	BR	$[\log_{10}(\rho^{-1})]'$	14.97	23.14	0.8991	0.7843
49	GBR	$[\log_{10}(\rho^{-1})]''$	5.89	48.06	0.9535	0.2934	Ridge	ρ	19.93	23.15	0.8189	0.7897
50	SVR	$\log_{10}(\rho^{-1})$	13.43	48.26	0.7211	0.3121	PLSR	ρ	19.65	23.26	0.8229	0.7850
51	RFR	$[\log_{10}(\rho^{-1})]'$	6.67	48.28	0.9455	0.3495	PLSR	ρ'	17.88	23.38	0.8534	0.7817
52	GBR	ρ''	3.30	48.42	0.9860	0.3455	SVR	$[\log_{10}(\rho^{-1})]''$	5.05	23.44	0.9895	0.7964
53	SVR	ρ	13.36	48.48	0.7242	0.2898	LL	ρ	18.31	23.47	0.8470	0.7794
54	MLPR	ρ_{CR}	9.14	48.55	0.8759	0.0847	ABR	ρ_{CR}	20.35	23.50	0.8561	0.8113
55	ABR	$[\log_{10}(\rho^{-1})]'$	11.87	48.74	0.8175	0.3373	Ridge	148 indices	23.73	23.50	0.7422	0.7795
56	LL	148 indices	14.95	48.76	0.6507	0.2376	LL	ρ'	15.73	23.70	0.8885	0.7762

Continued on next page

Table S.17 – Continued from previous page

Rank	Training on 2019–2020 data and testing on 2021–2022 data						Training and testing based on 80%-20% random split of all data					
	Method[1]	Input data	Training %RMSE	Testing %RMSE	Training r^2	Testing r^2	Method[1]	Input data	Training %RMSE	Testing %RMSE	Training r^2	Testing r^2
57	ABR	ρ'	11.93	48.77	0.8253	0.3651	PLSR	$[\log_{10}(\rho^{-1})]'$	18.20	23.74	0.8480	0.7737
58	RFR	ρ'	9.02	48.77	0.8886	0.3499	Lasso	ρ'	15.45	23.76	0.8924	0.7745
59	RFR	ρ''	7.23	49.07	0.9383	0.3811	KR	$[\log_{10}(\rho^{-1})]''$	16.83	23.81	0.8742	0.7750
60	GBR	148 indices	8.95	49.15	0.8873	0.2911	Ridge	$[\log_{10}(\rho^{-1})]''$	16.86	23.82	0.8738	0.7750
61	RFR	148 indices	8.94	49.68	0.8933	0.3505	BR	$[\log_{10}(\rho^{-1})]''$	14.95	23.83	0.8999	0.7710
62	RFR	$[\log_{10}(\rho^{-1})]''$	8.01	49.79	0.9209	0.3210	BR	ρ''	14.92	23.98	0.9001	0.7669
63	GBR	ρ_{CR}	3.90	49.92	0.9796	0.3333	KR	ρ''	17.15	24.07	0.8693	0.7683
64	KR	148 indices	14.61	50.18	0.6662	0.1967	Ridge	ρ''	17.20	24.08	0.8684	0.7682
65	GBR	ρ	8.40	50.34	0.9028	0.2783	LL	$[\log_{10}(\rho^{-1})]'$	16.17	24.24	0.8822	0.7631
66	ABR	$\log_{10}(\rho^{-1})$	14.66	50.43	0.7368	0.3518	Lasso	$[\log_{10}(\rho^{-1})]'$	16.11	24.25	0.8829	0.7628
67	ABR	ρ''	11.96	50.47	0.8280	0.3772	DTR	ρ''	22.67	24.28	0.7644	0.7580
68	ABR	ρ	14.68	50.54	0.7284	0.3414	ABR	148 indices	22.16	24.49	0.8019	0.7742
69	RFR	ρ_{CR}	6.46	50.71	0.9517	0.3411	Lasso	$[\log_{10}(\rho^{-1})]''$	16.60	24.65	0.8767	0.7561
70	DTR	ρ'	16.03	50.84	0.5984	0.3081	LL	$[\log_{10}(\rho^{-1})]''$	16.76	24.67	0.8745	0.7559
71	ABR	$[\log_{10}(\rho^{-1})]''$	12.21	50.89	0.8154	0.3096	ABR	$\log_{10}(\rho^{-1})$	25.49	24.80	0.7303	0.7848
72	SVR	$[\log_{10}(\rho^{-1})]'$	5.80	51.08	0.9499	0.3464	DTR	148 indices	23.75	25.11	0.7413	0.7420
73	DTR	148 indices	17.01	51.09	0.5475	0.3284	ABR	ρ	25.37	25.21	0.7483	0.7864
74	GBR	$\log_{10}(\rho^{-1})$	8.65	51.17	0.9020	0.2581	LL	ρ''	19.30	25.38	0.8334	0.7441
75	PLSR	148 indices	14.79	51.29	0.6579	0.1557	Lasso	ρ''	19.29	25.38	0.8336	0.7440
76	DTR	$[\log_{10}(\rho^{-1})]'$	15.60	51.45	0.6196	0.1886	Ridge	$\log_{10}(\rho^{-1})$	22.69	25.41	0.7650	0.7437
77	DTR	ρ''	16.89	51.83	0.5540	0.3015	Lasso	ρ	22.46	25.46	0.7693	0.7453
78	RFR	ρ	9.82	51.87	0.8711	0.3136	DTR	$[\log_{10}(\rho^{-1})]'$	21.00	25.59	0.7977	0.7342
79	ABR	148 indices	15.56	51.95	0.6431	0.3320	PLSR	$[\log_{10}(\rho^{-1})]''$	19.03	25.80	0.8339	0.7317
80	RFR	$\log_{10}(\rho^{-1})$	9.60	52.01	0.8764	0.3121	Lasso	$\log_{10}(\rho^{-1})$	22.79	25.82	0.7624	0.7384
81	SVR	148 indices	13.41	52.09	0.7219	0.2173	PLSR	ρ''	19.77	25.87	0.8208	0.7310
82	ABR	ρ_{CR}	12.37	52.38	0.8049	0.3285	DTR	ρ'	19.38	26.00	0.8278	0.7237
83	SVR	ρ'	6.98	52.43	0.9286	0.3648	KNR	$\log_{10}(\rho^{-1})$	25.48	26.21	0.7099	0.7544
84	KNR	ρ'	14.83	52.47	0.6602	0.2886	KNR	ρ	26.04	26.55	0.6988	0.7537
85	KNR	$[\log_{10}(\rho^{-1})]''$	15.54	52.69	0.6358	0.2972	LL	$\log_{10}(\rho^{-1})$	16.89	27.15	0.8698	0.7101
86	DTR	ρ_{CR}	15.16	52.75	0.6406	0.2865	DTR	ρ_{CR}	23.23	27.17	0.7525	0.6964
87	KNR	$[\log_{10}(\rho^{-1})]'$	14.54	52.78	0.6718	0.2498	DTR	$[\log_{10}(\rho^{-1})]''$	21.69	28.63	0.7842	0.6642
88	KNR	ρ''	15.66	52.83	0.6345	0.3442	DTR	ρ	24.51	29.30	0.7246	0.6466

Continued on next page

Table S.17 – Continued from previous page

Rank	Training on 2019–2020 data and testing on 2021–2022 data						Training and testing based on 80%-20% random split of all data					
	Method[1]	Input data	Training %RMSE	Testing %RMSE	Training r^2	Testing r^2	Method[1]	Input data	Training %RMSE	Testing %RMSE	Training r^2	Testing r^2
89	LL	ρ_{CR}	14.46	53.09	0.6757	0.0834	DTR	$\log_{10}(\rho^{-1})$	24.23	29.41	0.7307	0.6450
90	BR	ρ_{CR}	14.28	53.10	0.6839	0.0727	BR	$\log_{10}(\rho^{-1})$	18.32	31.38	0.8471	0.6370
91	Lasso	ρ_{CR}	14.48	53.19	0.6750	0.0825	KNR	ρ_{CR}	22.83	34.05	0.7833	0.5873
92	DTR	$[\log_{10}(\rho^{-1})]''$	16.68	53.19	0.5651	0.2078	MLPR	ρ_{CR}	15.37	34.52	0.8964	0.5596
93	SVR	ρ_{CR}	9.00	53.22	0.8795	0.2769	Lasso	ρ_{CR}	22.79	35.67	0.7645	0.5163
94	KNR	148 indices	15.94	53.39	0.6098	0.3057	LL	ρ_{CR}	22.79	35.68	0.7645	0.5162
95	Ridge	ρ_{CR}	14.06	53.64	0.6933	0.0649	KR	$\log_{10}(\rho^{-1})$	16.63	35.76	0.8740	0.5783
96	KR	ρ_{CR}	14.06	53.64	0.6934	0.0648	BR	ρ_{CR}	19.29	36.08	0.8309	0.5269
97	DTR	ρ	16.70	53.68	0.5638	0.2712	KNR	ρ'	30.20	36.97	0.6144	0.5024
98	DTR	$\log_{10}(\rho^{-1})$	16.61	53.87	0.5685	0.2647	KNR	$[\log_{10}(\rho^{-1})]'$	32.22	38.28	0.6022	0.5417
99	KNR	ρ	17.32	54.95	0.5550	0.3068	KR	ρ_{CR}	21.50	38.49	0.7911	0.4719
100	KNR	$\log_{10}(\rho^{-1})$	17.61	55.19	0.5385	0.3187	Ridge	ρ_{CR}	21.48	38.49	0.7915	0.4720
101	KNR	ρ_{CR}	15.98	56.61	0.6153	0.2434	KNR	$[\log_{10}(\rho^{-1})]''$	41.09	48.60	0.5374	0.3539
102	SVR	ρ''	4.80	57.86	0.9679	0.3312	KNR	ρ''	39.15	49.05	0.6130	0.3110
103	SVR	$[\log_{10}(\rho^{-1})]''$	5.07	58.53	0.9638	0.3194	GPR	148 indices	0.00	49.13	1.0000	0.1467
104	GPR	ρ	0.00	59.37	1.0000	0.0000	GPR	ρ	0.00	49.45	1.0000	0.0006
105	GPR	ρ'	0.00	59.37	1.0000	0.0000	GPR	ρ'	0.00	49.45	1.0000	0.0000
106	GPR	ρ''	0.00	59.37	1.0000	0.0000	GPR	ρ''	0.00	49.45	1.0000	0.0000
107	GPR	$\log_{10}(\rho^{-1})$	0.00	59.37	1.0000	0.0000	GPR	$\log_{10}(\rho^{-1})$	0.00	49.45	1.0000	0.0007
108	GPR	$[\log_{10}(\rho^{-1})]'$	0.00	59.37	1.0000	0.0000	GPR	$[\log_{10}(\rho^{-1})]'$	0.00	49.45	1.0000	0.0000
109	GPR	$[\log_{10}(\rho^{-1})]''$	0.00	59.37	1.0000	0.0000	GPR	$[\log_{10}(\rho^{-1})]''$	0.00	49.45	1.0000	0.0000
110	GPR	ρ_{CR}	0.00	59.37	1.0000	0.0000	GPR	ρ_{CR}	0.00	49.45	1.0000	0.0000
111	GPR	148 indices	0.00	59.37	1.0000	0.0001	PLSR	$\log_{10}(\rho^{-1})$	18.46	51.85	0.8438	0.4102
112	PLSR	ρ_{CR}	14.65	60.51	0.6643	0.0334	PLSR	ρ_{CR}	23.03	55.71	0.7567	0.2780

[1] AdaBoostRegressor, ABR; BayesianRidge, BR; DecisionTreeRegressor, DTR; GaussianProcessRegressor, GPR; GradientBoostingRegressor, GBR; KernelRidge, KR; KNeighborsRegressor, KNR; LassoLars, LL; MLPRegressor, MLPR; PLSRegression, PLSR; RandomForestRegressor, RFR; and support vector regression, SVR.

Table S.18: Goodness-of-fit statistics, including root mean squared errors (%RMSE) and coefficients of determination (r^2) between measured and modeled mass-basis cotton leaf chlorophyll $a+b$ (Chl $a+b$, mg g^{-1}) for training and testing of 14 machine learning methods from Python's "scikit-learn" package with 8 input data sets derived from leaf spectral reflectance (ρ). The 8 input data sets included spectral reflectance (ρ); the first and second derivatives of reflectance (ρ' and ρ'', respectively); the base-10 logarithm of the inverse of reflectance ($\log_{10}\rho^{-1}$) and its first and second derivatives [$(\log_{10}\rho^{-1})'$ and $(\log_{10}\rho^{-1})''$, respectively]; continuum-removed reflectance (ρ_{CR}); and the set of 148 spectral indices from Table S.1. Models were trained and tested by experiment using data from the 2019–2020 and 2021–2022 cotton field studies at Maricopa, Arizona, USA, respectively. Models were also trained and tested using an 80% and 20% random split of all from both experiments. Results are ranked according to the %RMSE of model testing.

	Training on 2019–2020 data and testing on 2021–2022 data						Training and testing based on 80%-20% random split of all data					
Rank	Method[1]	Input data	Training %RMSE	Testing %RMSE	Training r^2	Testing r^2	Method[1]	Input data	Training %RMSE	Testing %RMSE	Training r^2	Testing r^2
1	ABR	148 indices	12.20	16.61	0.7900	0.2277	SVR	ρ'	4.90	12.65	0.9600	0.7525
2	RFR	$[\log_{10}(\rho^{-1})]'$	6.66	17.20	0.9458	0.1613	SVR	$[\log_{10}(\rho^{-1})]'$	4.22	12.70	0.9704	0.7503
3	ABR	ρ'	10.72	17.23	0.8483	0.1075	MLPR	$[\log_{10}(\rho^{-1})]'$	7.25	12.81	0.9138	0.7437
4	ABR	$[\log_{10}(\rho^{-1})]'$	10.74	17.52	0.8443	0.1231	MLPR	ρ'	7.78	12.89	0.9002	0.7410
5	RFR	ρ'	6.02	17.62	0.9582	0.1546	SVR	$[\log_{10}(\rho^{-1})]''$	3.61	13.11	0.9797	0.7404
6	RFR	$[\log_{10}(\rho^{-1})]''$	6.47	17.64	0.9525	0.1121	MLPR	ρ	11.25	13.20	0.7791	0.7290
7	ABR	ρ''	10.30	17.94	0.8698	0.0617	SVR	ρ''	3.99	13.21	0.9747	0.7368
8	GBR	148 indices	6.06	17.94	0.9481	0.1644	SVR	ρ_{CR}	6.81	13.25	0.9216	0.7256
9	GBR	$[\log_{10}(\rho^{-1})]'$	6.43	18.12	0.9418	0.1236	MLPR	148 indices	10.95	13.36	0.7909	0.7212
10	RFR	148 indices	6.47	18.14	0.9482	0.1807	SVR	148 indices	10.62	13.38	0.8032	0.7215
11	RFR	ρ''	5.15	18.31	0.9710	0.0983	MLPR	$[\log_{10}(\rho^{-1})]''$	7.66	13.51	0.9056	0.7158
12	GBR	ρ'	5.44	18.36	0.9588	0.1038	GBR	ρ''	3.76	13.72	0.9793	0.7084
13	ABR	$[\log_{10}(\rho^{-1})]''$	10.26	18.52	0.8692	0.0403	GBR	$[\log_{10}(\rho^{-1})]''$	3.29	13.77	0.9835	0.7081
14	GBR	ρ''	5.59	18.86	0.9564	0.0607	MLPR	$\log_{10}(\rho^{-1})$	11.27	13.77	0.7780	0.7049
15	DTR	ρ'	15.55	18.87	0.6295	0.1401	SVR	$\log_{10}(\rho^{-1})$	10.04	13.80	0.8244	0.7023
16	ABR	ρ_{CR}	11.68	19.17	0.8132	0.1332	Ridge	$[\log_{10}(\rho^{-1})]'$	11.00	13.89	0.7915	0.6991
17	SVR	148 indices	10.25	19.31	0.8406	0.1005	KR	$[\log_{10}(\rho^{-1})]'$	11.01	13.90	0.7913	0.6991
18	GBR	$[\log_{10}(\rho^{-1})]''$	4.51	19.55	0.9718	0.0441	BR	$[\log_{10}(\rho^{-1})]'$	10.35	13.90	0.8150	0.6999
19	BR	148 indices	13.14	19.57	0.7357	0.1307	GBR	$[\log_{10}(\rho^{-1})]'$	6.67	13.99	0.9287	0.6978
20	Ridge	148 indices	13.20	19.59	0.7330	0.1371	SVR	ρ	9.32	14.02	0.8487	0.6926
21	KNR	$[\log_{10}(\rho^{-1})]'$	13.57	19.61	0.7265	0.1040	PLSR	$[\log_{10}(\rho^{-1})]'$	12.65	14.26	0.7170	0.6840
22	KNR	ρ'	13.51	19.62	0.7302	0.0713	GBR	ρ'	4.77	14.29	0.9660	0.6845
23	MLPR	148 indices	10.28	19.63	0.8400	0.1258	MLPR	ρ''	7.49	14.32	0.9099	0.6831
24	Lasso	148 indices	12.72	19.73	0.7520	0.1015	PLSR	148 indices	13.18	14.33	0.6930	0.6789

Continued on next page

Table S.18 – Continued from previous page

Rank	Training on 2019–2020 data and testing on 2021–2022 data						Training and testing based on 80%-20% random split of all data					
	Method[1]	Input data	Training %RMSE	Testing %RMSE	Training r^2	Testing r^2	Method[1]	Input data	Training %RMSE	Testing %RMSE	Training r^2	Testing r^2
25	KNR	ρ''	13.99	19.84	0.7183	0.0910	RFR	ρ''	6.82	14.35	0.9414	0.6911
26	KNR	ρ_{CR}	15.02	19.88	0.6723	0.2102	KR	ρ	11.66	14.36	0.7609	0.6785
27	PLSR	$[\log_{10}(\rho^{-1})]'$	10.47	20.09	0.8319	0.0881	KR	ρ'	11.05	14.38	0.7899	0.6788
28	Lasso	$[\log_{10}(\rho^{-1})]'$	10.07	20.23	0.8469	0.1099	Ridge	ρ'	11.06	14.38	0.7894	0.6788
29	LL	$[\log_{10}(\rho^{-1})]'$	10.05	20.24	0.8473	0.1097	LL	148 indices	13.01	14.45	0.7011	0.6737
30	ABR	ρ	12.78	20.24	0.7700	0.2102	BR	ρ'	10.35	14.47	0.8152	0.6772
31	RFR	$\log_{10}(\rho^{-1})$	7.26	20.29	0.9367	0.2415	KR	148 indices	13.14	14.51	0.6951	0.6708
32	LL	148 indices	12.29	20.29	0.7685	0.0678	Ridge	ρ	12.35	14.56	0.7319	0.6689
33	RFR	ρ_{CR}	6.49	20.29	0.9522	0.1382	GBR	148 indices	7.78	14.59	0.9020	0.6694
34	Ridge	$[\log_{10}(\rho^{-1})]'$	9.64	20.36	0.8598	0.0915	PLSR	ρ'	11.98	14.63	0.7462	0.6721
35	SVR	$[\log_{10}(\rho^{-1})]'$	4.31	20.37	0.9732	0.1510	BR	ρ	12.53	14.65	0.7239	0.6647
36	KR	$[\log_{10}(\rho^{-1})]'$	9.60	20.39	0.8610	0.0913	RFR	$[\log_{10}(\rho^{-1})]''$	5.65	14.71	0.9608	0.6798
37	KNR	$[\log_{10}(\rho^{-1})]''$	13.28	20.39	0.7425	0.1186	KR	$[\log_{10}(\rho^{-1})]''$	10.81	14.77	0.8012	0.6646
38	BR	$[\log_{10}(\rho^{-1})]'$	9.48	20.48	0.8644	0.0909	Ridge	$[\log_{10}(\rho^{-1})]''$	10.82	14.77	0.8010	0.6646
39	ABR	$\log_{10}(\rho^{-1})$	13.06	20.50	0.7592	0.2177	PLSR	ρ	12.14	14.77	0.7393	0.6629
40	DTR	148 indices	14.67	20.61	0.6701	0.1202	BR	$[\log_{10}(\rho^{-1})]''$	10.21	14.77	0.8218	0.6669
41	MLPR	$\log_{10}(\rho^{-1})$	10.78	20.61	0.8232	0.1582	RFR	ρ'	5.41	14.78	0.9635	0.6691
42	RFR	ρ	7.50	20.63	0.9307	0.2334	ABR	ρ'	11.21	14.78	0.7956	0.6775
43	KR	148 indices	12.14	20.63	0.7741	0.0485	RFR	$[\log_{10}(\rho^{-1})]'$	6.63	14.78	0.9393	0.6641
44	DTR	$[\log_{10}(\rho^{-1})]'$	13.48	20.79	0.7214	0.0979	Lasso	$[\log_{10}(\rho^{-1})]'$	11.64	14.87	0.7659	0.6550
45	GBR	ρ_{CR}	3.95	20.81	0.9793	0.1045	LL	$[\log_{10}(\rho^{-1})]'$	11.64	14.87	0.7657	0.6550
46	SVR	ρ_{CR}	4.96	21.00	0.9648	0.1493	Lasso	148 indices	13.47	14.90	0.6793	0.6529
47	KR	$\log_{10}(\rho^{-1})$	10.23	21.06	0.8402	0.0819	RFR	148 indices	5.95	14.93	0.9512	0.6583
48	Ridge	$\log_{10}(\rho^{-1})$	11.19	21.30	0.8084	0.1033	LL	ρ	12.22	14.99	0.7373	0.6504
49	PLSR	148 indices	12.19	21.36	0.7724	0.0604	Ridge	148 indices	13.58	15.01	0.6745	0.6479
50	DTR	$[\log_{10}(\rho^{-1})]''$	13.91	21.41	0.7034	0.0569	GBR	$\log_{10}(\rho^{-1})$	4.55	15.10	0.9716	0.6492
51	Ridge	ρ'	9.60	21.49	0.8612	0.0749	ABR	$[\log_{10}(\rho^{-1})]'$	10.93	15.12	0.8067	0.6537
52	KR	ρ'	9.59	21.50	0.8614	0.0749	ABR	ρ''	10.96	15.13	0.8238	0.6673
53	KNR	148 indices	14.16	21.55	0.6985	0.1216	GBR	ρ_{CR}	6.13	15.16	0.9459	0.6427
54	SVR	ρ'	3.72	21.55	0.9803	0.0783	BR	148 indices	13.80	15.17	0.6637	0.6403
55	Lasso	ρ'	10.11	21.55	0.8455	0.0942	ABR	$[\log_{10}(\rho^{-1})]''$	10.89	15.20	0.8259	0.6653
56	BR	ρ'	9.50	21.56	0.8639	0.0747	GBR	ρ	5.47	15.21	0.9573	0.6435

Continued on next page

Table S.18 – Continued from previous page

Rank	Training on 2019–2020 data and testing on 2021–2022 data						Training and testing based on 80%-20% random split of all data					
	Method[1]	Input data	Training %RMSE	Testing %RMSE	Training r^2	Testing r^2	Method[1]	Input data	Training %RMSE	Testing %RMSE	Training r^2	Testing r^2
57	LL	ρ'	10.11	21.57	0.8458	0.0942	Lasso	ρ'	12.01	15.25	0.7510	0.6373
58	GBR	$\log_{10}(\rho^{-1})$	6.99	21.63	0.9339	0.2030	LL	ρ'	12.01	15.25	0.7509	0.6372
59	GBR	ρ	6.57	21.64	0.9413	0.1828	LL	$\log_{10}(\rho^{-1})$	11.00	15.29	0.7873	0.6396
60	PLSR	ρ'	10.57	21.71	0.8287	0.0625	Lasso	$[\log_{10}(\rho^{-1})]''$	12.24	15.33	0.7438	0.6349
61	KR	ρ	10.19	21.71	0.8414	0.0778	LL	$[\log_{10}(\rho^{-1})]''$	12.26	15.34	0.7429	0.6342
62	PLSR	$\log_{10}(\rho^{-1})$	11.48	21.76	0.7981	0.1006	KR	ρ''	11.00	15.42	0.7940	0.6390
63	MLPR	ρ	10.40	21.83	0.8358	0.1274	Ridge	ρ''	11.01	15.42	0.7935	0.6389
64	Ridge	$[\log_{10}(\rho^{-1})]''$	10.06	21.86	0.8481	0.0592	BR	ρ''	10.33	15.43	0.8174	0.6419
65	KR	$[\log_{10}(\rho^{-1})]''$	10.06	21.86	0.8481	0.0592	RFR	$\log_{10}(\rho^{-1})$	7.35	15.49	0.9276	0.6357
66	PLSR	ρ	11.37	21.94	0.8017	0.0847	PLSR	$[\log_{10}(\rho^{-1})]''$	11.89	15.50	0.7502	0.6388
67	Lasso	$[\log_{10}(\rho^{-1})]''$	10.38	22.12	0.8377	0.0587	PLSR	ρ_{CR}	12.81	15.52	0.7099	0.6317
68	LL	$[\log_{10}(\rho^{-1})]''$	10.37	22.13	0.8378	0.0587	Lasso	ρ	13.63	15.65	0.6724	0.6186
69	SVR	$[\log_{10}(\rho^{-1})]''$	2.39	22.14	0.9926	0.1510	RFR	ρ	8.81	15.66	0.8896	0.6272
70	BR	$[\log_{10}(\rho^{-1})]''$	9.65	22.22	0.8599	0.0584	KR	$\log_{10}(\rho^{-1})$	11.37	15.72	0.7724	0.6238
71	LL	$\log_{10}(\rho^{-1})$	10.79	22.24	0.8220	0.0760	Lasso	ρ''	12.35	15.84	0.7384	0.6131
72	PLSR	$[\log_{10}(\rho^{-1})]''$	11.61	22.29	0.7934	0.0385	LL	ρ''	12.35	15.84	0.7383	0.6130
73	SVR	ρ''	2.38	22.40	0.9927	0.0859	RFR	ρ_{CR}	5.81	15.93	0.9599	0.6089
74	SVR	$\log_{10}(\rho^{-1})$	9.74	22.60	0.8566	0.0614	Ridge	$\log_{10}(\rho^{-1})$	11.45	15.94	0.7695	0.6149
75	SVR	ρ	9.73	22.67	0.8572	0.0576	KNR	148 indices	14.02	16.00	0.6550	0.6018
76	KR	ρ''	10.04	22.69	0.8488	0.0541	PLSR	ρ''	12.63	16.10	0.7181	0.6167
77	Ridge	ρ''	10.03	22.70	0.8491	0.0541	ABR	148 indices	12.25	16.14	0.7452	0.6083
78	BR	ρ_{CR}	10.57	22.73	0.8303	0.0881	ABR	ρ_{CR}	11.77	16.40	0.7773	0.5888
79	DTR	ρ	16.48	22.77	0.5836	0.2089	KNR	$[\log_{10}(\rho^{-1})]'$	14.58	16.59	0.6326	0.5734
80	KR	ρ_{CR}	10.22	22.87	0.8413	0.0891	ABR	ρ	13.44	16.77	0.6867	0.5632
81	Ridge	ρ_{CR}	10.21	22.88	0.8415	0.0891	MLPR	ρ_{CR}	8.23	16.77	0.8868	0.5950
82	BR	$\log_{10}(\rho^{-1})$	11.92	22.88	0.7828	0.1081	ABR	$\log_{10}(\rho^{-1})$	13.86	17.00	0.6705	0.5552
83	DTR	$\log_{10}(\rho^{-1})$	16.55	22.88	0.5804	0.1971	KNR	ρ'	15.03	17.01	0.6067	0.5505
84	BR	ρ''	9.72	23.01	0.8580	0.0529	KNR	ρ_{CR}	14.65	17.05	0.6354	0.5571
85	Lasso	ρ''	10.74	23.04	0.8262	0.0468	DTR	ρ''	14.75	17.14	0.6152	0.5437
86	LL	ρ''	10.74	23.04	0.8262	0.0468	DTR	$[\log_{10}(\rho^{-1})]''$	14.91	17.50	0.6069	0.5285
87	LL	ρ	10.35	23.08	0.8364	0.0656	DTR	148 indices	14.91	17.55	0.6068	0.5257
88	MLPR	$[\log_{10}(\rho^{-1})]'$	6.16	23.15	0.9451	0.0494	DTR	$\log_{10}(\rho^{-1})$	16.21	17.69	0.5355	0.5141

Continued on next page

Table S.18 – Continued from previous page

	Training on 2019–2020 data and testing on 2021–2022 data						Training and testing based on 80%-20% random split of all data					
Rank	Method[1]	Input data	Training %RMSE	Testing %RMSE	Training r^2	Testing r^2	Method[1]	Input data	Training %RMSE	Testing %RMSE	Training r^2	Testing r^2
89	DTR	ρ''	13.39	23.28	0.7253	0.0506	Lasso	$\log_{10}(\rho^{-1})$	13.73	17.74	0.6676	0.5304
90	GPR	148 indices	0.00	23.37	1.0000	0.0013	DTR	$[\log_{10}(\rho^{-1})]'$	14.02	17.99	0.6525	0.5096
91	GPR	ρ	0.00	23.37	1.0000	0.0000	KNR	ρ	16.70	18.01	0.5186	0.5091
92	GPR	ρ'	0.00	23.37	1.0000	0.0000	KNR	ρ''	16.76	18.22	0.5313	0.5005
93	GPR	ρ''	0.00	23.37	1.0000	0.0000	DTR	ρ	17.06	18.33	0.4853	0.4802
94	GPR	$\log_{10}(\rho^{-1})$	0.00	23.37	1.0000	0.0000	KNR	$\log_{10}(\rho^{-1})$	17.26	18.34	0.4932	0.5019
95	GPR	$[\log_{10}(\rho^{-1})]'$	0.00	23.37	1.0000	0.0000	BR	$\log_{10}(\rho^{-1})$	12.59	18.39	0.7213	0.5170
96	GPR	$[\log_{10}(\rho^{-1})]''$	0.00	23.37	1.0000	0.0000	KNR	$[\log_{10}(\rho^{-1})]''$	17.13	18.48	0.5296	0.5167
97	GPR	ρ_{CR}	0.00	23.37	1.0000	0.0000	DTR	ρ'	15.35	18.48	0.5835	0.4760
98	BR	ρ	12.06	23.45	0.7777	0.0933	PLSR	$\log_{10}(\rho^{-1})$	12.21	19.42	0.7364	0.4941
99	MLPR	$[\log_{10}(\rho^{-1})]''$	6.45	23.55	0.9405	0.0652	DTR	ρ_{CR}	16.09	19.51	0.5422	0.4204
100	Ridge	ρ	12.16	23.55	0.7741	0.0947	BR	ρ_{CR}	12.00	19.54	0.7482	0.4847
101	PLSR	ρ''	11.32	23.61	0.8038	0.0233	KR	ρ_{CR}	12.32	19.63	0.7350	0.4773
102	MLPR	ρ''	6.82	24.17	0.9331	0.0489	Ridge	ρ_{CR}	12.33	19.63	0.7346	0.4771
103	MLPR	ρ_{CR}	7.59	24.19	0.9153	0.0444	LL	ρ_{CR}	12.67	22.66	0.7199	0.3828
104	DTR	ρ_{CR}	14.11	24.50	0.6949	0.1149	Lasso	ρ_{CR}	12.64	22.85	0.7212	0.3789
105	Lasso	$\log_{10}(\rho^{-1})$	12.01	24.59	0.7794	0.0845	GPR	148 indices	0.00	25.09	1.0000	0.1032
106	MLPR	ρ'	6.49	24.64	0.9389	0.0463	GPR	ρ	0.00	25.31	1.0000	0.0176
107	Lasso	ρ	12.15	25.30	0.7739	0.0681	GPR	$\log_{10}(\rho^{-1})$	0.00	25.31	1.0000	0.0175
108	PLSR	ρ_{CR}	11.31	25.67	0.8041	0.0825	GPR	ρ'	0.00	25.31	1.0000	0.0000
109	KNR	ρ	17.35	25.71	0.5520	0.0404	GPR	ρ''	0.00	25.31	1.0000	0.0000
110	KNR	$\log_{10}(\rho^{-1})$	17.00	25.80	0.5699	0.0435	GPR	$[\log_{10}(\rho^{-1})]'$	0.00	25.31	1.0000	0.0000
111	LL	ρ_{CR}	10.65	28.03	0.8276	0.0407	GPR	$[\log_{10}(\rho^{-1})]''$	0.00	25.31	1.0000	0.0000
112	Lasso	ρ_{CR}	10.58	28.25	0.8297	0.0404	GPR	ρ_{CR}	0.00	25.31	1.0000	0.0000

[1] AdaBoostRegressor, ABR; BayesianRidge, BR; DecisionTreeRegressor, DTR; GaussianProcessRegressor, GPR; GradientBoostingRegressor, GBR; KernelRidge, KR; KNeighborsRegressor, KNR; LassoLars, LL; MLPRegressor, MLPR; PLSRegression, PLSR; RandomForestRegressor, RFR; and support vector regression, SVR.

Table S.19: Goodness-of-fit statistics, including root mean squared errors (%RMSE) and coefficients of determination (r^2) between measured and modeled mass-basis cotton leaf chlorophyll a (Chl a, mg g^{-1}) for training and testing of 14 machine learning methods from Python's "scikit-learn" package with 8 input data sets derived from leaf spectral reflectance (ρ). The 8 input data sets included spectral reflectance (ρ); the first and second derivatives of reflectance (ρ' and ρ'', respectively); the base-10 logarithm of the inverse of reflectance ($\log_{10}\rho^{-1}$) and its first and second derivatives [$(\log_{10}\rho^{-1})'$ and $(\log_{10}\rho^{-1})''$, respectively]; continuum-removed reflectance (ρ_{CR}); and the set of 148 spectral indices from Table S.1. Models were trained and tested by experiment using data from the 2019–2020 and 2021–2022 cotton field studies at Maricopa, Arizona, USA, respectively. Models were also trained and tested using an 80% and 20% random split of all from both experiments. Results are ranked according to the %RMSE of model testing.

	Training on 2019–2020 data and testing on 2021–2022 data						Training and testing based on 80%-20% random split of all data					
Rank	Method[1]	Input data	Training %RMSE	Testing %RMSE	Training r^2	Testing r^2	Method[1]	Input data	Training %RMSE	Testing %RMSE	Training r^2	Testing r^2
1	RFR	$[\log_{10}(\rho^{-1})]'$	6.55	16.97	0.9510	0.1135	SVR	ρ'	4.67	12.75	0.9614	0.7414
2	SVR	$[\log_{10}(\rho^{-1})]'$	4.67	17.40	0.9698	0.1231	SVR	$[\log_{10}(\rho^{-1})]'$	4.39	12.98	0.9660	0.7306
3	KNR	$[\log_{10}(\rho^{-1})]''$	13.40	17.55	0.7451	0.1307	MLPR	$[\log_{10}(\rho^{-1})]'$	8.05	13.10	0.8864	0.7234
4	ABR	$[\log_{10}(\rho^{-1})]'$	10.71	17.74	0.8514	0.0792	SVR	$[\log_{10}(\rho^{-1})]''$	3.62	13.17	0.9782	0.7306
5	ABR	ρ'	11.01	17.75	0.8531	0.0618	MLPR	ρ'	7.78	13.24	0.8945	0.7173
6	SVR	$[\log_{10}(\rho^{-1})]''$	2.43	17.79	0.9926	0.1232	SVR	ρ''	3.72	13.26	0.9765	0.7275
7	RFR	$[\log_{10}(\rho^{-1})]''$	6.91	17.80	0.9485	0.0734	MLPR	148 indices	10.63	13.50	0.7891	0.7059
8	KNR	$[\log_{10}(\rho^{-1})]'$	13.49	17.88	0.7362	0.1119	MLPR	$[\log_{10}(\rho^{-1})]''$	7.77	13.74	0.8970	0.6970
9	ABR	ρ_{CR}	11.70	17.92	0.8218	0.0859	MLPR	ρ_{CR}	8.02	13.78	0.8856	0.6928
10	SVR	ρ''	2.42	18.09	0.9927	0.0698	SVR	148 indices	10.69	13.80	0.7859	0.6933
11	SVR	ρ_{CR}	5.34	18.18	0.9604	0.1064	SVR	ρ_{CR}	7.10	13.80	0.9094	0.6916
12	ABR	ρ	13.32	18.19	0.7654	0.1449	MLPR	ρ	10.85	13.84	0.7794	0.6895
13	KNR	ρ_{CR}	15.08	18.28	0.6787	0.1304	GBR	$[\log_{10}(\rho^{-1})]''$	6.92	14.07	0.9215	0.6921
14	GBR	ρ'	5.60	18.46	0.9578	0.0692	SVR	$\log_{10}(\rho^{-1})$	10.25	14.27	0.8042	0.6692
15	KNR	ρ''	14.28	18.56	0.7159	0.0708	SVR	ρ	10.09	14.33	0.8102	0.6658
16	ABR	$[\log_{10}(\rho^{-1})]''$	10.11	18.56	0.8782	0.0354	GBR	ρ''	3.56	14.35	0.9804	0.6729
17	SVR	ρ'	3.52	18.56	0.9830	0.0616	BR	$[\log_{10}(\rho^{-1})]'$	10.43	14.42	0.7988	0.6647
18	ABR	ρ''	10.20	18.66	0.8750	0.0341	MLPR	ρ''	7.91	14.43	0.8932	0.6658
19	GPR	148 indices	0.00	18.70	1.0000	0.0002	KR	$[\log_{10}(\rho^{-1})]'$	11.02	14.44	0.7764	0.6632
20	GPR	ρ	0.00	18.70	1.0000	0.0000	Ridge	$[\log_{10}(\rho^{-1})]'$	11.03	14.45	0.7758	0.6631
21	GPR	ρ'	0.00	18.70	1.0000	0.0000	GBR	ρ'	6.54	14.59	0.9297	0.6591
22	GPR	ρ''	0.00	18.70	1.0000	0.0000	GBR	$[\log_{10}(\rho^{-1})]'$	6.55	14.61	0.9324	0.6607
23	GPR	$\log_{10}(\rho^{-1})$	0.00	18.70	1.0000	0.0000	PLSR	$[\log_{10}(\rho^{-1})]'$	11.98	14.78	0.7277	0.6485
24	GPR	$[\log_{10}(\rho^{-1})]'$	0.00	18.70	1.0000	0.0000	MLPR	$\log_{10}(\rho^{-1})$	11.96	14.99	0.7318	0.6351

Continued on next page

Table S.19 – Continued from previous page

	Training on 2019–2020 data and testing on 2021–2022 data						Training and testing based on 80%-20% random split of all data					
Rank	Method[1]	Input data	Training %RMSE	Testing %RMSE	Training r^2	Testing r^2	Method[1]	Input data	Training %RMSE	Testing %RMSE	Training r^2	Testing r^2
25	GPR	$[\log_{10}(\rho^{-1})]''$	0.00	18.70	1.0000	0.0000	KR	ρ	11.73	15.00	0.7403	0.6348
26	GPR	ρ_{CR}	0.00	18.70	1.0000	0.0000	LL	148 indices	13.15	15.01	0.6718	0.6348
27	ABR	$\log_{10}(\rho^{-1})$	13.04	18.71	0.7701	0.1273	KR	ρ'	11.07	15.02	0.7750	0.6368
28	ABR	148 indices	12.37	18.79	0.7863	0.1235	Ridge	ρ'	11.10	15.02	0.7738	0.6366
29	GBR	$[\log_{10}(\rho^{-1})]'$	4.92	18.83	0.9670	0.0719	PLSR	148 indices	13.56	15.06	0.6511	0.6330
30	RFR	ρ'	5.77	18.93	0.9644	0.0896	BR	ρ'	10.42	15.06	0.7999	0.6366
31	RFR	ρ_{CR}	6.60	19.10	0.9531	0.0895	ABR	$[\log_{10}(\rho^{-1})]''$	10.65	15.07	0.8216	0.6678
32	SVR	148 indices	10.39	19.10	0.8415	0.0626	RFR	ρ''	5.75	15.10	0.9589	0.6492
33	RFR	ρ''	6.15	19.23	0.9585	0.0554	RFR	$[\log_{10}(\rho^{-1})]'$	6.85	15.13	0.9330	0.6402
34	RFR	$\log_{10}(\rho^{-1})$	6.79	19.36	0.9482	0.1515	GBR	148 indices	6.13	15.14	0.9401	0.6316
35	GBR	148 indices	6.15	19.46	0.9491	0.1025	LL	$[\log_{10}(\rho^{-1})]'$	11.72	15.17	0.7460	0.6268
36	RFR	ρ	7.78	19.55	0.9277	0.1539	Lasso	$[\log_{10}(\rho^{-1})]'$	11.73	15.17	0.7457	0.6265
37	RFR	148 indices	6.99	19.55	0.9413	0.1178	Ridge	ρ	12.45	15.19	0.7074	0.6248
38	GBR	$[\log_{10}(\rho^{-1})]''$	5.35	19.63	0.9615	0.0488	ABR	$[\log_{10}(\rho^{-1})]'$	10.95	15.21	0.7940	0.6386
39	GBR	$\log_{10}(\rho^{-1})$	6.87	19.76	0.9391	0.1414	KR	148 indices	13.51	15.25	0.6541	0.6237
40	KNR	ρ'	13.36	19.78	0.7446	0.0575	ABR	ρ'	11.18	15.29	0.7952	0.6533
41	GBR	ρ	6.80	19.91	0.9393	0.1364	PLSR	ρ'	11.87	15.32	0.7326	0.6274
42	DTR	148 indices	14.89	20.01	0.6707	0.0925	PLSR	ρ	12.25	15.34	0.7151	0.6201
43	GBR	ρ_{CR}	4.27	20.39	0.9769	0.0693	BR	$[\log_{10}(\rho^{-1})]''$	10.54	15.34	0.7969	0.6271
44	MLPR	$\log_{10}(\rho^{-1})$	10.91	20.55	0.8244	0.0685	BR	ρ	12.79	15.36	0.6913	0.6162
45	GBR	ρ''	5.17	20.60	0.9639	0.0315	KR	$[\log_{10}(\rho^{-1})]''$	11.17	15.38	0.7736	0.6231
46	DTR	ρ	17.65	20.64	0.5374	0.1287	Ridge	$[\log_{10}(\rho^{-1})]''$	11.17	15.38	0.7734	0.6231
47	DTR	$\log_{10}(\rho^{-1})$	17.65	20.64	0.5374	0.1287	KR	$\log_{10}(\rho^{-1})$	11.45	15.42	0.7525	0.6159
48	KNR	148 indices	14.09	21.08	0.7103	0.0927	RFR	ρ'	6.61	15.45	0.9430	0.6330
49	Lasso	148 indices	13.03	21.27	0.7482	0.0743	LL	$\log_{10}(\rho^{-1})$	11.21	15.46	0.7627	0.6135
50	BR	148 indices	13.40	21.34	0.7336	0.0958	RFR	$[\log_{10}(\rho^{-1})]''$	6.74	15.47	0.9396	0.6347
51	MLPR	ρ	10.65	21.43	0.8327	0.0606	RFR	148 indices	6.07	15.50	0.9503	0.6212
52	SVR	ρ	9.27	21.65	0.8749	0.0207	LL	ρ	11.75	15.50	0.7393	0.6111
53	SVR	$\log_{10}(\rho^{-1})$	9.32	21.67	0.8733	0.0231	GBR	ρ	4.88	15.56	0.9661	0.6158
54	MLPR	148 indices	10.41	21.79	0.8414	0.0730	GBR	ρ_{CR}	5.30	15.63	0.9575	0.6080
55	DTR	$[\log_{10}(\rho^{-1})]'$	14.07	21.97	0.7063	0.0673	Lasso	148 indices	13.83	15.63	0.6373	0.6038
56	Lasso	$[\log_{10}(\rho^{-1})]'$	9.74	22.08	0.8614	0.0794	GBR	$\log_{10}(\rho^{-1})$	4.60	15.67	0.9678	0.6102

Continued on next page

Table S.19 – Continued from previous page

Rank	Training on 2019–2020 data and testing on 2021–2022 data						Training and testing based on 80%-20% random split of all data					
	Method[1]	Input data	Training %RMSE	Testing %RMSE	Training r^2	Testing r^2	Method[1]	Input data	Training %RMSE	Testing %RMSE	Training r^2	Testing r^2
57	LL	$[\log_{10}(\rho^{-1})]'$	9.73	22.11	0.8617	0.0791	LL	ρ'	11.95	15.68	0.7368	0.6015
58	PLSR	$\log_{10}(\rho^{-1})$	11.66	22.15	0.7981	0.0526	Lasso	ρ'	11.94	15.68	0.7371	0.6015
59	Ridge	148 indices	13.90	22.18	0.7142	0.1275	ABR	ρ''	10.89	15.76	0.8071	0.6281
60	KR	$[\log_{10}(\rho^{-1})]'$	9.34	22.44	0.8726	0.0617	Lasso	$[\log_{10}(\rho^{-1})]''$	12.55	15.82	0.7120	0.5977
61	Ridge	$[\log_{10}(\rho^{-1})]'$	9.34	22.44	0.8726	0.0617	LL	$[\log_{10}(\rho^{-1})]''$	12.56	15.82	0.7115	0.5975
62	DTR	$[\log_{10}(\rho^{-1})]''$	14.66	22.46	0.6812	0.0249	BR	148 indices	14.13	15.93	0.6213	0.5879
63	BR	$\log_{10}(\rho^{-1})$	12.07	22.46	0.7842	0.0620	BR	ρ''	10.63	16.02	0.7933	0.5993
64	BR	$[\log_{10}(\rho^{-1})]'$	9.27	22.53	0.8745	0.0616	KR	ρ''	11.30	16.05	0.7679	0.5943
65	KNR	$\log_{10}(\rho^{-1})$	18.03	22.60	0.5323	0.0114	Ridge	ρ''	11.33	16.06	0.7669	0.5941
66	KNR	ρ	18.03	22.74	0.5310	0.0087	Ridge	148 indices	14.36	16.06	0.6094	0.5814
67	PLSR	ρ	10.77	22.87	0.8278	0.0602	PLSR	$[\log_{10}(\rho^{-1})]''$	12.14	16.10	0.7203	0.5968
68	BR	ρ_{CR}	10.54	22.92	0.8369	0.0737	Lasso	ρ	13.67	16.26	0.6466	0.5716
69	Ridge	ρ	11.99	23.01	0.7869	0.0527	RFR	$\log_{10}(\rho^{-1})$	7.54	16.46	0.9206	0.5736
70	LL	$\log_{10}(\rho^{-1})$	10.74	23.05	0.8292	0.0353	RFR	ρ_{CR}	7.64	16.50	0.9244	0.5697
71	KR	148 indices	12.16	23.17	0.7806	0.0027	RFR	ρ	8.33	16.53	0.8989	0.5688
72	DTR	ρ_{CR}	14.56	23.19	0.6854	0.0657	LL	ρ''	12.15	16.53	0.7295	0.5665
73	BR	ρ	12.23	23.21	0.7784	0.0534	Lasso	ρ''	12.15	16.54	0.7295	0.5664
74	DTR	ρ'	14.76	23.39	0.6766	0.0984	ABR	148 indices	12.52	16.59	0.7150	0.5715
75	KR	ρ	9.80	23.52	0.8580	0.0449	ABR	ρ_{CR}	11.64	16.71	0.7676	0.5543
76	KR	ρ_{CR}	9.89	23.59	0.8559	0.0745	Lasso	$\log_{10}(\rho^{-1})$	13.54	16.73	0.6532	0.5491
77	Ridge	ρ_{CR}	9.88	23.60	0.8561	0.0745	KNR	148 indices	14.16	16.80	0.6227	0.5466
78	LL	148 indices	12.09	23.67	0.7832	0.0009	PLSR	ρ''	12.83	16.85	0.6877	0.5659
79	KR	ρ'	9.32	23.88	0.8732	0.0470	BR	ρ_{CR}	12.02	16.89	0.7295	0.5537
80	Ridge	ρ'	9.30	23.90	0.8739	0.0471	KNR	$[\log_{10}(\rho^{-1})]'$	14.03	16.93	0.6294	0.5397
81	PLSR	$[\log_{10}(\rho^{-1})]'$	10.86	23.91	0.8250	0.0337	Ridge	ρ_{CR}	12.36	16.96	0.7145	0.5476
82	BR	ρ'	9.28	23.92	0.8743	0.0471	KR	ρ_{CR}	12.38	16.97	0.7138	0.5473
83	PLSR	148 indices	12.43	23.93	0.7707	0.0189	KNR	$[\log_{10}(\rho^{-1})]''$	15.29	17.05	0.5697	0.5435
84	Lasso	ρ'	9.95	23.93	0.8555	0.0573	ABR	ρ	13.94	17.54	0.6531	0.5111
85	KR	$\log_{10}(\rho^{-1})$	9.95	23.94	0.8536	0.0359	BR	$\log_{10}(\rho^{-1})$	12.78	17.56	0.6916	0.5196
86	LL	ρ'	9.93	23.96	0.8561	0.0575	Ridge	$\log_{10}(\rho^{-1})$	12.79	17.57	0.6911	0.5194
87	Lasso	$\log_{10}(\rho^{-1})$	12.29	24.03	0.7764	0.0503	KNR	ρ_{CR}	14.40	17.57	0.6132	0.4994
88	LL	ρ	10.26	24.07	0.8443	0.0367	KNR	ρ'	14.86	17.71	0.5899	0.5033

Continued on next page

Table S.19 – Continued from previous page

	Training on 2019–2020 data and testing on 2021–2022 data						Training and testing based on 80%-20% random split of all data					
Rank	Method[1]	Input data	Training %RMSE	Testing %RMSE	Training r^2	Testing r^2	Method[1]	Input data	Training %RMSE	Testing %RMSE	Training r^2	Testing r^2
89	PLSR	ρ'	10.66	24.27	0.8313	0.0346	ABR	$\log_{10}(\rho^{-1})$	13.97	18.01	0.6430	0.4781
90	PLSR	$[\log_{10}(\rho^{-1})]''$	11.28	24.36	0.8110	0.0301	KNR	ρ''	15.92	18.07	0.5244	0.4735
91	PLSR	ρ_{CR}	11.55	24.41	0.8020	0.0861	PLSR	$\log_{10}(\rho^{-1})$	12.37	18.31	0.7096	0.5006
92	Lasso	$[\log_{10}(\rho^{-1})]''$	10.35	24.48	0.8440	0.0466	DTR	$[\log_{10}(\rho^{-1})]''$	14.81	18.48	0.5841	0.4523
93	LL	$[\log_{10}(\rho^{-1})]''$	10.33	24.49	0.8445	0.0465	DTR	$[\log_{10}(\rho^{-1})]'$	14.36	18.52	0.6089	0.4503
94	Ridge	$[\log_{10}(\rho^{-1})]''$	9.82	24.56	0.8599	0.0435	KNR	$\log_{10}(\rho^{-1})$	17.30	18.87	0.4494	0.4450
95	KR	$[\log_{10}(\rho^{-1})]''$	9.82	24.56	0.8599	0.0435	KNR	ρ	16.95	18.90	0.4693	0.4335
96	Lasso	ρ_{CR}	10.72	24.63	0.8310	0.0516	LL	ρ_{CR}	12.80	19.02	0.6940	0.4584
97	LL	ρ_{CR}	10.72	24.63	0.8312	0.0492	DTR	ρ_{CR}	15.34	19.22	0.5533	0.4121
98	DTR	ρ''	13.65	24.65	0.7236	0.0445	DTR	ρ''	14.74	19.32	0.5876	0.4194
99	Lasso	ρ	12.45	24.79	0.7703	0.0409	Lasso	ρ_{CR}	12.86	19.35	0.6913	0.4462
100	BR	$[\log_{10}(\rho^{-1})]''$	9.49	25.00	0.8690	0.0425	DTR	148 indices	15.01	19.45	0.5726	0.3942
101	PLSR	ρ''	11.47	25.07	0.8046	0.0141	DTR	ρ	17.58	19.48	0.4135	0.3849
102	MLPR	$[\log_{10}(\rho^{-1})]'$	5.86	25.66	0.9522	0.0329	DTR	$\log_{10}(\rho^{-1})$	17.58	19.48	0.4135	0.3849
103	KR	ρ''	9.76	25.93	0.8617	0.0344	DTR	ρ'	15.61	19.98	0.5379	0.3710
104	Ridge	ρ''	9.76	25.93	0.8618	0.0344	PLSR	ρ_{CR}	12.95	20.29	0.6816	0.4338
105	BR	ρ''	9.55	26.21	0.8674	0.0341	GPR	148 indices	0.00	24.62	1.0000	0.0970
106	Lasso	ρ''	10.62	26.52	0.8356	0.0278	GPR	ρ	0.00	24.85	1.0000	0.0239
107	LL	ρ''	10.62	26.53	0.8358	0.0278	GPR	ρ'	0.00	24.85	1.0000	0.0000
108	MLPR	$[\log_{10}(\rho^{-1})]''$	6.62	26.89	0.9392	0.0410	GPR	ρ''	0.00	24.85	1.0000	0.0000
109	MLPR	ρ'	5.78	27.59	0.9535	0.0217	GPR	$\log_{10}(\rho^{-1})$	0.00	24.85	1.0000	0.0239
110	MLPR	ρ_{CR}	7.13	28.03	0.9279	0.0157	GPR	$[\log_{10}(\rho^{-1})]'$	0.00	24.85	1.0000	0.0000
111	MLPR	ρ''	6.53	28.11	0.9410	0.0270	GPR	$[\log_{10}(\rho^{-1})]''$	0.00	24.85	1.0000	0.0000
112	Ridge	$\log_{10}(\rho^{-1})$	8.81	33.52	0.8855	0.0120	GPR	ρ_{CR}	0.00	24.85	1.0000	0.0000

[1] AdaBoostRegressor, ABR; BayesianRidge, BR; DecisionTreeRegressor, DTR; GaussianProcessRegressor, GPR; GradientBoostingRegressor, GBR; KernelRidge, KR; KNeighborsRegressor, KNR; LassoLars, LL; MLPRegressor, MLPR; PLSRegression, PLSR; RandomForestRegressor, RFR; and support vector regression, SVR.

Table S.20: Goodness-of-fit statistics, including root mean squared errors (%RMSE) and coefficients of determination (r^2) between measured and modeled mass-basis cotton leaf chlorophyll b (Chl b, mg g^{-1}) for training and testing of 14 machine learning methods from Python's "scikit-learn" package with 8 input data sets derived from leaf spectral reflectance (ρ). The 8 input data sets included spectral reflectance (ρ); the first and second derivatives of reflectance (ρ' and ρ'', respectively); the base-10 logarithm of the inverse of reflectance ($\log_{10}\rho^{-1}$) and its first and second derivatives [$(\log_{10}\rho^{-1})'$ and $(\log_{10}\rho^{-1})''$, respectively]; continuum-removed reflectance (ρ_{CR}); and the set of 148 spectral indices from Table S.1. Models were trained and tested by experiment using data from the 2019–2020 and 2021–2022 cotton field studies at Maricopa, Arizona, USA, respectively. Models were also trained and tested using an 80% and 20% random split of all from both experiments. Results are ranked according to the %RMSE of model testing.

	Training on 2019–2020 data and testing on 2021–2022 data						Training and testing based on 80%-20% random split of all data					
Rank	Method[1]	Input data	Training %RMSE	Testing %RMSE	Training r^2	Testing r^2	Method[1]	Input data	Training %RMSE	Testing %RMSE	Training r^2	Testing r^2
1	MLPR	$[\log_{10}(\rho^{-1})]'$	8.59	33.92	0.9264	0.0870	SVR	ρ_{CR}	10.19	16.87	0.9357	0.8075
2	MLPR	ρ'	10.21	34.00	0.8936	0.1016	MLPR	$[\log_{10}(\rho^{-1})]'$	9.52	16.91	0.9446	0.8070
3	MLPR	ρ''	10.14	34.04	0.8975	0.1045	MLPR	ρ	16.90	17.07	0.8146	0.8028
4	MLPR	$[\log_{10}(\rho^{-1})]''$	9.84	34.16	0.9045	0.1012	MLPR	ρ'	10.96	17.15	0.9259	0.8021
5	BR	148 indices	18.25	34.20	0.6362	0.2290	GBR	ρ'	10.95	17.37	0.9262	0.7965
6	Ridge	148 indices	18.21	34.23	0.6375	0.2270	MLPR	$\log_{10}(\rho^{-1})$	17.38	17.38	0.8037	0.7961
7	GBR	ρ'	7.96	34.35	0.9388	0.1456	SVR	$[\log_{10}(\rho^{-1})]'$	6.68	17.51	0.9730	0.7926
8	GBR	$[\log_{10}(\rho^{-1})]''$	7.63	34.42	0.9444	0.1568	MLPR	148 indices	16.71	17.51	0.8186	0.7925
9	PLSR	ρ'	16.49	34.82	0.7026	0.1258	SVR	ρ'	5.89	17.65	0.9788	0.7887
10	Lasso	148 indices	18.19	34.91	0.6384	0.2138	GBR	$[\log_{10}(\rho^{-1})]'$	13.15	17.74	0.8909	0.7874
11	LL	$[\log_{10}(\rho^{-1})]''$	16.95	35.01	0.6914	0.1014	GBR	$[\log_{10}(\rho^{-1})]''$	8.20	17.80	0.9587	0.7863
12	Lasso	$[\log_{10}(\rho^{-1})]''$	16.95	35.01	0.6914	0.1014	PLSR	148 indices	19.68	17.85	0.7460	0.7854
13	GBR	ρ_{CR}	6.99	35.37	0.9534	0.1720	GBR	ρ''	8.03	17.88	0.9623	0.7846
14	PLSR	$[\log_{10}(\rho^{-1})]''$	17.44	35.41	0.6674	0.1113	LL	148 indices	19.61	17.96	0.7479	0.7825
15	ABR	ρ'	14.08	35.55	0.8154	0.1639	Ridge	148 indices	19.66	17.98	0.7465	0.7822
16	GBR	148 indices	11.43	35.58	0.8717	0.2485	KR	148 indices	19.71	18.02	0.7454	0.7813
17	Lasso	ρ''	17.24	35.58	0.6804	0.1274	RFR	ρ'	9.04	18.04	0.9561	0.7808
18	LL	ρ''	17.24	35.58	0.6804	0.1274	GBR	148 indices	11.56	18.06	0.9172	0.7789
19	KR	$[\log_{10}(\rho^{-1})]'$	15.93	35.80	0.7250	0.1518	SVR	$\log_{10}(\rho^{-1})$	15.48	18.11	0.8454	0.7785
20	Ridge	$[\log_{10}(\rho^{-1})]'$	15.94	35.81	0.7248	0.1519	RFR	148 indices	11.77	18.20	0.9170	0.7758
21	BR	ρ''	15.74	35.84	0.7336	0.1233	GBR	ρ_{CR}	11.14	18.23	0.9249	0.7754
22	BR	$[\log_{10}(\rho^{-1})]''$	15.59	35.86	0.7388	0.0867	RFR	ρ''	8.09	18.24	0.9672	0.7768
23	KR	ρ''	16.11	35.86	0.7208	0.1296	SVR	ρ	14.83	18.31	0.8584	0.7742
24	Ridge	ρ''	16.11	35.86	0.7208	0.1297	SVR	148 indices	16.23	18.37	0.8308	0.7722

Continued on next page

Table S.20 – Continued from previous page

Rank	Training on 2019–2020 data and testing on 2021–2022 data						Training and testing based on 80%-20% random split of all data					
	Method[1]	Input data	Training %RMSE	Testing %RMSE	Training r^2	Testing r^2	Method[1]	Input data	Training %RMSE	Testing %RMSE	Training r^2	Testing r^2
25	KR	ρ'	15.78	35.86	0.7305	0.1643	MLPR	$[\log_{10}(\rho^{-1})]''$	8.68	18.40	0.9552	0.7717
26	Ridge	ρ'	15.79	35.87	0.7302	0.1645	RFR	$[\log_{10}(\rho^{-1})]'$	10.04	18.43	0.9449	0.7723
27	BR	$[\log_{10}(\rho^{-1})]'$	16.09	35.87	0.7194	0.1535	RFR	$[\log_{10}(\rho^{-1})]''$	9.09	18.51	0.9573	0.7717
28	PLSR	ρ''	17.51	35.89	0.6648	0.1558	GBR	ρ	6.18	18.68	0.9791	0.7677
29	PLSR	$\log_{10}(\rho^{-1})$	16.64	35.89	0.6974	0.1222	Ridge	ρ'	17.12	18.83	0.8123	0.7606
30	KR	$[\log_{10}(\rho^{-1})]''$	16.02	35.92	0.7247	0.0906	KR	ρ'	17.12	18.83	0.8125	0.7606
31	Ridge	$[\log_{10}(\rho^{-1})]''$	16.02	35.92	0.7247	0.0906	BR	148 indices	20.53	18.83	0.7238	0.7612
32	BR	ρ'	16.01	35.97	0.7224	0.1684	Lasso	148 indices	20.39	18.84	0.7274	0.7613
33	RFR	ρ'	8.01	36.01	0.9456	0.2077	KR	ρ	18.48	19.02	0.7770	0.7581
34	MLPR	148 indices	15.31	36.03	0.7467	0.1421	SVR	ρ''	4.98	19.16	0.9857	0.7531
35	RFR	148 indices	11.04	36.08	0.8843	0.2517	MLPR	ρ''	9.74	19.19	0.9429	0.7549
36	RFR	ρ''	7.22	36.12	0.9602	0.1707	Ridge	ρ	18.99	19.21	0.7645	0.7529
37	LL	ρ'	16.01	36.18	0.7222	0.1797	Ridge	$[\log_{10}(\rho^{-1})]'$	17.17	19.22	0.8113	0.7506
38	Lasso	ρ'	16.00	36.18	0.7224	0.1797	KR	$[\log_{10}(\rho^{-1})]'$	17.15	19.22	0.8116	0.7505
39	GBR	$[\log_{10}(\rho^{-1})]'$	4.55	36.24	0.9807	0.0886	ABR	ρ'	16.77	19.25	0.8542	0.7799
40	LL	148 indices	17.52	36.30	0.6645	0.1253	BR	ρ'	15.66	19.25	0.8425	0.7526
41	LL	$[\log_{10}(\rho^{-1})]'$	16.63	36.40	0.7000	0.1637	GBR	$\log_{10}(\rho^{-1})$	5.87	19.30	0.9801	0.7521
42	Lasso	$[\log_{10}(\rho^{-1})]'$	16.62	36.42	0.7001	0.1634	PLSR	ρ'	19.41	19.31	0.7530	0.7492
43	ABR	148 indices	17.52	36.60	0.7000	0.2243	RFR	ρ_{CR}	10.68	19.36	0.9417	0.7504
44	ABR	$[\log_{10}(\rho^{-1})]'$	14.01	36.87	0.8228	0.1097	BR	$[\log_{10}(\rho^{-1})]'$	15.90	19.60	0.8377	0.7428
45	KR	148 indices	17.37	36.96	0.6701	0.1073	BR	ρ	19.71	19.61	0.7463	0.7421
46	RFR	$[\log_{10}(\rho^{-1})]'$	7.68	36.98	0.9511	0.1310	RFR	ρ	12.48	19.62	0.9160	0.7440
47	LL	$\log_{10}(\rho^{-1})$	16.80	37.05	0.6919	0.1326	SVR	$[\log_{10}(\rho^{-1})]''$	3.67	19.62	0.9928	0.7418
48	MLPR	ρ	15.18	37.05	0.7504	0.1293	RFR	$\log_{10}(\rho^{-1})$	9.33	19.66	0.9572	0.7415
49	Lasso	ρ	17.45	37.10	0.6677	0.1584	PLSR	$[\log_{10}(\rho^{-1})]'$	19.83	19.71	0.7422	0.7382
50	LL	ρ	16.90	37.29	0.6882	0.1265	Lasso	ρ'	17.74	19.72	0.7973	0.7383
51	Ridge	$\log_{10}(\rho^{-1})$	16.84	37.39	0.6904	0.1408	LL	ρ'	17.74	19.72	0.7972	0.7382
52	KR	$\log_{10}(\rho^{-1})$	16.86	37.43	0.6897	0.1415	PLSR	ρ	19.05	19.76	0.7620	0.7431
53	Lasso	$\log_{10}(\rho^{-1})$	17.42	37.52	0.6688	0.1644	Lasso	$[\log_{10}(\rho^{-1})]'$	18.12	19.80	0.7885	0.7357
54	DTR	ρ'	18.56	37.60	0.6235	0.1501	LL	$[\log_{10}(\rho^{-1})]'$	18.12	19.80	0.7884	0.7357
55	ABR	ρ''	14.28	37.77	0.8314	0.1162	LL	ρ	19.02	19.95	0.7637	0.7359
56	KR	ρ	16.91	37.77	0.6879	0.1300	Ridge	$[\log_{10}(\rho^{-1})]''$	16.56	19.95	0.8267	0.7315

Continued on next page

Table S.20 – Continued from previous page

Rank	Training on 2019–2020 data and testing on 2021–2022 data						Training and testing based on 80%-20% random split of all data					
	Method[1]	Input data	Training %RMSE	Testing %RMSE	Training r^2	Testing r^2	Method[1]	Input data	Training %RMSE	Testing %RMSE	Training r^2	Testing r^2
57	PLSR	$[\log_{10}(\rho^{-1})]'$	17.53	37.84	0.6639	0.1373	KR	$[\log_{10}(\rho^{-1})]''$	16.55	19.95	0.8269	0.7315
58	PLSR	148 indices	17.28	37.90	0.6735	0.0522	ABR	$[\log_{10}(\rho^{-1})]'$	17.05	20.03	0.8547	0.7674
59	GBR	ρ''	5.11	37.90	0.9769	0.1240	KNR	148 indices	20.67	20.12	0.7223	0.7267
60	Ridge	ρ	17.13	37.96	0.6798	0.1434	Ridge	ρ''	17.08	20.14	0.8151	0.7281
61	PLSR	ρ	17.74	38.03	0.6561	0.1423	KR	ρ''	17.05	20.14	0.8157	0.7281
62	BR	ρ	17.47	38.26	0.6672	0.1664	BR	$[\log_{10}(\rho^{-1})]''$	15.27	20.15	0.8515	0.7289
63	ABR	$[\log_{10}(\rho^{-1})]''$	14.40	38.26	0.8335	0.0725	ABR	148 indices	19.44	20.24	0.7793	0.7554
64	RFR	$[\log_{10}(\rho^{-1})]''$	10.42	38.40	0.9070	0.1482	ABR	ρ_{CR}	18.29	20.34	0.8138	0.7423
65	BR	$\log_{10}(\rho^{-1})$	17.55	38.58	0.6640	0.1805	ABR	ρ''	17.39	20.38	0.8303	0.7402
66	ABR	ρ_{CR}	15.42	38.78	0.7906	0.2091	BR	ρ''	15.47	20.45	0.8472	0.7239
67	DTR	148 indices	18.86	38.91	0.6110	0.1428	Lasso	$[\log_{10}(\rho^{-1})]''$	17.36	20.51	0.8078	0.7171
68	MLPR	$\log_{10}(\rho^{-1})$	16.43	38.92	0.7061	0.2004	LL	$[\log_{10}(\rho^{-1})]''$	17.42	20.52	0.8065	0.7168
69	RFR	ρ_{CR}	11.47	39.06	0.8806	0.2522	ABR	$[\log_{10}(\rho^{-1})]''$	17.48	20.52	0.8349	0.7443
70	DTR	$[\log_{10}(\rho^{-1})]'$	18.82	39.17	0.6130	0.0723	LL	ρ''	18.25	20.98	0.7872	0.7043
71	GBR	ρ	10.55	39.68	0.8929	0.1494	Lasso	ρ''	18.26	20.98	0.7871	0.7043
72	DTR	ρ_{CR}	19.41	39.81	0.5883	0.2126	Lasso	ρ	21.50	21.15	0.6978	0.6995
73	ABR	$\log_{10}(\rho^{-1})$	17.87	39.85	0.7002	0.2204	PLSR	ρ''	20.14	21.43	0.7339	0.6971
74	DTR	ρ''	19.63	39.96	0.5789	0.1137	PLSR	$[\log_{10}(\rho^{-1})]''$	18.68	21.44	0.7710	0.6984
75	SVR	$[\log_{10}(\rho^{-1})]'$	5.53	40.03	0.9690	0.0920	KNR	ρ	24.56	21.74	0.6161	0.6970
76	GBR	$\log_{10}(\rho^{-1})$	8.93	40.12	0.9235	0.1429	DTR	148 indices	19.69	21.97	0.7457	0.6801
77	ABR	ρ	17.72	40.14	0.7002	0.2119	DTR	ρ	23.68	22.21	0.6323	0.6664
78	SVR	148 indices	15.67	40.63	0.7360	0.1161	KNR	$\log_{10}(\rho^{-1})$	24.97	22.37	0.6074	0.6830
79	KNR	ρ'	18.90	40.96	0.6178	0.1063	DTR	ρ'	18.81	22.86	0.7678	0.6632
80	RFR	$\log_{10}(\rho^{-1})$	8.70	41.14	0.9385	0.1905	DTR	$\log_{10}(\rho^{-1})$	23.02	22.98	0.6524	0.6431
81	DTR	$[\log_{10}(\rho^{-1})]''$	19.36	41.20	0.5902	0.1037	ABR	$\log_{10}(\rho^{-1})$	21.40	23.07	0.7392	0.6695
82	SVR	ρ_{CR}	6.80	41.31	0.9528	0.1378	DTR	$[\log_{10}(\rho^{-1})]''$	22.33	23.19	0.6728	0.6424
83	SVR	ρ'	7.02	41.68	0.9497	0.1064	ABR	ρ	21.63	23.28	0.7162	0.6518
84	RFR	ρ	12.47	41.77	0.8528	0.1827	LL	$\log_{10}(\rho^{-1})$	18.01	23.58	0.7883	0.6507
85	KNR	ρ''	18.60	41.93	0.6439	0.0826	DTR	ρ''	21.61	23.90	0.6937	0.6234
86	SVR	$\log_{10}(\rho^{-1})$	16.11	42.21	0.7229	0.1181	DTR	$[\log_{10}(\rho^{-1})]'$	18.93	24.33	0.7649	0.6055
87	KNR	$[\log_{10}(\rho^{-1})]'$	17.93	42.51	0.6540	0.0448	DTR	ρ_{CR}	22.38	24.45	0.6715	0.5992
88	SVR	ρ	15.88	42.56	0.7317	0.1081	KR	$\log_{10}(\rho^{-1})$	18.01	24.77	0.7883	0.6241

Continued on next page

Table S.20 – Continued from previous page

	Training on 2019–2020 data and testing on 2021–2022 data						Training and testing based on 80%-20% random split of all data					
Rank	Method[1]	Input data	Training %RMSE	Testing %RMSE	Training r^2	Testing r^2	Method[1]	Input data	Training %RMSE	Testing %RMSE	Training r^2	Testing r^2
89	KNR	148 indices	19.80	43.24	0.5775	0.1245	Lasso	$\log_{10}(\rho^{-1})$	22.08	24.96	0.6813	0.5961
90	KNR	$[\log_{10}(\rho^{-1})]''$	19.23	43.31	0.6210	0.0406	KNR	ρ_{CR}	24.13	25.32	0.6620	0.6220
91	KNR	ρ_{CR}	20.22	44.82	0.5769	0.2204	KNR	ρ'	27.35	26.86	0.5512	0.5683
92	DTR	ρ	20.89	45.53	0.5228	0.1034	Ridge	$\log_{10}(\rho^{-1})$	18.88	27.30	0.7673	0.5629
93	DTR	$\log_{10}(\rho^{-1})$	20.60	46.12	0.5362	0.0955	BR	$\log_{10}(\rho^{-1})$	19.98	27.88	0.7393	0.5422
94	SVR	ρ''	2.85	46.15	0.9928	0.0795	KNR	$[\log_{10}(\rho^{-1})]'$	28.90	29.10	0.5216	0.5273
95	SVR	$[\log_{10}(\rho^{-1})]''$	2.86	46.21	0.9929	0.1099	PLSR	$\log_{10}(\rho^{-1})$	19.09	31.36	0.7610	0.4892
96	KNR	ρ	21.65	46.98	0.5103	0.2294	KNR	ρ''	34.93	34.90	0.3921	0.3896
97	KNR	$\log_{10}(\rho^{-1})$	21.70	47.14	0.5087	0.2223	Lasso	ρ_{CR}	20.36	35.23	0.7312	0.3995
98	GPR	ρ	0.00	48.13	1.0000	0.0000	LL	ρ_{CR}	20.38	35.49	0.7307	0.3951
99	GPR	ρ'	0.00	48.13	1.0000	0.0000	KNR	$[\log_{10}(\rho^{-1})]''$	36.31	36.56	0.3441	0.3451
100	GPR	ρ''	0.00	48.13	1.0000	0.0000	GPR	148 indices	0.00	38.12	1.0000	0.1408
101	GPR	$\log_{10}(\rho^{-1})$	0.00	48.13	1.0000	0.0000	GPR	ρ	0.00	38.41	1.0000	0.0009
102	GPR	$[\log_{10}(\rho^{-1})]'$	0.00	48.13	1.0000	0.0000	GPR	ρ'	0.00	38.41	1.0000	0.0000
103	GPR	$[\log_{10}(\rho^{-1})]''$	0.00	48.13	1.0000	0.0000	GPR	ρ''	0.00	38.41	1.0000	0.0000
104	GPR	ρ_{CR}	0.00	48.13	1.0000	0.0000	GPR	$\log_{10}(\rho^{-1})$	0.00	38.41	1.0000	0.0009
105	GPR	148 indices	0.00	48.13	1.0000	0.0010	GPR	$[\log_{10}(\rho^{-1})]'$	0.00	38.41	1.0000	0.0000
106	BR	ρ_{CR}	16.70	51.44	0.6971	0.0055	GPR	$[\log_{10}(\rho^{-1})]''$	0.00	38.41	1.0000	0.0000
107	MLPR	ρ_{CR}	10.78	52.77	0.8798	0.0020	GPR	ρ_{CR}	0.00	38.41	1.0000	0.0000
108	Ridge	ρ_{CR}	16.43	52.96	0.7067	0.0040	BR	ρ_{CR}	18.51	38.84	0.7773	0.3660
109	KR	ρ_{CR}	16.43	52.99	0.7069	0.0039	KR	ρ_{CR}	19.28	39.37	0.7589	0.3492
110	LL	ρ_{CR}	16.67	54.92	0.6986	0.0057	Ridge	ρ_{CR}	19.28	39.37	0.7591	0.3493
111	Lasso	ρ_{CR}	16.64	55.47	0.6996	0.0053	MLPR	ρ_{CR}	12.75	42.66	0.8980	0.3416
112	PLSR	ρ_{CR}	17.26	67.21	0.6745	0.0000	PLSR	ρ_{CR}	20.65	59.82	0.7204	0.1751

[1] AdaBoostRegressor, ABR; BayesianRidge, BR; DecisionTreeRegressor, DTR; GaussianProcessRegressor, GPR; GradientBoostingRegressor, GBR; KernelRidge, KR; KNeighborsRegressor, KNR; LassoLars, LL; MLPRegressor, MLPR; PLSRegression, PLSR; RandomForestRegressor, RFR; and support vector regression, SVR.

Table S.21: Mean permutation importances for using each of 148 spectral vegetation indices to estimate area-basis and mass-basis chlorophyll $a+b$ (Chl $a+b$), chlorophyll a (Chl a), and chlorophyll b (Chl b) with random forest machine learning models. To avoid effects of multicollinearity, indices were grouped into 10 feature sets using hierarchical clustering, and random forest models were fit with one spectral index randomly chosen from each feature set. Iterating over 10,000 unique model fits for each chlorophyll metric ensured each spectral index was evaluated multiple times. Permutation importances were computed as the reduction in model fit score when values of a feature input to random forest models were permuted. The importances of spectral indices are ranked from greatest to least.

	Chl $a+b$ (μg cm^{-2})		Chl a (μg cm^{-2})		Chl b (μg cm^{-2})		Chl $a+b$ (mg g^{-1})		Chl a (mg g^{-1})		Chl b (mg g^{-1})	
Rank	Cluster:Index[1]	Imp.	Cluster:Index	Imp.	Cluster:Index	Imp.	Cluster:Index	Imp.	Cluster: Index	Imp.	Cluster:Index	Imp.
1	G:WUMCARI	1.413	G:WUMCARI	1.302	G:WUMCARI	0.991	G:CPSR2	0.603	C:CRSR3	0.502	G:WLREIP2	0.684
2	G:WUOSAVI	1.299	G:WUOSAVI	1.260	G:WLREIP2	0.961	C:CRSR3	0.526	C:PSSRB	0.443	G:WLCWMRG	0.653
3	G:WLCWMRG	1.260	G:WLCWMRG	1.179	G:WUMOR	0.961	G:GTSR2	0.401	C:PSNDB	0.442	G:WUMCARI	0.612
4	G:WLREIP2	1.233	G:NDVI3	1.171	G:WLCWMRG	0.921	C:PSSRB	0.367	G:CPSR2	0.381	G:MSR2	0.606
5	G:DD	1.199	G:WUMSR	1.169	G:DD	0.860	C:PSNDB	0.366	G:GTSR2	0.288	G:DD	0.594
6	G:WUMOR	1.183	G:DD	1.158	G:MSR2	0.858	G:GSUM1	0.343	B:CAI	0.252	G:WUOSAVI	0.594
7	G:MSR2	1.179	G:WLREIP2	1.156	G:SMNDVI	0.850	G:NDVI3	0.331	C:CAINT	0.249	G:DCNI	0.589
8	G:SMNDVI	1.170	G:GSUM1	1.151	H:CRSR5	0.847	B:CAI	0.321	G:ZTSR2	0.242	G:SMNDVI	0.585
9	G:GSUM1	1.157	G:MSR2	1.117	G:WUOSAVI	0.799	H:CRSR2	0.313	G:CRSR4	0.233	G:MND3	0.565
10	G:DDN	1.146	G:CRSR4	1.115	G:DCNI	0.791	G:WUMSR	0.310	G:GSUM1	0.230	G:WLREIPG	0.545
11	G:NDVI3	1.144	G:SMNDVI	1.106	G:DDN	0.790	G:ZTSR2	0.307	G:WUMSR	0.229	G:CPSR2	0.533
12	G:WUMSR	1.137	G:MND3	1.099	G:MND3	0.783	G:CRSR4	0.303	G:VSR	0.224	G:GSUM1	0.532
13	G:MND3	1.129	G:DDN	1.099	G:WLREIPG	0.751	G:WUOSAVI	0.298	G:NDVI3	0.221	H:MMR	0.520
14	G:GTSR2	1.113	G:ZTSR2	1.085	H:MMR	0.742	G:WLREIP2	0.256	H:CRSR2	0.208	G:MTCI	0.516
15	G:WLREIPG	1.082	G:GTSR2	1.084	G:MND1	0.699	G:VSR	0.251	G:WLREIPG	0.203	G:DDN	0.510
16	G:CRSR4	1.079	G:WUMOR	1.077	G:GSUM1	0.687	G:WLCWMRG	0.250	G:DD	0.201	G:WUMOR	0.510
17	G:DNDR	1.074	G:WLREIPG	1.066	G:DNDR	0.673	G:WLREIPG	0.243	G:WUOSAVI	0.201	G:GTSR2	0.504
18	G:ZTSR2	1.069	G:DNDR	1.056	G:NDVI3	0.668	G:DD	0.237	G:MND1	0.193	G:MND1	0.503
19	G:MTCI	1.052	G:MND1	1.042	G:DDR1	0.664	G:MND1	0.237	G:MND3	0.192	G:NDVI3	0.503
20	G:MND1	1.041	G:MTCI	1.010	G:MTCI	0.662	G:DCNI	0.237	G:MTCI	0.191	G:WUMSR	0.502
21	G:DCNI	1.004	G:VSR	0.987	G:CRSR1	0.662	G:MND3	0.229	G:WLCWMRG	0.188	G:DNDR	0.499
22	G:VSR	0.912	G:DCNI	0.915	G:WUMSR	0.649	G:MTCI	0.221	G:WLREIP2	0.185	B:CAI	0.498
23	G:DDR1	0.828	G:WLREIPE	0.851	G:ZTDP21	0.647	G:DNDR	0.217	G:DNDR	0.179	G:DDR1	0.492
24	G:WLREIPE	0.814	G:VDR	0.837	G:GTSR2	0.637	G:DDN	0.209	G:DDN	0.174	G:CRSR1	0.488
25	G:ZTDP21	0.797	G:DDR1	0.805	G:CRSR4	0.625	G:DDR1	0.203	G:DDR1	0.171	G:CRSR4	0.470
26	G:VDR	0.770	G:ZTDR1	0.772	G:BDR	0.605	H:ZTSR3	0.198	G:DCNI	0.163	G:ZTDP21	0.467
27	G:ZTDR1	0.710	G:ZTDP21	0.685	G:ZTSR2	0.585	G:SMNDVI	0.193	G:MSR2	0.149	G:BDR	0.455
28	G:WLREIP	0.675	G:WLREIP	0.680	G:VSR	0.499	G:MSR2	0.188	G:SMNDVI	0.148	G:ZTSR2	0.443
29	G:BDR	0.645	G:BDR	0.592	G:WLREIPE	0.480	C:CAINT	0.182	G:WLREIPE	0.145	G:VSR	0.394
30	G:CRSR1	0.508	G:CPSR2	0.475	G:BRI2	0.441	H:CI	0.175	G:ZTDR1	0.145	H:ZTSR3	0.365
31	G:CPSR2	0.507	G:DDR2	0.423	G:VDR	0.406	G:WLREIPE	0.161	H:CI	0.141	H:MOR	0.358
32	H:MMR	0.485	G:CRSR1	0.361	H:CPSR1	0.398	G:WUMCARI	0.161	H:ZTSR3	0.140	G:WLREIPE	0.354
33	H:CRSR5	0.465	H:CRSR5	0.285	G:WLREIP	0.393	H:MOR	0.160	G:VDR	0.138	G:VDR	0.340
34	G:BRI2	0.391	G:BRI2	0.285	H:CI	0.392	G:ZTDR1	0.150	I:PRI2	0.134	H:CI	0.326
35	G:DDR2	0.345	G:GRRREM	0.273	G:CPSR2	0.358	G:VDR	0.141	G:WUMCARI	0.128	H:CRSR5	0.322
36	G:GRRREM	0.268	H:MMR	0.266	H:MOR	0.352	H:ZTSR4	0.135	H:ZTDP22	0.111	H:ZTSR1	0.306
37	H:CPSR1	0.213	H:ZTDP22	0.247	G:ZTDR1	0.342	I:PRI2	0.133	J:ARI	0.104	H:CARI	0.303

Continued on next page

Table S.21 – Continued from previous page

	Chl $a+b$ (μg cm^{-2})		Chl a (μg cm^{-2})		Chl b (μg cm^{-2})		Chl $a+b$ (mg g^{-1})		Chl a (mg g^{-1})		Chl b (mg g^{-1})	
Rank	Cluster:Index[1]	Imp.	Cluster:Index	Imp.	Cluster:Index	Imp.	Cluster:Index	Imp.	Cluster: Index	Imp.	Cluster:Index	Imp.
38	H:CI	0.199	C:CRSR3	0.197	H:CARI	0.319	H:ZTSR1	0.132	I:PSRI	0.102	H:MCARI	0.299
39	H:MOR	0.189	H:CI	0.171	H:MCARI	0.315	H:CAR	0.131	H:ZTSR4	0.102	H:ZTSR4	0.257
40	J:ARI	0.187	H:MOR	0.150	J:ARI	0.267	H:MCARI	0.129	H:ZTSR1	0.099	G:WLREIP	0.248
41	H:ZTDP22	0.187	H:CPSR1	0.149	H:ZTSR3	0.256	H:MMR	0.123	G:DDR2	0.097	G:ZTDR1	0.237
42	C:CRSR3	0.186	H:MCARI	0.145	G:GRRREM	0.205	G:DPI	0.123	G:WUMOR	0.091	G:DPI	0.215
43	H:MCARI	0.183	J:ARI	0.129	G:DDR2	0.202	H:CARI	0.121	J:EGFN	0.090	G:BRI2	0.206
44	H:CARI	0.176	H:CARI	0.126	H:ZTSR4	0.174	I:PSRI	0.111	G:DPI	0.085	H:CRSR2	0.199
45	H:ZTSR3	0.121	J:BMSR	0.101	H:AIVI	0.159	H:CRSR5	0.110	G:CRSR1	0.085	H:ZTSR5	0.192
46	H:TCI	0.103	H:ZTDPR1	0.090	H:BMDVI	0.150	G:WUMOR	0.110	H:MMR	0.083	B:MSI	0.190
47	H:BMDVI	0.101	H:ZTSR3	0.086	B:CAI	0.150	G:CRSR1	0.104	B:NDNI	0.079	H:CAR	0.179
48	H:AIVI	0.098	H:AIVI	0.085	H:TCI	0.132	H:WUTOR	0.097	H:MCARI	0.077	G:BRI1	0.172
49	H:CAR	0.097	J:MND2	0.084	H:ZTSR5	0.129	B:MSI	0.095	H:ZTSR5	0.077	H:AIVI	0.157
50	H:ZTDPR1	0.092	H:TOR	0.084	H:CAR	0.120	G:ZTDP21	0.091	H:CARI	0.076	H:CPSR1	0.154
51	H:ZTSR4	0.089	H:CAR	0.078	H:DREIP	0.117	H:ZTDP22	0.087	H:MOR	0.076	B:LCA	0.154
52	H:TCARI	0.089	H:TCI	0.077	A:EVI	0.112	H:ZTSR5	0.086	H:CAR	0.074	C:PSNDB	0.135
53	H:BD	0.080	H:ZTSR5	0.076	G:BRI1	0.102	H:CPSR1	0.083	H:WUTOR	0.071	F:NPQI	0.134
54	H:ZTDPR2	0.079	H:WUTOR	0.075	H:ZTSR1	0.102	G:DDR2	0.082	H:CRSR5	0.071	C:PSSRB	0.133
55	J:BMLSR	0.075	J:GNDVI	0.074	G:DPI	0.099	G:BDR	0.080	H:CPSR1	0.069	H:TCI	0.130
56	J:GNDVI	0.074	J:PRI	0.073	J:CVI	0.095	J:ARI	0.076	H:ZTDPR1	0.068	B:NDWI	0.122
57	H:TOR	0.073	H:BMDVI	0.073	C:CRSR3	0.090	J:EGFN	0.075	I:PRI3	0.067	I:PSRI	0.121
58	H:DREIP	0.073	H:ZTSR4	0.072	H:TCARI	0.085	H:AIVI	0.069	B:MSI	0.066	B:SRWI	0.120
59	H:ZTSR5	0.072	J:BMLSR	0.072	H:SPVI	0.081	G:WLREIP	0.068	F:SRPI	0.061	G:GRRREM	0.120
60	A:EVI	0.069	J:GRRGM	0.069	H:ZTDPR2	0.079	B:WI	0.067	F:NDPI	0.060	I:PRI2	0.118
61	H:ZTSR6	0.067	H:ZTDPR2	0.069	B:LCA	0.078	B:PSR	0.067	J:BGI2	0.060	G:DDR2	0.107
62	G:DPI	0.066	H:ZTSR6	0.068	H:ZTDP22	0.077	B:NDWI	0.063	F:NPCI	0.060	H:TOR	0.106
63	J:BMSR	0.064	J:MND4	0.066	H:ZTSR6	0.075	B:SRWI	0.062	J:PRI	0.060	H:ZTSR6	0.100
64	J:GRRGM	0.064	J:GTSR1	0.062	J:MND2	0.073	H:TOR	0.061	H:BMDVI	0.060	J:ARI	0.100
65	J:MND4	0.063	H:TCARI	0.060	H:TOR	0.073	F:NDPI	0.056	A:EVI	0.060	H:TCARI	0.094
66	H:ZTSR1	0.060	H:BD	0.058	F:NPQI	0.072	B:NDNI	0.055	H:AIVI	0.060	C:CRSR3	0.092
67	J:PRI	0.059	J:DND	0.054	A:FSUM	0.072	H:BMDVI	0.052	H:ZTDPR2	0.058	H:DREIP	0.090
68	A:FSUM	0.059	H:ZTSR1	0.050	A:DVI	0.070	H:TCARI	0.051	J:RGI	0.058	B:PSR	0.088
69	J:MND2	0.059	A:EVI	0.049	D:CRI700	0.070	F:NPQI	0.051	J:MND2	0.055	B:WI	0.087
70	H:WUTOR	0.058	J:CVI	0.048	J:VARI	0.068	I:PRI3	0.051	J:CVI	0.054	F:NDPI	0.085
71	J:DND	0.058	G:DPI	0.047	J:MND4	0.066	F:NPCI	0.051	J:DND	0.051	H:BD	0.079
72	F:SIPI	0.057	H:DREIP	0.045	H:BD	0.066	H:TCI	0.050	F:NPQI	0.049	H:BMDVI	0.073
73	A:PVI	0.056	C:CAINT	0.043	J:DSR2	0.065	F:SRPI	0.050	J:GMSR	0.049	H:ZTDP22	0.073
74	A:GEMI	0.055	J:GSUM2	0.042	J:DSR1	0.064	J:DND	0.048	H:ZTSR6	0.048	J:BGI2	0.071
75	A:DVI	0.055	J:RGI	0.038	H:ZTDPR1	0.064	H:ZTSR6	0.048	J:MND4	0.048	J:DND	0.069
76	A:ZTSUM	0.054	H:CRSR2	0.038	J:BGI1	0.064	J:BGI2	0.046	H:TOR	0.047	H:ZTDPR2	0.066
77	H:CRSR2	0.053	A:PVI	0.038	A:ZTSUM	0.064	H:ZTDPR1	0.046	J:NDVI2	0.047	J:DSR1	0.064
78	A:WDVI	0.053	A:FSUM	0.038	A:PVI	0.064	J:RGI	0.044	J:GSUM2	0.047	J:BMLSR	0.062
79	J:GTSR1	0.053	F:SIPI	0.038	A:WDVI	0.064	H:ZTDPR2	0.044	H:SPVI	0.046	J:EGFN	0.061
80	H:ESUM2	0.051	A:GEMI	0.037	H:ESUM2	0.063	B:PD	0.042	J:DSR2	0.046	J:MND2	0.059
81	A:EVI2	0.046	A:DVI	0.037	J:GI	0.062	J:CVI	0.041	B:PSR	0.045	J:VARI	0.059
82	J:CVI	0.045	D:CRI700	0.037	J:GMSR	0.060	J:GRRGM	0.040	B:WI	0.045	J:MND4	0.059

Continued on next page

Table S.21 – Continued from previous page

Rank	Chl $a+b$ (μg cm^{-2})		Chl a (μg cm^{-2})		Chl b (μg cm^{-2})		Chl $a+b$ (mg g^{-1})		Chl a (mg g^{-1})		Chl b (mg g^{-1})	
	Cluster:Index[1]	Imp.	Cluster:Index	Imp.	Cluster:Index	Imp.	Cluster:Index	Imp.	Cluster: Index	Imp.	Cluster:Index	Imp.
83	A:MSAVI2	0.043	A:WDVI	0.036	J:NDVI2	0.059	J:BMLSR	0.040	A:FSUM	0.045	H:ZTDPR1	0.059
84	A:RDVI	0.043	J:CRI500	0.036	J:BGI2	0.059	J:MND2	0.039	H:TCI	0.044	B:PD	0.056
85	A:RVIOPT	0.042	I:PRI3	0.036	I:PRI2	0.058	J:PRI	0.039	F:SIPI	0.043	J:CVI	0.053
86	A:SAVI	0.042	C:PSNDB	0.035	I:PSRI	0.057	J:GMSR	0.039	A:DVI	0.042	J:GRRGM	0.053
87	H:SPVI	0.042	A:EVI2	0.035	A:GEMI	0.055	J:GTSR1	0.038	J:GI	0.042	J:GI	0.052
88	A:MSAVI1	0.041	A:ZTSUM	0.034	J:DND	0.054	J:VARI	0.037	A:ESUM1	0.042	J:TGI	0.052
89	J:RGI	0.041	J:BGI2	0.034	J:GRRGM	0.052	F:SIPI	0.036	J:GNDVI	0.041	J:BGI1	0.051
90	G:BRI1	0.040	A:SAVI	0.034	A:EVI2	0.051	J:BMSR	0.036	J:BGI1	0.040	J:GNDVI	0.051
91	J:GSUM2	0.038	C:PSSRB	0.034	J:RGI	0.051	J:NDVI2	0.035	A:ZTSUM	0.040	J:GMSR	0.051
92	I:PSRI	0.037	A:MSAVI2	0.033	A:RDVI	0.048	J:GNDVI	0.035	J:GTSR1	0.040	J:DSR2	0.051
93	C:PSNDB	0.037	A:RVIOPT	0.032	B:MSI	0.047	J:DSR2	0.035	A:PVI	0.040	H:ESUM2	0.049
94	D:CRI700	0.036	J:TGI	0.032	H:CRSR2	0.046	D:CPSR3	0.035	J:BMLSR	0.040	J:NDVI2	0.048
95	C:PSSRB	0.036	A:RDVI	0.032	F:SIPI	0.046	A:EVI	0.034	A:WDVI	0.040	B:NDNI	0.045
96	C:CAINT	0.035	I:PRI2	0.031	J:BMLSR	0.046	G:BRI2	0.034	D:CPSR3	0.039	J:BMSR	0.042
97	J:VARI	0.035	J:EGFN	0.031	A:SAVI	0.045	J:GSUM2	0.034	J:VARI	0.039	J:GTSR1	0.041
98	F:NPQI	0.034	J:GMSR	0.030	J:EGFN	0.045	J:MND4	0.034	H:ESUM2	0.039	F:SRPI	0.040
99	J:GMSR	0.034	J:NDVI2	0.030	A:RVIOPT	0.044	H:SPVI	0.032	G:WLREIP	0.039	F:NPCI	0.039
100	J:TGI	0.033	A:MSAVI1	0.030	J:GNDVI	0.044	J:GI	0.031	J:GRRGM	0.039	H:WUTOR	0.038
101	A:ESUM1	0.033	J:WUTCARI	0.030	F:NDPI	0.043	H:BD	0.030	J:BMSR	0.039	J:RGI	0.038
102	J:BGI2	0.032	J:GI	0.029	A:MSAVI2	0.042	J:CRI500	0.030	D:PSNDC	0.039	J:MTVI2	0.037
103	J:DSR1	0.032	H:ESUM2	0.029	J:GTSR1	0.042	G:GRRREM	0.029	J:CRI500	0.038	J:MCARI2	0.036
104	J:GI	0.031	J:DSR2	0.028	B:NDWI	0.040	G:BRI1	0.029	B:SRWI	0.038	I:PRI3	0.036
105	J:DSR2	0.031	B:CAI	0.028	J:TGI	0.040	D:CRI700	0.028	D:CRI700	0.037	H:SPVI	0.034
106	J:NDVI2	0.031	J:DSR1	0.027	J:MCARI2	0.040	D:PSNDC	0.028	B:NDWI	0.037	D:CRI700	0.034
107	I:PRI2	0.030	H:SPVI	0.027	J:MTVI2	0.040	D:PSSRC	0.028	A:GEMI	0.037	D:CPSR3	0.034
108	J:CRI500	0.029	G:BRI1	0.027	A:ESUM1	0.040	J:DSR1	0.026	H:BD	0.037	C:CAINT	0.032
109	B:CAI	0.029	D:PSNDA	0.026	D:NDVI	0.040	J:TGI	0.025	B:PD	0.036	A:EVI	0.029
110	J:EGFN	0.027	D:JSR	0.025	D:BRSR	0.040	B:LCA	0.024	D:PSSRC	0.036	D:BRSR	0.029
111	J:MTVI2	0.026	I:PSRI	0.025	B:SRWI	0.039	A:ESUM1	0.024	H:TCARI	0.035	D:PSSRC	0.029
112	J:WUTCARI	0.026	D:PSSRA	0.025	J:BMSR	0.038	A:FSUM	0.023	A:RVIOPT	0.034	D:PSNDC	0.028
113	J:MCARI1	0.026	D:CPSR3	0.025	A:MSAVI1	0.037	H:ESUM2	0.023	G:ZTDP21	0.034	D:WDRVI2	0.028
114	J:MTVI1	0.026	B:NDLI	0.024	D:WDRVI	0.036	J:BGI1	0.023	A:EVI2	0.034	D:MSR	0.028
115	J:MCARI2	0.025	J:VARI	0.024	H:WUTOR	0.036	A:DVI	0.022	A:RDVI	0.034	F:SIPI	0.028
116	I:PRI3	0.025	A:ESUM1	0.023	I:PRI3	0.036	D:BRSR	0.022	G:BDR	0.033	D:JSR	0.028
117	B:NDLI	0.023	D:PSNDC	0.022	J:MCARI1	0.035	A:WDVI	0.021	J:TGI	0.033	D:WDRVI	0.028
118	D:PSNDA	0.023	D:NDVI	0.022	J:MTVI1	0.035	B:NDLI	0.021	A:SAVI	0.032	J:GSUM2	0.027
119	F:NDPI	0.022	J:MTVI2	0.022	D:TSAVI	0.035	D:MSR	0.021	G:BRI2	0.032	D:NDVI	0.026
120	D:CPSR3	0.022	D:WNR	0.022	D:JSR	0.035	A:ZTSUM	0.021	J:WUTCARI	0.029	D:TSAVI	0.024
121	D:WDRVI2	0.022	D:MSR	0.020	D:MSR	0.034	A:PVI	0.021	D:WNR	0.029	D:PSSRA	0.023
122	B:LCA	0.021	D:TSAVI	0.020	D:PSSRC	0.034	A:RVIOPT	0.021	J:DSR1	0.027	D:PSNDA	0.022
123	D:TSAVI	0.021	D:BRSR	0.020	J:CRI500	0.032	D:WNR	0.021	A:MSAVI2	0.027	J:MTVI1	0.022
124	D:JSR	0.021	D:WDRVI	0.020	D:WDRVI2	0.032	A:GEMI	0.020	G:BRI1	0.027	D:SAVI2	0.022
125	D:WDRVI	0.021	D:PSSRC	0.020	D:PSNDC	0.031	A:SAVI	0.020	D:NDVI	0.026	J:CRI500	0.022
126	D:BRSR	0.020	D:WDRVI2	0.019	D:PSNDA	0.030	D:WDRVI2	0.020	D:WDRVI2	0.026	J:MCARI1	0.021
127	D:MSR	0.020	J:MCARI2	0.019	D:CPSR3	0.030	D:NDVI	0.020	A:MSAVI1	0.026	A:FSUM	0.021

Continued on next page

Table S.21 – Continued from previous page

	Chl $a+b$ (μg cm^{-2})		Chl a (μg cm^{-2})		Chl b (μg cm^{-2})		Chl $a+b$ (mg g^{-1})		Chl a (mg g^{-1})		Chl b (mg g^{-1})	
Rank	Cluster:Index[1]	Imp.	Cluster:Index	Imp.	Cluster:Index	Imp.	Cluster:Index	Imp.	Cluster: Index	Imp.	Cluster:Index	Imp.
128	D:NDVI	0.020	F:NPQI	0.019	J:GSUM2	0.029	A:RDVI	0.020	D:WDRVI	0.026	B:NDLI	0.021
129	D:PSSRA	0.020	J:MCARI1	0.018	B:WI	0.028	D:JSR	0.020	D:MSR	0.025	D:OSAVI	0.021
130	D:WNR	0.019	F:NDPI	0.018	B:PSR	0.028	D:WDRVI	0.020	D:BRSR	0.025	A:DVI	0.020
131	D:PSSRC	0.018	B:NDNI	0.017	D:PSSRA	0.028	J:WUTCARI	0.020	D:JSR	0.025	D:NLI	0.019
132	J:BGI1	0.018	J:MTVI1	0.016	C:PSSRB	0.027	A:EVI2	0.020	B:NDLI	0.024	J:TVI	0.019
133	J:TVI	0.018	D:SAVI2	0.015	J:PRI	0.027	D:PSNDA	0.019	H:DREIP	0.023	D:TSAVI2	0.019
134	D:PSNDC	0.017	J:BGI1	0.014	J:TVI	0.026	D:TSAVI	0.019	D:TSAVI	0.023	A:PVI	0.019
135	F:SRPI	0.016	B:WI	0.013	D:WNR	0.026	A:MSAVI2	0.018	D:PSNDA	0.022	D:WNR	0.019
136	F:NPCI	0.016	B:LCA	0.013	J:WUTCARI	0.026	D:PSSRA	0.018	D:PSSRA	0.022	J:PRI	0.019
137	D:SAVI2	0.016	B:PSR	0.013	C:PSNDB	0.026	A:MSAVI1	0.017	G:GRRREM	0.016	A:ZTSUM	0.019
138	D:OSAVI	0.013	F:SRPI	0.013	F:SRPI	0.024	H:DREIP	0.014	B:LCA	0.015	A:WDVI	0.019
139	B:PSR	0.013	F:NPCI	0.013	F:NPCI	0.024	J:MTVI2	0.011	J:MTVI2	0.013	A:MSAVI2	0.019
140	D:TSAVI2	0.013	J:TVI	0.012	D:SAVI2	0.023	J:MCARI2	0.011	J:MCARI2	0.012	A:GEMI	0.018
141	B:WI	0.013	D:OSAVI	0.010	C:CAINT	0.019	D:SAVI2	0.010	D:SAVI2	0.011	A:SAVI	0.018
142	D:NLI	0.012	B:MSI	0.010	D:OSAVI	0.019	J:TVI	0.007	J:MCARI1	0.011	A:ESUM1	0.017
143	B:NDNI	0.011	D:TSAVI2	0.009	D:TSAVI2	0.019	D:NLI	0.006	J:MTVI1	0.011	A:MSAVI1	0.017
144	B:MSI	0.008	D:NLI	0.009	D:NLI	0.018	J:MCARI1	0.006	J:TVI	0.011	J:WUTCARI	0.017
145	B:NDWI	0.008	B:PD	0.008	B:NDLI	0.018	J:MTVI1	0.006	D:NLI	0.007	A:EVI2	0.016
146	B:SRWI	0.008	B:NDWI	0.007	B:NDNI	0.016	D:OSAVI	0.006	D:OSAVI	0.006	A:RDVI	0.016
147	B:PD	0.005	B:SRWI	0.006	B:PD	0.007	D:TSAVI2	0.005	D:TSAVI2	0.006	A:RVIOPT	0.016
148	E:WLPD	0.001	E:WLPD	0.000	E:WLPD	0.000	E:WLPD	-0.002	E:WLPD	-0.002	E:WLPD	0.001

[1] See Table S.1 for definitions and formulations of spectral vegetation indices.

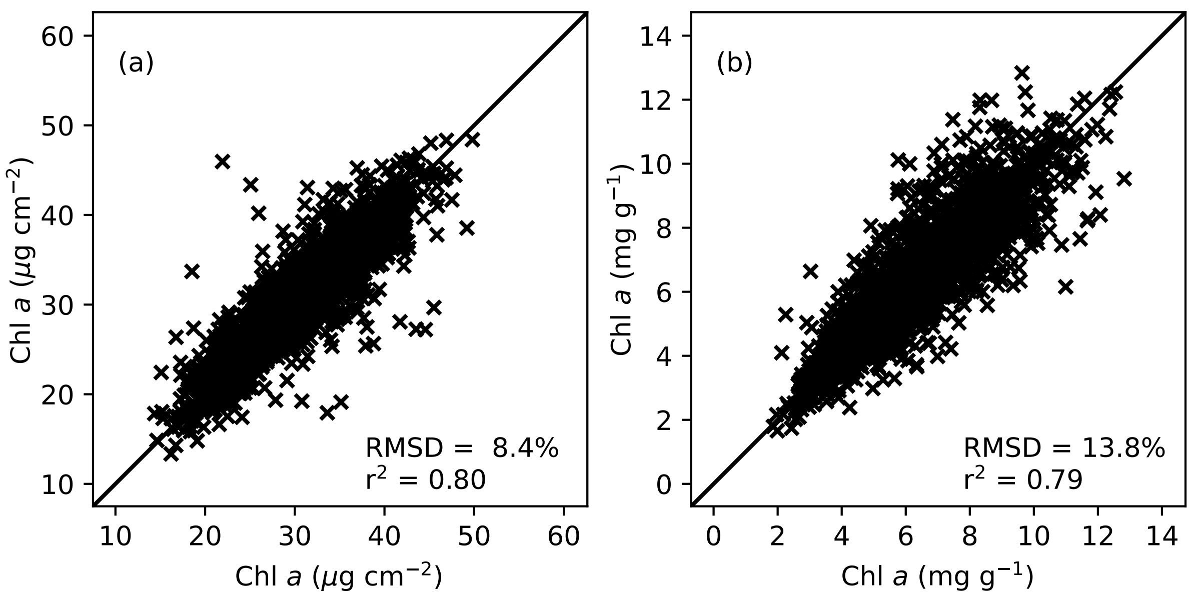

Figure S.1: Comparison of cotton leaf chlorophyll *a* (Chl *a*) extractions among paired tissue samples from the same cotton leaf (n=2,916) in units of a) μg cm^{-2} for area-basis estimates and b) mg g^{-1} for mass-basis estimates. Samples were collected during a 2019-2020 field study at Maricopa, Arizona.

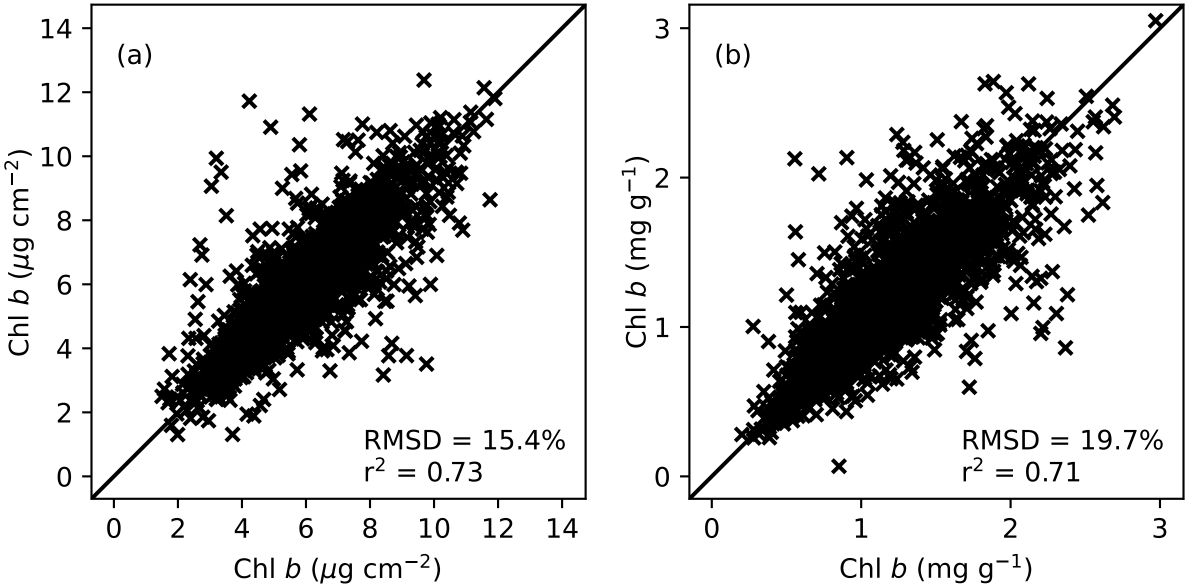

Figure S.2: Comparison of cotton leaf chlorophyll *b* (Chl *b*) extractions among paired tissue samples from the same cotton leaf (n=2,916) in units of a) μg cm^{-2} for area-basis estimates and b) mg g^{-1} for mass-basis estimates. Samples were collected during a 2019-2020 field study at Maricopa, Arizona.

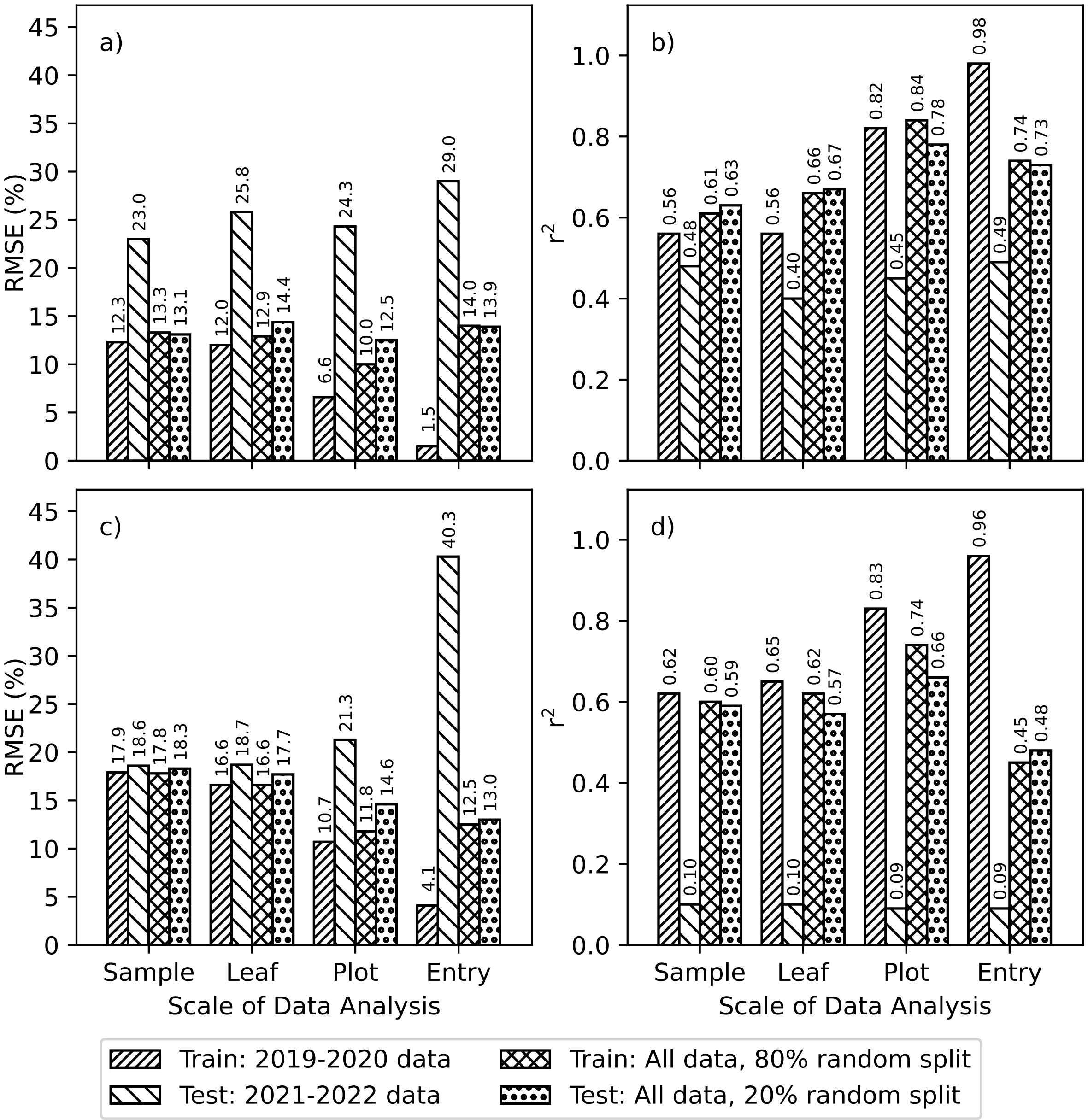

Figure S.3: Goodness-of-fit statistics for partial least squares regression (PLSR) models that were fit using cotton leaf chlorophyll *a* (Chl *a*) and spectral reflectance data at four different scales (i.e., sample, leaf, plot, and entry) and using two methods to split the data for training and testing phases (i.e., by experiment and by using an 80% and 20% random split of combined data from both experiments). Results are shown as a) root mean squared errors (RMSE) and b) coefficients of determination (r^2) for area-basis Chl *a* (μg cm^{-2}) and c) RMSE and d) r^2 for mass-basis Chl *a* (mg g^{-1}).

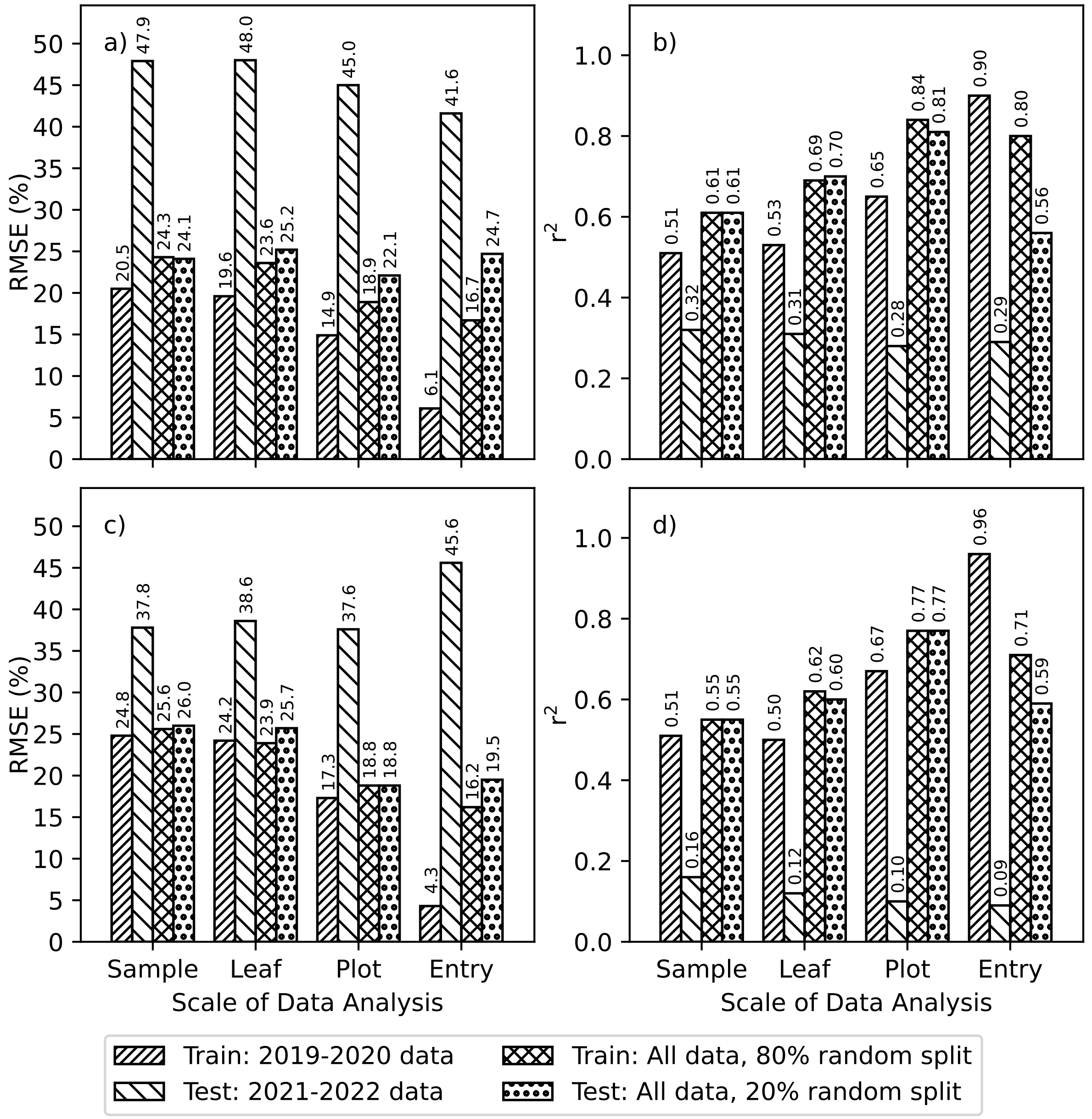

Figure S.4: Goodness-of-fit statistics for partial least squares regression (PLSR) models that were fit using cotton leaf chlorophyll *b* (Chl *b*) and spectral reflectance data at four different scales (i.e., sample, leaf, plot, and entry) and using two methods to split the data for training and testing phases (i.e., by experiment and by using an 80% and 20% random split of combined data from both experiments). Results are shown as a) root mean squared errors (RMSE) and b) coefficients of determination (r^2) for area-basis Chl *b* (μg cm^{-2}) and c) RMSE and d) r^2 for mass-basis Chl *b* (mg g^{-1}).

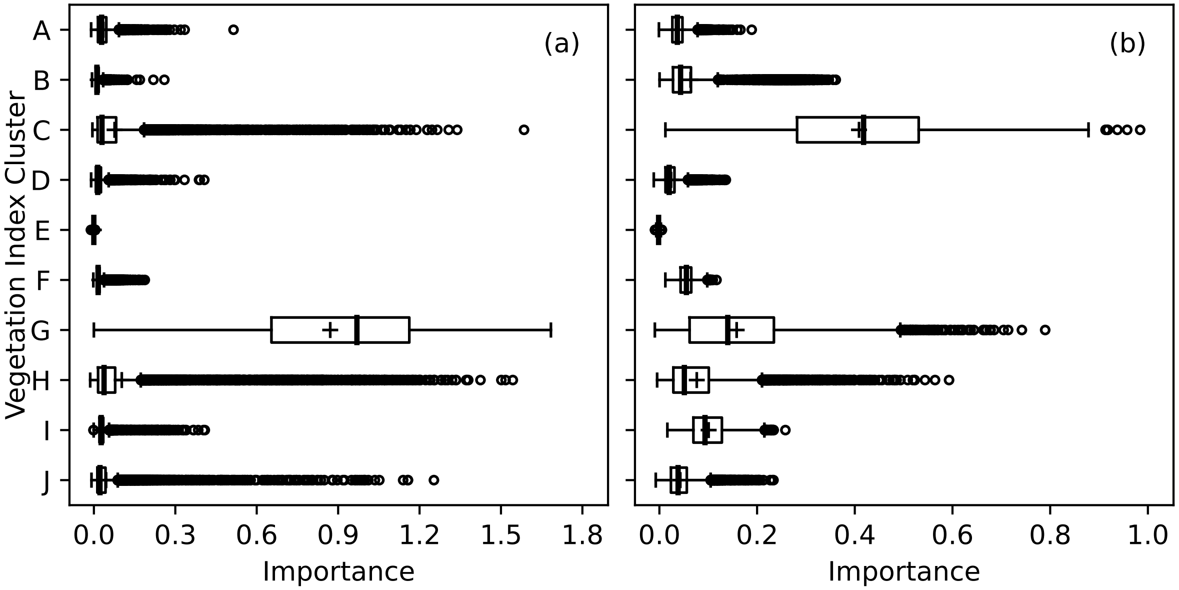

Figure S.5: Permutation importances (computed as the reduction in model fit score when values of a feature input to random forest models were permuted) among 10 clusters of 148 spectral vegetation indices for estimation of a) area-basis chlorophyll a (μg cm^{-2}) and b) mass-basis chlorophyll a (mg g^{-1}).

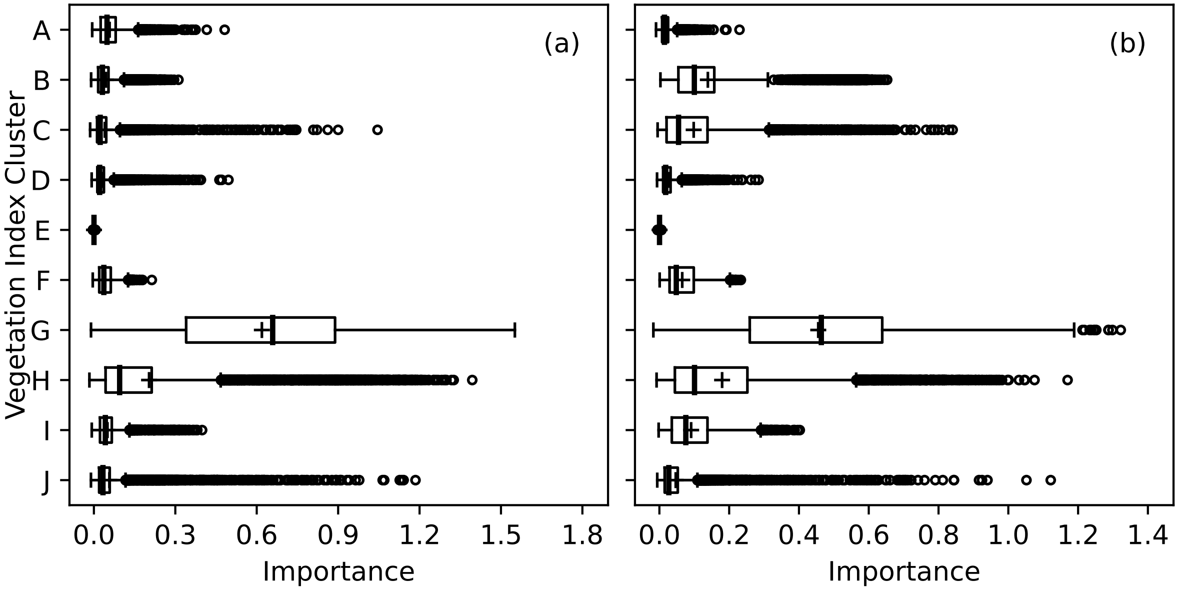

Figure S.6: Permutation importances (computed as the reduction in model fit score when values of a feature input to random forest models were permuted) among 10 clusters of 148 spectral vegetation indices for estimation of a) area-basis chlorophyll b (μg cm^{-2}) and b) mass-basis chlorophyll b (mg g^{-1}).

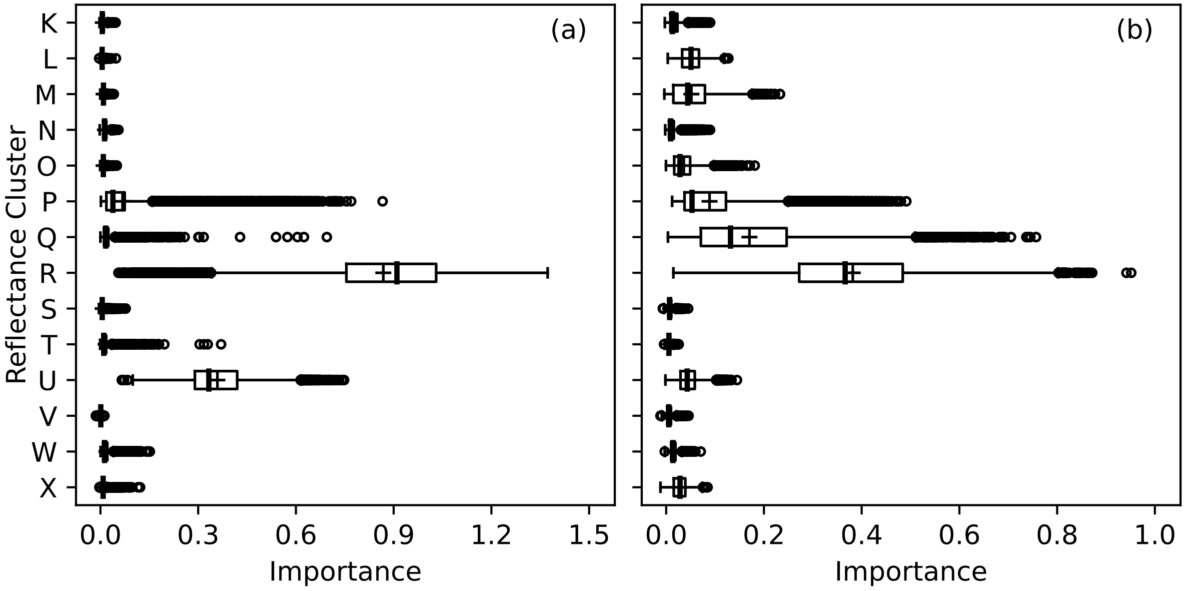

Figure S.7: Permutation importances (computed as the reduction in model fit score when values of a feature input to random forest models were permuted) among 14 clusters of 2151 spectral reflectance wavebands at 350-2500 nm for estimation of a) area-basis chlorophyll a (μg cm^{-2}) and b) mass-basis chlorophyll a (mg g^{-1}).

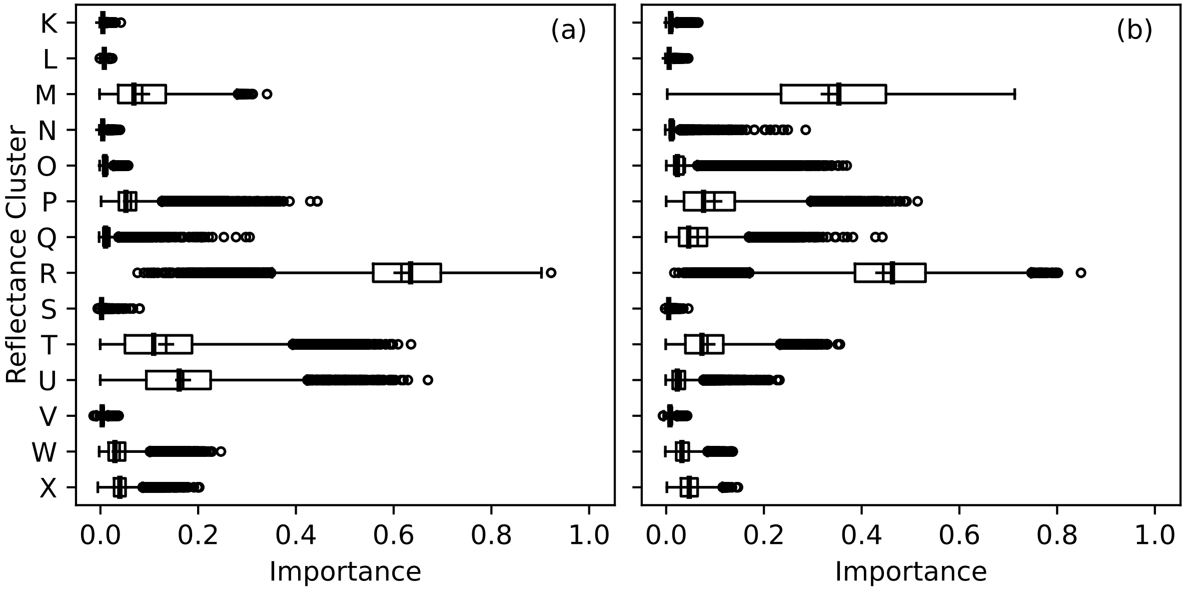

Figure S.8: Permutation importances (computed as the reduction in model fit score when values of a feature input to random forest models were permuted) among 14 clusters of 2151 spectral reflectance wavebands at 350-2500 nm for estimation of a) area-basis chlorophyll b (μg cm^{-2}) and b) mass-basis chlorophyll b (mg g^{-1}).

References

Baret, F., Guyot, D., and Major, D. J. (1989). TSAVI: A vegetation index which minimizes soil brightness effects on LAI and APAR estimation. In *Proceedings of the 12th Canadian Symposium on Remote Sensing, IGARSS '89*, volume 3, pages 1355–1358, Piscataway, NJ, USA. Vancouver, Canada, 10-14 July, IEEE.

Baret, F. and Guyot, G. (1991). Potentials and limits of vegetation indices for LAI and APAR assessment. *Remote Sensing of Environment*, 35(2–3):161–173.

Birth, G. S. and McVey, G. R. (1968). Measuring the color of growing turf with a reflectance spectrophotometer. *Agronomy Journal*, 60(6):640–643.

Blackburn, G. A. (1998a). Quantifying chlorophylls and carotenoids at leaf and canopy scales: An evaluation of some hyperspectral approaches. *Remote Sensing of Environment*, 66:273–285.

Blackburn, G. A. (1998b). Spectral indices for estimating photosynthetic pigment concentrations: A test using senescent tree leaves. *International Journal of Remote Sensing*, 19(4):657–675.

Boochs, F., Kupfer, G., Dockter, K., and Kühbauch, W. (1990). Shape of the red edge as vitality indicator for plants. *International Journal of Remote Sensing*, 11(10):1741–1753.

Broge, N. H. and Leblanc, E. (2000). Comparing prediction power and stability of broadband and hyperspectral vegetation indices for estimation of green leaf area index and canopy chlorophyll density. *Remote Sensing of Environment*, 76(2):156–172.

Buschmann, C. and Nagel, E. (1993). In vivo spectroscopy and internal optics of leaves as basis for remote sensing of vegetation. *International Journal of Remote Sensing*, 14(4):711–722.

Carter, G. A. (1994). Ratios of leaf reflectances in narrow wavebands as indicators of plant stress. *International Journal of Remote Sensing*, 15(3):697–703.

Chappelle, E. W., Kim, M. S., and McMurtrey III, J. E. (1992). Ratio analysis of reflectance spectra (RARS): An algorithm for the remote estimation of the concentrations of chlorophyll A, chlorophyll B, and carotenoids in soybean leaves. *Remote Sensing of Environment*, 39(3):239–247.

Chen, J. M. (1996). Evaluation of vegetation indices and a modified simple ratio for boreal applications. *Canadian Journal of Remote Sensing*, 22(3):229–242.

Chen, P., Haboudane, D., Tremblay, N., Wang, J., Vigneault, P., and Li, B. (2010). New spectral indicator assessing the efficiency of crop nitrogen treatment in corn and wheat. *Remote Sensing of Environment*, 114(9):1987–1997.

Cho, M. A. and Skidmore, A. K. (2006). A new technique for extracting the red edge position from hyperspectral data: The linear extrapolation method. *Remote Sensing of Environment*, 101(2):181–193.

Clevers, J. G. P. W. (1989). Application of a weighted infrared-red vegetation index for estimating leaf area index by correcting for soil moisture. *Remote Sensing of Environment*, 29(1):25–37.

Collins, W. (1978). Remote sensing of crop type and maturity. *Photogrammetric Engineering and Remote Sensing*, 44(1):43–55.

Dash, J. and Curran, P. J. (2004). The MERIS terrestrial chlorophyll index. *International Journal of Remote Sensing*, 25(23):5403–5413.

Datt, B. (1998). Remote sensing of chlorophyll a, chlorophyll b, chlorophyll a+b, and total carotenoid content in eucalyptus leaves. *Remote Sensing of Environment*, 66(2):111–121.

Datt, B. (1999a). A new reflectance index for remote sensing of chlorophyll content in higher plants: Tests using Eucalyptus leaves. *Journal of Plant Physiology*, 154(1):30–36.

Datt, B. (1999b). Visible/near infrared reflectance and chlorophyll content in eucalyptus leaves. *International Journal of Remote Sensing*, 20(14):2741–2759.

Daughtry, C. S. T. (2001). Discriminating crop residues from soil by shortwave infrared reflectance. *Agronomy Journal*, 93(1):125–131.

Daughtry, C. S. T., Hunt, Jr., E. R., Doraiswamy, P. C., and McMurtrey III, J. E. (2005). Remote sensing the spatial distribution of crop residues. *Agronomy Journal*, 97(3):864–871.

Daughtry, C. S. T., Walthall, C. L., Kim, M. S., De Colstoun, E. B., and McMurtrey, III, J. E. (2000). Estimating corn leaf chlorophyll concentration from leaf and canopy reflectance. *Remote Sensing of Environment*, 74(2):229–239.

Eitel, J. U. H., Long, D. S., Gessler, P. E., and Smith, A. M. S. (2007). Using in-situ measurements to evaluate the new RapidEye™ satellite series for prediction of wheat nitrogen status. *International Journal of Remote Sensing*, 28(18):4183–4190.

Elvidge, C. D. and Chen, Z. (1995). Comparison of broad-band and narrow-band red and near-infrared vegetation indices. *Remote Sensing of Environment*, 54:38–48.

Filella, I., Amaro, T., Araus, J. L., and Peñuelas, J. (1996). Relationship between photosynthetic radiation-use efficiency of barley canopies and the photochemical reflectance index (PRI). *Physiologia Plantarum*, 96(2):211–216.

Filella, I. and Peñuelas, J. (1994). The red edge position and shape as indicators of plant chlorophyll content, biomass and hydric status. *International Journal of Remote Sensing*, 15(7):1459–1470.

Filella, I., Serrano, L., Serra, J., and Peñuelas, J. (1995). Evaluating wheat nitrogen status with canopy reflectance indices and discriminant analysis. *Crop Science*, 35(5):1400–1405.

Gamon, J. A., Peñuelas, J., and Field, C. B. (1992). A narrow-waveband spectral index that tracks diurnal changes in photosynthetic efficiency. *Remote Sensing of Environment*, 41(1):35–44.

Gamon, J. A. and Surfus, J. S. (1999). Assessing leaf pigment content and activity with a reflectometer. *New Phytologist*, 143(1):105–117.

Gao, B. (1996). NDWI - A normalized difference water index for remote sensing of vegetation liquid water from space. *Remote Sensing of Environment*, 58(3):257–266.

Gitelson, A. and Merzlyak, M. N. (1994). Quantitative estimation of chlorophyll-a using reflectance spectra: Experiments with autumn chestnut and maple leaves. *Journal of Photochemistry and Photobiology, B: Biology*, 22(3):247–252.

Gitelson, A. A. (2004). Wide dynamic range vegetation index for remote quantification of biophysical characteristics of vegetation. *Journal of Plant Physiology*, 161(2):165–173.

Gitelson, A. A., Gritz, Y., and Merzlyak, M. N. (2003). Relationships between leaf chlorophyll content and spectral reflectance and algorithms for non-destructive chlorophyll assessment in higher plant leaves. *Journal of Plant Physiology*, 160(3):271–282.

Gitelson, A. A., Kaufman, Y. J., and Merzlyak, M. N. (1996). Use of a green channel in remote sensing of global vegetation from EOS-MODIS. *Remote Sensing of Environment*, 58(3):289–298.

Gitelson, A. A., Kaufman, Y. J., Stark, R., and Rundquist, D. (2002a). Novel algorithms for remote estimation of vegetation fraction. *Remote Sensing of Environment*, 80(1):76–87.

Gitelson, A. A. and Merzlyak, M. N. (1996). Signature analysis of leaf reflectance spectra: Algorithm development for remote sensing of chlorophyll. *Journal of Plant Physiology*, 148(3-4):494–500.

Gitelson, A. A. and Merzlyak, M. N. (1997). Remote estimation of chlorophyll content in higher plant leaves. *International Journal of Remote Sensing*, 18(12):2691–2697.

Gitelson, A. A., Merzlyak, M. N., and Chivkunova, O. B. (2001). Optical properties and nondestructive estimation of anthocyanin content in plant leaves. *Photochemistry and Photobiology*, 74(1):38–45.

Gitelson, A. A., Viña, A., Ciganda, V., Rundquist, D. C., and Arkebauer, T. J. (2005). Remote estimation of canopy chlorophyll content in crops. *Geophysical Research Letters*, 32(8):1–4.

Gitelson, A. A., Zur, Y., Chivkunova, O. B., and Merzlyak, M. N. (2002b). Assessing carotenoid content in plant leaves with reflectance spectroscopy. *Photochemistry and Photobiology*, 75(3):272–281.

Goel, N. S. and Qin, W. (1994). Influences of canopy architecture on relationships between various vegetation indices and LAI and FPAR: A computer simulation. *Remote Sensing Reviews*, 10(4):309–347.

Guyot, G. and Baret, F. (1988). Utilisation de la haute resolution spectrale pour suivre l'etat des couverts vegetaux. In *Spectral Signatures of Objects in Remote Sensing*, Aussois (Modane), France. European Space Agency.

Haboudane, D., Miller, J. R., Pattey, E., Zarco-Tejada, P. J., and Strachan, I. B. (2004). Hyperspectral vegetation indices and novel algorithms for predicting green LAI of crop canopies: Modeling and validation in the context of precision agriculture. *Remote Sensing of Environment*, 90(3):337–352.

Haboudane, D., Miller, J. R., Tremblay, N., Zarco-Tejada, P. J., and Dextraze, L. (2002). Integrated narrow-band vegetation indices for prediction of crop chlorophyll content for application to precision agriculture. *Remote Sensing of Environment*, 81(2-3):416–426.

Haboudane, D., Tremblay, N., Miller, J. R., and Vigneault, P. (2008). Remote estimation of crop chlorophyll content using spectral indices derived from hyperspectral data. *IEEE Transactions on Geoscience and Remote Sensing*, 46(2):423–436.

He, L., Song, X., Feng, W., Guo, B., Zhang, Y., Wang, Y., Wang, C., and Guo, T. (2016). Improved remote sensing of leaf nitrogen concentration in winter wheat using multi-angular hyperspectral data. *Remote Sensing of Environment*, 174:122–133.

Horler, D. N. H., Dockray, M., and Barber, J. (1983). The red edge of plant leaf reflectance. *International Journal of Remote Sensing*, 4(2):273–288.

Huete, A., Didan, K., Miura, T., Rodriguez, E. P., Gao, X., and Ferreira, L. G. (2002). Overview of the radiometric and biophysical performance of the MODIS vegetation indices. *Remote Sensing of Environment*, 83(1-2):195–213.

Huete, A. R. (1988). A soil-adjusted vegetation index (SAVI). *Remote Sensing of Environment*, 25:295–309.

Huete, A. R., Post, D. F., and Jackson, R. D. (1984). Soil spectral effects on 4-space vegetation discrimination. *Remote Sensing of Environment*, 15(2):155–165.

Hunt, Jr., E. R. and Rock, B. N. (1989). Detection of changes in leaf water content using near- and middle-infrared reflectances. *Remote Sensing of Environment*, 30(1):43–54.

Hunt, Jr., R. E., Daughtry, C. S. T., Eitel, J. U. H., and Long, D. S. (2011). Remote sensing leaf chlorophyll content using a visible band index. *Agronomy Journal*, 103(4):1090–1099.

Jackson, R. D., Pinter, Jr., P. J., Reginato, R. J., and Idso, S. B. (1980). Hand-Held Radiometry. Technical report, U.S. Department of Agriculture.

Jiang, Z., Huete, A. R., Didan, K., and Miura, T. (2008). Development of a two-band enhanced vegetation index without a blue band. *Remote Sensing of Environment*, 112(10):3833–3845.

Jordan, C. F. (1969). Derivation of leaf area index from quality of light on the forest floor. *Ecology*, 50(4):663–666.

Kim, M. S., Daughtry, C. S. T., Chappelle, E. W., McMurtrey, J. E., and Walthall, C. L. (1994). The use of high spectral resolution bands for estimating absorbed photosynthetically active radiation (Apar). In *Proceedings of the Sixth Symposium on Physical Measurements and Signatures in Remote Sensing*, pages 299–306, Val D'lsere, France. 17–21 January.

Le Maire, G., François, C., and Dufrêne, E. (2004). Towards universal broad leaf chlorophyll indices using PROSPECT simulated database and hyperspectral reflectance measurements. *Remote Sensing of Environment*, 89(1):1–28.

Le Maire, G., François, C., Soudani, K., Berveiller, D., Pontailler, J.-Y., Bréda, N., Genet, H., Davi, H., and Dufrêne, E. (2008). Calibration and validation of hyperspectral indices for the estimation of broadleaved forest leaf chlorophyll content, leaf mass per area, leaf area index and leaf canopy biomass. *Remote Sensing of Environment*, 112(10):3846–3864.

Lichtenthaler, H. K., Gitelson, A., and Lang, M. (1996). Non-destructive determination of chlorophyll content of leaves of a green and an aurea mutant of tobacco by reflectance measurements. *Journal of Plant Physiology*, 148(3–4):483–493.

Maccioni, A., Agati, G., and Mazzinghi, P. (2001). New vegetation indices for remote measurement of chlorophylls based on leaf directional reflectance spectra. *Journal of Photochemistry and Photobiology B: Biology*, 61(1–2):52–61.

Major, D. J., Baret, F., and Guyot, G. (1990). A ratio vegetation index adjusted for soil brightness. *International Journal of Remote Sensing*, 11(5):727–740.

Merzlyak, M. N., Gitelson, A. A., Chivkunova, O. B., and Rakitin, V. Y. (1999). Non-destructive optical detection of pigment changes during leaf senescence and fruit ripening. *Physiologia Plantarum*, 106(1):135–141.

Miller, J. R., Hare, E. W., and Wu, J. (1990). Quantitative characterization of the vegetation red edge reflectance 1. An inverted-Gaussian reflectance model. *International Journal of Remote Sensing*, 11(10):1755–1773.

Oppelt, N. and Mauser, W. (2001). The chlorophyll content of maize (zea mays) derived with the Airborne Imaging Spectrometer AVIS. In *Proceedings of the 8th International Symposium on Physical Measurements and Signatures in Remote Sensing*, pages 407–412, Aussois, France.

Oppelt, N. and Mauser, W. (2004). Hyperspectral monitoring of physiological parameters of wheat during a vegetation period using AVIS data. *International Journal of Remote Sensing*, 25(1):145–159.

Peng, Y. and Gitelson, A. A. (2011). Application of chlorophyll-related vegetation indices for remote estimation of maize productivity. *Agricultural and Forest Meteorology*, 151(9):1267–1276.

Peñuelas, J., Baret, F., and Filella, I. (1995a). Semi-empirical indices to assess carotenoids/chlorophyll-a ratio from leaf spectral reflectance. *Photosynthetica*, 31(2):221–230.

Peñuelas, J., Filella, I., Biel, C., Serrano, L., and Savé, R. (1993). The reflectance at the 950-970 nm region as an indicator of plant water status. *International Journal of Remote Sensing*, 14(10):1887–1905.

Peñuelas, J., Filella, I., Lloret, P., Muñoz, F., and Vilajeliu, M. (1995b). Reflectance assessment of mite effects on apple trees. *International Journal of Remote Sensing*, 16(14):2727–2733.

Peñuelas, J., Gamon, J. A., Fredeen, A. L., Merino, J., and Field, C. B. (1994). Reflectance indices associated with physiological changes in nitrogen- and water-limited sunflower leaves. *Remote Sensing of Environment*, 48(2):135–146.

Peñuelas, J., Pinol, J., Ogaya, R., and Filella, I. (1997). Estimation of plant water concentration by the reflectance Water Index WI (R900/R970). *International Journal of Remote Sensing*, 18(13):2869–2875.

Pinty, B. and Verstraete, M. M. (1992). GEMI: a non-linear index to monitor global vegetation from satellites. *Vegetatio*, 101(1):15–20.

Qi, J., Chehbouni, A., Huete, A. R., Kerr, Y. H., and Sorooshian, S. (1994). A modified soil adjusted vegetation index. *Remote Sensing of Environment*, 24:119–126.

Reyniers, M., Walvoort, D. J. J., and De Baardemaaker, J. (2006). A linear model to predict with a multi-spectral radiometer the amount of nitrogen in winter wheat. *International Journal of Remote Sensing*, 27(19):4159–4179.

Richardson, A. J. and Wiegand, C. L. (1977). Distinguishing vegetation from soil background information. *Photogrammetric Engineering & Remote Sensing*, 43(12):1541–1552.

Rondeaux, G., Steven, M., and Baret, F. (1996). Optimization of soil-adjusted vegetation indices. *Remote Sensing of Environment*, 55:95–107.

Roujean, J. and Breon, F. (1995). Estimating PAR absorbed by vegetation from bidirectional reflectance measurements. *Remote Sensing of Environment*, 51(3):375–384.

Rouse, Jr., J. W., Haas, R. H., Schell, J. A., and Deering, D. W. (1973). Monitoring the vernal advancement and retrogradation (green wave effect) of natural vegetation, Program Report RSC 1978-1. Technical report, Remote Sensing Center, Texas A&M University, College Station, 93p. (NTIS no. E73-10693).

Serrano, L., Peñuelas, J., and Ustin, S. L. (2002). Remote sensing of nitrogen and lignin in Mediterranean vegetation from AVIRIS data: Decomposing biochemical from structural signals. *Remote Sensing of Environment*, 81(2–3):355–364.

Sims, D. A. and Gamon, J. A. (2002). Relationships between leaf pigment content and spectral reflectance across a wide range of species, leaf structures and developmental stages. *Remote Sensing of Environment*, 81(2–3):337–354.

Sonobe, R. and Wang, Q. (2017). Towards a universal hyperspectral index to assess chlorophyll content in deciduous forests. *Remote Sensing*, 9:191.

Tucker, C. J. (1979). Red and photographic infrared linear combinations for monitoring vegetation. *Remote Sensing of Environment*, 8:127–150.

Vincini, M., Frazzi, E., and D'Alessio, P. (2006). Angular dependence of maize and sugar beet VIs from directional CHRIS/Proba data. In *Proceedings of the 4th ESA CHRIS PROBA Workshop*.

Vincini, M., Frazzi, E., and D'Alessio, P. (2008). A broad-band leaf chlorophyll vegetation index at the canopy scale. *Precision Agriculture*, 9(5):303–319.

Vogelmann, J. E., Rock, B. N., and Moss, D. M. (1993). Red edge spectral measurements from sugar maple leaves. *International Journal of Remote Sensing*, 14(8):1563–1575.

Wu, C., Niu, Z., Tang, Q., and Huang, W. (2008). Estimating chlorophyll content from hyperspectral vegetation indices: Modeling and validation. *Agricultural and Forest Meteorology*, 148(8-9):1230–1241.

Zarco-Tejada, P. J., Berjón, A., López-Lozano, R., Miller, J. R., Martín, P., Cachorro, V., González, M. R., and De Frutos, A. (2005). Assessing vineyard condition with hyperspectral indices: Leaf and canopy reflectance simulation in a row-structured discontinuous canopy. *Remote Sensing of Environment*, 99(3):271–287.

Zarco-Tejada, P. J., Miller, J. R., Mohammed, G. H., and Noland, T. L. (2000a). Chlorophyll fluorescence effects on vegetation apparent reflectance: I. Leaf-level measurements and model simulation. *Remote Sensing of Environment*, 74(3):582–595.

Zarco-Tejada, P. J., Miller, J. R., Mohammed, G. H., Noland, T. L., and Sampson, P. H. (2000b). Chlorophyll fluorescence effects on vegetation apparent reflectance: II. Laboratory and airborne canopy-level measurements with hyperspectral data. *Remote Sensing of Environment*, 74(3):596–608.

Zarco-Tejada, P. J., Miller, J. R., Mohammed, G. H., Noland, T. L., and Sampson, P. H. (2001a). Estimation of chlorophyll fluorescence under natural illumination from hyperspectral data. *International Journal of Applied Earth Observation and Geoinformation*, 3(4):321–327.

Zarco-Tejada, P. J., Miller, J. R., Noland, T. L., Mohammed, G. H., and Sampson, P. H. (2001b). Scaling-up and model inversion methods with narrowband optical indices for chlorophyll content estimation in closed forest canopies with hyperspectral data. *IEEE Transactions on Geoscience and Remote Sensing*, 39(7):1491–1507.

Zarco-Tejada, P. J., Pushnik, J. C., Dobrowski, S., and Ustin, S. L. (2003a). Steady-state chlorophyll a fluorescence detection from canopy derivative reflectance and double-peak red-edge effects. *Remote Sensing of Environment*, 84(2):283–294.

Zarco-Tejada, P. J., Rueda, C. A., and Ustin, S. L. (2003b). Water content estimation in vegetation with MODIS reflectance data and model inversion methods. *Remote Sensing of Environment*, 85(1):109–124.
